# Altered proteome turnover and remodeling by short-term caloric restriction or rapamycin rejuvenate the aging heart

**DOI:** 10.1111/acel.12203

**Published:** 2014-02-25

**Authors:** Dao-Fu Dai, Pabalu P Karunadharma, Ying A Chiao, Nathan Basisty, David Crispin, Edward J Hsieh, Tony Chen, Haiwei Gu, Danijel Djukovic, Daniel Raftery, Richard P Beyer, Michael J MacCoss, Peter S Rabinovitch

**Affiliations:** 1 Department of Pathology, University of Washington1959 NE Pacific Ave, Seattle, WA, 98195, USA; 2 Department of Genome Sciences, University of Washington1959 NE Pacific Ave, Seattle, WA, 98195, USA; 3 Department of Anesthesiology and Pain Medicine, University of Washington1959 NE Pacific Ave, Seattle, WA, 98195, USA; 4 Department of Environmental Health, University of Washington1959 NE Pacific Ave, Seattle, WA, 98195, USA

**Keywords:** caloric restriction, cardiac aging, dynamics, proteomics, rapamycin

## Abstract

Chronic caloric restriction (CR) and rapamycin inhibit the mechanistic target of rapamycin (mTOR) signaling, thereby regulating metabolism and suppressing protein synthesis. Caloric restriction or rapamycin extends murine lifespan and ameliorates many aging-associated disorders; however, the beneficial effects of shorter treatment on cardiac aging are not as well understood. Using a recently developed deuterated-leucine labeling method, we investigated the effect of short-term (10 weeks) CR or rapamycin on the proteomics turnover and remodeling of the aging mouse heart. Functionally, we observed that short-term CR and rapamycin both reversed the pre-existing age-dependent cardiac hypertrophy and diastolic dysfunction. There was no significant change in the cardiac global proteome (823 proteins) turnover with age, with a median half-life 9.1 days in the 5-month-old hearts and 8.8 days in the 27-month-old hearts. However, proteome half-lives of old hearts significantly increased after short-term CR (30%) or rapamycin (12%). This was accompanied by attenuation of age-dependent protein oxidative damage and ubiquitination. Quantitative proteomics and pathway analysis revealed an age-dependent decreased abundance of proteins involved in mitochondrial function, electron transport chain, citric acid cycle, and fatty acid metabolism as well as increased abundance of proteins involved in glycolysis and oxidative stress response. This age-dependent cardiac proteome remodeling was significantly reversed by short-term CR or rapamycin, demonstrating a concordance with the beneficial effect on cardiac physiology. The metabolic shift induced by rapamycin was confirmed by metabolomic analysis.

## Introduction

Caloric restriction (CR) extends lifespan in a wide variety of organisms ranging from yeast to mice and may attenuate several age-related diseases including diabetes, cancer, and neurodegenerative disease (Weindruch *et al*., 1986; Speakman & Mitchell, 2011). Long-term caloric restriction has been shown to ameliorate age-associated cardiac hypertrophy and diastolic dysfunction (Taffet *et al*., 1997; Niemann *et al*., 2010; Shinmura *et al*., 2011) as well as cardiomyopathy in rodents and in monkeys (Maeda *et al*., 1985; Colman *et al*., 2009). Although short-term caloric restriction improved ischemic tolerance in the aged heart (Shinmura *et al*., 2005), the beneficial effect of short-term caloric restriction on cardiac hypertrophy and diastolic dysfunction is not well understood.

Several nutrient sensing pathways have been implicated in the beneficial effect of caloric restriction on aging process (Kenyon, 2010); however, interest in the mechanistic target of rapamycin (TOR) has increased following the demonstration that long-term rapamycin (RP) treatment, initiated at 9 or 18 months of age, extends lifespan in murine models (Harrison *et al*., 2009; Miller *et al*., 2011). Mechanistic target of rapamycin (mTOR) regulates metabolism and cellular growth by sensing nutrient status and the growth factor signaling. The mechanisms by which TOR exerts its effects on aging involve the modulation of protein synthesis (Steffen *et al*., 2008), ribosomal biogenesis, and autophagy through mTOR complex 1 (mTORC1) and downstream targets ribosomal S6 kinase (S6K) and the translational repressor 4E-BP1 (Guertin & Sabatini, 2007; Sengupta *et al*., 2010). Mice with deletion of S6K1 have increased lifespan and resistance to age-related pathologies (Selman *et al*., 2009), and activation of 4E-BP has been shown to mediate the lifespan extension effect of dietary restriction in Drosophila (Zid *et al*., 2009). Furthermore, 4E-BP has been shown to act downstream of TOR to modulate cardiac functional aging in Drosophila (Wessells *et al*., 2009).

Although the inhibition of mTOR is well known to ameliorate pressure-overload-induced cardiac hypertrophy (McMullen *et al*., 2004), the effect of RP on murine cardiac aging is less established. Both CR and RP have been shown to suppress protein synthesis and increase autophagy. We have recently developed a novel method to measure proteome dynamics using ^2^H_3_ leucine heavy isotope labeling (Hsieh *et al*., 2012). In this study, we applied this novel method to investigate the effect of short-term CR or RP on cardiac aging, proteome dynamics, and protein abundance.

## Results

### Experimental diet and deuterated-leucine labeling

**Figure 1:**
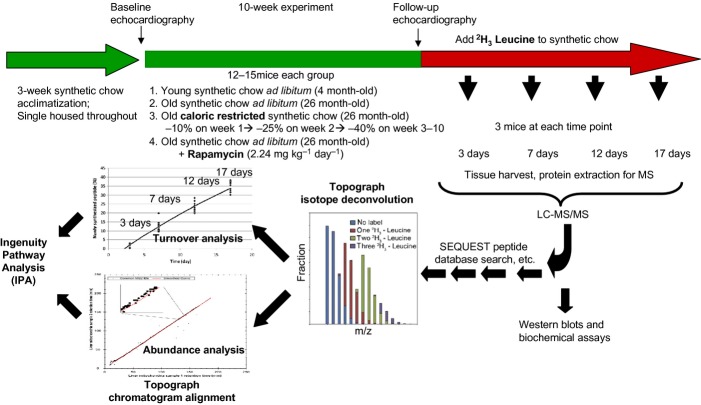
Summary of experimental design. After 3 weeks on synthetic chow diet for acclimatization, young (4 month old) and old (26 month old) female mice had baseline echocardiography and were placed on a synthetic diet *ad libitum* (control group), 40% caloric restriction (progressively over 3 weeks), or *ad libitum* plus microencapsulated rapamycin 2.24 mg kg^−1^day^−1^. After 10 weeks, echocardiography was repeated and the mice were switched to the same synthetic diet but with ^2^H_3_-leucine fully substituted for normal leucine. Tissue from 3 mice per treatment were harvested 3, 7, 12, and 17 days thereafter. Protein extraction was followed by LC-MS/MS. Topograph was applied to calculate the fraction of newly synthesized peptides, as well as the relative abundance of all isotopomers. The percentage newly synthesized peptides for each protein were plotted for the triplicate mice at each time point to derive the rate constant and half-lives based on first order kinetics. For abundance analysis, Topograph aligned the chromatograms to normalize the recovery times of the corresponding ions in each sample and obtain the areas under the curve for every peptide identified in any one sample. (see method S1 for further detail).

The experimental design is summarized in Fig. 1. Mice were acclimatized to synthetic diet for 3 weeks. During the first week, there was a 7% decline of mouse body weight, after which it stabilized. At week 3, 4- and 26-month-old mice were fed *ad libitum* synthetic chow control diet (CL), caloric restricted (CR), or *ad libitum* plus rapamycin (RP) for 10 weeks. Rapamycin was used at the concentration and formulation previously shown by the NIA Interventions Testing Program to extend mouse lifespan (Harrison *et al*., 2009). Old mice fed caloric-restricted synthetic chow diet lost 25% of body weight over the first 3 weeks, after which weight stabilized (Fig. S1). The body weight of old CL and old RP remained unaltered throughout the 10-week period; young mice CL gained 7.8% of body weight, normal growth for age. Mice are then placed on a deuterated-leucine diet while maintaining CR, RP, or CL conditions. At 4 time points thereafter, mice were euthanized and cardiac proteins analyzed by LC-MCS/MS. Topograph software was used to deconvolute the mass spectra to determine the isotope distributions of deuterated label in each peptide and used this to calculate the precursor-pool-adjusted turnover rate of proteins (Fig. S2, Table S1, Method S1)(Hsieh *et al*., 2012).

### Reversal of aging cardiac dysfunction by short-term rapamycin or CR

**Figure 2:**
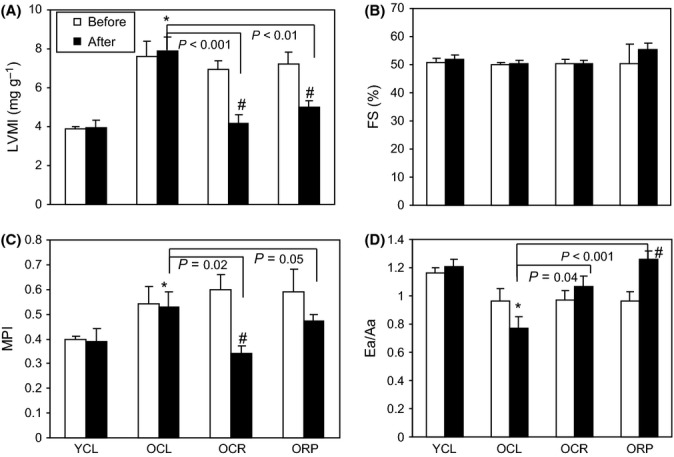
Echocardiography. (A) Left ventricular mass index (LVMI) is significantly higher in old hearts at baseline (25 months old), when compared to young hearts (3 months old), indicating age-dependent left ventricular hypertrophy. After 10 weeks of CR or RP in old mice, LVMI is significantly lower than old control mice (*P* < 0.001 and *P* = 0.004, respectively). It is also significantly reversed when compared with the baseline echocardiography of each mouse (*P* < 0.01 for both CR and RP), indicating reversal of age-dependent ventricular hypertrophy. (B) Fractional shortening does not significantly change with age or treatment group. (C). Myocardial performance index (MPI) significantly worsened (increased) in old mice at baseline. CR or RP significantly improved the MPI in old hearts when compared with control mice. (*P* = 0.02 and 0.05, respectively). (D) Diastolic function measured by tissue Doppler imaging Ea/Aa significantly declined in old hearts. While old CL mice have progressive decline of Ea/Aa after 10 weeks, treatment with CR or RP significantly increased Ea/Aa. CL: *ad libitum* control diet, and CR: caloric restriction. *P < 0.05 vs. YCL; ^#^*P* < 0.05 versus pretreatment. *n* = 5–8.

At baseline, the aged heart phenotype in mice recapitulates the age-related changes of the human heart (Dai *et al*., 2009), including left ventricular hypertrophy and impairment of myocardial performance and diastolic dysfunction, as measured by echocardiography (Fig. 2). Old mice fed *ad libitum* with a control diet (OCL) had an approximately twofold increase in left ventricular mass index (LVMI, Fig. 2A) compared to young controls (YCL), indicating left ventricular hypertrophy. There was no significant change in systolic function measured by fractional shortening (Fig. 2B). The myocardial performance index (MPI), a sensitive marker of systolic and diastolic function, significantly increased with age (Fig. 2C), indicating a greater fraction of inefficient time spent without ejection in systole. Diastolic function measured by tissue Doppler Ea/Aa significantly decreased in OCL mice (Fig. 2D), falling below the value of 1.0 which is the definition of diastolic dysfunction. While 10 weeks of control diet did not affect any of the above parameters, OCR mice demonstrated a large decline of LVMI, falling to a level comparable to YCL young mice and significantly lower than that of OCL mice (*P* < 0.001, Fig. 2A). This was closely recapitulated by 10-week RP (*P* = 0.004). The worsening of myocardial performance (MPI) in old mice was also significantly reversed by both CR and RP (*P* = 0.016 and 0.05, respectively, Fig. 2C), to levels comparable to YCL. Likewise, the age-related decline in diastolic function was significantly improved by both CR and RP (*P* = 0.04 and *P* < 0.001, respectively, Fig. 2D) as compared to OCL. These findings suggest that short-term CR or RP effectively induce regression of age-related left ventricular hypertrophy, amelioration of myocardial performance, and reversal of diastolic dysfunction.

### Measurement of global cardiac proteome dynamics in aged heart and its modification by short-term rapamycin or CR

Caloric restriction and RP are associated with the reduction in mTOR signaling and reduced protein synthesis (Laplante & Sabatini, 2012). Alterations in mTOR signaling observed in this study included a significant increase in S6 ribosomal protein phosphorylation in the aged heart, which was significantly attenuated by both CR and RP (Fig. S3). There was no significant change of 4EBP1 phosphorylation with aging, CR, or RP (Fig. S3).

**Figure 3:**
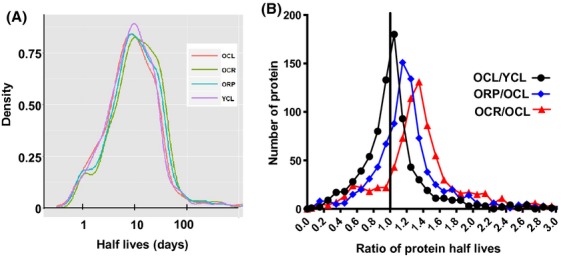
Global proteomic half-lives. (A) Histograms of half-lives (days). YCL versus OCL: n.s., OCL versus OCR *P* < 0.001; OCL versus ORP, *P* = 0.038; OCR versus OR, *P* = 0.08. (B) Histograms of half-life ratios for two-group comparisons. *P* = 0.09 for YCL/OCL >1, *P* < 0.001 for both OCR/OCL and ORP/OCL >1.

The proteomics analysis of turnover rates found a wide distribution in protein half-lives (Fig. 3A, Table S1). The median half-lives of 823 proteins in the YCL heart were 9.1 days, similar to 8.8 days in the OCL heart. Short-term CR significantly increased proteome half-lives by approximately 30% (11.4 days), when compared with OCL (*P* < 0.001). Short-term RP also significantly increased old heart proteome half-lives by 12% to a median of 9.8 days (*P* = 0.04 compared to OCL).

A more sensitive analysis of treatment differences is performed by comparing the ratio of half-lives of each individual protein in two treatment groups. The pairwise comparison in Fig. 3B shows that the median half-lives of OCL are 10% longer than YCL (*P* = 0.09). Both short-term CR or RP significantly increased the half-lives of old heart proteins: OCR are 45% longer than OCL, and ORP are 27% longer than OCL (both *P* < 0.001, Fig. 3B).

**Figure 4:**
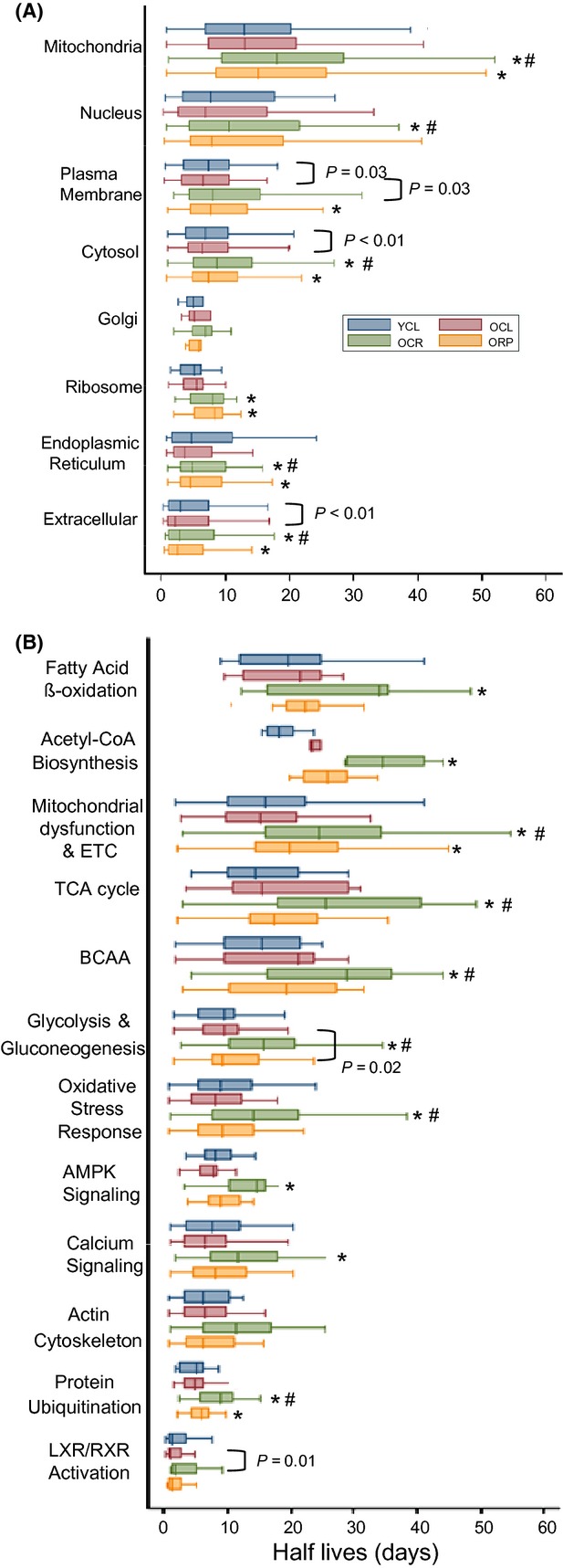
Protein half-lives (days) stratified by (A) cellular compartment, (B) most significant canonical pathways in ingenuity pathway analysis. *P < 0.01 for OCR versus OCL or ORP versus OCL, ^#^*P* < 0.01 for OCR versus ORP.

To describe the variation of protein half-lives in different cellular compartments, we grouped the components proteins of 8 chief compartments. As shown in Fig. 4A, extracellular proteins have the shortest half-lives in YCL, with the median of 3.4 days, and mitochondrial proteins have the longest half-lives, with the median of 13.2 days, with other compartments falling in between. The half-lives of plasma membrane and cytosolic proteins in OCL are approximately 8% and approximately 17% shorter, respectively, than those of YCL (*P* = 0.03 and *P* < 0.01, Fig. 4A). Caloric restriction significantly increased protein half-lives in old mice in all compartments except for Golgi; the magnitude of increase are as follows: 42.8% for mitochondria, 38% for cytosol, 39.5% for extracellular, 46% for ER, 47.3% for ribosome, 74.8% for nucleus, and 75.4% for plasma membrane (*P* < 0.01 for all except for nucleus, *P* = 0.03, Fig. 4A). ORP mice had significantly increased protein half-lives in mitochondria (27.8%), plasma membrane (32.1%), cytosol (23.2%), and ribosome (54.0%) compartments (*P* < 0.01 for all, Fig. 4A).

### Pathway analysis of proteome dynamics and remodeling in aged heart and the effect of CR and rapamycin

To identify protein pathways that are differentially regulated by CR and RP, we used ingenuity pathway analysis (IPA). A complete list of the significantly altered protein abundances with aging and their canonical pathways are given in Table S2. There were 589 proteins whose turnover was significantly changed in one or more group comparison (q < 0.05). The 12 pathways that were most significantly altered in protein turnover are shown in Fig. 4B. Proteins involved in signaling have the shortest half-lives, including proteins in LXR/RXR activation, protein ubiquitination, actin cytoskeleton, calcium, and AMPK signaling as well as oxidative stress response pathways. Proteins involved in metabolism have longer half-lives, including those involved in glycolysis/gluconeogenesis, branched-chain amino acid (BCAA) degradation, TCA cycle, mitochondrial dysfunction and electron transport chain (ETC), acetyl CoA, and fatty acid beta oxidation. Caloric restriction significantly increased the half-lives of proteins in all these IPA pathways by 48–94% (*P* ≤ 0.01 for all), except for actin cytoskeleton. Rapamycin in old mice significantly increased half-lives only for mitochondrial dysfunction (24.9%, *P* < 0.01), glycolysis/gluconeogenesis (12.8%, *P* = 0.02), and protein ubiquitin pathways (20.3%, *P* < 0.01), each to a much lesser extent than the effect of CR.

**Figure 5:**
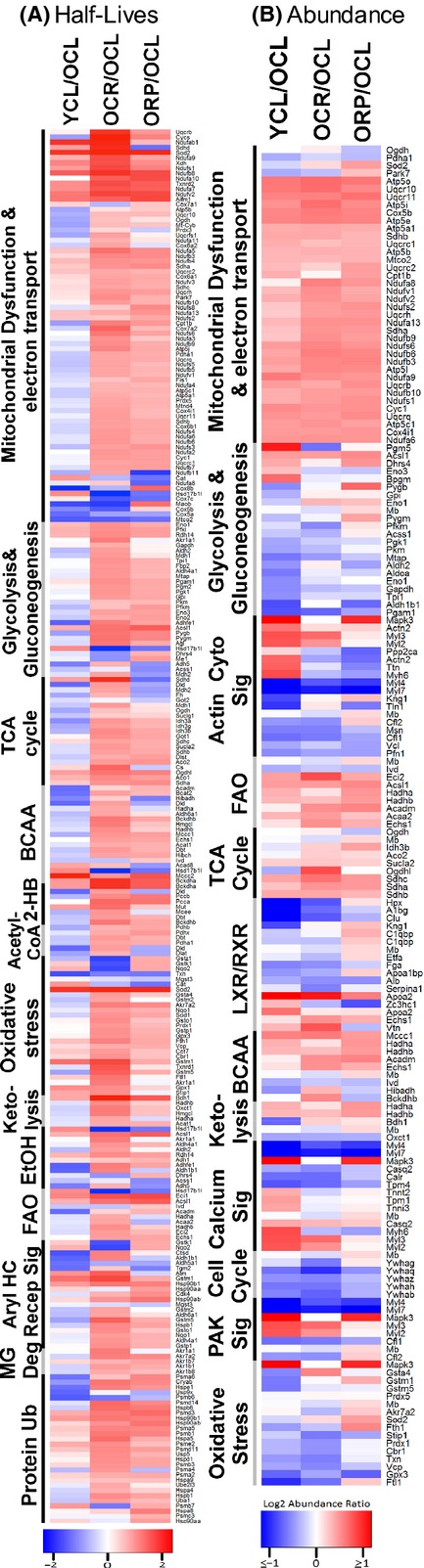
Heat maps of (A) half-life differences, (B) abundance differences for YCL/OCL, OCR/OCL, and ORP/OCL comparisons, ordered by the rank of significance of differ-ences in ingenuity pathway analysis. In part A, red indicates longer and blue indicates shorter half-lives. In part B, red indicates higher and blue indicates lower abundance. The component protein IDs are listed in Tabls S4 for abundance difference and Table S5 for turnover differences. FAO: fatty acid oxidation, BCAA: branched-chain amino acid metabolism; 2-HB: 2-oxobutanoate; MG: methylglyoxal; and Ub: Ubiquitination.

To examine and compare differences between groups for protein turnover (Table S1) and protein abundance (Table S3), we created heat map of proteins in IPA canonical pathways, ordered by the significance of the IPA pathway (Fig. 5). Figure 5A displays the heat maps of the protein half-life ratios. Compared with OCL, YCL has shorter half-lives for 58% of proteins involved in mitochondrial dysfunction and ETC, and longer half-lives for the remainder. Consistent with Fig. 3, short-term CR in old age hearts confers significantly longer half-lives, and this is true for the great majority of the proteins. Interestingly, the heat map shows that RP closely recapitulates CR in increasing mitochondrial dysfunction and ETC protein half-lives, despite its weaker effect than CR overall (Fig. 3).

Figure 5B shows the heat map of protein abundance ratios between groups. There were 327 proteins whose abundance was significantly changed in one or more group comparison (q < 0.05). Proteins involved in mitochondrial dysfunction and ETC are significantly more abundant in YCL as compared to OCL, indicating a significant decrease in these proteins with age; this difference is concordant for the great majority of proteins of mitochondrial dysfunction and ETC CR and RP in old mice significantly reverse the aging difference, restoring protein abundances in this pathway to levels very comparable to YCL. The increased mitochondrial protein abundance by CR and RP was not associated with increase in mitochondrial copy number (Fig. S4A), mRNA levels of mitochondrial biogenesis regulator, PGC1α, or its downstream transcriptional factor, TFAM (Fig. S4B and C). Similar effects on proteome, although not as consistent across all proteins, were seen in proteins involved in fatty acid beta oxidation and TCA cycle pathways. Conversely, YCL has significantly lower abundance of the majority of proteins involved in glycolysis/gluconeogenesis, oxidative stress response, and cell cycle than OCL. Caloric restriction is effective in reducing abundance of most of these proteins in these three pathways in old mice toward the youthful levels; in contrast, RP had mixed effects within all but the cell cycle pathway, which was effectively restored to the young phenotype. The decrease in protein abundance in glycolysis together with the increased abundance in fatty acid oxidation by RP in old mice suggested that RP shifts substrate utilization in old heart from glucose to fatty acid, similar to previous observation from skeletal muscle cells (Sipula *et al*., 2006).

**Figure 6:**
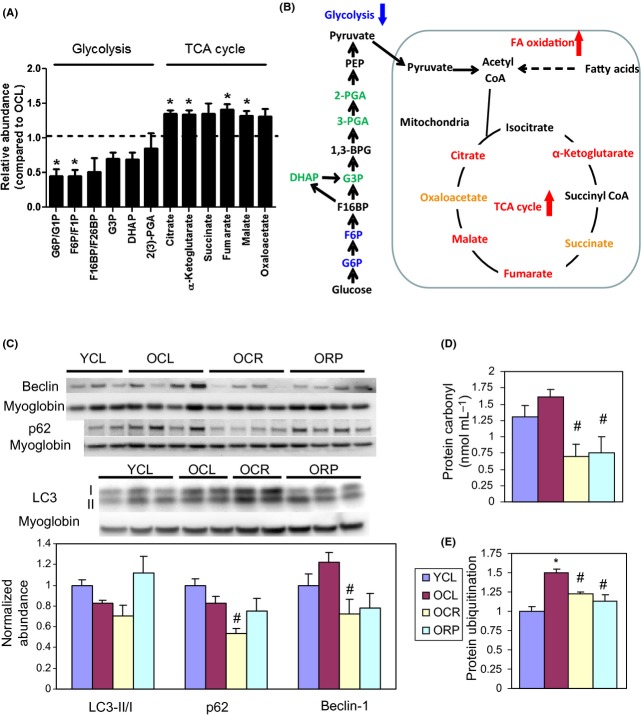
Metabolic profiling and biochemical assay. (A) Relative abundance of the substrates in the glycolytic pathway and TCA cycle in ORP compared to OCL by targeted metabolic profiling. When compared with OCL heart, ORP hearts have significantly lower glucose-6-phosphate and fructose-6-phosphate (both are glycolytic metabolites), and significantly higher a-ketoglutarate, fumarate, malate, and citrate (all are TCA cycle metabolites). *P < 0.05 compared with OCL. See Table S6 for numerical data. (B) A schematic diagram summarizing the changes in metabolism by rapamycin in old heart. (C) Western blots of autophagic markers show no significant change of LC3 II/I, p62, or beclin-1 in cardiac aging. However, OCR has significantly lower p62 than that in OCL. ^#^*P* < 0.05 compared with OCL. (D) Both CR and RP significantly reduce the age-dependent increase in protein carbonyls (nmol mL^−1^). ^#^*P* < 0.05 compared with OCL. (E). Both CR and RP significantly reduce the age-dependent increase in protein ubiquitination.*P < 0.05 compared with YCL and ^#^*P* < 0.05 compared with OCL. *n* = 3–8. G6P: glucose 6-phosphate; G1P: glucose 1-phosphate; F6P: fructose 6-phosphate; F1P: fructose 1-phosphate; F16BP: fructose 1,6-bisphosphate; F26BP: fructose 2,6-biphosphate; G3P: glyceraldehyde 3-phosphate; DHAP:dihydroxyacetone phosphate; 2(3)-PGA: 2- or 3-phosphoglycerate; and PEP: phosphoenolpyruvate. Isomers of same molecular weight, that is, G6P versus G1P, F6P versus F1P, and F16BP versus F26BP, were not distinguishable by the LC-MS/MS-based metabolic profiling method.

To confirm this observation, we performed a targeted metabolic profiling of aged heart tissue in OCL and ORP mice. Figure 6A,B, and Table S6 show that when compared with OCL, ORP has significantly higher TCA cycle metabolites: α-ketoglutarate, fumarate, malate, and citrate, with ratios of 1.32–1.41 (*P* = 0.001–0.02); and higher succinate and oxaloacetate with borderline significance (*P* = 0.08–0.09). For glycolytic intermediates, ORP has significantly lower glucose-6 phosphate (ratio: 0.45, *P* = 0.05) and fructose-6 phosphate (ratio: 0.45, *P* = 0.05), and nonsignificant decrease in the remaining glycolytic intermediates, when compared with OCL (Fig. 6A,B, Table S6).

### Alterations in protein autophagy, ubiquitination and damage by rapamycin and caloric restriction

We observed no significant change in LC3 II/I, p62, and beclin-1, markers of autophagy, in cardiac aging (Fig. 6C). However, OCR substantially reduced p62 (*P* < 0.05). Consistent with our previous observation (Dai *et al*., 2009), there is an increase in oxidative damage in old hearts, as shown by a higher level of protein carbonylation. This increase is significantly reversed by CR and RP (Fig. 6D). The ubiquitin-proteosomal pathway is one of the major mechanisms for degradation of damaged proteins. Figure 6E shows that protein ubiquitination significantly increased in OCL hearts and this was significantly reversed by CR and RP. The reduction in protein damage and ubiquitination is consistent with the slower rate of protein turnover seen in CR- and RP-treated old hearts.

## Discussion

This study demonstrates that short-term, 10-week treatment with CR or RP, initiated at old age, reverses many of the pre-existing functional deficits of cardiac aging, rather than just slowing down the aging process. This rejuvenation of the heart includes reversal of age-dependent cardiac hypertrophy, diastolic dysfunction, and impairment of myocardial performance (Fig. 2). In parallel with the effect on cardiac physiology, short-term RP closely recapitulate CR to restore key components of the mitochondrial proteome to youthful levels, including those involved in mitochondrial function and electron transport chains, fatty acid oxidation, and TCA cycles (Fig. 5A, Table S3). In some other pathways, such as glycolysis/gluconeogenesis, oxidative stress response, and cell cycle, CR was effective in reducing abundance of most of these proteins in old mice toward the youthful levels; in contrast, RP had mixed effects and reversed to a smaller extent.

This study is also the first to elucidate the global dynamics of the aging cardiac proteome, calculating the turnover rate of 823 proteins (containing at least one leucine) in a single experiment (Figs 3–5B, Table S1). It was carried out by analyzing LC-MS data with the Topograph software program. Topograph deconvolutes the isotopologue distributions to determine the amount of heavy label in the amino acid precursor pool (Fig. S2 and S5) and from this, correctly calculates the percentage of peptides that are newly synthesized (18). This novel method of turnover analysis does, however, have several limitations. First, it requires feeding mice with a synthetic diet, not regular mouse chow. Second, the analysis of the protein turnover is limited to proteins containing at least one leucine. Some important proteins without leucine, such as collagen (which increased in age-dependent cardiac fibrosis), were therefore not included in the turnover analysis (these were, however, included in the abundance analysis). Third, the errors in half-life measurement of proteins with very short (<3 days) or very long (>50 days) half-lives are higher using our current labeling protocol (Fig. S6).

The proteome in the aged heart is notable for the significant decline in proteins involved in mitochondrial function, ETC, and fatty acid metabolism. Conversely, aged hearts have significantly increased protein abundance in glycolysis/gluconeogenesis, acute phase response, and LXR/RXR and other signaling pathways. This remodeling is similar to our previous observations in pressure-overload-induced heart failure, including the decline in proteins in mitochondrial function, ETC, and fatty acid oxidation, as well as an increase in proteins of glycolysis/gluconeogenesis pathways (Dai *et al*., 2012). This switch in preferential substrate utilization from more efficient fatty acid oxidation in the mitochondrial-rich normal heart to less efficient glycolysis/glucose oxidation in failing hearts has been extensively described in human and animal models of heart failure (Kolwicz & Tian, 2009). Although aged hearts do not demonstrate the full-blown phenotypes of end-stage heart failure, the concomitant changes in both functional performance (Fig. 2) and metabolic proteomes (Fig. 5B) are obvious. The age-related decline in proteins in mitochondrial function, ETC, and fatty acid metabolism is reversed by 10-week CR or RP treatment. As the increased mitochondrial protein abundance following CR and RP occurred without increasing mitochondrial biogenesis and mtDNA copy number, this suggests that the proteomic remodeling may be due to increased abundance of these mitochondrial proteins that resulted from better preserved protein quality and slower turnover. These findings emphasize the crucial roles of mitochondria in aging and are consistent with previous studies showing that preservation of mitochondria by overexpression of catalase targeted to mitochondria (Dai *et al*., 2009) or by inhibition of insulin signaling (Anderson *et al*., 2009; Zhang *et al*., 2012) attenuates age-dependent cardiomyopathy (Li *et al*., 2008).

Rapamycin has been shown to reduce glycolysis and facilitate a switch in fat metabolism by increasing fatty acid oxidation in skeletal muscle (Sipula *et al*., 2006). By using a targeted metabolomics approach, we confirm that RP significantly reduces glycolytic metabolites and significantly increases TCA cycle metabolites (Fig. 6A,B and Table S6) in old mouse hearts. The increased TCA cycle metabolites despite the lower glycolytic intermediates suggest increased proportion of TCA cycle substrate are coming from fatty acid oxidation. The concerted effect of RP on these metabolic pathways are consistent with the changes in protein levels: increase in proteins involved in TCA cycle and fatty acid oxidation, and decrease in proteins involved in glycolysis (Fig. 5B). In contrast to this beneficial effect, RP treatment has been reported to induce detrimental metabolic phenotypes, including insulin resistance, hyperlipidemia, and glucose intolerance, all of which are associated with shortened lifespan. Some of these effects may be due to inhibition of mTORC2 (Lamming *et al*. 2012). This RP paradox may be reconciled, at least in part, by the differential effects of acute versus short-term RP treatment; as recently reported, 2 or 6 weeks of RP result in detrimental metabolic alterations, while 20-week RP treatment conferred beneficial metabolic profiles, including improved insulin sensitivity and increased oxygen consumption (Fang *et al*., 2013). The current study using a 10-week protocol of CR or RP is consistent with these 20-week short-term effects, as it ameliorated the age-dependent decline in metabolic proteins and reversed the adverse metabolic remodeling in the aged heart. The involvement of mTOCR2 in this metabolic changes will require further investigation.

Two recent studies have reported contradictory results of RP on the aging mouse heart. Neff *et al*. reported that chronic RP treatment for 1 year initiated at late life (20–22 months) reduced aging heart’s increased dimensional measures, but did not show any effect on systolic function, mean flow velocity or pressure gradient across aortic, and pulmonic valves in male mice (Neff *et al*., 2013). Flynn *et al*. reported that short-term RP treatment for 12 weeks initiated at late life (24 months) attenuates age-related cardiac hypertrophy and marginally improves systolic function in female mice, with a reduction in age-related inflammation (Flynn *et al*., 2013). The discrepancy observed in these studies could be explained by both difference in duration of treatment and gender differences. It is notable that neither of these studies have reported the effect of RP on diastolic dysfunction, which is one of the most prominent findings in aged hearts of mice and humans (Lakatta & Levy, 2003; Dai *et al*., 2009). In this study, CR and RP improve diastolic function in old mice; this reversal of diastolic dysfunction is potentially related to better preserved mitochondria (Dai *et al*., 2009). It is well known that diastolic heart failure (heart failure with preserved ejection fraction) is the predominant type in elderly human patients without structural or coronary heart disease and that there is no effective treatment to date. Hence, our observation that short-term CR or RP ameliorates age-dependent diastolic dysfunction is clinically relevant and supports the potential of therapeutics development for the treatment of age-associated diastolic heart failure.

Our study further shows that both CR and RP decreased protein oxidative damage in the aged heart (Fig. 6D). This is consistent with the observed decrease in protein ubiquitination (Fig. 6E), the rate-limiting step for the proteosomal degradation of oxidized proteins (Breusing & Grune, 2008). Also consistent with this, CR and RP decreased the proteomics turnover rate globally (extended half-lives), and this is especially true for mitochondrial proteins. Whether CR increases or decreases proteomics turnover has previously been a topic of debate, and the effect of RP on protein turnover has not been well established. Caloric restriction and RP induce autophagy, which would suggest increased protein turnover. However, Miller and colleagues recently reported that CR and RP do not alter overall protein synthesis in heart (Drake *et al*., 2013; Miller *et al*., 2013). Price *et al*., on the other hand, showed that CR reduces protein turnover of 80% of proteins in liver (Price *et al*., 2012). Our study showed that CR greatly reduced protein turnover in the heart, which is consistent with the study in liver (Price *et al*., 2012). We showed that RP also reduced turnover in the heart. The absence of CR or RP effect on protein synthesis rate in the studies by Miller and colleagues is likely due to the limitation of the method of ‘bulk measurement’. Bulk analysis of overall protein labeling level is skewed by the synthesis rate of a few highly abundant structural proteins, whereas in our study and the study by Price *et al*., measurements of average protein turnover changes in large numbers of individual proteins are not weighted toward the most abundant structural proteins.

Both CR and RP have been shown to induce autophagy (Laplante & Sabatini, 2012), which is consistent with our unpublished observation that RP for 1–2 weeks induced autophagy (data not shown). Interestingly, the current study demonstrates that at the end of the 10-week CR or RP treatment does not increase the levels of autophagic markers, LC3-II/I, and beclin-1 (Fig. 6C). The absence of persistent induction of autophagy by CR or RP after 10 weeks undoubtedly relates to the temporal response of protein homeostasis. Upregulation of autophagy is generally observed acutely and allows damaged or aggregated proteins to be recycled, reaching a new equilibrium with proteins of better quality by 10 weeks. Likewise, a recent study shows that constitutive activation of TORC1 decreased translational fidelity and increased amino acid misincorporation errors and that RP reversed these effects as it slowed the rate of ribosomal elongation in mouse embryonic fibroblasts (Conn & Qian, 2013). A further mechanism of preservation of protein quality by CR depends on mitochondrial SIRT3-mediated antioxidant effects, as reported by Someya *et al*. using mouse model of age-dependent sensorineural hearing loss. In response to CR, SIRT3 directly deacetylates and activates mitochondrial isocitrate dehydrogenase 2, leading to increased NADPH levels and an increased ratio of reduced-to-oxidized glutathione in mitochondria, thereby enhancing the mitochondrial glutathione antioxidant defense system (Someya *et al*., 2010). Hence, 10-week CR or RP results in improved protein quality and decreased protein damage, with resulting longer protein half-lives and reduced protein degradation. Indeed, CR substantially reduced p62 in OCR hearts, while the RP effect is modest. p62 has been shown to recognize ubiquitinated protein aggregates and target them to the autophagosome or proteasome for degradation (Seibenhener *et al*., 2004; Yao, 2010). The reduced protein damage in OCR may explain the reduced p62 levels without increased autophagy. These changes make sense in the context of reduced nutrients or nutrient sensing where the organism needs to cope with decreased amino acid substrate and energy availability.

It is noteworthy that the increased protein ubiquitination and trend toward increased carbonylation of proteins in untreated old hearts (Fig. 6D,E) are not accompanied by increased protein turnover (shorter half-lives) in old hearts [in fact, it trends toward slower turnover, consistent with a prior report (Niedermuller, 1986)]. This indicates that proteostasis is impaired in old hearts, a result that is consistent with previous reports in other tissues and organisms [reviewed in (Taylor & Dillin, 2011)].

The translational repressor 4E-BP, a downstream target of mTOR, has been shown to mediate the lifespan extension effect of dietary restriction in Drosophila by enhancement of mitochondrial activity (Zid *et al*., 2009). Using translational state array analysis, Zid *et al*. further demonstrated a preferential translation of mitochondrial genes after CR and proposed that this was related to shorter and less complex 5′UTRs. In contrast, a recent study applying transcriptome-scale ribosome profiling demonstrates that mTOR inhibition by Torin 1 in MEFs globally suppressed mRNA translation with no preferential regulation of mRNA with complex 5′UTRs (Thoreen *et al*., 2012). Our measurement of protein synthesis does not find any evidence of a preferential change in the dynamics of mitochondrial proteins in aged hearts after 10 weeks of CR or RP (Fig. 5B); however, because the relative abundance of mitochondrial proteins is increased at 10 weeks (Fig. 5A), we cannot rule out the possibility of preferential synthesis at earlier times after CR or RP treatment.

In summary, treatment with either CR or RP for 10 weeks in old mice effectively reverses the pre-existing cardiac hypertrophy and diastolic dysfunction, attenuates age-dependent protein oxidative damage and ubiquitination, significantly decreases protein turnover rate, and ameliorates proteome remodeling in aging hearts.

## Methods

Detailed methods are provided in the supplement.

### Experimental diet, isotope labeling, and echocardiography

Forty-five C57BL/6 female mice (25 months old) from the National Institute of Aging (Charles River) were handled according to the guidelines of the Institutional Animal Care Committee of the University of Washington. This single gender was used to eliminate gender differences in response, and because the National Institute of Aging Interventions Testing Program previously demonstrated a larger lifespan extension by rapamycin in females (10). One week after arrival, mice were started on a synthetic diet (Harlan Teklad diet #TD.99366), nutritionally similar to the NIH-31 standard for rodents. Three weeks later, at 26 months of age, the mice were individually housed and randomly assigned to three diet regimens for 10 weeks (Fig. 1): (i) *ad libitum* synthetic diet (CL, control group), (ii) caloric-restricted synthetic diet (CR), and (iii) *ad libitum* synthetic diet added with microencapsulated rapamycin (2.24 mg kg^−1^ day^−1^, purchased from the University of Texas Health Science Center, San Antonio). Caloric restriction mice received a diet that was 10% less than CL in week 1, 25% less in week 2, and 40% less from week 3–10. Young mice at 4 months of age received the same *ad libitum* synthetic diet as young control (YCL). After 10 weeks, treatment groups were maintained but all mice were switched to leucine-deficient synthetic diet (TD.09846, Harlan Teklad, Madison, WI, USA) supplemented with 11.1 g kg^−1^ of [5,5,5 – ^2^H_3_] – L – leucine (Cambridge Isotope Laboratory, MA, USA), the same leucine content as diet # TD. 10943. Although we measured newly synthesized proteins by heavy label incorporation, as old mouse body weights were not significantly changed during the last 7 weeks of the experiment and young weights changed only 1% per week (Fig. S1), we believe that protein synthesis and degradation are essentially in equilibrium at this time. In addition, the abundance of all peptides identified remains unchanged over the labeling period (Fig. S7, regression slopes centered over zero), indicating that proteins were at steady state at the time of labeling.

Echocardiography was performed at baseline and at the end of experiments (10 weeks) under 0.5% isoflurane, as described, using a Siemens Acuson CV-70 (Siemens Medical Solution, Mountain View, CA, USA) equipped with a 13 MHz probe(9).

### Western blots and protein carbonyl assay

Antibodies used for the Western blots were beclin-1, LC3 (both from Novus Biologicals), p62, phospho-4EBP1, 4EBP1, phospho-EEF2, phospho-S6, S6, and ubiquitin (all from cell signaling). Cardiac tissue protein carbonyl was measured using OxiSelect protein carbonyl ELISA kit (Cell Biolabs, San Diego, CA, USA).

### Sample preparation and analysis by mass spectrometry

Three to four mice of each experimental group were euthanized by cervical dislocation at each of the four time points shown in Fig. 1. The heart was removed immediately; then ventricular tissues were homogenized in cold buffer, processed and trypsin-digested, and loaded to LC-MS/MS, using a Waters nanoAcquity LC system and a Thermo Scientific LTQ-FT Ultra.

### Topograph analysis of peptides turnover and relative abundance

The Topograph software program was developed for the deconvolution and measurement of peptide isotopologue abundances from LC-MS chromatograms, and the calculation of peptide turnover rates, as previously described (18) (http://proteome.gs.washington.edu/software/topograph/). After the % newly synthesized peptide was calculated, for each of the multiple peptides that uniquely mapped to one protein (mean 50.1, median 21.5), these values were plotted for each sample at each time point to generate an exponential curve following a first order kinetics(Fig. S3B). Using a logarithmic transformation, the first order protein turnover rate (slope) is determined by linear regression. For detail, see the statistical method below, the supplementary methods, and (Hsieh *et al*., 2012).

For comparison of relative abundance between two experimental groups, we applied Topograph chromatogram alignment to correct chromatographic drift that may occur during the LC-MS/MS and allowing comparisons of low abundance analytes that may be detected in only one but not the other samples. A LOESS regression was used to find the best fit line through the data points (Fig. S8). For peptides that were identified in one sample, the regression of the identified peptide’s MS/MS scan number is used to estimate a window for the same peptide in the other samples and a matching chromatographic peak was identified within that time range.

### Metabolic profiling of cardiac tissue extract

Pulverized cardiac tissues were resuspended in 80:20 methanol:water, and soluble extracts were collected and dried by speed vac. Extracts were reconstituted in 5 mm ammonium acetate in 95% water/5% acetonitrile + 0.5% acetic acid and filtered prior to LC-MS analysis. The filtered samples were injected to the LC system which was composed of two Agilent 1260 binary pumps, an Agilent 1260 auto-sampler and an Agilent 1290 column compartment containing a column-switching valve (Agilent Technologies, Santa Clara, CA, USA). The chromatography was performed using solvents A (5 mm ammonium acetate in water + 0.5% acetic acid + 0.5% acetonitrile) and B (acetonitrile + 0.5% acetic acid + 0.5% water), with 5% B for 2 min, 5% B to 80% B in 3 min, 80% B for 3 min, 80% B to 5% B in 3 min, and 5% B for 7 min.

After the chromatographic separation, MS ionization and data acquisition was performed using AB Sciex QTrap 5500 mass spectrometer (AB Sciex, Toronto, ON, Canada) equipped with electrospray ionization (ESI) source. Multiple-reaction-monitoring (MRM) mode was used for targeted data acquisition. The extracted MRM peaks were integrated using MultiQuant 2.1 software (AB Sciex).

### Statistical analysis

Statistical analyses were performed using either Stata IC10 (StataCorp, College Station, TX, USA), R or Bioconductor (Fred Hutchinson Cancer Research Center, Seattle, WA, USA). Analyses used only peptides that mapped to a single protein. For the cases where a protein consisted of more than one peptide, statistical models were modified to appropriately account for the multiple peptides by using a blocking factor. For each protein, we applied a nonlinear regression fits of first order exponential curves to the % newly synthesized protein using: y = 100 + β1^eαt^. To determine whether the slopes α were statistically significantly different between experimental group, we used ANCOVA. Half-lives were calculated according to first order kinetics: t1/2 = ln(2)/slope. The equality of distribution of protein half-lives between two groups (Fig. 3A) was compared by a Kolmogorov–Smirnov test. The equality of distribution of protein half-life ratios (Fig. 3B) was examined using a test of proportions.

For proteomics relative abundance data, statistically significant changes of proteins between experimental groups were determined using a linear model of peptide abundance to calculate fold changes of proteins between experimental groups in the same manner as a two-sample *t*-test using the R/Bioconductor software. The linear model gave *P*-values that were adjusted for multiplicity with the Bioconductor package q-value, which allows for selecting statistically significant genes while controlling the estimated false discovery rate.

The networks and canonical pathways were generated through the use of ingenuity pathway analysis (IPA, Ingenuity Systems, www.ingenuity.com). A q < 0.05 was set to identify molecules whose expression was significantly differentially regulated. Canonical pathway analysis identified the pathways from the IPA library of canonical pathways that were significant to the data set. The significance of the association between the data set and the canonical pathway was derived by taking a ratio of the number of molecules from the data set that map to the pathway divided by the total number of molecules present in the canonical pathway. The Fisher’s exact test was used to calculate a *P*-value reflecting the probability that the association between the mapped proteins in the data set and the canonical pathway is explained by chance alone.

## Supporting Information

**Fig. S1** Body weights of mice over the course of the experiment. **Fig. S2** Topograph plot for turnover analysis. **Fig. S3** mTOR Western. **Fig. S4** Mitochondrial copy number and mitochondrial biogenesis markers did not increase with CR or RP. **Fig. S5** Precursor pool increased over time in all experimental group. **Fig. S6** Distribution of relative standard error of half-lives. **Fig. S7** Linear regression fits of peptide abundance (peptide isotopologue area) over the labeling period indicate no significant change. **Fig. S8** Topograph LOESS regression of chromatogram alignment for abundance analysis.

**Method S1** Altered proteome turnover and remodeling by subacute caloric restriction and rapamycin rejuvenate the aging heart

**Table S1** Slope of proteome dynamics (turnover) **Table S2** (A) Pathway analysis of YCL versus OCL. (B) Pathway analysis of OCR versus OCL. (C) Pathway analysis of ORP versus OCL. **Table S3** Protein abundance ratio (log2 of fold change) **Table S4** Top pathway abundance ratio (for heat map) **Table S5** Top pathway half-life ratios (for heat map) **Table S6** Metabolic profiling
