# Supplementary material for: Altered proteome turnover and remodeling by short-term caloric restriction or rapamycin rejuvenate the aging heart
Source: Aging Cell. 2014 Feb 25;13(3):529–39. doi: 10.1111/acel.12203 (PMC4040127; doi:10.1111/acel.12203)
Supplement: Supplementary file 1 — Fig. S1 Body weights of mice over the course of the experiment. Fig. S2 Topograph plot for turnover analysis. Fig. S3 mTOR Western. Fig. S4 Mitochondrial copy number and mitochondrial biogenesis markers did not increase with CR or RP. Fig. S5 Precursor pool increased over time in all experimental group. Fig. S6 Distribution of relative standard error of half-lives. Fig. S7 Linear regression fits of peptide abundance (peptide isotopologue area) over the labeling period indicate no significant change. Fig. S8 Topograph LOESS regression of chromatogram alignment for abundance analysis. [file acel0013-0529-sd1.pdf]

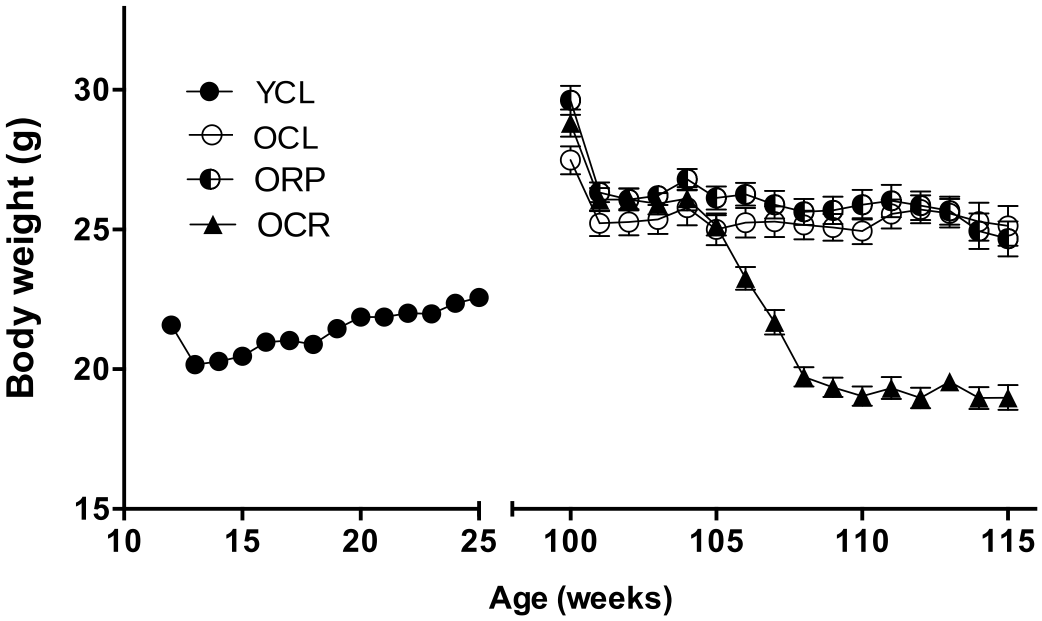

Fig. S1 Body weights of mice over the course of the experiment.

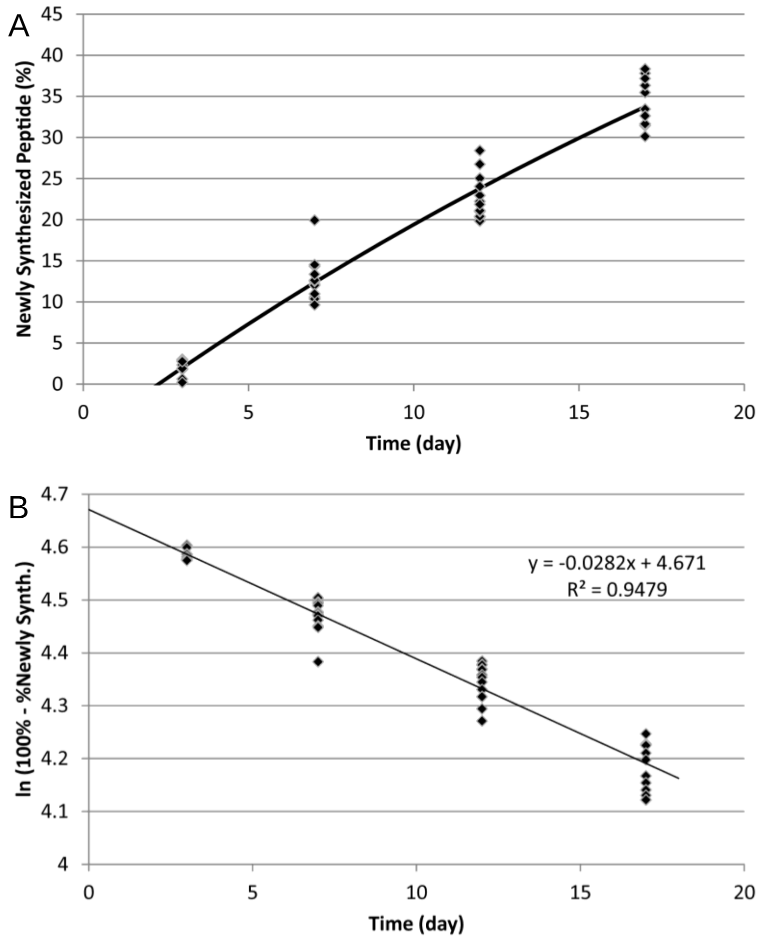

Fig. S2 Topograph plot for turnover analysis. A) The percentage of newly synthesized peptides of each mouse at each time point for each protein was used to plot a synthesis curve of the protein. An example of subunit 7 of cytochrome bc1 from the old AL hearts was plotted over time. B) The natural log of the synthesis curve of the peptides was plotted through the data points. From this, the slope of the linear regression was calculated to determine the rate constant of protein turnover, following first order kinetics.

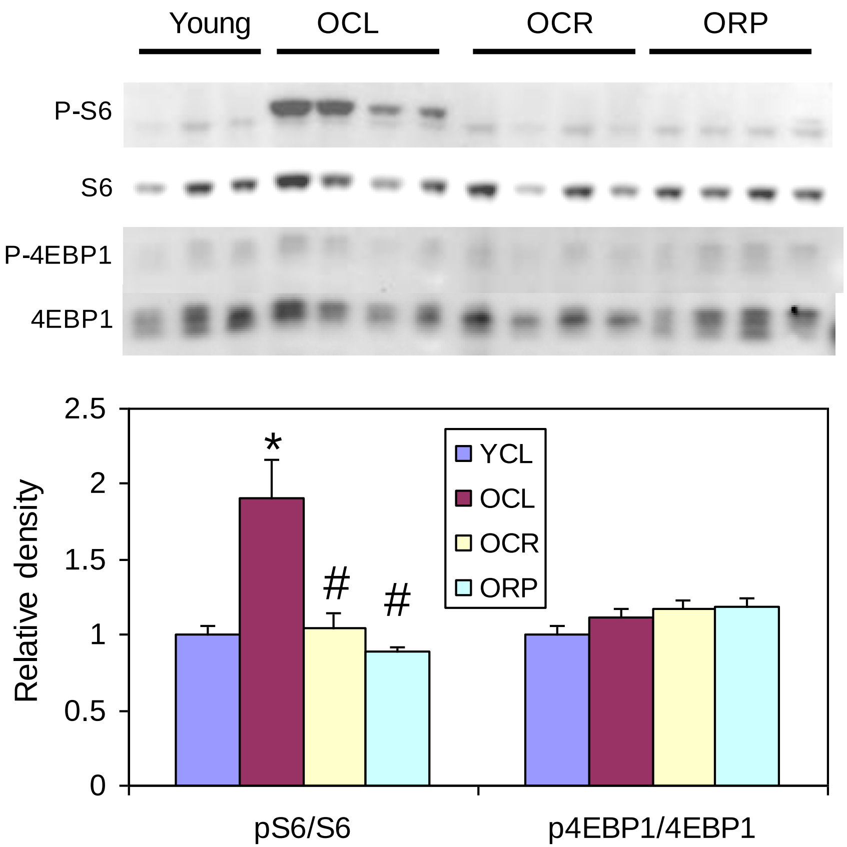

Fig. S3 mTOR Western. Phosphorylation of S6 ribosomal protein significantly increased in aged hearts, which are significantly attenuated by CR or RP. No effect of aging, CR or RP was observed for 4EBP1 phosphorylation. *p<0.05 vs. YCL; #p<0.05 vs. OCL

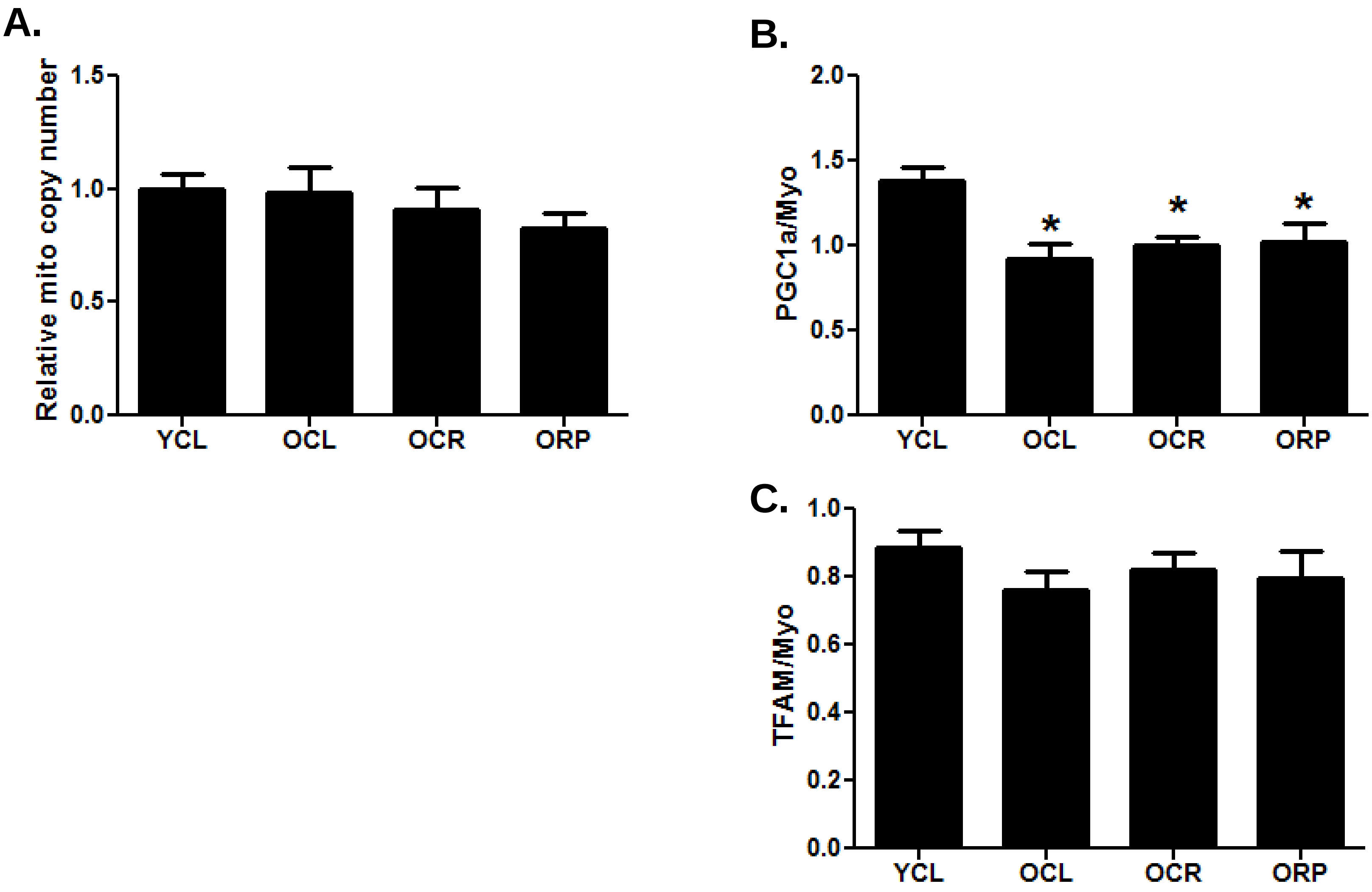

Fig. S4 Mitochondrial copy number and mitochondrial biogenesis markers did not increase with CR or RP. A)Mitochondrial copy number, measured by the ratio of mitochondrial DNA (ND1) to nuclear DNA (CYP1a1), is not altered by aging, CR and RP. B) Levels o f mitochondrial biogenesis marker, PGC-1α, reduce with aging but are not altered by CR and RP. C) Levels of mitochondrial transcriptional factor, TFAM, are not affected by aging, CR and RP. *$p<0.05$ compared to YCL.

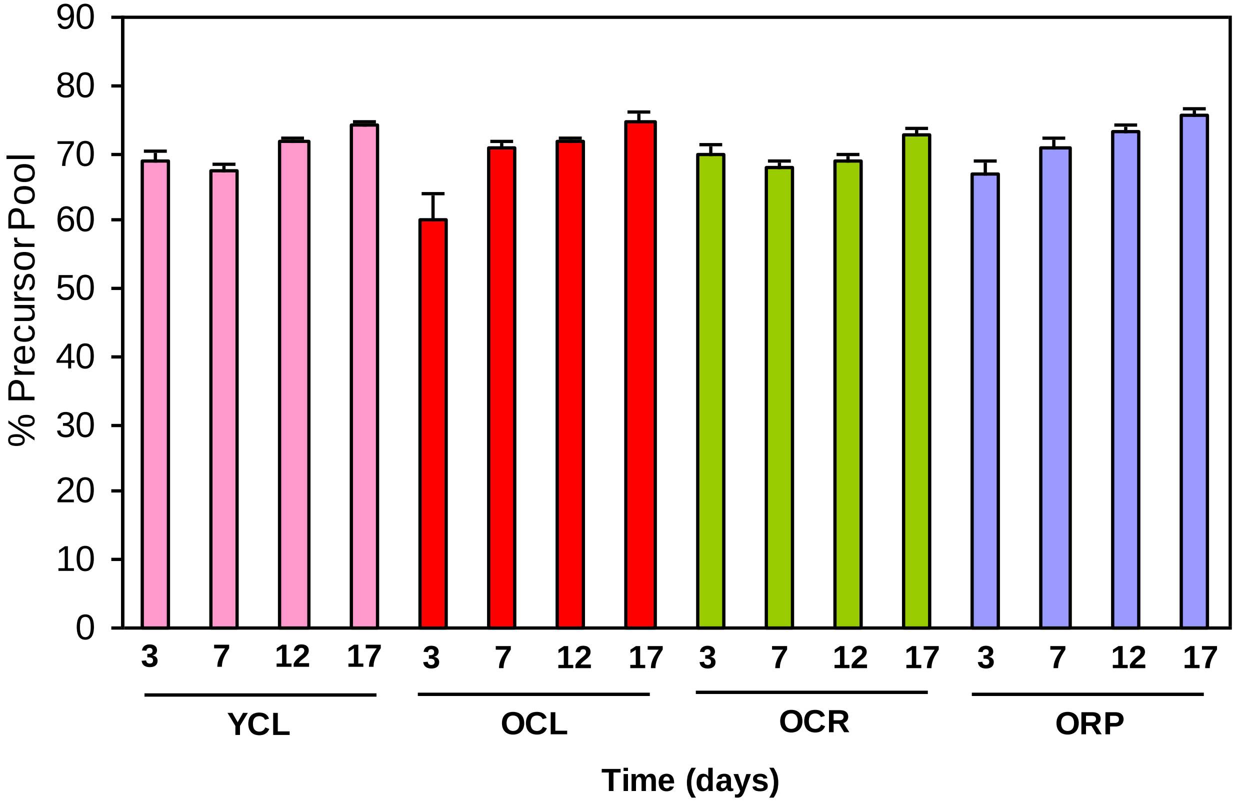

Fig. S5 Precursor pool increased over time in all experimental group.

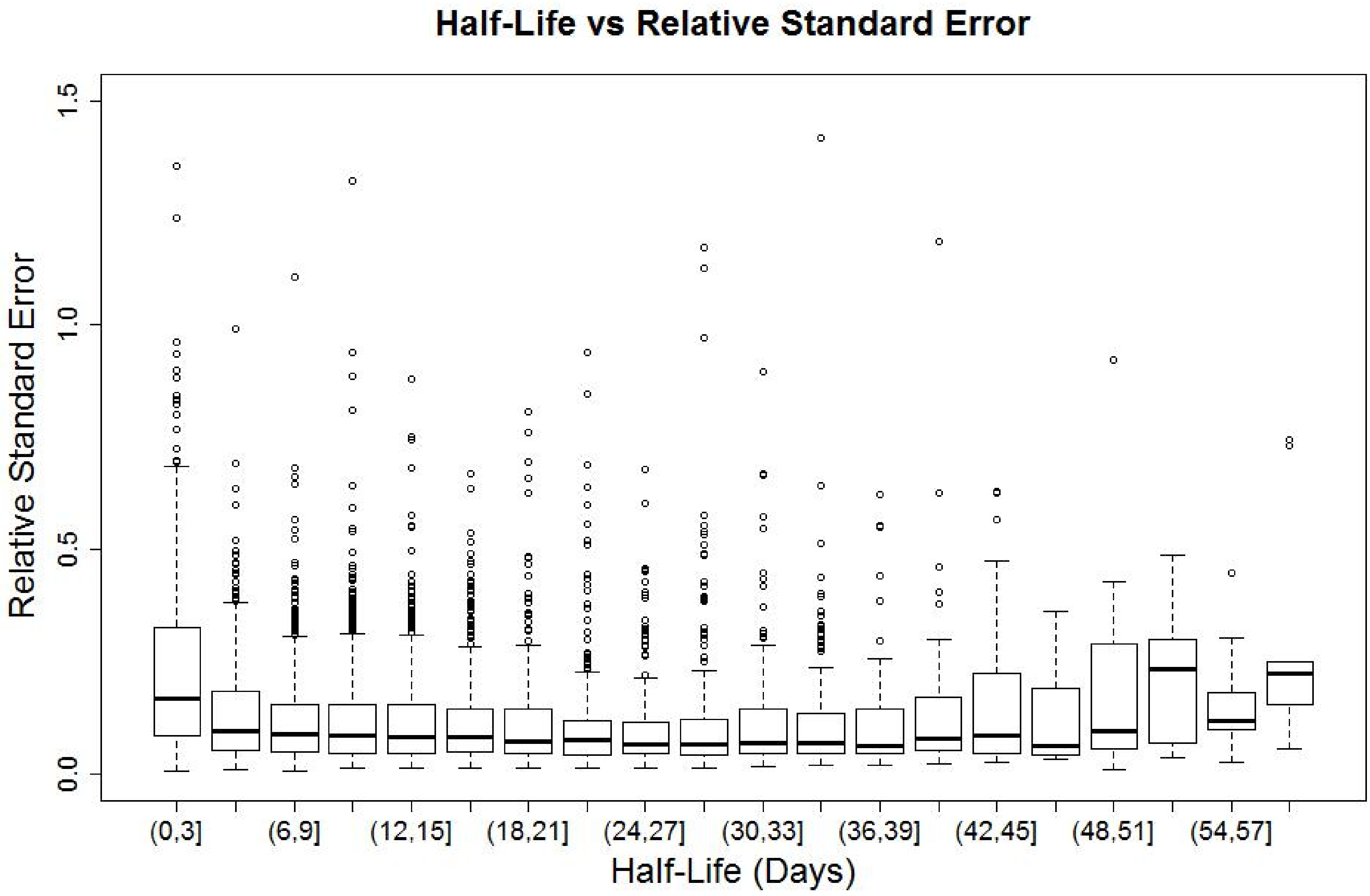

Fig. S6. Distribution of relative standard error of half lives

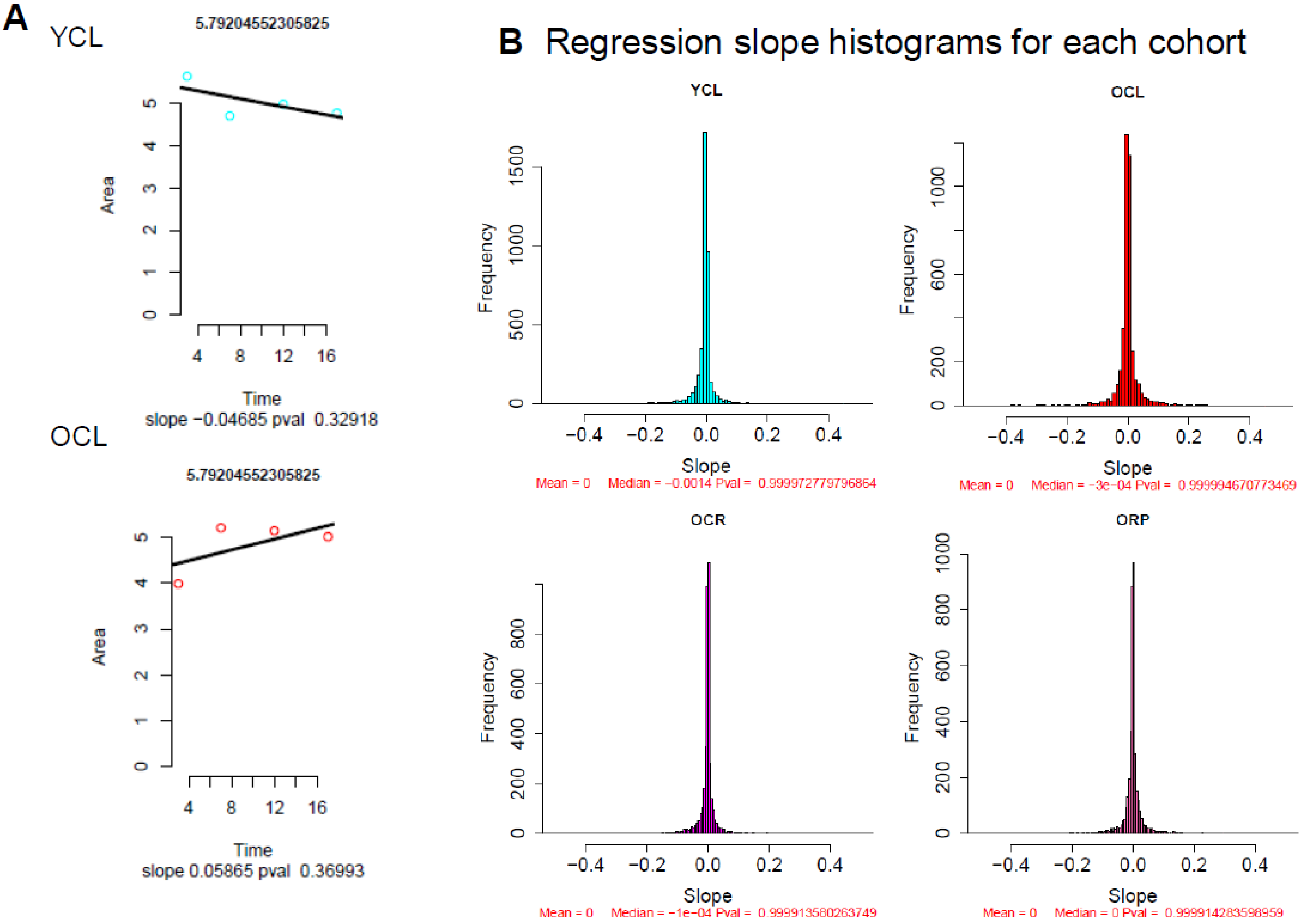

Fig. S7 Linear regression fits of peptide abundance (peptide isotopologue area) over the labeling period indicate no significant change.

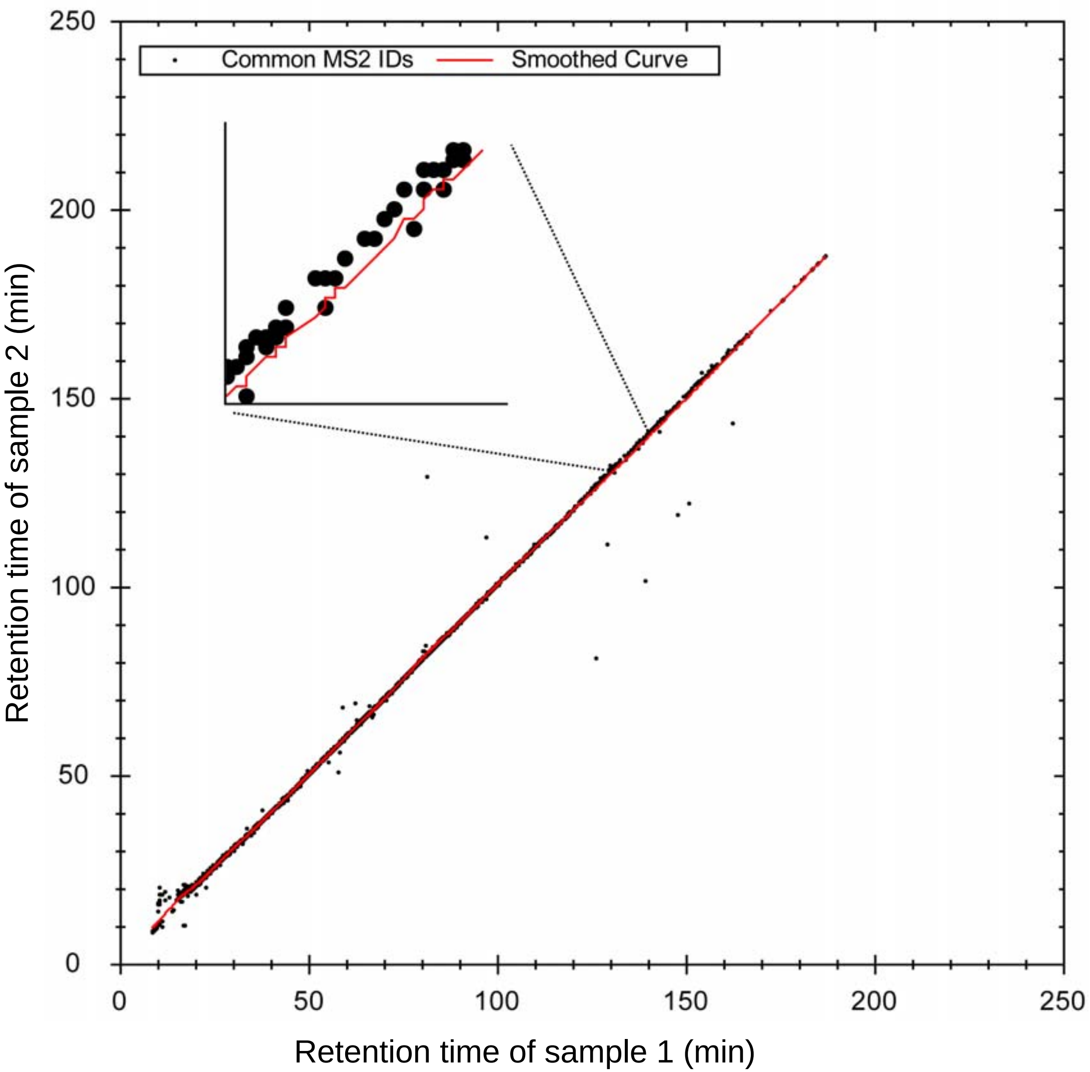

Fig S8. Topograph LOESS regression of chromatogram alignment for abundance analysis. Topograph realigned the MS/MS chromatograms of peptide ions from different samples. For two given MS/MS chromatograms, the MS/MS scan numbers for peptides identified in both samples were plotted against each other in a scatter plot. A LOESS regression was used to find the best fit line through the data points. For peptides that were identified in one sample, we used the identified peptide's MS/MS scan number and the LOESS regression to identify the corresponding retention time in the other samples.
