# Supplementary material for: Altered proteome turnover and remodeling by short-term caloric restriction or rapamycin rejuvenate the aging heart
Source: Aging Cell. 2014 Feb 25;13(3):529–39. doi: 10.1111/acel.12203 (PMC4040127; doi:10.1111/acel.12203)
Supplement: Supplementary file 2 — Method S1 Altered proteome turnover and remodeling by subacute caloric restriction and rapamycin rejuvenate the aging heart [file acel0013-0529-sd2.doc]

**Altered proteome turnover and remodeling by subacute caloric restriction and rapamycin rejuvenate the aging heart**

Dai, et al

**Supplementary Methods**

**Animals**

C57BL/6 female mice were purchased from the National Institute of Aging Charles River colony at 3 and 25 mo age. Upon arrival mice were housed in a barrier specific-pathogen-free facility maintained at 70-74 °F, 45-55% humidity, with 28 air changes/hour and 12/12-h light/dark cycle, as described(D*ai et a*l. 2009). All animals were handled according to the guidelines of the Institutional Animal Care Committee of the University of Washington and the National Institute of Health. One week after arrival, mice were started on a synthetic diet (Harlan Teklad diet #TD.99366) that was nutritionally similar to the NIH-31 standard for rodents. The use of this diet facilitated the subsequent introduction of heavy-labeled [5,5,5 - ^2^H_3_] Leucine that enabled protein turnover and abundance measurements. Mouse weights and food intake were recorded weekly. Three weeks later, at 4 and 26 months of age, the mice were individually housed and randomly assigned to three groups (Fig.1). One group was maintained on an *ad libitum* synthetic diet regimen (control) while the other two groups were started on RP-containing diet or caloric restriction (CR, see below). All mice were maintained on these three diet regimens for 10 weeks. There was an average of 7.4% body weight loss on the synthetic diet that stabilized after one week (Fig. S1). There was no loss in body weight in the *ad libitum* and RP groups after the initial weight loss.

**Diet Regimens and Feeding**

Mice in the CR group received a vitamin and mineral adjusted diet (Harlan diet # TD. 10943) designed to provide the same amount of essential nutrients that the control group received. CR diet per mouse were calculated based on the age-matched *ad libitum* mouse intake normalized to body weight then multiplying by each restricted animal's initial body weight (prior to start of restriction). Caloric restricted mice received a diet that was 10% less than *ad libitum* consumption in week 1, 25% less in week 2, and 40% less thereafter. Rapamycin-treated mice received 14mg RP per kg of food (2.24mg of RP per kg body weight per day), The microencapsulated RP was purchased from the University of Texas Health Science Center, San Antonio and was of the same manufacture and dosage as that of the NIA interventional testing program (Harris*on et a*l. 2009). The plasma RP concentration for RP treated mice was 43.8±3.8 ng/mL at 5 weeks after RP feeding.

**Mouse stable isotope labeling**

After 10 weeks of CR and RP, all mice (young control, old AL, old CR, old RP) were switched to leucine-deficient synthetic diet (TD.09846, Harlan Teklad, Madison,WI) supplemented with 11.1 g/kg of [5,5,5 - ^2^H_3_] - L - leucine (Cambridge Isotope Laboratory, MA), the same leucine content as diet # TD. 10943. There was no significant change in mouse body weight (Fig. S1) and food consumption over the course of the 17 day heavy leucine labeling. Three mice of each experimental group were euthanized for tissue collections and proteomics analysis at each of the following time points: days 3, 7, 12 and 17 after switching to ^2^H_3_ - leucine diet.

**Echocardiography**

Echocardiography was performed at baseline and at the end of experiments (10 weeks) as described using Siemens Acuson CV-70 equipped with 13MHz probe(9). Briefly, isoflurane 0.5% mixed with O2 was used to provide adequate sedation but minimal cardiac suppression during echocardiography. M-mode, conventional and Tissue Doppler, and functional calculations were performed according to American Society of Echocardiography guidelines. An increase in MPI (calculated as the ratio of the sum of isovolemic contraction and relaxation time to LV ejection time) indicates that a greater fraction of systole is spent to cope with the pressure changes during isovolemic phases, and has been shown to reflect both LV systolic and diastolic dysfunction (30).

**Mitochondrial DNA copy number determination**

Total DNA were extracted from cardiac tissue with PureLink^TM^ Genomic DNA extraction kit (Life Technology), according to manufacturer’s protocol. Quantitative real-time PCR of mitochondrial gene NADH dehydrogenase 1 (ND1) and a single-copy nuclear gene cytochrome P4501A1 (cyp1A1) were performed in a Rotor Gene 3000. Mitochondrial DNA copy numbers were estimated by the ratio of the amount ND1 and cyp1A1. The following primers were used for ND1 (forward: GAACGCAAAATCTTAGGGTACATACA, reverse: GCCGTATGGACCAACAATGTT) and for cyp1A1 (forward: GACACAGTGATTGGCAGAGATC, reverse: AACGGATCTATGGTCTGACCTGT)(D*ai et a*l. 2009).

**Gene expression analysis of mitochondrial biogenesis markers**

RNA was extracted from cardiac tissue with Qiagen RNeasy kit and cDNA was generated. Gene expression analysis of mitochondrial biogenesis markers, PGC1-α (Mm00731216) and TFAM (Mm00447485), were performed with Taqman Gene Expression Assays in Rotor Gene 3000. Gene expression of myoglobin (Mm00442968) was used for normalization.

**Western blots and protein carbonyl assay**

Antibodies used for the Western blots were Beclin-1, LC3 (both from Novus Biologicals), p62, phospho-4EBP1, 4EBP1, phospho-EEF2, phospho-S6 and S6 (all from Cell Signaling), EEF2 (Santa Cruz), and Donkey anti-rabbit secondary antibody (Thermo Scientific). Cardiac tissue protein carbonyl was measured using OxiSelect protein carbonyl ELISA kit, according to manufacturer`s protocol (Cell Biolabs, San Diego, CA).

**Sample Preparation and Analysis by Mass Spectrometry**

Four mice from each experimental group at each time point were processed for proteomics. Mice were euthanized by cervical dislocation. The heart was removed immediately and rinsed in cold saline, then ventricular tissues were homogenized in cold isolation buffer (250 mM sucrose, 1 mM EGTA, 10 mM HEPES, 10 mM Tris-HCl pH7.4). The lysates were centrifuged at 800 x g for 10 minutes to get rid of the debris. For mass spectrometery, the total lysates were suspended with Rapigest (Waters Corporation, Milford, MA) to a final concentration of 0.1% and boiled for 5 min. The samples were treated with 5 mM DTT at 60 °C for 30 minutes to reduce the disulfide bonds. The free sulfhydryls were then alkylated with treatment of 15 mM iodoacetamide at room temperature for 30 minutes. Trypsin was added to a final concentration of 1:100 (trypsin/ protein) and the sample was digested at 37 °C for 2 hours. The trypsin and Rapigest were hydrolyzed by 200 mM HCl, at 37 °C for 30 minutes. The samples were then centrifuged for 10 minutes at 20,000 g and the supernatant was washed using a Waters Oasis MCX sample extraction column, according to the manufacturer`s protocol (Waters Corporation, Milford,MA). The digested samples were then loaded to the ultra-performance liquid chromatography and mass spectrometry (UPLC-MS/MS), using a Waters nanoAcquity LC system and a Thermo Scientific LTQ-FT Ultra. The LC mobile phase consisted of buffer A (water, 0.1% formic acid) and buffer B (acetonitrile, 0.1% formic acid).

**LC-MS/MS and peptide database search.**

LC-MS/MS analysis of the tryptic digested samples was performed with a Waters nanoAcquity UPLC and a Thermo Scientific LTQ-FT Ultra. Briefly, 1 – 2 μg of digested protein sample was loaded onto a 4 cm long trapping column (100 μm inner diameter) then separated over a 35 cm long (75 μm inner diameter) homemade fused silica capillary column, packed with Jupiter Proteo C-12 resin (Phenomenex), at a flow rate of 250 nl/min. The mobile phase gradient used consisted of buffer A (100% water, 0.1% formic acid) and buffer B (100% acetonitrile, 0.1% formic acid). A linear gradient (2% to 32% buffer B in buffer A) was done from 0 to 180 minutes, followed by 5 minutes at 80% buffer B and a re-equilibration step of 15 minutes at 2% buffer B. The LTQ-FT Ultra was operated with a scan cycle of one MS scan in the ICR cell (50,000 resolving power FWHM at 400 m/z) followed by five data-dependent MS/MS scans in the ion trap (2,000 target ions, dynamic exclusion was set to 30 seconds with a repeat count of 1).

High resolution MS data was processed using Bullseye algorithm (Hsi*eh et a*l. 2010) to assign the accurate precursor ion mass to the low resolution tandem mass spectrometry data. MS/MS spectra were searched by SEQUEST (ver.27) against a mouse IPI database (v3.57). The search was done with fully-tryptic specificity, a static mass modification of 57.021 on cysteines and a precursor mass tolerance of ±10 ppm. A dynamic modification of 3.0188325 for leucines was set to account for [5,5,5 - 2H3] - leucines. The precursor monoisotopic mass tolerance was set to ± 10 ppm. Peptide spectrum match false discovery rates were determined by the Percolator algorithm(Ka*ll et a*l. 2007) with a q-value threshold of 0.01. The raw data is available at https://sites.google.com/a/uw.edu/maccoss/home/supplementary_data/

**Topograph analysis of peptides turnover**

The Topograph software program was developed for the deconvolution and measurement of peptide isotopologue abundances from LC-MS chromatograms, and the calculation of peptide turnover rates, as previously described (Hsi*eh et a*l. 2012) (http://proteome.gs.washington.edu/software/topograph/). Briefly, the isotope distribution for each possible labeled form of the peptide was calculated using the method described by Kubinyi(Kubinyi 1991). A list of m/z windows was generated for all isotope peaks including all charge states. Using these m/z windows, the fraction of each isotopologue in an MS spectrum was determined by calculating the fractional amount of each theoretical isotopologue distribution that best fits the observed spectral signal by a least squares analysis(Brauman 1966). The total abundance of each isotopologue was determined by integrating over time, across its chromatographic peak. The newly synthesized peptides used amino acid from the cellular amino acid pool, which consisted of a mixture of unlabeled and labeled isotopologs. Topograph calculates this mixture, named precursor pool, for each time point, as shown in Fig. S5 and applies this value to accurately calculate the percent newly synthesized peptide. A deconvolution score was calculated for each scan by a dot product of the observed isotope distribution and the predicted isotope distribution, and normalizing to the observed isotope distribution, and was intensity weighted and summed across the chromatographic peak. Peptides with a deconvolution score of less than 0.98 were excluded from the turnover analysis.

After the percent newly synthesized peptide was calculated, it was plotted for each sample at each time point to generate an exponential curve following a first order kinetics (Fig. S2A), of which the natural logarithmic transformation could be determined by a linear regression (Fig. S2B). For detail of the method for peptide turnover calculation, see Hsieh et al(Hsi*eh et a*l. 2012).

**Topograph analysis of relative abundance**

For comparison of relative abundance between two experimental groups, we applied Topograph chromatogram alignment in order to reduce the effect of chromatographic drift that may occur during the LC-MS/MS and allowing comparisons of low abundance analytes that may be detected in only one but not the other samples. This chromatogram alignment enables an accurate comparison of peptide peak areas under the curves between samples. Briefly, for two given LC-MS/MS chromatograms, the MS/MS scan number for peptides identified in both samples were plotted against each other in a scatter plot. A LOESS regression was used to find the best fit line through the data points (Fig. S8). For peptides that were identified in one sample, Topograph applied the LOESS regression and the identified peptide's MS/MS scan number to estimate an approximate time range of the same peptide in the other samples. A matching chromatographic peak was then identified within that time range.

**Metabolic profiling of cardiac tissue extract**

Pulverized cardiac tissues were resuspended in 80:20 methanol:water and incubated on dry ice for 30 min. Supernatants containing soluble extracts were stored. The tissues were resuspended with 80:20 methanol:water and incubated on ice for a second extraction. A third extraction was performed by sonicating the tissue suspension in ice-water sonication bath. The 3 extractions were pooled together and dried by speed vac.

Dried samples were reconstituted in 5 mM ammonium acetate in 95% water/5% acetonitrile + 0.5% acetic acid and filtered prior to LC-MS analysis. The filtered samples were injected to the LC system which was composed of two Agilent 1260 binary pumps, an Agilent 1260 auto-sampler and Agilent 1290 column compartment containing a column-switching valve (Agilent Technologies, Santa Clara, CA). The chromatography was performed using Solvents A (5 mM ammonium acetate in H_2_O + 0.5% acetic acid + 0.5% acetonitrile) and B (acetonitrile + 0.5% acetic acid + 0.5% water), with 5% B for 2 min, 5% B to 80% B in 3 min, 80% B for 3 min, 80% B to 5% B in 3 min, and 5% B for 7 min.

After the chromatographic separation, MS ionization and data acquisition was performed using an AB Sciex QTrap 5500 mass spectrometer (AB Sciex, Toronto, ON, Canada) equipped with electrospray ionization (ESI) source. Multiple-reaction-monitoring (MRM) mode was used for targeted data acquisition. The extracted MRM peaks were integrated using MultiQuant 2.1 software (AB Sciex, Toronto, ON, Canada). Reproducibility was excellent with average CV values for metabolites measured in quality control samples of 4% and 5% for positive and negative ion mode detection, respectively.

**Statistical analysis**

Continuous data were presented as means ± SEM. Paired t-tests were applied to evaluate baseline and post-treatment continuous data (echocardiography). One-way ANOVA was used to compare differences among multiple experimental groups, followed by post-hoc tests for significance. P<0.05 was considered significant. All statistical analysis was performed using Stata IC10, R or Bioconductor package.

For proteomics turnover data, we used a multi-step approach to determine the difference in proteins between experimental groups. First, we excluded data from

non-unique peptides by comparing the peptide sequences to the protein sequences obtained from Uniprot (http://www.uniprot.org). Our analysis only focuses on unique peptides that map to a single protein. For the cases where a protein consisted of more than one peptide, statistical models were modified to appropriately account for the multiple peptides by using a blocking factor. For each protein we applied non-linear regression fits of first order exponential curves to the % newly synthesized protein using: y = 100 + β1^eαt^. To determine whether the slopes α were statistically significantly different between experimental groups, we used ANCOVA. Half lives were calculated according to first order kinetics: t1/2 = ln(2)/ slope.

Second, we performed non-linear regression fits of first order exponential curves to the percent newly synthesized for each protein using the following equation: y = 100 + β_1_e^αt^, where the two parameters to be determined were the intercept, β_1_, and the slope, α. To reduce non-specific signal, we used several filters: slope < 0 (deuterated leucine increase over time), x-intercepts, t(y=0)> 0 (deuterated leucine incorporated after starting the labeled diet ), and deconvolution score (derived from Topograph) >0.98. Using the optimum filters allowed us to find a common x-intercept for each experimental group, which is the median x-intercept of all peptides: 1.56 for YCL, 1.61 for OCL, 1.93 for OCR and 1.49 for ORP, all in days. The second part of this second step used the best choice for β1 for each experimental group, and refit a first order exponential: y = 100(1- e ^α(t-t^_0_)), to determine the slope, α, where t_0_, the time when y=0, is determined from β_1_ from the previous equation.

Third, we determined which slopes were statistically significantly different between experimental groups by a standard ANCOVA analysis to determine if the protein slopes were different between groups. Half lives were calculated according to first order kinetics: t_1/2_ = ln(2)/ slope.

For proteomics relative abundance data, in order to determine statistically significant changes of proteins between experimental groups, we used a linear model of peptide abundance to calculate fold changes of proteins between experimental groups in the same manner as a two-sample t-test using the Bioconductor software (Gentlem*an et a*l. 2004). For the cases where a protein consisted of more than one peptide, the linear model was modified to appropriately account for the multiple peptides by using a blocking factor. The linear model gave p-values that were adjusted for multiplicity with the Bioconductor package q-value, which allows for selecting statistically significant genes while controlling the estimated false discovery rate.

The networks and canonical pathways were generated through the use of Ingenuity Pathway Analysis (IPA, Ingenuity Systems, www.ingenuity.com). A data set containing gene identifiers and corresponding expression values (natural log of fold change) and p-values between two-group comparisons were used. Each gene was mapped to its corresponding object in the Ingenuity® Knowledge Base. A q<0.05 was set to identify molecules whose expression was significantly differentially regulated. These molecules, called Network Eligible molecules, were overlaid onto a global molecular network developed from information contained in the Ingenuity Knowledge Base. Networks of Eligible Molecules were then algorithmically generated based on their connectivity. Canonical pathway analysis identified the pathways from the IPA library of canonical pathways that were significant to the data set. The significance of the association between the data set and the canonical pathway is derived by: 1) A ratio of the number of molecules from the data set that map to the pathway divided by the total number of molecules that map to the canonical pathway is displayed. 2) Fisher's exact test was used to calculate a p-value reflecting the probability that the association between the genes in the dataset and the canonical pathway is explained by chance alone. The mitochondrial dysfunction pathway consists of proteins of the electron transport chain and proteins whose defects are associated with mt dysfunction related diseases.

The equality of distribution of protein half lives between two groups (Fig. 3A) was compared by a Kolmogorov-Smirnov test. The equality of distribution of protein half-life ratio was examined using a test of proportions (Fig.3B).

**REFERENCES**

Brauman JI (1966). Least Squares Analysis and Simplification of Multi-Isotope Mass Spectra. *Anal. Chem.* **38**, 607-610.

Dai DF, Santana LF, Vermulst M, Tomazela DM, Emond MJ, MacCoss MJ, Gollahon K, Martin GM, Loeb LA, Ladiges WC , Rabinovitch PS (2009). Overexpression of catalase targeted to mitochondria attenuates murine cardiac aging. *Circulation*. **119**, 2789-2797.

Gentleman RC, Carey VJ, Bates DM, Bolstad B, Dettling M, Dudoit S, Ellis B, Gautier L, Ge Y, Gentry J, Hornik K, Hothorn T, Huber W, Iacus S, Irizarry R, Leisch F, Li C, Maechler M, Rossini AJ, Sawitzki G, Smith C, Smyth G, Tierney L, Yang JY , Zhang J (2004). Bioconductor: open software development for computational biology and bioinformatics. *Genome Biol*. **5**, R80.

Harrison DE, Strong R, Sharp ZD, Nelson JF, Astle CM, Flurkey K, Nadon NL, Wilkinson JE, Frenkel K, Carter CS, Pahor M, Javors MA, Fernandez E , Miller RA (2009). Rapamycin fed late in life extends lifespan in genetically heterogeneous mice. *Nature*. **460**, 392-395.

Hsieh EJ, Hoopmann MR, MacLean B , MacCoss MJ (2010). Comparison of database search strategies for high precursor mass accuracy MS/MS data. *J Proteome Res*. **9**, 1138-1143.

Hsieh EJ, Shulman NJ, Dai DF, Vincow ES, Karunadharma PP, Pallanck L, Rabinovitch PS , MacCoss MJ (2012). Topograph, a software platform for precursor enrichment corrected global protein turnover measurements. *Mol Cell Proteomics*. **11**, 1468-1474.

Kall L, Canterbury JD, Weston J, Noble WS , MacCoss MJ (2007). Semi-supervised learning for peptide identification from shotgun proteomics datasets. *Nat Methods*. **4**, 923-925.

Kubinyi H (1991). Calculation of isotope distributions in mass spectrometry. A trivial solution for a non-trivial problem. . *Anal. Chim. Acta*. **247**, 107-119.
