# Supplementary material for: Altered proteome turnover and remodeling by short-term caloric restriction or rapamycin rejuvenate the aging heart
Source: Aging Cell. 2014 Feb 25;13(3):529–39. doi: 10.1111/acel.12203 (PMC4040127; doi:10.1111/acel.12203)
Supplement: Supplementary file 3 — Table S1 Slope of proteome dynamics (turnover) Table S2 (A) Pathway analysis of YCL versus OCL. (B) Pathway analysis of OCR versus OCL. (C) Pathway analysis of ORP versus OCL. Table S3 Protein abundance ratio (log2 of fold change) Table S4 Top pathway abundance ratio (for heat map) Table S5 Top pathway half-life ratios (for heat map) Table S6 Metabolic profiling [file acel0013-0529-sd3.pdf]

STAB 1. Slope of proteome dynamics (turnover)

UniProtKB	Entrez	YCL			OCL			OCR			ORP					
		slope	SE	r2	slope	SE	r2	slope	SE	r2	slope	SE	r2			
P06151	16828	0.0899602	0.001205	0.9672	0.0916038	0.001029	0.9786	0.07163292	0.001181	0.95214	0.0759035	0.0012178	0.95831			
P21550	13808	0.0614529	0.000731	0.9764	0.0572131	0.00099	0.9584	0.04393463	0.000789	0.94691	0.04425343	0.0006956	0.96449			
P70349	15254	0.063581	0.00071	0.9929	0.0628949	0.001023	0.9867	0.04562609	0.000742	0.98464	0.05440517	0.0008047	0.98918			
P09411	18655	0.0594831	0.000993	0.9455	0.057974	0.000865	0.9611	0.0421826	0.000655	0.95274	0.04660544	0.0007683	0.95337			
Q9CZ13	22273	0.0258964	0.000332	0.9554	0.0254441	0.000733	0.8306	0.01984145	0.000423	0.88612	0.01962004	0.0003858	0.91345			
Q9CY10	NA	0.0246749	0.000903	0.8431	0.0402833	0.003582	0.5393	0.03300492	0.004025	0.34799	0.03115585	0.0014783	0.80005			
OTTMUSP0(	NA	0.0778271	0.000927	0.998	0.0774887	0.001042	0.9978	0.05577453	0.001189	0.99367	0.06241613	0.0013794	0.99417			
P06745	14751	0.0711144	0.001473	0.9148	0.070564	0.001631	0.9047	0.05266589	0.000887	0.94131	0.05626041	0.0012147	0.91628			
P48771	12866	0.1008993	0.001014	0.9971	0.0935149	0.002017	0.9885	0.05054787	0.001593	0.972	0.07964961	0.001625	0.9897			
Q9Z2I9	20916	0.033972	0.000579	0.9512	0.0318657	0.000868	0.8968	0.02370207	0.000798	0.83199	0.02602608	0.0006997	0.90103			
B2RXT3	NA	0.0511412	0.001912	0.7648	0.0669654	0.006997	0.3196	0.04125112	0.000985	0.8788	0.0441341	0.0062284	0.1983			
P02088	NA	0.0254613	0.000928	0.848	0.0383366	0.001755	0.8085	0.03273009	0.003891	0.36144	0.03172374	0.0016248	0.78728			
P59511	223838	0.0543772	0.000674	0.9983	0.0532792	0.001586	0.9912	0.03925635	0.001486	0.98171	0.04207558	0.0013356	0.99002			
Q8BMS1	97212	0.0304608	0.000564	0.8585	0.0288339	0.00322	0.1632	0.02287651	0.003824	0.06939	0.03070756	0.0041171	0.11647			
P99028	66576	0.0303502	0.000697	0.9629	0.0322551	0.000781	0.9638	0.02344092	0.00064	0.9477	0.02429055	0.0007578	0.94136			
P70695	14120	0.0911748	0.002298	0.9937	0.0905796	0.001676	0.9966	0.06678674	0.001587	0.99271	0.07614474	0.003639	0.97332			
Q5FW75	NA	0.0632844	0.002233	0.9503	0.061252	0.002543	0.9447	0.04581526	0.002211	0.91282	0.04724402	0.002331	0.91942			
A2CEK3	NA	0.0675243	0.001015	0.9968	0.0685558	0.00206	0.9893	0.05006064	0.001155	0.9926	0.05518053	0.0013939	0.9924			
XP_0014779	NA	0.0691148	0.000758	0.9983	0.0669715	0.002219	0.9902	0.05092069	0.001428	0.98988	0.19150384	0.1078155	0.22289			
Q9R0X4	56360	0.0892851	0.013333	0.882	0.0311502	0.009156	0.5626	0.04972911	0.033992	0.16288	0.03617106	0.0096506	0.58416			
Q8BMF3	109264	0.0293937	0.001157	0.8555	0.0279169	0.014128	0.0383	0.02167348	0.001373	0.71144	0.02260105	0.0013328	0.74972			
Q9QYR9	171210	0.0293121	0.001239	0.7757	0.0421398	0.009187	0.1298	0.02236026	0.00396	0.1653	0.02323974	0.0012083	0.72402			
Q9CQA3	67680	0.0558082	0.001772	0.8551	0.0524045	0.009266	0.186	0.04008034	0.00208	0.68715	0.042095	0.0015638	0.83326			
Q8BKZ9	27402	0.0292629	0.000818	0.9668	0.0277721	0.000963	0.9563	0.02245493	0.000772	0.95055	0.02053308	0.0007781	0.94825			
Q91VR2	11949	0.0262173	0.000607	0.9414	0.0236997	0.000898	0.8766	0.01971715	0.000552	0.914	0.01975	0.0006642	0.89475			
P26443	14661	0.0330908	0.001227	0.9099	0.0324483	0.001773	0.8395	0.02842732	0.00176	0.77896	0.02580602	0.0013573	0.85158			
Q9D1H9	76293															
Q8JZL0	68910	0.0000534	7.99E-06	0.7613	0.0005654	0.000261	0.2815	0.0000622	1.49E-05	0.55385	0.00014202	5.76E-05	0.33641			
Q9CQZ5	67130	0.0410181	0.000904	0.9861	0.0390966	0.000753	0.9908	0.02906588	0.001006	0.96644	0.03180395	0.0011156	0.97016			
P53395	13171	0.0334213	0.001362	0.8918	0.0297926	0.001741	0.8229	0.02229206	0.00112	0.84438	0.02451978	0.0011431	0.87789			
P14869	11837	0.121388	0.003383	0.9892	0.117732	0.005131	0.9777	0.07795804	0.002456	0.9863	0.07111786	0.0028826	0.98067			
Q9D0K2	67041	0.0611367	0.001576	0.898	0.0562422	0.008682	0.2152	0.03580788	0.00775	0.10656	0.04915008	0.0025492	0.70574			
P05202	14719	0.0248909	0.000766	0.7584	0.0232492	0.005023	0.0688	0.0236196	0.003516	0.11749	0.02577925	0.0045276	0.09993			
P14602	15507	0.1977375	0.007465	0.9152	0.180745	0.003574	0.9771	0.12504633	0.003021	0.96452	0.14112643	0.0024762	0.98275			
Q9R062	27357	0.1327257	0.004636	0.967	0.1843032	0.047279	0.378	0.08281504	0.003568	0.95227	0.17029811	0.0511717	0.30701			
Q99K24	106947	0.0298644	0.003108	0.8684	0.0305167	0.004076	0.8237	0.02708877	0.002971	0.85586	0.02910686	0.0029662	0.88919			
Q9CQ62	67460	0.0281953	0.001282	0.8188	0.0237402	0.001631	0.6971	0.01810762	0.001002	0.75859	0.02275767	0.0021322	0.55054			
P23927	12955	0.3024672	0.022892	0.6312	0.2219416	0.013826	0.7411	0.1357912	0.003351	0.94098	0.15197106	0.0026653	0.97306			
Q6P9Q7	NA	0.0362909	0.001466	0.9548	0.0353769	0.001538	0.9549	0.02578376	0.001861	0.86872	0.02972082	0.0018063	0.91546			

O35658	NA	0.0409286	0.002606	0.8916	0.034675	0.002658	0.8545	0.03015236	0.002247	0.8491	0.03116336	0.0022908	0.87681
Q3U132	67596	0.0259215	0.000524	0.9951	0.0230999	0.000656	0.9904	0.01977343	0.00048	0.99183	0.01955592	0.0006989	0.98491
Q8K1M6	74006	0.101374	0.004389	0.9727	0.0791122	0.009058	0.8266	0.08507105	0.014394	0.74431	0.07517214	0.0058964	0.93125
Q9Z2U1	26442	0.124775	0.003503	0.9891	0.1385057	0.006083	0.9774	0.09230307	0.002609	0.98894	0.09517809	0.0053025	0.96409
Q1XH17	434246	0.1165341	0.003153	0.8841	0.1047296	0.003597	0.8363	0.10407968	0.012823	0.26472	0.09379438	0.0031703	0.83817
B1ATE2	NA	0.0055263	0.002914	0.2646	0.004366	0.003907	0.111	0.00862599	0.003929	0.28656	0.00454878	0.0024106	0.26258
P62806	1E+08	0.0085922	0.000965	0.5732	0.0104304	0.00097	0.6983	0.00945047	0.00175	0.34232	0.01209477	0.0032149	0.21724
Q9WUR2	23986	0.0353598	0.001577	0.8554	0.0331977	0.00275	0.6663	0.02504687	0.001171	0.8386	0.02809304	0.0018635	0.75188
Q9CQ75	17991	0.0341887	0.000696	0.9881	0.0342728	0.009975	0.3208	0.02522554	0.000897	0.96466	0.02634082	0.001077	0.95989
Q8VDQ1	77219	0.1618814	0.004571	0.989	0.1432221	0.012368	0.9179	0.09428263	0.001179	0.99781	0.1461169	0.0112467	0.93363
P16045	16852	0.0942467	0.001769	0.9899	0.0908422	0.004582	0.9402	0.06658969	0.001944	0.9775	0.08002697	0.0018189	0.98725
P47738	11669	0.1100765	0.001325	0.9801	0.094284	0.00179	0.9582	0.07140332	0.001039	0.97119	0.09281884	0.0012422	0.97845
Q3ULL9	NA	0.0412256	0.00319	0.8883	0.035448	0.002797	0.8942	0.02306429	0.002417	0.80542	0.02863379	0.0025842	0.89112
Q80Y62	NA	0.0247087	0.001431	0.9613	0.023544	0.001634	0.9497	0.02127809	0.000999	0.97005	0.01953225	0.001201	0.96007
Q8CI94	110078	0.1470659	0.008057	0.5132	0.1125325	0.006599	0.5131	0.06822092	0.002179	0.74988	0.0764145	0.0019615	0.84377
O08997	11927	0.1203491	0.001824	0.992	0.1157836	0.004123	0.9587	0.07545672	0.001428	0.98588	0.09787491	0.0062503	0.88457
Q64737	14450	0.0735796	0.001905	0.9822	0.0755013	0.003138	0.9602	0.05834545	0.002362	0.96065	0.06355312	0.0022307	0.97244
P68368	22145	0.1031116	0.001868	0.9954	0.0978287	0.002665	0.9912	0.0806351	0.004758	0.95352	0.09500169	0.0022148	0.99352
Q3V1I7	NA	0.1359497	0.013103	0.9389	0.1066225	0.067984	0.4505	0.17714773	0.045301	0.65653	0.29976778	0.2350846	0.2454
Q3TEI4	74211	0.0276119	0.001888	0.9469	0.0275661	0.003272	0.8765	0.0239214	0.001555	0.94415	0.02291704	0.0019752	0.93085
Q80SU7	1E+08	0.0212936	0.000974	0.9715	0.0194584	0.001174	0.9582	0.01200336	0.000859	0.9331	0.01327996	0.000794	0.95887
Q6PHN9	77407	0.1504396	0.0039	0.9907	0.1473759	0.003748	0.9923	0.11744535	0.003343	0.98957	0.12691278	0.0021721	0.99679
Q8BVI5	228960	0.1043647	0.008758	0.9103	0.0878198	0.003761	0.9802	0.08534155	0.011284	0.80336	0.10898853	0.0215346	0.69957
Q8BFS6	223978	0.0524649	0.002949	0.9664	0.0510739	0.005043	0.9031	0.04655855	0.003534	0.93031	0.04342223	0.004554	0.89207
P17183	13807	0.060687	0.001981	0.9853	0.0548254	0.002759	0.9705	0.04426842	0.001674	0.98038	0.04144084	0.0014056	0.98638
Q9WUZ7	50795	0.154827	0.003212	0.994	0.1492747	0.007141	0.9733	0.06854297	0.002751	0.97794	0.12076231	0.0051611	0.97855
Q3UHC8	NA	0.0323149	0.002485	0.9235	0.0320636	0.00285	0.9134	0.02909337	0.002869	0.88015	0.03138516	0.0024251	0.93315
Q3UGR5	76987	0.0614975	0.001008	0.9881	0.0611216	0.001386	0.9798	0.04405704	0.00122	0.96737	0.04933132	0.0011857	0.97797
ENSMUSP0(	NA	0.0361738	0.002929	0.9159	0.0363857	0.003239	0.9132	0.03311631	0.002683	0.91582	0.03492222	0.0029875	0.91927
Q99KR7	105675	0.0274853	0.001097	0.9559	0.0237965	0.001246	0.9359	0.01789015	0.000906	0.93071	0.0198952	0.0009633	0.94463
Q3LAC4	109294	0.0015202	0.001132	0.1141	0.0010088	0.004232	0.0047	0.00216883	0.001253	0.17634	0.00079044	0.0005443	0.14949
Q9D3D9	66043	0.0249887	0.00069	0.9682	0.0233622	0.000847	0.9536	0.01846611	0.000779	0.93348	0.01801804	0.0009445	0.91227
O55143	11938	0.2005582	0.017164	0.3746	0.1789398	0.016202	0.3753	0.12817494	0.015132	0.24341	0.15498104	0.0154911	0.33133
Q9CQ60	66171	0.0559777	0.003583	0.8386	0.0574611	0.002696	0.9099	0.0430386	0.001991	0.90513	0.04678715	0.0024541	0.88766
Q922U2	110308	0.337935	0.103641	0.2825	0.322983	0.142355	0.1896	0.50807986	0.120852	0.39563	0.32374857	0.1301575	0.21198
P68037	1.01E+08	0.1245489	0.002657	0.9937	0.1202339	0.003737	0.9885	0.0955797	0.003043	0.98601	0.11568171	0.0029154	0.99244
P19157	14870	0.058269	0.002548	0.9475	0.0585399	0.007763	0.6946	0.04160575	0.004281	0.76511	0.0483419	0.0027666	0.92432
P99029	54683	0.035481	0.00041	0.9807	0.0335317	0.000483	0.974	0.02627685	0.00041	0.96492	0.0278134	0.0004369	0.96915
P35486	18597	0.0429314	0.000442	0.9784	0.038715	0.000632	0.9539	0.0303497	0.000612	0.92209	0.03161991	0.0005785	0.94287
P56380	66401	0.0735596	0.005611	0.8957	0.0565783	0.005139	0.8645	0.04635906	0.003316	0.90298	0.04709042	0.0043138	0.84415
Q9WUB3	19309	0.1185413	0.003623	0.7158	0.1116863	0.003731	0.7017	0.06723037	0.000916	0.92623	0.07570796	0.003385	0.56381
P04247	17189	0.0467006	0.001297	0.7712	0.0489117	0.00593	0.1684	0.03664235	0.001392	0.64593	0.04171037	0.0018987	0.59098

P63325	67097	0.1524876	0.007726	0.9653	0.1310047	0.003844	0.9898	0.09348915	0.00405	0.97618	0.08881996	0.0017578	0.99532
Q8CE68	NA	0.1712905	0.014808	0.6172	0.1600379	0.015029	0.5894	0.09988916	0.00678	0.70921	0.12948911	0.0221325	0.30774
P11352	NA	0.0393476	0.002051	0.8998	0.055943	0.003274	0.8875	0.03934464	0.002892	0.82597	0.05143882	0.0029505	0.90745
Q91V92	104112	0.1227519	0.008398	0.9553	0.130863	0.010766	0.9426	0.12323809	0.012757	0.88606	0.14504533	0.0142563	0.9119
P30285	640611	0.0255694	0.00867	0.3832	0.0241626	0.018418	0.1254	0.02028811	0.007179	0.36325	0.01705291	0.009149	0.22451
Q9CQH3	66046	0.0291604	0.00093	0.9637	0.0262316	0.000724	0.9733	0.02075182	0.000539	0.97368	0.02199941	0.0009102	0.94347
P51174	NA	0.027063	0.000837	0.8059	0.0247021	0.001532	0.5362	0.0203206	0.001073	0.58252	0.019661	0.0004511	0.89409
Q9ESN3	60455	0.1076559	0.002901	0.9906	0.0965068	0.003497	0.9845	0.0836473	0.004241	0.96526	0.08289093	0.0030059	0.98447
P00397	NA	0.0670139	0.004786	0.8167	0.0688597	0.00523	0.8202	0.05433207	0.004998	0.70687	0.05778445	0.0050055	0.76473
P17742	268373	0.091799	0.003439	0.889	0.0926616	0.003422	0.9049	0.06703881	0.003764	0.78092	0.08448585	0.0037891	0.86589
OTTMUSP0(	NA	0.0340747	0.002471	0.9314	0.0247393	0.002597	0.8832	0.01627098	0.000921	0.95705	0.01813955	0.0017502	0.89951
P97443	12180	0.3050019	0.051693	0.6719	0.3708463	0.094064	0.5445	0.31746438	0.129555	0.30016	0.27506725	0.0639753	0.58712
Q9ERS2	67184	0.0445323	0.005776	0.5431	0.0505453	0.02948	0.0613	0.0411079	0.005958	0.48278	0.03700623	0.0065925	0.41184
O88685	19182	0.1832784	0.006862	0.952	0.1796134	0.008151	0.9436	0.18200218	0.032537	0.49439	0.14977091	0.0065257	0.94442
Q8VE95	223665	0.057148	0.003709	0.9481	0.0475104	0.003188	0.9487	0.03498746	0.00268	0.92412	0.04275492	0.0054109	0.83879
O35683	54405	0.0184303	0.003409	0.6762	0.0218336	0.003295	0.7853	0.01667156	0.004188	0.5309	0.01657173	0.0034779	0.65422
Q08AU7	68515	0.0027791	0.002303	0.0942	0.0031587	0.00437	0.0417	0.00350938	0.00431	0.04853	0.00326381	0.0070222	0.02115
B0QZL1	NA	0.0437687	0.002214	0.9726	0.0424817	0.001776	0.9811	0.03137169	0.001492	0.96932	0.0340074	0.0014301	0.98092
P17950	NA	0.0244542	0.004879	0.834	0.0188813	0.001717	0.9527	0.01555321	0.000435	0.99533	0.01851884	0.0038479	0.74328
XP_0014767	NA	0.1050756	0.004448	0.9807	0.3210428	0.079228	0.5988	0.14265386	0.009772	0.95091	0.19625675	0.028695	0.83864
XP_0014765	NA	0.0281226	0.006245	0.5916	0.0271197	0.007241	0.539	0.03256351	0.016628	0.2278	0.02246273	0.006299	0.5145
P50544	11370	0.0667572	0.004783	0.3143	0.0575135	0.003477	0.4271	0.04230749	0.002876	0.33479	0.04505155	0.0013043	0.76037
Q60994	NA	0.145728	0.010154	0.8766	0.2705613	0.055768	0.4849	0.1020673	0.009442	0.80116	0.0942012	0.0067321	0.88678
Q5DU05	214552	0.0031407	0.000427	0.8064	0.0026995	0.000878	0.4861	0.00316813	0.001034	0.43872	0.0037917	0.0009896	0.59483
P09542	17897	0.0566967	0.001577	0.9048	0.0693942	0.010363	0.2805	0.04776034	0.001241	0.91877	0.04434027	0.0013862	0.89976
Q91Y97	230163	0.1902796	0.049914	0.645	0.3458149	0.181121	0.313	0.13349423	0.035242	0.61454	0.25292613	0.0953551	0.43875
Q9JJ26	54483	0.0006103	9.14E-05	0.7612	0.0006211	0.000159	0.5601	0.00052324	0.000117	0.58936	0.00052237	0.0001152	0.63159
Q4ACU6	58234	0.0207336	0.001222	0.96	0.0295149	0.004984	0.8539	0.02023333	0.002055	0.89811	0.02206338	0.0054673	0.7651
Q8BIJ6	636969	0.1529091	0.03715	0.4714	0.1693859	0.13129	0.1135	0.12819913	0.031542	0.47855	0.11403996	0.0224068	0.63328
P07309	22139	1.128945	0.089593	0.8593	1.0018871	0.112386	0.791	0.91994106	0.063626	0.88562	1.07986238	0.086873	0.86073
O70468	NA	0.0912816	0.010032	0.2828	0.0790693	0.006798	0.4304	0.05991098	0.005993	0.33095	0.07185574	0.0115466	0.17706
P00920	12349	0.0168913	0.000501	0.8809	0.0417721	0.009564	0.1238	0.03504801	0.009289	0.08615	0.02307233	0.0011876	0.74675
Q9JHI5	56357	0.0353183	0.000755	0.9271	0.0314279	0.001017	0.8659	0.03583837	0.006893	0.1386	0.0280932	0.0010641	0.82192
Q05512-2	NA	0.070112	0.003857	0.9706	0.0691995	0.002603	0.9888	0.05103772	0.004078	0.93999	0.06066981	0.0047946	0.95241
P02535	NA	0.0305925	0.008171	0.3336	0.1003901	0.090655	0.0578	0.06028401	0.067202	0.04789	0.04460808	0.0236234	0.17338
P62855	1E+08	0.4101483	0.082561	0.638	0.7264549	0.165085	0.6377	0.31374886	0.078885	0.58984	0.17456106	0.0383214	0.63359
Q3V3T4	NA	0.0289092	0.000716	0.9933	0.0245354	0.001851	0.9461	0.02028317	0.00155	0.93455	0.01975273	0.0021013	0.89834
Q9QXX4	50799	0.0253699	0.001009	0.8802	0.0259964	0.001173	0.866	0.01891075	0.000953	0.81386	0.0201063	0.0008253	0.89857
P70404	15929	0.0586412	0.002243	0.8381	0.0472976	0.001436	0.9012	0.03578095	0.001167	0.87362	0.03982648	0.001512	0.8557
Q07417	11409	0.0308528	0.002333	0.4429	0.0328373	0.00746	0.0948	0.02140267	0.002311	0.28515	0.0242696	0.00287	0.2745
P04104	16678	0.02221	0.004168	0.6697	0.019894	0.016185	0.1118	0.037419	0.011903	0.41378	0.02172913	0.0217388	0.08327
P09541	NA	0.2124937	0.037711	0.2428	0.2143232	0.043285	0.2122	0.17490912	0.036608	0.18142	0.275844	0.0472752	0.29338

Q9D0M3	66445	0.0220688	0.000408	0.9566	0.021591	0.000486	0.9444	0.01613289	0.000406	0.92354	0.01648481	0.0005168	0.89767
A2AQP0	668940	0.0015128	0.001438	0.0733	0.0015987	0.002359	0.0369	0.00272692	0.002191	0.09959	0.00149575	0.0012729	0.10319
Q61171	1.01E+08	0.0741124	0.002312	0.9057	0.0812633	0.001873	0.9534	0.06469549	0.001893	0.91539	0.07004336	0.001179	0.97406
XP_207492	NA	0.1800003	0.032506	0.7541	0.3552726	0.280272	0.1673	0.20668834	0.116776	0.20702	0.23011411	0.1913352	0.15312
P48036	11747	0.0763347	0.001783	0.9381	0.0826179	0.001608	0.9593	0.05313183	0.001389	0.91785	0.07617516	0.001305	0.96818
XP_904967	NA	0.0907232	0.001439	0.9965	0.0901954	0.002285	0.9924	0.06494683	0.0016	0.99157	0.07405436	0.0023953	0.9876
P31786	13167	0.0603176	0.001201	0.9829	0.0604439	0.0013	0.9827	0.04110861	0.002736	0.83691	0.05129546	0.0012642	0.97744
Q9CQJ8	66218	0.0306267	0.000973	0.9332	0.0283998	0.001554	0.8477	0.0199095	0.000846	0.88636	0.02313362	0.0015443	0.78079
P18872	14681	0.0078276	0.003794	0.2331	0.0100086	0.002162	0.6608	0.01674666	0.022193	0.03908	0.01106016	0.0065428	0.19233
O70456	55948	0.0000512	1.07E-05	0.6201	0.0001446	0.000104	0.1627	0.0000376	2.05E-05	0.20505	0.0000276	4.58E-06	0.76737
Q9CRB9	66075	0.0248342	0.002429	0.6553	0.0231425	0.002076	0.7172	0.02190826	0.002845	0.5055	0.01919939	0.0026021	0.49745
Q9R244	22064	0.0111796	0.003272	0.6251	0.0068758	0.007673	0.0819	0.01201618	0.003026	0.69254	0.00769937	0.0053373	0.34221
P62962	18643	0.1117622	0.00188	0.9841	0.1118585	0.00218	0.981	0.09179436	0.003446	0.92563	0.10325446	0.0022195	0.9783
Q9DCV4	66302	0.0207596	0.00585	0.4404	0.0237157	0.007486	0.4357	0.0211116	0.003589	0.65784	0.02652747	0.0075307	0.45272
P23953	13884	0.9556905	0.108036	0.7098	0.884447	0.128475	0.6371	0.71458281	0.097435	0.6497	0.7993231	0.1333338	0.57101
Q06770	12401	0.7417983	0.070851	0.6425	0.7537848	0.078747	0.6165	0.74983716	0.063276	0.70064	0.68099117	0.0945938	0.48515
Q8BH95	93747	0.0277616	0.002364	0.5496	0.0278399	0.004982	0.2379	0.02122034	0.001789	0.55238	0.02207136	0.0023365	0.48174
P70398	22284	0.041981	0.009746	0.5699	0.0224515	0.005437	0.6079	0.02300877	0.014007	0.16159	0.02741054	0.0055423	0.67087
Q8K183	216134	0.0872659	0.002586	0.9879	0.0850041	0.002551	0.9893	0.06165165	0.003495	0.95695	0.06774992	0.0038361	0.96295
P03903	17720	0.0095346	0.001829	0.6765	0.0137148	0.002243	0.7727	0.01149372	0.001446	0.81858	0.01052782	0.0034747	0.45491
Q8CDU4-2	NA	0.0027976	0.000713	0.5419	0.0010558	0.000407	0.3796	0.00163259	0.000493	0.47795	0.00248568	0.0012241	0.27264
Q9DB20	1E+08	0.0256092	0.001017	0.8431	0.0460419	0.014344	0.0988	0.01969697	0.000746	0.85195	0.01846538	0.0007985	0.84115
Q9DCX2	71679	0.078742	0.022928	0.0691	0.0738329	0.019467	0.0944	0.06793944	0.019061	0.07399	0.04656683	0.0136289	0.07748
Q9D023	70456	0.0580741	0.001523	0.9732	0.0544632	0.001809	0.9618	0.04186205	0.001422	0.95587	0.04570983	0.0017987	0.94998
O08677	16644	0.891754	0.069484	0.6703	0.9401128	0.054102	0.7724	0.75400016	0.032871	0.83496	0.91640144	0.051088	0.78716
Q8QZS1	227095	0.0302702	0.000936	0.876	0.027779	0.000789	0.9071	0.02159986	0.000763	0.84502	0.02202263	0.0005964	0.91541
XP_484732	NA	0.0863854	0.001608	0.9952	0.0937125	0.007531	0.9281	0.06994211	0.00194	0.98934	0.07240893	0.0017412	0.99311
Q9CQQ7	11950	0.0250565	0.000777	0.9147	0.0241063	0.000822	0.911	0.02207649	0.001983	0.55352	0.01940001	0.0008436	0.85873
P18242	13033	0.4218622	0.061609	0.6346	0.1879961	0.009052	0.935	0.12336095	0.004621	0.95318	0.18068971	0.013919	0.83211
P53810	18738	0.11423	0.005997	0.9654	0.116457	0.004512	0.9852	0.08231963	0.003977	0.97276	0.1012245	0.0085227	0.9338
P56716	19888	0.00929	0.003396	0.3653	0.0081898	0.00318	0.3988	0.00711993	0.002716	0.38443	0.0105547	0.0039566	0.41576
Q8BZ25	244859	0.6231656	0.176975	0.6078	0.4450905	0.18904	0.4802	0.98691401	0.29615	0.68955	0.8724169	0.1903899	0.72411
Q3TC72	NA	0.036581	0.003067	0.8506	0.090501	0.055029	0.1191	0.02716983	0.002158	0.85911	0.03223749	0.0023233	0.89329
B2RXC6	NA	0.9938052	0.158062	0.7385	0.6549337	0.214895	0.4363	0.95524008	0.15579	0.72866	0.98939776	0.113259	0.86412
P12979	17928	0.0145155	0.000138	0.9987	0.013958	0.000495	0.9851	0.01160571	0.000416	0.98233	0.01016225	0.0002147	0.99467
Q62465	26949	0.0444819	0.010509	0.8175	0.0532886	0.066	0.2458	0.18347228	0.36945	0.10977	0.0574568	0.0125825	0.87422
Q9CR61	66916	0.0297232	0.001088	0.8956	0.0279938	0.002284	0.664	0.02146649	0.001229	0.78218	0.02128943	0.0010542	0.84117
Q8VCT4	104158	0.4271483	0.047831	0.5963	0.3437689	0.040783	0.5728	0.21564229	0.034056	0.4046	0.23275347	0.0436811	0.32865
P24472	14860	0.0858626	0.0143	0.9001	0.0793965	0.003096	0.9836	0.06004809	0.002747	0.97154	0.0719832	0.0053305	0.94801
Q8BK30	78330	0.0308264	0.001076	0.9832	0.0325907	0.000967	0.9895	0.0250341	0.001247	0.96644	0.02643113	0.0014169	0.96667
Q9DCM2	76263	0.0377246	0.001874	0.8729	0.0415305	0.001972	0.8987	0.09446128	0.030052	0.14555	0.03321802	0.0021727	0.8209
B9EHC7	NA	0.0491901	0.001725	0.9656	0.0534931	0.002411	0.9517	0.03887081	0.001899	0.93526	0.04345062	0.0017372	0.96157

P56399	22225	0.1366732	0.002037	0.9943	0.1423868	0.002578	0.9919	0.10025653	0.002428	0.98441	0.11944496	0.0017533	0.99464
Q9JLT4	NA	0.0300483	0.004758	0.7402	0.0427524	0.124291	0.0098	0.01895715	0.002848	0.77312	0.02209198	0.0033093	0.78785
Q91VM9	74776	0.0347072	0.000846	0.9579	0.0316459	0.00106	0.933	0.02328487	0.000753	0.92822	0.02688638	0.0008999	0.93309
P17563	20341	0.0824353	0.017864	0.3364	0.0706759	0.006552	0.7394	0.07493202	0.024139	0.17319	0.05358947	0.0063755	0.63851
P49722	NA	0.1187497	0.008188	0.8538	0.1292242	0.005358	0.9387	0.10479306	0.005747	0.89987	0.1039783	0.0049398	0.92679
Q99J27	11416	0.0853256	0.001814	0.9937	0.086927	0.002247	0.992	0.06879781	0.00124	0.99547	0.07267901	0.001937	0.99155
P52480-2	18746	0.1024781	0.003452	0.9535	0.0996841	0.003232	0.9635	0.07495357	0.002715	0.94776	0.08128306	0.002769	0.95776
P17751	21991	0.0640901	0.001441	0.9304	0.063769	0.001362	0.9456	0.04564927	0.001102	0.92208	0.052867	0.0012456	0.93511
Q9D172	1E+08	0.0332845	0.0009	0.9231	0.0306129	0.000975	0.907	0.02491556	0.000808	0.89302	0.02615143	0.0009861	0.87662
P14131	1.01E+08	0.1093129	0.0038	0.9845	0.1033608	0.003082	0.9912	0.06983477	0.005275	0.94602	0.0768301	0.002333	0.98906
Q00623	NA	0.8993119	0.057349	0.7834	0.9916595	0.076617	0.7495	0.78401499	0.042918	0.82661	0.91763869	0.0733437	0.73652
Q91WD5	226646	0.0627315	0.010968	0.1449	0.0707017	0.014082	0.1305	0.06312764	0.015296	0.08188	0.0563424	0.0178384	0.05637
Q8JZQ2	69597	0.2474709	0.095029	0.4041	0.7164488	0.131823	0.7869	0.2365337	0.090195	0.34599	0.0553606	0.0047089	0.93253
P20152	22352	0.1887723	0.022571	0.311	0.1485155	0.019925	0.2729	0.11935327	0.017833	0.22762	0.17268826	0.0233432	0.28396
P12382	18641	0.0843776	0.007184	0.9079	0.1011613	0.011462	0.8665	0.07155851	0.007949	0.85268	0.07728227	0.0115946	0.78734
Q99L13	58875	0.0487446	0.009475	0.1807	0.0325891	0.002603	0.5811	0.02981792	0.003106	0.41115	0.05129881	0.0177159	0.07083
P41251	18173	0.3364303	0.175646	0.2341	0.3467919	0.180412	0.2514	0.59574406	0.198863	0.42788	0.36273493	0.1424852	0.37074
Q3U0B3	192970	0.0916304	0.010664	0.8406	0.0758756	0.011509	0.798	0.18090303	0.078584	0.27459	0.10670986	0.0235794	0.63055
Q7TQS8	NA	0.0512538	0.012923	0.5291	0.0740375	0.016078	0.6386	0.06294914	0.018002	0.46621	0.059914	0.0151258	0.56663
O55124	NA	0.0729537	0.004072	0.4962	0.0642898	0.00226	0.7356	0.06520956	0.001754	0.81251	0.04728254	0.0014533	0.78904
O35963	19338	0.1984628	0.00805	0.9775	0.2151026	0.019509	0.9102	0.14264704	0.004621	0.98552	0.156796	0.003419	0.99433
P06728	11808	0.4168108	0.122469	0.3166	0.3811861	0.123808	0.358	0.32045164	0.089006	0.34146	0.43655449	0.1386156	0.34298
O08709	NA	0.0652022	0.002	0.8763	0.0623081	0.002279	0.847	0.05956175	0.011154	0.15455	0.05778965	0.0029813	0.73424
Q9WTP7	56248	0.063812	0.009819	0.8407	0.0802636	0.010589	0.8518	0.06113039	0.006914	0.86691	0.04879752	0.009507	0.79008
Q791V5	56428	0.0313628	0.001887	0.914	0.0292583	0.002295	0.8621	0.0211475	0.001845	0.8041	0.01908519	0.0024476	0.73431
P14152	17449	0.0666171	0.004848	0.4456	0.0599623	0.001863	0.8314	0.04457337	0.001658	0.74926	0.05733891	0.0060514	0.2985
P42125	NA	0.0394556	0.012634	0.0881	0.0585264	0.028603	0.0475	0.02703122	0.005762	0.1834	0.02472243	0.005089	0.21933
Q6NZQ8	72925	0.043887	0.00309	0.9351	0.0324628	0.003788	0.8596	0.01894223	0.001253	0.94229	0.03169573	0.0032641	0.8871
O35593	59029	0.1800986	0.015767	0.9422	0.1704846	0.009012	0.9755	0.09710563	0.010219	0.94753	0.1167108	0.0048073	0.98992
Q99LB2	NA	0.026009	0.000869	0.8699	0.0246439	0.001334	0.7463	0.03423099	0.009288	0.09203	0.02042899	0.0007402	0.86783
Q921G7	66841	0.0434259	0.005344	0.1989	0.0525693	0.008908	0.132	0.02710779	0.002313	0.34053	0.02828646	0.0055966	0.09919
P40142	21881	0.1063549	0.025972	0.1217	0.0773581	0.015181	0.1924	0.074055	0.017829	0.1196	0.08048784	0.012301	0.29162
P12787	12858	0.1325549	0.027294	0.1631	0.0735673	0.025526	0.0767	0.09452699	0.025956	0.10104	0.09571995	0.0303256	0.0882
P47934	12908	0.0334108	0.002035	0.701	0.0440549	0.013501	0.0989	0.03767808	0.006151	0.2493	0.03066587	0.0037147	0.41517
P13020	227753	0.1947934	0.047861	0.441	0.0910167	0.043638	0.3522	0.23144883	0.066454	0.44711	0.14095588	0.0334447	0.57741
Q9WVK4	13660	0.080146	0.003987	0.9735	0.0712775	0.004325	0.9679	0.03852833	0.00416	0.85968	0.06064038	0.0104417	0.78936
Q8C2L9	NA	0.0077034	0.002331	0.686	0.0101999	0.007631	0.3733	0.00871274	0.001781	0.70533	0.02360895	0.0249895	0.2293
P29699	11625	0.8510286	0.082751	0.7306	0.7972117	0.100947	0.6093	0.78825779	0.062402	0.79956	0.73715269	0.0819605	0.64255
Q3UMP2	NA	0.0739773	0.001937	0.9905	0.0689764	0.003301	0.9732	0.04409275	0.001979	0.97258	0.06070725	0.0014096	0.99357
P59997	NA	0.0129334	0.003838	0.4479	0.0090407	0.001984	0.6337	0.01569317	0.00893	0.18072	0.01166751	0.0025062	0.64363
O55103	19153	0.0147961	0.003714	0.6134	0.0297272	0.00788	0.7034	0.01422082	0.00372	0.54914	0.01334858	0.0054346	0.42991
Q8K370	71985	0.0319173	0.001149	0.9279	0.0336085	0.002815	0.7072	0.02124412	0.001083	0.85171	0.02636648	0.0008411	0.94699

Q9CZ42	69225	0.0259673	0.004139	0.6631	0.0314708	0.004737	0.6776	0.02320285	0.003071	0.74054	0.02063392	0.0038699	0.62579
Q80W27	NA	0.0340268	0.00125	0.992	0.0323095	0.000641	0.9976	0.02829141	0.003583	0.8863	0.03015303	0.0018394	0.97816
P34914	13850	0.0936697	0.002085	0.9148	0.0939595	0.002215	0.9188	0.07082275	0.002603	0.80179	0.08223205	0.0019629	0.91789
O70251	55949	0.1727445	0.006854	0.9799	0.1580038	0.006671	0.9808	0.10689588	0.014025	0.8288	0.11688592	0.0042961	0.98799
Q924D0	NA	0.0427685	0.010182	0.4137	0.0661287	0.018355	0.382	0.02325636	0.004518	0.51454	0.03398079	0.0028821	0.86336
P80313	12468	0.1870276	0.011876	0.9538	0.2101727	0.01586	0.936	0.15763785	0.099089	0.17417	0.18506423	0.0105919	0.96828
Q9D8B4	NA	0.0433841	0.001445	0.9772	0.0416989	0.002106	0.9538	0.03448201	0.001539	0.95435	0.03986591	0.001617	0.9666
Q9CZR8	66399	0.0609372	0.002194	0.9626	0.0595038	0.002085	0.9691	0.04028025	0.001703	0.95397	0.04955691	0.0022557	0.94517
Q99LX0	57320	0.0570947	0.001075	0.9631	0.0597261	0.001517	0.9417	0.04165947	0.000897	0.94982	0.0492906	0.0013157	0.93102
Q9CQR4	66834	0.0314871	0.001585	0.8935	0.0312576	0.001855	0.8632	0.02253892	0.002181	0.67246	0.03133105	0.0023293	0.79729
Q8CDI6	320696	0.0002051	7.78E-05	0.3317	0.0003913	8.98E-05	0.6127	0.0003453	0.000102	0.45021	0.00037215	0.0001222	0.43592
Q9CPP6	68202	0.0389961	0.001277	0.9609	0.0444927	0.004047	0.7856	0.02628946	0.000718	0.96964	0.03105932	0.0018071	0.89407
P03921	NA	0.0286111	0.001356	0.9119	0.0273183	0.001668	0.8759	0.02257089	0.00186	0.77808	0.0257477	0.0013904	0.9098
Q9CRB8	67900	0.0500982	0.00134	0.9811	0.0453268	0.001053	0.9872	0.03796141	0.000895	0.98415	0.03621428	0.0016042	0.95324
Q91X72	15458	0.5222961	0.046709	0.5187	0.648579	0.043795	0.658	0.50457265	0.038215	0.55285	0.64977838	0.0429387	0.63966
Q9QUM9	26443	0.3220431	0.064428	0.4998	0.1918684	0.013924	0.892	0.16891717	0.038685	0.39667	0.13010318	0.0099344	0.87724
P62631	13628	0.105463	0.00611	0.7582	0.1000926	0.015756	0.3325	0.08991839	0.014436	0.29891	0.09392731	0.0182949	0.25258
Q8CHT0	212647	0.0240596	0.002311	0.6514	0.0225999	0.002767	0.5716	0.01750037	0.001974	0.57131	0.01712061	0.003272	0.34931
O88441	53375	0.0406935	0.00269	0.8841	0.03585	0.002278	0.905	0.02609588	0.00161	0.89443	0.03012143	0.002852	0.79934
Q02053	22201	0.1311216	0.015073	0.483	0.1167798	0.004464	0.8849	0.0883644	0.005339	0.77395	0.10069069	0.0033178	0.92749
Q9CPU0	109801	0.1759697	0.040026	0.2093	0.0971685	0.028236	0.1561	0.14439394	0.045874	0.11533	0.12169966	0.0318689	0.17447
P54071	269951	0.0314464	0.001534	0.6214	0.0289888	0.002199	0.4425	0.02728281	0.002032	0.42293	0.02552783	0.0017001	0.51072
P28650	11565	0.0961063	0.006838	0.7916	0.1513442	0.030812	0.3704	0.07508669	0.007387	0.66093	0.09236446	0.0074131	0.76761
Q60759	270076	0.0536394	0.054537	0.0882	0.0951535	0.106546	0.0739	0.32384766	0.170457	0.21732	0.30478012	0.1373614	0.30918
A2AGD7	NA	0.003086	0.002565	0.0937	0.0025271	0.000617	0.5826	0.00521235	0.002219	0.31498	0.00364298	0.0019031	0.24989
XP_487581	NA	0.0038746	0.000897	0.5714	0.0035467	0.00116	0.4377	0.00243859	0.000795	0.40198	0.0047905	0.0007599	0.76809
P80317	NA	0.2463971	0.034154	0.6755	0.2110279	0.013649	0.9157	0.15565963	0.006677	0.9628	0.14587057	0.012868	0.83172
P47857	18642	0.2057659	0.013988	0.516	0.1756067	0.015544	0.4083	0.15371113	0.015956	0.30648	0.13126268	0.0071153	0.65281
P08752	14678	0.1065579	0.004567	0.9749	0.1408508	0.017747	0.84	0.08792328	0.004047	0.9712	0.14514375	0.0084614	0.96082
P52825	12896	0.1282663	0.020488	0.2237	0.1159301	0.019021	0.2291	0.08835277	0.016191	0.17136	0.06749631	0.013752	0.14862
Q99LC3	67273	0.1049288	0.01946	0.1624	0.1045707	0.024557	0.1232	0.05521225	0.013641	0.09906	0.04657663	0.0133956	0.08449
Q8R1S0	217707	0.0532171	0.005513	0.8859	0.0486801	0.013416	0.6869	0.04588312	0.013125	0.54997	0.04356263	0.0057812	0.86318
P25444	667847	0.1103039	0.002526	0.9932	0.115465	0.005606	0.9815	0.0788122	0.002511	0.98697	0.07349547	0.0025673	0.98795
Q80YV2	232679	0.0098811	0.000269	0.9897	0.0131006	0.003129	0.6144	0.010648	0.000964	0.90375	0.01490013	0.0033117	0.62783
P13634	NA	0.0198618	0.002625	0.3073	0.0234803	0.00122	0.7695	0.02715141	0.004715	0.20447	0.02101152	0.0011745	0.74249
P01027	1E+08	0.5446756	0.044196	0.4808	0.5091046	0.050937	0.413	0.44690425	0.03354	0.49796	0.55520843	0.0486785	0.4628
O88844	15926	0.0500316	0.007552	0.511	0.1025828	0.033627	0.2149	0.04945841	0.004526	0.75384	0.05648652	0.0056208	0.75372
P35700	18477	0.1007048	0.004061	0.8662	0.1013434	0.004124	0.8753	0.07455051	0.002886	0.87887	0.0867739	0.0033508	0.88751
Q60988	NA	0.2033187	0.032477	0.7808	0.1771277	0.017443	0.9116	0.14239666	0.007842	0.97343	0.17316942	0.0163561	0.93339
Q9CPU4	66447	0.0496378	0.005806	0.936	0.0486428	0.00481	0.9624	0.05185435	0.032884	0.45321	0.05462646	0.0212306	0.48606
Q99LC5	110842	0.0409743	0.005223	0.1605	0.0304879	0.001902	0.4794	0.02390598	0.001134	0.58463	0.0302851	0.0055572	0.09715
P18760	1E+08	0.1621306	0.003627	0.9657	0.1625277	0.00506	0.9416	0.11158619	0.003377	0.93814	0.1474388	0.004437	0.94684

Q9D2G2	78920	0.0200994	0.00049	0.9172	0.0182398	0.001997	0.3872	0.01495963	0.001346	0.45013	0.01397093	0.001508	0.39403
P62245	1E+08	0.1862285	0.089985	0.2478	0.2835066	0.081505	0.5475	0.13436403	0.017869	0.81306	0.25722227	0.0835265	0.44143
Q9D7B6	NA	0.066061	0.033351	0.0644	0.0842901	0.046967	0.0641	0.07328765	0.04254	0.0479	0.11415136	0.0566572	0.07373
O55143-2	11938	0.1245672	0.010337	0.9732	0.099149	0.00867	0.9703	0.07203802	0.006605	0.95966	0.0943967	0.0056089	0.97588
Q8K1Z0	67914	0.0559443	0.001109	0.9673	0.0580024	0.001325	0.9633	0.04125431	0.001012	0.9524	0.04867441	0.0013364	0.94852
Q99MN9	66904	0.0458201	0.007394	0.1861	0.038929	0.008872	0.1151	0.02677879	0.001519	0.65324	0.02414577	0.0015346	0.63223
A2AR50	241308	0.0017068	0.000484	0.7131	0.0014488	0.002496	0.0404	0.00217791	0.00073	0.59723	0.00126797	0.0012309	0.15028
Q9DBB8	71755	0.0788951	0.015772	0.8929	0.0658718	0.008469	0.938	0.02136172	0.005548	0.88114	0.04688534	0.0077379	0.92446
Q9DCT2	624814	0.1085699	0.023186	0.1513	0.0944055	0.02813	0.0952	0.07280015	0.018466	0.11138	0.06822964	0.0251868	0.06308
Q6NSW3	NA	0.0010198	0.000268	0.5088	0.0005756	0.000215	0.3732	0.00106762	0.00026	0.54687	0.00099686	0.0003137	0.45704
Q9CW46	71766	0.0380265	0.014082	0.3987	0.0460313	0.040808	0.1037	0.05854397	0.030019	0.21363	0.05388404	0.0262432	0.27707
Q9DC61	66865	0.1183085	0.001858	0.9941	0.1104072	0.005194	0.9417	0.06853879	0.002375	0.96972	0.09842666	0.0021446	0.9892
OTTMUSP0(	NA	0.0199288	0.001759	0.9211	0.0284586	0.012092	0.4091	0.01937849	0.003151	0.82542	0.02835492	0.0058172	0.70378
O35129	12034	0.0193405	0.001841	0.6675	0.0207806	0.002663	0.5541	0.01512992	0.001548	0.6346	0.01750068	0.0028488	0.45068
Q9DBP5	66588	0.156061	0.02646	0.713	0.0990806	0.007599	0.9341	0.07036163	0.003138	0.97291	0.08946377	0.001799	0.99517
O88668	433375	0.3894151	0.157528	0.5046	0.923518	0.154887	0.8556	0.70444657	0.128818	0.81032	0.95157848	0.0961693	0.899
P34884	17319	0.0513482	0.001507	0.9798	0.0513929	0.001878	0.9715	0.03987156	0.002093	0.94042	0.04099014	0.0012653	0.97947
ENSMUSP0(	NA	0.0006805	0.000474	0.1283	0.0018431	0.007905	0.0045	0.00192518	0.006243	0.00726	0.00177389	0.0046008	0.01224
Q9Z2K1	16666	0.0197087	0.008173	0.3677	0.0222765	0.008722	0.3948	0.03438578	0.007287	0.73571	0.03278553	0.0137065	0.44975
Q9CPQ8	1E+08	0.0263199	0.000794	0.9743	0.0268898	0.000992	0.9671	0.02031827	0.000626	0.97319	0.02027978	0.0010397	0.93834
Q9CQC7	1E+08	0.0542949	0.005565	0.7665	0.0635233	0.008172	0.7073	0.04519206	0.00515	0.72639	0.04607813	0.0062753	0.68321
Q8JZN5	229211	0.0660218	0.015096	0.7051	0.142805	0.041426	0.8559	0.03961466	0.008982	0.82942	0.07978592	0.0383978	0.38149
Q9Z2W0	13437	0.103105	0.004307	0.9679	0.1121449	0.004899	0.9704	0.07955707	0.003817	0.94971	0.09424624	0.0076572	0.88338
Q9CWR0	NA	0.070865	0.021269	0.4423	0.0632373	0.022234	0.4027	0.05030216	0.020046	0.34415	0.12918784	0.0454923	0.44642
O09161	12373	0.1344308	0.043997	0.1134	0.1984099	0.048934	0.2044	0.13310665	0.039736	0.14531	0.09967722	0.0363589	0.11299
Q8BFZ3	238880	0.1226898	0.00349	0.9888	0.1341449	0.005347	0.9813	0.104208	0.010381	0.87801	0.12478825	0.0044164	0.98519
Q8R0P4	66273	0.2324129	0.068947	0.448	0.17677	0.019474	0.8729	0.07896831	0.014562	0.67748	0.19245571	0.0212069	0.87283
Q91VA7	NA	0.0704653	0.007058	0.3603	0.0592744	0.000817	0.9719	0.04097588	0.000896	0.92196	0.04703231	0.001135	0.9177
Q3UM45	66385	0.138585	0.016833	0.8714	0.1025322	0.004926	0.9774	0.0731784	0.004122	0.96332	0.07931759	0.0035194	0.98449
A6H6E2	105450	0.0980747	0.003903	0.9783	0.1094124	0.01116	0.889	0.07625771	0.006088	0.92347	0.09447441	0.0200988	0.64804
ENSMUSP0(	NA	0.0256477	0.002437	0.9022	0.0187434	0.002441	0.8428	0.01688946	0.002399	0.7797	0.01840661	0.002515	0.81697
Q9CQ65	66902	0.0553375	0.006397	0.852	0.0535366	0.004563	0.9198	0.03931757	0.004393	0.8512	0.04532665	0.005164	0.86523
P10126	13627	0.3833518	0.07056	0.4959	0.337168	0.05407	0.5486	0.19768176	0.030543	0.55198	0.21119927	0.0560245	0.33667
Q9DC69	66108	0.0525989	0.011056	0.1846	0.0630641	0.014349	0.1888	0.02828182	0.001809	0.71181	0.049321	0.0134582	0.13112
Q9ERI6	105014	0.0736364	0.00179	0.9941	0.0872164	0.004536	0.9788	0.06072638	0.009272	0.8428	0.06723655	0.0051554	0.94447
P51667	17906	0.0582065	0.001174	0.9654	0.0510276	0.00177	0.9152	0.04035458	0.01542	0.07221	0.0443385	0.0013215	0.93832
Q61292	16779	0.9332372	0.275989	0.9196	1.3531328	0.260774	0.9309	0.94976146	0.522896	0.76739	1.37480309	0.4921842	0.88639
P20065	19241	0.5283105	0.069217	0.8062	0.6809514	0.081728	0.8526	0.3797217	0.078697	0.62448	0.50237506	0.0762269	0.78353
Q9DBJ1	18648	0.0643482	0.002221	0.9406	0.0680264	0.002269	0.9473	0.04809543	0.002671	0.85724	0.05430697	0.0023616	0.92157
P53986	20501	0.1354822	0.007435	0.9736	0.4138004	0.146222	0.5717	0.09625991	0.006872	0.96083	0.12408288	0.0034328	0.99391
P56565	NA	0.1101496	0.004972	0.9009	0.1203044	0.003943	0.949	0.07846942	0.003748	0.89396	0.10829287	0.0040195	0.94407
Q9CQN6	66154	0.0301261	0.003531	0.8198	0.0211179	0.002543	0.8414	0.02937191	0.00248	0.90337	0.01920436	0.003243	0.72955

Q9D8T7	380773	0.0576086	0.016314	0.4711	0.0464065	0.00882	0.7346	0.04265687	0.00679	0.75223	0.04738852	0.0131032	0.52152
Q8R164	68021	0.1036555	0.067225	0.0758	0.0305632	0.004601	0.6383	0.02011979	0.005507	0.32282	0.02243786	0.0022469	0.80602
P61014	18821	0.5244728	0.085801	0.7274	0.5365801	0.115214	0.6438	0.27663373	0.079299	0.46502	0.36515025	0.0595122	0.75829
Q9EQI5	NA	0.3278507	0.085809	0.5289	0.1572572	0.144331	0.1165	0.58000842	0.162783	0.47557	0.54743415	0.2145831	0.4818
P56480	11947	0.036988	0.002961	0.239	0.02897	0.000888	0.7116	0.02740068	0.001064	0.57421	0.02598523	0.0011004	0.56232
P01864	NA	0.0562109	0.006393	0.7557	0.3133612	0.0703	0.4529	0.10477009	0.010934	0.78599	0.24461487	0.077756	0.2836
O08749	13382	0.0430392	0.008524	0.1126	0.0237361	0.00112	0.715	0.01816566	0.000462	0.88478	0.02968802	0.0072677	0.08705
Q5EBG6	243912	0.3483302	0.040157	0.5523	0.3169648	0.034934	0.6039	0.18184922	0.005623	0.95525	0.19970373	0.005077	0.96447
Q62388	11920	0.0781914	0.012508	0.8669	0.1090036	0.015643	0.8292	0.05553848	0.007732	0.88055	0.11831968	0.0306852	0.65017
Q00897	20703	0.7876539	0.12687	0.5881	0.7725186	0.170705	0.5059	0.63004797	0.089181	0.62458	0.6385613	0.1293724	0.52548
Q9DCW4	110826	0.0351744	0.0016	0.7583	0.0279633	0.000313	0.9836	0.02526319	0.001901	0.53109	0.02443644	0.0014804	0.66543
ENSMUSP0(	NA	0.0051125	0.000723	0.7811	0.0058967	0.000518	0.9152	0.00375507	0.000434	0.8426	0.00336027	0.0005601	0.74994
P56501	22229	0.1058835	0.01251	0.9348	0.1381273	0.046726	0.7444	0.11099642	0.051272	0.70089	0.11689002	0.0340865	0.85465
P05201	14718	0.0807294	0.005942	0.3506	0.0819758	0.008474	0.2366	0.05789651	0.005218	0.2624	0.06471938	0.0040925	0.45464
Q9Z2I0	56384	0.0339909	0.002513	0.8883	0.035493	0.00355	0.8547	0.03152035	0.002089	0.90467	0.02553798	0.0024113	0.8423
P50446	16687	0.0619008	0.080952	0.0401	0.1459605	0.044137	0.4768	0.1675976	0.141066	0.09795	0.15296176	0.1232067	0.16154
P18826	18679	0.0076425	0.004065	0.2275	0.0017682	0.002511	0.0431	0.00152761	0.000732	0.25107	0.00209914	0.0016164	0.12322
Q76MZ3	51792	0.175555	0.02627	0.411	0.19816	0.03305	0.3997	0.08797974	0.006377	0.74542	0.11215338	0.009106	0.73747
Q9Z2Z6	57279	0.0376911	0.001849	0.8612	0.0422372	0.003915	0.6752	0.02656366	0.001474	0.82889	0.03280237	0.0028935	0.69275
O88799	NA	0.1979854	0.001713	0.999	0.1928213	0.009753	0.9751	0.17142696	0.006693	0.98204	0.22511269	0.0123979	0.97921
P07759	NA	0.7341599	0.108573	0.6751	0.5120663	0.154713	0.4064	0.72326485	0.078319	0.76636	0.74879909	0.1482798	0.58622
Q6IFX2	68239	0.0286621	0.009706	0.4422	0.0772074	0.020036	0.6796	0.05868697	0.009448	0.84645	0.61084232	0.2095288	0.67998
P35441	NA	0.5076356	0.071509	0.5701	0.3575897	0.079243	0.4696	0.61553516	0.088351	0.57416	0.66940937	0.0888485	0.70284
Q61147	12870	0.5995266	0.051583	0.558	0.7962603	0.042608	0.7333	0.68399195	0.033119	0.74892	0.78039946	0.0444773	0.70633
Q60864	20867	0.0937144	0.010966	0.8589	0.1173443	0.014862	0.8618	0.08254921	0.008715	0.91813	0.12051624	0.0056675	0.98906
P62075	30055	0.2337423	0.06502	0.3597	0.0791159	0.010048	0.7294	0.21114236	0.098105	0.16178	0.08073948	0.016284	0.56406
Q8BVI4	110391	0.0875527	0.017639	0.266	0.0568594	0.00404	0.7735	0.04601888	0.003112	0.76545	0.05486314	0.0031297	0.83893
P61148	14164	0.2193064	0.01581	0.9322	0.218842	0.018686	0.9195	0.19823064	0.008166	0.98004	0.19188818	0.018975	0.89498
Q64727	22330	0.064931	0.010082	0.2059	0.0613901	0.01008	0.206	0.06765588	0.010726	0.19816	0.05088531	0.0033738	0.62412
Q8QZT1	110446	0.0318176	0.00046	0.9671	0.0301476	0.000557	0.9534	0.02426857	0.000506	0.93266	0.02484827	0.0004709	0.95116
Q924X2	12895	0.104003	0.015781	0.1885	0.0780174	0.010486	0.2607	0.05092197	0.010072	0.11913	0.05761211	0.0111603	0.13551
P50752	21956	0.1227755	0.002749	0.934	0.1112588	0.002787	0.9316	0.1320654	0.00789	0.67158	0.10063937	0.0027265	0.9203
O54724	19285	0.0556715	0.003184	0.9592	0.0486834	0.002	0.9801	0.03139386	0.001207	0.97971	0.04469107	0.0009841	0.99422
Q03265	11946	0.0410386	0.002734	0.3519	0.0372403	0.002397	0.4021	0.03024727	0.000619	0.84994	0.03154476	0.0011687	0.66378
P45952	11364	0.0758896	0.015384	0.1734	0.04252	0.012578	0.1035	0.03938117	0.010581	0.10506	0.03580114	0.0027233	0.63116
P03976	NA	0.0490138	0.015455	0.4362	0.1551467	0.121595	0.1289	0.12804177	0.141995	0.06346	0.11445422	0.1306431	0.08754
Q99J99	NA	0.126636	0.003865	0.9926	0.1109379	0.003009	0.9942	0.07425221	0.004648	0.95868	0.09580636	0.00304	0.99201
Q6ZQ20	NA	0.096965	0.113946	0.0492	0.1203142	0.106039	0.0969	0.37184242	0.166424	0.27746	0.1057017	0.1455946	0.04207
P62814	11966	0.2830626	0.039251	0.9123	0.414335	0.1348	0.6116	0.35159317	0.11517	0.5381	0.2656234	0.1137875	0.5215
Q8BWF0	214579	0.1365286	0.048339	0.0897	0.0802679	0.03674	0.063	0.09933223	0.032887	0.10353	0.10171083	0.0398232	0.08415
P12367	NA	0.0141972	0.005442	0.3271	0.0169844	0.006965	0.3313	0.01446702	0.004458	0.44756	0.09778166	0.038563	0.34887
Q8R0F8	NA	0.0476594	0.002238	0.9805	0.0489505	0.001276	0.9919	0.03195353	0.001365	0.97508	0.04064348	0.0021658	0.96971

P21614	14473	0.5491652	0.070914	0.5404	0.641498	0.077358	0.6153	0.45461829	0.049627	0.59977	0.688261	0.0736349	0.63601
Q9WV35	11811	0.0715446	0.006096	0.9138	0.0769437	0.005568	0.9409	0.05884699	0.003647	0.94897	0.06877714	0.0037791	0.96786
Q8R2K3	NA	0.0369587	0.002053	0.9586	0.0356478	0.001267	0.9851	0.03007711	0.004049	0.79759	0.02678893	0.0014886	0.96427
Q8VEK3	51810	0.424466	0.087503	0.6441	0.415448	0.089328	0.6432	0.12945327	0.008926	0.94179	0.14407305	0.0088869	0.95983
Q8BW75	109731	0.1029373	0.050015	0.1748	0.0424825	0.020945	0.186	0.1284106	0.066156	0.14621	0.02498401	0.0060244	0.47512
Q08284	NA	0.6257705	0.064223	0.5687	0.6495402	0.06834	0.5969	0.5703886	0.057282	0.57596	0.63039508	0.0731555	0.54497
Q60597	18293	0.1337617	0.008859	0.3128	0.1057904	0.007712	0.3069	0.09226715	0.007923	0.21676	0.09968737	0.0078056	0.28214
Q99N96	94061	0.0839321	0.004942	0.9697	0.0676284	0.008546	0.8867	0.03944625	0.029107	0.16948	0.06548986	0.0066204	0.89895
A0JLR7	NA	0.4074149	0.090436	0.4384	0.464531	0.080993	0.5682	0.38232526	0.083808	0.4178	0.29141772	0.0684226	0.42049
P07901	15519	0.3230267	0.048196	0.4783	0.3514982	0.057211	0.4562	0.36388161	0.058865	0.48857	0.26874072	0.040413	0.48477
Q91Z53	76238	0.0481089	0.002702	0.8299	0.0540776	0.001818	0.9405	0.04042196	0.000696	0.98111	0.04375828	0.0014549	0.94073
P09103	18453	0.4048539	0.034737	0.591	0.3783628	0.038838	0.5335	0.22605673	0.0248	0.46919	0.19332745	0.0242902	0.40517
Q3TS19	NA	0.0207989	0.001049	0.9704	0.0240957	0.00173	0.9604	0.01558083	0.001157	0.93789	0.0268556	0.0013123	0.98356
P06537	NA												
P70670	17938	0.3415725	0.020085	0.9864	0.331654	0.023158	0.9762	0.22435036	0.006622	0.99653	0.26821034	0.0181129	0.98208
Q9JKB1	50933	0.6282899	0.147127	0.5657	0.5959028	0.129727	0.6573	0.78854308	0.163107	0.62539	0.69693023	0.1413709	0.66945
Q9JKS4	24131	0.1714483	0.035193	0.2124	0.1746181	0.039354	0.2101	0.12829791	0.03304	0.14487	0.14818621	0.0351226	0.20508
P56382	67126	0.0303976	0.001238	0.9541	0.0296697	0.001026	0.971	0.02383624	0.001449	0.90319	0.02189734	0.0009386	0.95609
P15626	14863	0.0487869	0.003497	0.8403	0.0428589	0.003767	0.792	0.03350266	0.002825	0.77423	0.04291414	0.0038996	0.76597
Q9D0F9	72157	0.0945413	0.010742	0.2833	0.0874862	0.013141	0.203	0.07659388	0.013096	0.14732	0.0829771	0.0170313	0.1251
Q9CZB0	66052	0.0316284	0.002075	0.8891	0.0337	0.001379	0.9614	0.02631468	0.000895	0.96755	0.02762725	0.0015092	0.93058
P03888	NA	0.2446864	0.058361	0.2209	0.1530533	0.05065	0.1687	0.14573241	0.052709	0.12401	0.17208364	0.0526756	0.18505
Q8C0M9	66514	0.0092083	0.003227	0.3677	0.0186789	0.004926	0.5451	0.01497562	0.004053	0.49375	0.02425682	0.0037358	0.77843
Q8CG76	110198	0.0620467	0.008179	0.5723	0.0572974	0.005575	0.7354	0.03708587	0.001327	0.94781	0.03972821	0.0021324	0.90132
Q9Z1J3	NA	0.078382	0.003825	0.9677	0.0766832	0.004628	0.9581	0.05102139	0.002648	0.96367	0.06984568	0.0095755	0.82867
Q91WK5	NA	0.1812974	0.1002	0.2904	0.414906	0.200017	0.3497	0.39283281	0.15869	0.37996	0.73717357	0.1563453	0.6136
Q64105	NA	0.0588245	0.002779	0.8534	0.0627015	0.002474	0.9043	0.042974	0.002517	0.78679	0.07095349	0.0180646	0.18946
Q6P8J7	76722	0.0820721	0.015572	0.1168	0.0516533	0.013348	0.0764	0.03802767	0.005192	0.19751	0.05907741	0.0132077	0.09712
Q99NB1	68738	0.0654835	0.005147	0.3882	0.057597	0.005637	0.3169	0.05991944	0.009257	0.14917	0.08324183	0.0142596	0.13519
Q6X7S9	386655	0.1004403	0.005963	0.953	0.1226439	0.005699	0.9747	0.09726699	0.008042	0.91266	0.08355319	0.0063122	0.94093
Q8CGK3	74142	0.0370914	0.001748	0.8946	0.0377044	0.001325	0.9418	0.02945886	0.001309	0.90046	0.03841204	0.0116354	0.18195
Q9CPQ3	223696	0.0430339	0.005118	0.8549	0.0283707	0.005976	0.7146	0.02800779	0.003648	0.80805	0.03087459	0.0055772	0.71861
Q8BG32	69077	0.1952623	0.011504	0.96	0.2197099	0.006125	0.9931	0.15001251	0.004834	0.98871	0.166459	0.0040639	0.99407
Q60605	433688	0.6364987	0.072475	0.5504	0.7304131	0.093665	0.5802	0.48755419	0.065599	0.47935	0.62725655	0.0711249	0.60396
P03893	NA	0.0206516	0.001269	0.9498	0.0224697	0.001851	0.9305	0.03270793	0.015945	0.23109	0.01857225	0.0016054	0.91772
Q62048	18611	0.7202819	0.079095	0.8645	0.7769881	0.122066	0.7715	0.50058786	0.099158	0.66222	0.85853441	0.0596713	0.94954
Q9D2J7	319196	0.0282577	0.019698	0.2554	0.0148935	0.009173	0.3052	0.02578122	0.021131	0.19877	0.10239196	0.0712584	0.40767
Q9D892	16434	0.0928632	0.001939	0.9952	0.0919201	0.003173	0.9871	0.06417191	0.002362	0.98269	0.0841761	0.0026656	0.99007
Q9D6Y9	74185	0.0670939	0.009079	0.7845	0.1004185	0.003499	0.9833	0.05458723	0.00849	0.76077	0.08866146	0.0058417	0.94271
P11499	15516	0.3291304	0.025545	0.5589	0.3543443	0.029727	0.5505	0.25561404	0.02458	0.45033	0.21535054	0.0169747	0.57906
P63101	22631	0.1052968	0.015355	0.3918	0.0956353	0.003047	0.939	0.07419817	0.002617	0.91778	0.08691365	0.0032198	0.91926
Q04857	12833	0.0500809	0.018297	0.3486	0.0289865	0.00722	0.5732	0.07095139	0.09659	0.03711	0.02597667	0.0117249	0.2903

P70195	19177	0.1045979	0.011579	0.8812	0.1411222	0.011155	0.9412	0.22954937	0.080577	0.42456	0.11537226	0.0189131	0.80524
P03987	NA	0.1772521	0.043008	0.2393	0.2472087	0.056622	0.34	0.22572745	0.048525	0.34003	0.23083119	0.0654225	0.26801
Q99L47	70356	0.2235218	0.025004	0.8601	0.3385017	0.065239	0.6917	0.28588413	0.102345	0.35788	0.20328364	0.0174936	0.91839
Q7TQ48	106393	0.0244756	0.001028	0.8065	0.0247187	0.001358	0.7357	0.01519572	0.000898	0.66717	0.02007769	0.0017353	0.52939
P97450	11957	0.0321861	0.000508	0.9928	0.0309387	0.00049	0.9938	0.02083988	0.000614	0.97546	0.02490671	0.0005045	0.98985
P11438	16783	0.677215	0.095866	0.8194	0.7810829	0.092803	0.8656	0.71002153	0.08789	0.82337	0.88029769	0.0808301	0.90812
P51885	17022	0.0574907	0.011176	0.3373	0.0400124	0.004389	0.589	0.02498926	0.003387	0.4757	0.04885388	0.0055137	0.57936
P06801	17436	0.1226806	0.041617	0.2368	0.119598	0.005486	0.95	0.16705787	0.040461	0.37021	0.08982174	0.004207	0.94801
Q8R4N0	69634	0.028842	0.001797	0.6352	0.0277444	0.002339	0.516	0.0195429	0.001064	0.68675	0.02236284	0.0010707	0.77315
Q9EPB5	68607	0.0737897	0.013989	0.6987	0.0637142	0.105535	0.0295	0.06124394	0.026583	0.2749	0.23022698	0.0877174	0.3647
Q61554	NA	0.0487013	0.029764	0.1433	0.0210642	0.004616	0.5364	0.0487047	0.007208	0.71724	0.02959902	0.0075409	0.47541
O70370	13040	0.5392045	0.174336	0.5152	0.6078559	0.15828	0.5514	0.64649984	0.120718	0.70502	0.6544629	0.2374165	0.35182
Q99JY0	1.01E+08	0.0773596	0.005765	0.3322	0.072909	0.004505	0.4572	0.05117109	0.005952	0.16686	0.06624704	0.0066034	0.2387
Q8CI04	NA	0.0332356	0.011392	0.6803	0.0474994	0.015661	0.6969	0.06332417	0.025901	0.74929	0.03556841	0.0035054	0.97169
Q924M7	110119	0.0726983	0.003444	0.8576	0.1149886	0.024707	0.2416	0.05434601	0.004156	0.70086	0.06091043	0.0210642	0.12413
Q9D6Y7	110265	0.0649478	0.012753	0.6336	0.1033499	0.091231	0.0898	0.03147606	0.073629	0.01204	0.17023951	0.0720844	0.24704
Q8BFP9	228026	0.0103908	0.00491	0.2562	0.0141911	0.005985	0.3382	0.02326636	0.005799	0.53485	0.00857357	0.006314	0.15568
P28271	11428	0.0814812	0.005232	0.8965	0.0974781	0.015122	0.6244	0.06622651	0.00569	0.82366	0.07406806	0.0077081	0.7937
Q9JHW2	52633	0.0387388	0.001408	0.9154	0.0681594	0.019215	0.1643	0.02873372	0.00125	0.87711	0.03272868	0.0132495	0.0883
ENSMUSP0(	NA	0.025159	0.003357	0.8005	0.0217857	0.00876	0.3401	0.01935128	0.015834	0.0964	0.01835492	0.0027146	0.79209
P00493	15452	0.4535179	0.107013	0.6423	0.1546598	0.015111	0.905	0.1280951	0.011896	0.89919	0.16590421	0.0299046	0.77374
Q9DCS3	26922	0.0274274	0.002309	0.9216	0.0307619	0.001075	0.9867	0.01949473	0.0009	0.97711	0.02499775	0.0013276	0.97793
Q8R0N6	76187	0.1863113	0.058951	0.3331	0.095053	0.004968	0.9507	0.06087502	0.002048	0.97143	0.0743273	0.0057811	0.89207
Q921I1	22041	0.7738428	0.027771	0.6999	0.7957733	0.026972	0.7337	0.72908645	0.020709	0.77252	0.77684715	0.0216445	0.79069
Q8C156	215387	0.3244766	0.090605	0.4966	0.1340788	0.026891	0.6744	0.48799114	0.170682	0.38605	0.81581098	0.2148475	0.56724
Q61085	14787	0.0160005	0.000687	0.9801	0.0160478	0.001254	0.937	0.0122165	0.000647	0.96216	0.01240579	0.0011725	0.90318
P48962	11739	0.0294461	0.001165	0.8519	0.0327202	0.003463	0.4742	0.02137193	0.000921	0.8303	0.02346763	0.0017382	0.65504
P01837	NA	0.1747971	0.036875	0.3485	0.3193411	0.048776	0.5301	0.26068746	0.056406	0.39293	0.41187739	0.0713787	0.51786
P17182	1.01E+08	0.0382267	0.002155	0.7834	0.0445552	0.002307	0.818	0.03183566	0.00188	0.75712	0.03658539	0.0019417	0.805
P45376	11677	0.0703992	0.002836	0.7749	0.0647872	0.002404	0.8241	0.04877903	0.001699	0.82165	0.05320071	0.0017294	0.85927
B2RY56	67039	0.0004544	5.28E-05	0.8408	0.0004051	4.97E-05	0.8472	0.00027199	3.90E-05	0.78901	0.0004493	3.77E-05	0.92227
Q8BFR5	233870	0.038773	0.001785	0.7612	0.0329309	0.00209	0.6563	0.03542679	0.011527	0.05815	0.02863083	0.0019764	0.62298
Q9R0H2	59308	0.0265866	0.017105	0.2566	0.0237173	0.011615	0.3733	0.02216055	0.007675	0.58153	0.01691398	0.0072439	0.40529
Q9CZS1	72535	0.2156158	0.040623	0.2653	0.114812	0.027136	0.206	0.07635861	0.014301	0.2468	0.12692113	0.0321343	0.16847
Q9DCM0	66071	0.0334567	0.025777	0.252	0.0336338	0.00468	0.9281	0.01750271	0.011959	0.2343	0.03041482	0.0113869	0.54318
P47955	665298	0.2326953	0.036908	0.5955	0.1667422	0.005489	0.9767	0.15600869	0.041486	0.34372	0.13306193	0.0023189	0.99516
P63158	637733	0.0693827	0.002327	0.9845	0.079772	0.002128	0.9915	0.05609517	0.002873	0.96458	0.07411358	0.0016301	0.99423
Q8K3J1	225887	0.0238631	0.002683	0.8496	0.0225212	0.002842	0.8396	0.0202901	0.003589	0.69538	0.01541712	0.0025977	0.74589
Q64669	18104	0.0475931	0.00223	0.9119	0.0459925	0.002348	0.9121	0.03508539	0.001837	0.89239	0.03799013	0.0023926	0.87202
Q3ULJ0	333433	0.0990543	0.004731	0.9164	0.1595619	0.03527	0.3828	0.06002887	0.004446	0.82374	0.09206102	0.006751	0.8416
Q9CZU6	12974	0.0388575	0.008728	0.0882	0.0417346	0.009814	0.0922	0.02270308	0.006628	0.05388	0.04604793	0.0148708	0.05031
Q8CGW4	214105	0.0032477	0.000414	0.8149	0.0039977	0.000664	0.7513	0.00460385	0.001116	0.56685	0.00341135	0.0005978	0.73075

Q9QVP4	17898	0.3397777	0.092519	0.3032	0.3321674	0.075844	0.3676	0.28279677	0.088995	0.25827	0.21822338	0.0908142	0.18763
P21107	59069	0.0927611	0.007123	0.9658	0.0819975	0.003419	0.9948	0.0410681	0.00134	0.99365	0.05170278	0.0028113	0.99121
Q8CAQ8	76614	0.0357083	0.006809	0.095	0.04282	0.005968	0.1848	0.04365304	0.008018	0.10163	0.04165778	0.007682	0.1138
P54116	13830	0.160416	0.028521	0.725	0.1032088	0.004001	0.9837	0.07733637	0.00431	0.96407	0.08778866	0.0018451	0.9956
O89053	12721	0.5279523	0.262712	0.3659	1.4843718	0.277105	0.8516	0.34208763	0.158137	0.43818	0.68512004	0.3379842	0.40647
P97372	19188	0.0865973	0.010861	0.876	0.1065584	0.00469	0.9791	0.07464651	0.010812	0.87194	0.09240232	0.0103813	0.90828
Q8K480	259172	0.0275457	0.002967	0.915	0.034863	0.014914	0.4766	0.0263315	0.002495	0.93299	0.0180113	0.0051964	0.66692
P07724	11657	0.493564	0.010793	0.7208	0.5161374	0.012592	0.7003	0.42415515	0.010933	0.64264	0.51687281	0.0126196	0.69162
O35841	11800	0.0308613	0.009284	0.5011	0.0459037	0.01508	0.5367	0.0284011	0.00874	0.51362	0.02745112	0.0134276	0.41058
Q9D6X6	76453	0.0112217	0.001288	0.9382	0.0116781	0.001035	0.9408	0.00787433	0.000853	0.92407	0.00879936	0.001522	0.91764
Q9WVH9	23876	0.2280271	0.062889	0.4843	0.2371659	0.091996	0.3766	0.10215564	0.014297	0.79706	0.23863816	0.076636	0.44691
Q6LCR2	NA	0.0269798	0.000925	0.9849	0.0244479	0.000809	0.9892	0.0185126	0.001251	0.93995	0.01944101	0.0007819	0.98096
P58774	22004	0.1011034	0.020641	0.5217	0.0527738	0.009307	0.6165	0.10296981	0.063756	0.12657	0.10162086	0.0619363	0.1301
Q3TD78	NA	0.0592424	0.020744	0.2705	0.026141	0.003255	0.7456	0.02203656	0.002562	0.76286	0.02252034	0.0047452	0.5175
Q9CQX8	66128	0.0327784	0.001401	0.9256	0.031723	0.001897	0.8831	0.02587379	0.001594	0.85692	0.02417498	0.0013526	0.89619
Q93092	21351	0.5064891	0.097364	0.51	0.509628	0.09257	0.548	0.29244363	0.075513	0.37497	0.27878635	0.0854258	0.30737
Q05920	NA	0.1567739	0.034048	0.2204	0.1532333	0.044768	0.1399	0.10731387	0.028249	0.14965	0.08734906	0.0263848	0.13211
Q02566	17888	0.0817352	0.007391	0.2855	0.0939433	0.012804	0.1743	0.06601711	0.007687	0.20849	0.07407797	0.0132195	0.11656
Q9DCD0	110208	0.0689375	0.006654	0.7704	0.0681093	0.004223	0.9155	0.05344723	0.003671	0.88332	0.05348854	0.0041894	0.87166
Q8BGY7	108654	0.0397031	0.005152	0.8204	0.0440561	0.006217	0.8203	0.0376918	0.00818	0.60263	0.08668967	0.0169371	0.68584
Q3TP92	67181	0.0282448	0.003114	0.9427	0.0416197	0.002304	0.9939	0.0135132	0.004078	0.61067	0.02168147	0.0067411	0.77519
Q8CC88	219189	0.0495072	0.001265	0.948	0.0463867	0.001454	0.9365	0.03346641	0.001774	0.81266	0.04528467	0.0042457	0.62589
P48787	21954	0.1851662	0.015133	0.6692	0.1353137	0.003279	0.9638	0.11365095	0.002773	0.96109	0.11654359	0.0022867	0.97595
Q9R0P5	56431	0.1757795	0.040053	0.2065	0.2290657	0.040098	0.3243	0.18437777	0.044938	0.18532	0.1903935	0.0348216	0.32532
P52850	17423	0.01917	0.007151	0.3392	0.0077934	0.00382	0.2575	0.01377748	0.004914	0.35963	0.00844819	0.0032459	0.36082
P08003	12304	0.4953269	0.098793	0.4916	0.5370534	0.113781	0.5397	0.28571239	0.072255	0.39449	0.46814919	0.1152001	0.46501
P61971	668830	0.1264414	0.035925	0.3067	0.1334683	0.073288	0.1171	0.18825916	0.072691	0.18784	0.18397846	0.076376	0.18838
Q3TMY3	NA	0.0790944	0.012042	0.755	0.1928205	0.085893	0.2958	0.06453375	0.0059	0.89525	0.08769238	0.0083695	0.90146
Q91V64	66307	0.0862851	0.003519	0.982	0.1157213	0.009307	0.9392	0.07274773	0.006218	0.93192	0.08164839	0.0042324	0.97896
P14069	20200	0.1359106	0.005562	0.9676	0.1428325	0.009324	0.9325	0.09257189	0.004855	0.93565	0.13547271	0.0056012	0.96376
Q3UED7	NA	0.0440578	0.005571	0.8742	0.0250786	0.003895	0.8216	0.02971519	0.004199	0.80667	0.03264019	0.0062905	0.74947
Q9CQ91	66091	0.0344061	0.000749	0.9934	0.0335056	0.001203	0.9848	0.02379365	0.001185	0.96646	0.02991443	0.000636	0.99461
Q9WVL0	14874	0.0449145	0.005374	0.7212	0.0384032	0.002052	0.9359	0.10755415	0.043885	0.18198	0.03132815	0.0012107	0.96401
P11087	12842	0.0256973	0.008906	0.3729	0.1674424	0.105275	0.1741	0.17794846	0.071498	0.30674	0.04027012	0.0193893	0.28169
Q9JI75	18105	0.0763187	0.004374	0.9651	0.0908047	0.115602	0.0581	0.23478826	0.125828	0.22489	0.07272465	0.0145709	0.75692
Q8R2Q4	320806	0.1220209	0.021949	0.7555	0.1462614	0.005975	0.982	0.09866147	0.007394	0.94181	0.1279587	0.0150622	0.86774
P16546	20740	0.0553597	0.001563	0.9897	0.0435176	0.006542	0.7866	0.04277443	0.003207	0.92705	0.05545004	0.0029544	0.9724
Q61646	15439	0.180473	0.089113	0.1203	0.3056689	0.107834	0.2007	0.25859757	0.08145	0.20965	0.45789519	0.1194971	0.31453
Q9CQ69	22272	0.0229845	0.000667	0.9762	0.0203923	0.000709	0.9707	0.01595602	0.000611	0.95919	0.01645532	0.0006044	0.96738
Q9JJZ2	53857	0.0106466	0.001431	0.7981	0.0088857	0.000768	0.9178	0.00573897	0.000358	0.94833	0.00645722	0.0007713	0.85382
Q8VDN2	11928	0.0461317	0.003424	0.7841	0.0825733	0.027558	0.1761	0.03636362	0.001649	0.90504	0.04619764	0.0027456	0.86815
Q78ZA7	17955	0.5177003	0.112659	0.4389	0.4944795	0.102274	0.4832	0.50825689	0.102343	0.47739	0.3236001	0.0727604	0.48504

Q3TLK7	NA	0.0022804	0.001355	0.2613	0.0051892	0.00268	0.3191	0.00693205	0.002835	0.39914	0.00444351	0.0014907	0.55934
P35979	668706	1.036402	0.252129	0.6787	0.8731647	0.321808	0.4792	0.75742349	0.121203	0.82998	0.70221555	0.0710792	0.93308
P18572	12215	0.0962308	0.008921	0.8005	0.099137	0.012264	0.7233	0.07270501	0.005343	0.86865	0.08624105	0.0082833	0.81873
P59017	94044	0.0451675	0.004942	0.6988	0.0496078	0.004764	0.8007	0.08997934	0.031204	0.18763	0.04813195	0.0066065	0.60955
XP_0014739	NA	0.3561145	0.157676	0.2818	0.4328469	0.178167	0.3492	0.38174026	0.189242	0.23839	0.3876024	0.1605292	0.32698
Q60977	NA	0.0168565	0.007609	0.4499	0.0257605	0.012726	0.3692	0.01853863	0.003256	0.76423	0.01981379	0.0058411	0.65727
Q8BGK2	234072	0.1277609	0.008905	0.7861	0.1195343	0.002933	0.9696	0.08223401	0.004493	0.87928	0.10587714	0.0035919	0.94047
P43024	12861	0.0333222	0.001647	0.9692	0.0357191	0.001609	0.9762	0.0281008	0.001215	0.97449	0.0287286	0.0017674	0.95655
Q4PLS0	NA	0.030055	0.025656	0.5785	0.0128863	0.010215	0.3466	0.04175022	0.054759	0.36761	0.02520505	0.0101842	0.75385
P68040	14694	0.3278654	0.08649	0.5065	0.2490552	0.07781	0.4606	0.08890051	0.012006	0.79659	0.09341318	0.0235021	0.56831
P19783	12857	0.0276606	0.001539	0.8412	0.0255969	0.002309	0.7027	0.01943426	0.000857	0.89547	0.02179023	0.0009297	0.91506
P24452	NA	0.1010728	0.067164	0.0774	0.2022106	0.081056	0.1993	0.09402314	0.060875	0.07601	0.2157681	0.0628276	0.32055
Q6PGF3	216154	0.0001369	4.61E-05	0.3865	0.0002909	9.22E-05	0.4533	0.00023257	7.86E-05	0.38461	0.00039242	9.74E-05	0.57476
Q3V2M1	NA	0.0209117	0.003535	0.7143	0.0262058	0.00597	0.6162	0.00878802	0.002138	0.54674	0.02502966	0.0064455	0.55686
Q9D1G3	74770	0.028524	0.004395	0.7642	0.0396499	0.009105	0.5933	0.02560821	0.004362	0.74174	0.03990112	0.0054946	0.76722
P15327	12183	0.0395185	0.048351	0.0271	0.0220984	0.004388	0.5717	0.06695448	0.048871	0.07253	0.02026244	0.0034964	0.61527
Q9D8U6	69189	0.0147086	0.003641	0.5383	0.0190612	0.009707	0.2432	0.01338787	0.004195	0.42113	0.01021219	0.006261	0.18147
Q8VCM7	99571	0.9172931	0.051231	0.9016	0.9471596	0.047175	0.9222	0.64447518	0.060035	0.77739	0.9393989	0.0514349	0.90259
P70296	23980	0.1364262	0.021232	0.2592	0.1081171	0.017894	0.2617	0.08752298	0.017934	0.16794	0.07328643	0.0127641	0.24246
P23492	NA	0.0516828	0.003888	0.8426	0.1003958	0.055525	0.0954	0.0990964	0.05209	0.10161	0.11235869	0.074349	0.06662
P10639	22166	0.1322338	0.044659	0.1694	0.0557431	0.004088	0.834	0.08773844	0.040709	0.09959	0.09122939	0.0300568	0.19513
P08249	17448	0.0239196	0.000435	0.9309	0.0226209	0.000692	0.8465	0.01757322	0.000361	0.9142	0.02998339	0.0069714	0.08705
Q91ZA3	110821	0.0432067	0.007173	0.1867	0.0430652	0.009484	0.1268	0.02402905	0.001741	0.5434	0.02633189	0.0026959	0.3985
P47754	12343	0.0951637	0.004378	0.9712	0.0899781	0.002824	0.9883	0.07007982	0.002792	0.97827	0.07922937	0.0034112	0.97824
Q71RI9	229905	0.0500394	0.016719	0.0924	0.0214787	0.000884	0.8846	0.01682241	0.000709	0.86347	0.01646686	0.0008602	0.82635
P54822	11564	0.11163	0.006288	0.9517	0.1253556	0.017725	0.7576	0.09245999	0.012588	0.7604	0.0853575	0.0094087	0.84584
O08553	12934	0.0905408	0.018457	0.2454	0.0785808	0.004872	0.7856	0.06711287	0.01555	0.19277	0.10672123	0.0266046	0.18476
Q8BGQ7	234734	0.226939	0.02976	0.8409	0.2641321	0.028052	0.9466	0.17652501	0.034557	0.89688	0.22203909	0.0176214	0.96359
P16858	1.01E+08	0.0911951	0.006343	0.3955	0.0923415	0.006194	0.4416	0.07511121	0.006935	0.27009	0.09047199	0.0091217	0.25999
P97823	18777	0.0495932	0.003613	0.8177	0.0498967	0.003059	0.8779	0.04209255	0.003183	0.79899	0.04823071	0.0029922	0.87535
P01680	NA	0.1035448	0.053972	0.269	0.057127	0.023659	0.4928	0.08333012	0.025498	0.5427	0.27812602	0.1323008	0.35584
Q9D1I5	73724	0.0638167	0.004864	0.9451	0.0530199	0.005718	0.8866	0.03915161	0.003877	0.8947	0.05565965	0.0044172	0.94075
Q9Z2U2	30046	0.0039897	0.002157	0.1964	0.0038657	0.001877	0.2783	0.00142015	0.000682	0.23649	0.00290912	0.0007805	0.53656
Q06890	12759	0.5408135	0.124399	0.5745	0.6773904	0.178938	0.5657	0.31657363	0.077532	0.54356	0.13260337	0.0496443	0.37286
O88342	22388	0.1553983	0.019862	0.3869	0.1444827	0.024681	0.2803	0.09346716	0.018223	0.21508	0.09260504	0.0060572	0.73563
Q06986	20439	0.0096601	0.005388	0.1983	0.0057184	0.002455	0.3304	0.00563992	0.001588	0.47392	0.00864954	0.0022336	0.57686
Q7TMF3	66414	0.034977	0.001969	0.9158	0.0324418	0.002216	0.8955	0.02539153	0.001541	0.90349	0.02306557	0.0019497	0.84844
P21981	21817	0.1879256	0.027731	0.289	0.107579	0.007247	0.6551	0.09694787	0.017381	0.20321	0.16639248	0.027456	0.27671
O55126	NA	0.0297428	0.002679	0.4322	0.0449944	0.008986	0.151	0.02253083	0.002478	0.33653	0.0238605	0.0020516	0.48961
P16015	12350	0.3211319	0.060831	0.5946	0.3565283	0.080299	0.4726	0.13008441	0.00779	0.92687	0.2909247	0.0826969	0.38226
Q8R5L1	NA	0.0300803	0.000633	0.9938	0.0290544	0.000869	0.9894	0.02197961	0.000861	0.97895	0.0237257	0.0007933	0.98676
Q8BMS4	230027	0.0454852	0.001558	0.9682	0.0434729	0.001704	0.9702	0.03069076	0.001309	0.95153	0.04030142	0.0016815	0.95989

Q61233	18826	0.2106301	0.048108	0.2897	0.1681134	0.036401	0.299	0.1129676	0.039326	0.13926	0.19880577	0.0381944	0.36079
O35639	11745	0.0780668	0.001313	0.9961	0.0877519	0.003072	0.9855	0.05418643	0.004831	0.89985	0.08510932	0.002175	0.99222
Q9CQM9	30926	0.2966219	0.061679	0.6229	0.3151154	0.067292	0.6463	0.25710994	0.063986	0.5356	0.29270487	0.0753848	0.55681
P46412	NA	0.990101	0.079048	0.844	1.0056366	0.065882	0.9031	0.72100538	0.056623	0.85274	0.81962936	0.0569466	0.89231
Q9D855	NA	0.063252	0.023961	0.1107	0.0649948	0.023771	0.1348	0.02074403	0.001138	0.8513	0.04783912	0.026735	0.06134
Q923D2	233016	0.071323	0.022824	0.1324	0.1224941	0.040747	0.1481	0.08935652	0.022928	0.19426	0.11344701	0.0344163	0.16751
P14211	12317	0.446371	0.043008	0.6385	0.4121686	0.043806	0.5921	0.26457792	0.031148	0.50052	0.28135998	0.0311292	0.60205
A2AN99	NA	0.0561425	0.002127	0.9803	0.0622581	0.002922	0.9743	0.04035279	0.000994	0.99158	0.04637567	0.0024612	0.96731
Q99N87	77721	0.0449841	0.002888	0.9566	0.044901	0.004052	0.9178	0.03852145	0.002251	0.95436	0.03371884	0.0022002	0.95526
Q9R069	57278	0.5708083	0.273279	0.421	#VALUE!	NA	NA	#VALUE!	NA	NA	0.54666393	0.1826014	0.599
P13707	14555	0.2457641	0.05014	0.3695	0.2286174	0.063069	0.2427	0.1082308	0.026562	0.27855	0.15514441	0.0544856	0.17974
Q64433	15528	0.0818488	0.016453	0.1937	0.0479506	0.014833	0.1072	0.03134001	0.003517	0.44755	0.03501673	0.0057068	0.30204
Q11011	19155	0.2185805	0.045942	0.2706	0.1347526	0.020849	0.4105	0.07600133	0.008164	0.57144	0.11941011	0.0216669	0.33241
P97467	18484	0.3505435	0.096449	0.4101	0.3267584	0.136077	0.2426	0.24101539	0.108393	0.2479	0.19618665	0.1334602	0.21267
O70181	NA	0.1505027	0.018328	0.7625	0.1099873	0.022821	0.5774	0.16044833	0.050456	0.32503	0.122036	0.0080557	0.93482
Q4KML4	NA	0.1894523	0.073449	0.1977	0.3637685	0.107092	0.3341	0.25598741	0.101069	0.1864	0.34717134	0.1147428	0.26803
P05064	353204	0.1729486	0.013366	0.4245	0.1324507	0.00616	0.6796	0.11723537	0.008548	0.44042	0.14916435	0.0116187	0.43973
Q91XE4	71670	0.0851166	0.101452	0.0554	0.3069144	0.142588	0.3667	0.22867391	0.121039	0.28397	0.07050564	0.1285415	0.03624
P85094	664994	0.0621589	0.014808	0.1843	0.0464644	0.01678	0.0962	0.02766205	0.002105	0.69156	0.03469909	0.0021923	0.77917
P29788	22370	0.0170998	0.00104	0.9508	0.0152443	0.0011	0.9412	0.00893955	0.000801	0.89886	0.02557428	0.0112542	0.30086
Q8BS95	67549	0.150516	0.092987	0.1576	0.0042728	0.00237	0.2131	0.11801584	0.128232	0.05705	0.05915456	0.0318976	0.25591
Q9R0P3	1E+08	0.0585577	0.009406	0.2193	0.0554424	0.002708	0.776	0.04273747	0.002498	0.67173	0.048158	0.0024805	0.75096
Q9DCS9	68342	0.0352387	0.000832	0.9682	0.036138	0.001189	0.9486	0.02692208	0.000924	0.93503	0.02985563	0.0009594	0.94997
	NA												
P10493	18073	0.0336032	0.001741	0.9638	0.0418809	0.015164	0.4095	0.02572604	0.011089	0.29281	0.23819404	0.1595648	0.15661
Q3TZ89	240667	0.0000433	1.12E-05	0.5184	0.0000333	2.22E-05	0.1706	0.0000319	1.15E-05	0.3701	0.0000278	1.49E-05	0.22445
P97352	20196	0.0121896	0.001853	0.8964	0.0138445	0.003483	0.798	0.01207202	0.00207	0.82933	0.02965457	0.0030704	0.95888
Q3TTY5	16681	0.0648487	0.01892	0.4563	0.1118841	0.11034	0.0789	0.11787887	0.017357	0.76715	0.11579154	0.0212045	0.71305
P14685	22123	0.046593	0.015841	0.5905	0.0569402	0.023268	0.545	0.03618323	0.002762	0.95016	0.03239789	0.0088388	0.65746
P29391	NA	0.2050148	0.041781	0.4146	0.2261269	0.03389	0.5743	0.13882753	0.005942	0.93014	0.22317626	0.0254873	0.67451
Q8BP40	66659	0.0358204	0.008444	0.5625	0.0254188	0.002614	0.8874	0.02141522	0.006662	0.42467	0.02644309	0.0143092	0.22154
Q9Z1P6	66416	0.0502347	0.005161	0.8257	0.0710614	0.012364	0.6602	0.03374997	0.002211	0.92092	0.0375681	0.0070075	0.62835
Q9Z2Y8	114863	0.0924265	0.002075	0.992	0.0861548	0.00208	0.9913	0.05843835	0.00182	0.98096	0.0785604	0.0019438	0.9915
Q9D7J4	66359	0.0719945	0.001956	0.9956	0.0634106	0.002971	0.987	0.05188341	0.012573	0.77304	0.05480788	0.0022584	0.99158
Q9DCZ4	68316	0.0212106	0.00127	0.8913	0.0270699	0.002622	0.7862	0.01568015	0.00093	0.88766	0.02014139	0.001471	0.85419
P01843	NA	0.2488875	0.094424	0.3483	0.4998543	0.210538	0.3605	0.72277437	0.241142	0.49955	0.40611131	0.2018905	0.31015
Q9D6J6	72900	0.0585683	0.004858	0.6718	0.0932921	0.022763	0.2159	0.03739497	0.002104	0.81232	0.04032762	0.0024406	0.81494
Q91YT2	193670	0.2837375	0.139788	0.2556	0.3252638	0.140339	0.3495	0.15008798	0.111504	0.13118	0.10716498	0.1037834	0.0816
Q9EQI8	67308	0.0505381	0.005966	0.8997	0.0703925	0.013877	0.8109	0.04097051	0.00308	0.96719	0.04062702	0.011041	0.77195
Q9Z0S1	23827	0.042692	0.002006	0.9784	0.0414236	0.003641	0.9487	0.03235723	0.002424	0.95193	0.03901522	0.0025336	0.96736
Q63918	20324	0.1064004	0.012548	0.7998	0.1309917	0.010988	0.8988	0.19125404	0.061448	0.33769	0.12609934	0.1024873	0.09167
A3KMP2	239570	0.1519153	0.016072	0.8645	0.1115744	0.004467	0.9811	0.08257543	0.007443	0.89789	0.11150157	0.0099527	0.91273

Q8K4Z3	246703	0.0786061	0.026681	0.168	0.037952	0.001481	0.9412	0.0770042	0.031159	0.11721	0.03199548	0.0011676	0.95063
P01872	NA	0.0693934	0.030643	0.4608	0.1187815	0.073948	0.3404	0.0670966	0.039688	0.28993	0.09455458	0.0402813	0.37974
P11404	14077	0.0728198	0.00487	0.7153	0.0722059	0.005272	0.7089	0.04976662	0.003366	0.71065	0.0629566	0.0050522	0.66851
Q9D6J5	67264	0.0247251	0.007464	0.2033	0.0482007	0.036678	0.0458	0.01507904	0.000805	0.8932	0.02115143	0.0075023	0.17299
P21956	17304	0.0416615	0.001247	0.9876	0.0413066	0.002227	0.9663	0.03432245	0.002267	0.94243	0.13387234	0.0921246	0.14964
A7YL62	NA	0.4802085	0.116749	0.4347	0.7006324	0.142429	0.5874	0.54163837	0.137957	0.41199	0.88487659	0.0993466	0.7907
P68254	1.01E+08	0.1566638	0.004462	0.977	0.1775582	0.013822	0.873	0.10380305	0.003725	0.964	0.1525768	0.0052361	0.9714
Q9D1A2	66054	0.0876974	0.003877	0.9552	0.1240797	0.025556	0.4955	0.06607394	0.006889	0.7863	0.07594428	0.0049338	0.91152
Q6ZPL5	NA	0.0590509	0.041382	0.4043	0.777722	0.399276	0.4868	0.0469486	0.012054	0.79133	0.06441536	0.0364202	0.3427
Q99PT1	192662	0.0759731	0.001077	0.9897	0.0828323	0.001597	0.9817	0.05434315	0.001513	0.95698	0.07483496	0.0010469	0.99031
Q9D0S9	68917	0.0435839	0.001434	0.9696	0.0403131	0.001575	0.9633	0.02948692	0.001245	0.95087	0.0335194	0.0014281	0.95659
Q91VI7	107702	0.3029586	0.062311	0.5069	0.2365165	0.069497	0.3667	0.23751029	0.053194	0.46432	0.20627101	0.0283068	0.76845
Q9Z0X1	26926	0.0381419	0.00892	0.1209	0.061591	0.012653	0.1759	0.03224751	0.003898	0.35203	0.02566586	0.0045744	0.22095
Q9EQ20	104776	0.0438259	0.001776	0.7106	0.0378889	0.001718	0.6906	0.02991279	0.005326	0.11323	0.03571148	0.0056865	0.1538
P14094	11931	0.0930734	0.027647	0.2048	0.0815134	0.034333	0.1354	0.04600121	0.002477	0.88685	0.09271595	0.0351144	0.15855
P21300	11997	0.03094	0.002078	0.961	0.0331871	0.004535	0.87	0.02785094	0.001994	0.95126	0.02598171	0.0027731	0.91648
P63017	15481	0.3158114	0.035106	0.468	0.3216826	0.043561	0.4146	0.29736834	0.041186	0.38295	0.20681321	0.0342081	0.33364
Q9DCI3	76205												
P31001	13346	0.2095232	0.037478	0.2363	0.1689258	0.031071	0.2626	0.11716083	0.021181	0.24169	0.1026497	0.0219436	0.20864
P45591	12632	0.3354469	0.063834	0.4556	0.1624056	0.036035	0.374	0.19240908	0.0375	0.403	0.18107571	0.0369915	0.38672
P63038	15510	0.0288296	0.00516	0.1068	0.0293662	0.001268	0.7044	0.02076832	0.000617	0.82101	0.02327714	0.0009722	0.71725
Q6ZQA6	78908	0.1765953	0.11841	0.2411	0.5511653	0.138756	0.7245	0.02391546	0.002604	0.92339	0.40494832	0.1535661	0.49834
P26039	21894	0.2604406	0.067542	0.3043	0.38298	0.072188	0.4841	0.27288978	0.058386	0.3983	0.37627001	0.0655038	0.53223
Q91V12	70025	0.0336363	0.002247	0.9452	0.0275058	0.002668	0.8985	0.02610785	0.002016	0.92808	0.02604158	0.0012179	0.9786
Q9D8S4	104444	0.2403738	0.084798	0.4455	0.4554767	0.177046	0.5245	0.23086665	0.13212	0.23392	0.21314013	0.1529196	0.24459
P45377	14187	0.0364581	0.001596	0.9222	0.0339384	0.002338	0.8472	0.02732928	0.001083	0.9354	0.02925172	0.0017988	0.87436
P58771	22003	0.0881675	0.001789	0.9386	0.0902715	0.009554	0.3981	0.06680132	0.001792	0.89912	0.07300623	0.0018778	0.91745
A8DUK7	NA	0.0433596	0.013403	0.1085	0.0381505	0.006107	0.3484	0.05101038	0.015654	0.1099	0.02706446	0.0044456	0.33983
P01865	NA	0.125378	0.035066	0.2988	0.2486067	0.038536	0.5731	0.15596694	0.020078	0.68305	0.2514518	0.068553	0.35922
P09405	17975	0.1783691	0.024722	0.8002	0.3198236	0.083738	0.5701	0.21162982	0.018675	0.9211	0.15566521	0.0200896	0.83343
Q9CWJ9	108147	0.0674873	0.009479	0.7379	0.1734426	0.042118	0.4851	0.07429474	0.006085	0.90858	0.08061157	0.004694	0.94248
P84244	667250	0.0300038	0.008795	0.7442	0.0176097	0.004278	0.7385	0.02722972	0.004282	0.90998	0.01393132	0.0071431	0.65539
P61089	1E+08	0.0983727	0.002621	0.9902	0.1013214	0.006438	0.9763	0.08297457	0.005778	0.95818	0.09462334	0.003275	0.99168
A2ASS6	22138	0.1235069	0.022518	0.1474	0.1634211	0.027828	0.19	0.10364036	0.021743	0.12434	0.12801806	0.0230684	0.1772
Q3UIU2	230075	0.0272747	0.001212	0.9201	0.0260169	0.001226	0.9221	0.0191234	0.001093	0.87438	0.02096729	0.0011409	0.89887
P08228	20655	0.1790338	0.045432	0.1837	0.1646697	0.047732	0.1655	0.11912011	0.034525	0.14534	0.13062764	0.0407167	0.14043
P49817	12389	0.1251784	0.040715	0.2744	0.0522248	0.005018	0.8441	0.12070701	0.047632	0.2111	0.08090208	0.0206425	0.41114
P12710	14080	0.430815	0.102523	0.4453	0.3593947	0.062904	0.6998	0.35794585	0.086232	0.46281	0.48663927	0.1293895	0.44004
P19123	21924	0.0557017	0.001826	0.9065	0.0526897	0.002057	0.8914	0.0405033	0.001816	0.84686	0.05285252	0.0020724	0.88925
Q99KQ4	59027	0.1338882	0.011976	0.8278	0.1549933	0.010498	0.8971	0.08156011	0.008009	0.7586	0.1534484	0.0074547	0.94218
Q9JKF7	27393	0.0822223	0.093516	0.0561	0.1160867	0.014709	0.8499	0.04303837	0.012479	0.45935	0.09087511	0.1112644	0.05718
Q61037	NA	0.0621176	0.002727	0.9737	0.0616976	0.00137	0.9941	0.05049513	0.001319	0.99054	0.05191541	0.0007255	0.99766

P43274	50709	0.1568264	0.0882	0.1842	0.206454	0.095645	0.2797	0.01870016	0.00497	0.50282	0.02467365	0.0078746	0.44999
Q9CXZ1	NA	0.0377746	0.000923	0.9917	0.0349739	0.001025	0.9898	0.02587695	0.001023	0.97861	0.02786638	0.000933	0.98673
P58774-2	22004	0.0268274	0.005516	0.8255	0.2263448	0.128186	0.2804	0.03017717	0.037594	0.17681	0.0339263	0.0218675	0.54618
Q9R229	12154	0.7196003	0.110403	0.7943	0.6856473	0.248322	0.5596	0.68787863	0.152679	0.77185	0.56338702	0.2058088	0.48366
Q9CR68	66694	0.070646	0.01425	0.1449	0.0557167	0.0139	0.1139	0.04284656	0.014247	0.05833	0.05403551	0.0145477	0.0973
Q60854	20719	0.0997833	0.02743	0.1419	0.1825009	0.03386	0.2739	0.08192618	0.013893	0.29034	0.07320296	0.0069848	0.59747
Q8BGC4	225791	0.2018504	0.06363	0.4015	0.0524365	0.032163	0.1505	0.07155044	0.028733	0.25623	0.05276655	0.017856	0.35308
Q6IRU2	326618	0.2836494	0.057662	0.6335	0.451317	0.092214	0.6311	0.21843975	0.066305	0.41981	0.16240973	0.0096314	0.95951
P19096	14104	0.0938385	0.032216	0.1681	0.1641003	0.052251	0.2249	0.19333008	0.045767	0.29817	0.18706667	0.0503816	0.26622
Q62425	17992	0.04305	0.001342	0.9458	0.0411038	0.001499	0.9365	0.03406471	0.001195	0.93232	0.03479402	0.0012627	0.93706
P23242	14609	0.965545	0.129906	0.8467	0.9468575	0.124035	0.8535	0.171267	0.11788	0.13102	0.26643118	0.1467777	0.2305
O35643	11764	0.1561397	0.017604	0.9752	0.1360132	0.008147	0.9859	0.09985324	0.008845	0.977	0.10897104	0.0065928	0.98914
Q60932	22333	0.039275	0.006815	0.1475	0.0485263	0.010508	0.1145	0.03397753	0.007684	0.09286	0.03757773	0.0066972	0.15396
Q8VCW8	264895	0.0632367	0.004208	0.9186	0.0631449	0.002807	0.962	0.0355827	0.001668	0.9579	0.05145907	0.0022991	0.96346
P10649	14862	0.0754674	0.013347	0.2665	0.127128	0.029878	0.1904	0.05298144	0.00255	0.83223	0.10175199	0.0177035	0.30022
Q9CPQ1	621837	0.0999308	0.089091	0.0825	0.0915971	0.022724	0.5752	0.23132819	0.148953	0.16735	0.10407684	0.0425386	0.35241
Q80WQ9	1E+08	0.0314402	0.002314	0.9295	0.0326742	0.001981	0.9578	0.02315606	0.001895	0.91425	0.02246228	0.0012539	0.96396
P55264	11534	0.1187577	0.002628	0.9874	0.1135839	0.002117	0.9914	0.08008208	0.002082	0.98142	0.10943108	0.0030825	0.98131
P32261	11905	0.1795968	0.015259	0.9082	0.188083	0.025106	0.8239	0.14171874	0.010095	0.93367	0.17219474	0.0203204	0.85682
OTTMUSP0(	NA	0.0083242	0.001087	0.8542	0.0104441	0.00326	0.5945	0.00617624	0.000747	0.88374	0.01001589	0.0013597	0.88573
Q8BLF1	320024	#VALUE!	NA	NA	#VALUE!	NA	NA	#VALUE!	NA	NA	#VALUE!	NA	NA
P60766	12540	0.027688	0.015767	0.2357	0.0094081	0.010065	0.111	0.06270725	0.010242	0.75752	0.0508926	0.0274154	0.32989
Q8BTM8	192176	0.2674722	0.036902	0.9131	0.2196379	0.017869	0.9805	0.34610476	0.123149	0.53016	0.16103819	0.0135267	0.97255
P62827	1E+08	0.1053554	0.008592	0.7322	0.1996566	0.034931	0.3952	0.13031109	0.021698	0.38755	0.1059137	0.0055048	0.87891
Q3ULW3	NA	0.0310686	0.002079	0.941	0.0282617	0.001774	0.9549	0.02179412	0.005657	0.53313	0.02203771	0.0046068	0.65601
Q9CR21	1E+08	0.0289115	0.003062	0.6695	0.089855	0.037103	0.1435	0.02289877	0.00304	0.56321	0.07738155	0.0305944	0.14409
P12246	20219	0.24688	0.142071	0.201	0.426368	0.184065	0.309	0.64704959	0.108664	0.71693	0.32466292	0.1341482	0.328
Q8BH86	217830	0.028144	0.008793	0.2611	0.0169087	0.003296	0.5448	0.01421142	0.002535	0.53792	0.07737068	0.0501106	0.08706
Q60692	19175	0.1260955	0.009537	0.9308	0.0616082	0.021079	0.4158	0.10150872	0.002468	0.99179	0.07215868	0.0116084	0.76303
P58252	13629	0.1831223	0.014721	0.589	0.1961655	0.017519	0.5327	0.12614444	0.006887	0.7686	0.12394057	0.0071086	0.75621
P01942	NA	0.0234691	0.000676	0.87	0.0349865	0.001609	0.7641	0.04107712	0.008183	0.12715	0.04023811	0.0084257	0.13755
Q78IK4	68117	0.0268102	0.002846	0.8013	0.0290393	0.00432	0.7266	0.04613052	0.02131	0.1756	0.02406881	0.003316	0.73494
Q9D020	107569	0.0456065	0.007293	0.697	0.0467363	0.008029	0.6659	0.15241449	0.056366	0.28886	0.0368256	0.0094183	0.48862
Q99KI0	11429	0.0429185	0.003896	0.1409	0.0401339	0.004129	0.1285	0.02993424	0.003126	0.11065	0.02910726	0.0028076	0.14303
Q7TQI3	107260	0.2702453	0.064444	0.575	0.1534656	0.005676	0.9838	0.10087587	0.006447	0.94958	0.16078285	0.0154497	0.9078
P14824	11749	0.1230292	0.011475	0.3082	0.1053912	0.007208	0.4785	0.08808814	0.009436	0.24197	0.0959682	0.0071662	0.43283
Q9JI91	NA	0.0686835	0.008484	0.2932	0.0942078	0.017309	0.18	0.07965233	0.014317	0.16919	0.07236866	0.0135748	0.17827
Q571E4	50917	0.0037605	0.006929	0.0206	0.0113027	0.015922	0.0403	0.0037706	0.007099	0.01975	0.0073665	0.0064287	0.09863
A3KG59	242377	0.283099	0.057134	0.6369	0.2749296	0.141089	0.2404	0.14445154	0.0075	0.96363	0.17698094	0.0118208	0.94919
Q9DCZ1	66355	0.0774134	0.010726	0.6671	0.0645021	0.008787	0.658	0.04438726	0.005788	0.66976	0.09063096	0.0150305	0.58306
P07310	12715	0.0863304	0.009686	0.2293	0.0902366	0.010659	0.2399	0.06378265	0.007345	0.22216	0.05779284	0.0075804	0.20315
P00405	17709	0.2137362	0.052145	0.2054	0.1030973	0.045412	0.0816	0.20972616	0.05505	0.1759	0.17628149	0.063445	0.11747

P63030	1.01E+08	0.0708441	0.002235	0.9863	0.0793771	0.004922	0.9594	0.05512164	0.001994	0.98202	0.05856051	0.0026026	0.97685
Q99J39	56690	0.0211513	0.002743	0.8094	0.0195765	0.003286	0.7634	0.01111016	0.002756	0.53719	0.01404364	0.0037099	0.54423
P50543	1.01E+08	0.344208	0.07837	0.4079	0.348554	0.077224	0.4808	0.23941403	0.071285	0.28004	0.23305247	0.0658346	0.35268
P17710	15275	0.1216868	0.032294	0.1446	0.0722343	0.016495	0.1953	0.06344178	0.021016	0.09581	0.08439529	0.0277166	0.10624
O09111	104130	0.0294752	0.006926	0.3844	0.0240133	0.002425	0.8033	0.01874894	0.002667	0.63015	0.07534444	0.0506009	0.08457
O70433	14200	0.2126436	0.040611	0.4128	0.2836877	0.0729	0.3081	0.17717364	0.048754	0.26304	0.23963005	0.073615	0.24305
P27773	14827	0.3468564	0.032738	0.5416	0.3173554	0.032446	0.5446	0.14344203	0.007989	0.7894	0.17348192	0.0145493	0.63706
P28474	11532	0.2713908	0.047165	0.3716	0.1722926	0.037222	0.3131	0.1429849	0.04801	0.13887	0.24120688	0.0613416	0.23265
Q9CYR0	381760	0.0646917	0.00682	0.8653	0.0603794	0.004724	0.9316	0.04377882	0.003338	0.92474	0.04653111	0.0034561	0.93791
Q9Z1E4	14936	0.0782533	0.008887	0.5597	0.1211404	0.024142	0.3262	0.06754981	0.006912	0.59877	0.0710833	0.0077427	0.59656
P08074	12409	0.3551035	0.075594	0.6293	0.1398958	0.009336	0.9493	0.12727964	0.109937	0.08738	0.13028593	0.0080246	0.95994
O08528	15277	0.2468917	0.032702	0.5089	0.2486892	0.041841	0.424	0.08807839	0.008128	0.66937	0.24411506	0.0380811	0.45111
P62821	19324	0.1748559	0.0125	0.9422	0.1574071	0.018362	0.9018	0.14195764	0.007464	0.9731	0.18101615	0.0133853	0.94327
Q8K411	69617	0.0608284	0.008544	0.7836	0.0512436	0.001494	0.9899	0.03810068	0.001252	0.98511	0.07643319	0.0276559	0.38894
Q9CXV1	66925	0.155425	0.04925	0.2492	0.2015771	0.083638	0.1826	0.05710371	0.041548	0.05924	0.34813164	0.0916805	0.35674
Q9CWL2	69743	0.0016458	0.000304	0.785	0.0029069	0.000399	0.8837	0.00123662	0.000352	0.55289	0.00283528	0.0006414	0.70953
P19536	NA	0.0773755	0.023986	0.0934	0.0522653	0.026688	0.0437	0.10701685	0.038849	0.06924	0.04788589	0.0220648	0.05136
Q922F6	NA	0.5568204	0.111963	0.6555	0.393059	0.075703	0.7102	0.23258145	0.144617	0.19037	0.36097446	0.0702051	0.6878
Q61316	NA	0.3117104	0.039425	0.5231	0.2875807	0.037734	0.5475	0.23672046	0.043238	0.36565	0.26166338	0.0378738	0.52034
Q8BH59	78830	0.0471705	0.010993	0.0867	0.0450033	0.008464	0.1448	0.03111301	0.00734	0.08398	0.05867419	0.0157236	0.0749
Q9CQI6	72042	0.0492396	0.01585	0.4081	0.1477149	0.094977	0.1678	0.06847563	0.010401	0.75585	0.06228857	0.0159064	0.561
Q9D8Y0	NA	0.103507	0.051245	0.1403	0.0158364	0.005288	0.3326	0.09549846	0.038311	0.17645	0.03021793	0.0093961	0.34086
Q9CZ44	386649	0.0211299	0.002249	0.9169	0.0191932	0.002991	0.8547	0.01320479	0.001376	0.88477	0.06437178	0.0216997	0.55696
Q8VDK1	27045	0.0926989	0.015083	0.7296	0.1901463	0.090525	0.2688	0.07921348	0.020532	0.51532	0.07620333	0.0266877	0.40456
Q80X68	NA	0.2750641	0.148443	0.2556	0.080678	0.012041	0.8178	0.1672349	0.090508	0.22149	0.15464582	0.1475944	0.09075
Q9CR62	67863	0.0311391	0.002037	0.6425	0.0630582	0.015601	0.1253	0.02215712	0.000724	0.87551	0.02343075	0.0008607	0.86265
O35943	14297	0.0524934	0.005611	0.9358	0.0326923	0.009459	0.6656	0.04634941	0.002283	0.98095	0.0483776	0.0022987	0.98444
Q9QYG0	29811	0.1944378	0.005206	0.9238	0.2331375	0.008073	0.8949	0.14194055	0.003318	0.94282	0.19797698	0.0131789	0.70156
Q8K0Z7	70207	0.2261727	0.073298	0.4424	0.1149147	0.009003	0.9368	0.19896103	0.080236	0.30517	0.42681613	0.1176542	0.59387
Q9CW03	13006	0.0575748	0.014653	0.7942	0.0745352	0.030558	0.6648	0.03459865	0.030326	0.24551	0.03629404	0.0239309	0.36509
Q61598	14569	0.1331609	0.005159	0.8729	0.1274781	0.005003	0.8806	0.10082099	0.004885	0.80833	0.11857335	0.0047811	0.87359
Q9JMH6	NA	0.1192281	0.012704	0.8001	0.1124408	0.008453	0.885	0.05381847	0.005569	0.78224	0.10727611	0.0143191	0.72772
P80316	12465	0.2387846	0.066529	0.4792	0.1693109	0.016623	0.8963	0.1264768	0.02019	0.73704	0.11509509	0.0096178	0.92268
Q9EPK5	97064	0.0387628	0.002442	0.9474	0.035572	0.001596	0.9764	0.02824254	0.001167	0.97664	0.0290523	0.0048113	0.75238
P35980	19899	0.1106861	0.023504	0.9569	0.0221616	0.010836	0.5823	0.0614772	0.005786	0.95759	0.03279702	0.0149924	0.5447
Q91WS0	52637	0.0725491	0.04125	0.0671	0.1288688	0.049434	0.1626	0.09658269	0.055784	0.06972	0.04216818	0.0339239	0.04009
Q91YY4	246782	0.0492334	0.020397	0.3095	0.0429458	0.004344	0.8989	0.02452155	0.005684	0.65053	0.03160422	0.0036823	0.87007
O70250	56012	0.073797	0.006434	0.5885	0.0712393	0.00421	0.7946	0.08115905	0.019801	0.1603	0.06306267	0.0169232	0.16168
Q06185	11958	0.0258524	0.000944	0.964	0.0244743	0.001068	0.9545	0.01858307	0.000854	0.9423	0.01890648	0.0009892	0.93595
Q6PCY5	NA	0.0410106	0.002024	0.967	0.0381522	0.001799	0.974	0.02574503	0.001686	0.94339	0.03189896	0.0020729	0.95177
Q3UWA4	195359	0.0003446	7.97E-05	0.5718	0.0003909	0.000127	0.4424	0.00049476	0.000139	0.47524	0.00028869	0.0001177	0.33374
Q08857	12491	0.1398253	0.002343	0.9886	0.1444246	0.001993	0.993	0.10370528	0.002528	0.97679	0.13865386	0.0022143	0.9909

P40124	12331	0.2548624	0.068728	0.2764	0.2584899	0.051744	0.3902	0.37147347	0.084201	0.32732	0.39464393	0.0680008	0.46341
P03911	17719	0.0257471	0.001119	0.9233	0.0246822	0.001019	0.9392	0.01916593	0.000864	0.91959	0.0207394	0.0008774	0.93789
Q3V3A1	271697	0.0653686	0.008506	0.9517	0.0705315	0.005135	0.9792	0.04568937	0.00312	0.98169	0.05992828	0.0029309	0.99524
Q8R404	224904	0.0520209	0.00421	0.916	0.0413161	0.00229	0.9644	0.02752365	0.010627	0.34038	0.02822352	0.0015462	0.97086
Q9D6R2	67834	0.0338762	0.011091	0.0727	0.0235337	0.001031	0.8322	0.01722125	0.001413	0.55713	0.01959932	0.0023966	0.38911
Q99LY9	1.01E+08	0.0242431	0.001154	0.9384	0.0212669	0.001301	0.9145	0.01657757	0.000906	0.92033	0.01811434	0.0011992	0.90126
Q9D1L0	1E+08	0.8538808	0.105661	0.8132	0.9900193	0.097374	0.896	0.61461326	0.078679	0.81339	0.96747096	0.1135059	0.85824
Q8QZR5	76282	0.1149134	0.010465	0.9095	0.1306931	0.007415	0.9658	0.08168891	0.010907	0.87518	0.10611408	0.0119332	0.88773
A2A9C3	230676	0.0097756	0.005693	0.4957	0.00722	0.002301	0.496	0.01585587	0.010402	0.53743	0.01074974	0.0048368	0.55254
Q9QUH0	93692	0.0334628	0.004081	0.838	0.0362956	0.003904	0.8781	0.03636236	0.002471	0.93927	0.03181978	0.0024051	0.93584
Q8K0D5	28030	0.2376436	0.109254	0.2526	0.1409252	0.020349	0.8275	0.11092133	0.01955	0.72845	0.17830946	0.1068549	0.21781
P43023	12862	0.0477103	0.004368	0.8099	0.0435711	0.004814	0.7734	0.03421002	0.002983	0.81931	0.04601168	0.004207	0.82713
Q7TNG8	52815	0.1566014	0.024387	0.8375	0.1555114	0.031781	0.7054	0.11940475	0.035878	0.61274	0.12601836	0.0391744	0.53484
Q8R2G4	109979												
P48774	14866	0.1280851	0.00853	0.9912	0.0986882	0.0137	0.8964	0.05193249	0.011289	0.84104	0.08056745	0.0070214	0.94272
Q0KK59	217843	0.0100031	0.002359	0.7498	0.0067901	0.00169	0.7291	0.00409161	0.00056	0.86985	0.00578526	0.005661	0.11547
P05125	NA	0.2804652	0.057454	0.2461	0.4094666	0.061323	0.3824	0.27277782	0.044898	0.33583	0.4347182	0.0709309	0.35923
Q60936	67426	0.0455357	0.107376	0.0111	0.1529063	0.149682	0.065	0.07062066	0.109151	0.02403	0.1780605	0.1402302	0.09706
Q01853	269523	0.2593916	0.01986	0.5009	0.2769374	0.022585	0.4924	0.22683793	0.02033	0.43304	0.22780894	0.0202007	0.47423
Q8BM89	271970	0.0030245	0.003477	0.1314	0.0031319	0.007944	0.0374	0.00158191	0.000348	0.72138	0.0018144	0.0007332	0.4666
P56376	66204	0.0659548	0.001707	0.9907	0.061006	0.002035	0.9868	0.05472295	0.00339	0.94901	0.05558355	0.0016161	0.99079
P03930	17706	0.0346715	0.004516	0.5726	0.0305107	0.002303	0.8259	0.07800878	0.044392	0.067	0.02836855	0.044327	0.01066
O08804	NA	0.0857297	0.008748	0.8728	0.0790584	0.004313	0.9655	0.11428682	0.117243	0.06811	0.05742933	0.0048446	0.9274
Q8CI51	56376	0.079768	0.006226	0.9214	0.0918139	0.005442	0.9596	0.04936577	0.007804	0.78438	0.07505347	0.0042551	0.96585
P62270	1E+08	0.0737084	0.007261	0.888	0.0688712	0.008662	0.8405	0.05901827	0.004731	0.91747	0.05571088	0.0032852	0.95994
Q9DC70	75406	0.062513	0.017223	0.1664	0.0688072	0.0208	0.1565	0.03832368	0.003602	0.62824	0.05021441	0.004759	0.66139
P24527	16993	0.2090934	0.074291	0.2647	0.1546104	0.035758	0.3688	0.13843357	0.045196	0.28972	0.18533072	0.0629243	0.25018
O35459	51798	0.1503961	0.002022	0.98	0.1271911	0.002578	0.9594	0.10374139	0.002064	0.95901	0.13015365	0.0026918	0.95741
Q8VHN7	110789	0.0615155	0.087961	0.0338	0.0237128	0.017638	0.1531	0.22236138	0.151747	0.16332	0.15789165	0.1748645	0.07538
Q5SWD9	104662	0.0358712	0.003426	0.9821	#VALUE!	NA	NA	#VALUE!	NA	NA	0.05049244	0.0126949	0.84059
Q3TIT9	NA	0.0176477	0.001686	0.8867	0.0172882	0.001095	0.9541	0.01458004	0.002539	0.70191	0.01313496	0.0022596	0.73793
P50396	14567	0.1848076	0.073284	0.2844	0.103074	0.013991	0.8068	0.18378233	0.071935	0.31798	0.09541645	0.015666	0.77129
Q9WV98	30056	0.4445434	0.121918	0.3565	0.3223863	0.132194	0.271	0.13017545	0.085648	0.14164	0.42316237	0.1332884	0.35896
P35505	14085	0.074576	0.008675	0.8407	0.0606259	0.003528	0.9609	0.04600778	0.017203	0.33813	0.12283796	0.1041453	0.10389
P22599	20701	1.0512348	0.1095	0.8443	1.1502927	0.158921	0.755	0.89813754	0.068318	0.88708	0.8689573	0.1230892	0.71362
O35678	23945	0.2208952	0.044675	0.7534	0.209072	0.017265	0.9362	0.14707701	0.010671	0.95477	0.24744897	0.0251657	0.92358
P06909	NA	0.172176	0.077775	0.2738	0.5044404	0.108489	0.6431	0.22352455	0.115466	0.22377	0.11452687	0.026646	0.60622
P50462	13009	0.0653615	0.001893	0.9621	0.0607395	0.004506	0.8159	0.04915697	0.003203	0.83658	0.05809671	0.0031111	0.8971
Q505D7	403187	0.006591	0.00201	0.4343	0.0052459	0.000735	0.8095	0.00649973	0.001263	0.65415	0.00329618	0.0011618	0.40147
P41216	14081	0.057444	0.005801	0.2482	0.069743	0.009016	0.1919	0.04168919	0.004215	0.24654	0.03531745	0.0008777	0.85982
P22907	15288	0.1089648	0.019114	0.8025	0.1484086	0.006587	0.9902	0.14107651	0.02796	0.86422	0.11480959	0.0074399	0.97144
Q9CXJ4	74610	0.0562628	0.013475	0.5375	0.1830848	0.105044	0.202	0.11283647	0.016576	0.76797	0.12552431	0.1219311	0.08788

P28656	53605	0.8633409	0.087112	0.8752	0.6879002	0.081845	0.8548	0.57004869	0.098766	0.7041	0.72591611	0.0852936	0.85788
O08600	13804	0.1321122	0.009129	0.9544	0.1687009	0.012739	0.9564	0.0723962	0.005837	0.92209	0.15881361	0.0166616	0.91907
Q9WVA4	21346	0.3943397	0.048976	0.5746	0.4103036	0.058412	0.5175	0.27388587	0.04585	0.39788	0.21818047	0.0411574	0.40087
P09813	NA	2.2703792	0.081725	0.9923	2.2663895	0.201464	0.9694	0.84193799	0.099333	0.84677	1.12537011	0.1965378	0.78462
Q99LD8	51793	0.3957683	0.270555	0.211	0.1251241	0.013456	0.8963	0.08590494	0.014898	0.84714	0.13464406	0.0109046	0.9561
A2AKY4	241514	0.0239761	0.003232	0.7972	0.0296735	0.001927	0.9518	0.01279595	0.001378	0.86036	0.02159851	0.0031708	0.79452
Q99LP6	17713	0.0782735	0.010871	0.6927	0.0811135	0.009236	0.786	0.06581096	0.007413	0.74485	0.05631845	0.0092082	0.64045
P62204	12315	0.1200683	0.002341	0.9891	0.1346355	0.003963	0.9788	0.09190885	0.002362	0.98121	0.12942308	0.0027608	0.98875
O08797	NA	0.084619	0.021695	0.7172	0.1337021	0.012829	0.9395	0.09475854	0.016433	0.86929	0.10488218	0.0175116	0.92282
P35969	NA	0.0809621	0.002673	0.9787	0.0863143	0.003756	0.9724	0.0639334	0.002687	0.97251	0.06779419	0.0028977	0.9768
P27005	20201	0.1587975	0.076752	0.2342	0.1465867	0.088062	0.1652	0.24818021	0.102429	0.29544	0.21559907	0.0945576	0.28567
Q3UFU2	NA	0.0663428	0.011176	0.8545	0.0859229	0.005488	0.9722	0.0505526	0.007707	0.82702	0.07684674	0.0071241	0.95096
P68510	22629	0.1689326	0.020456	0.724	0.2942161	0.077739	0.4173	0.17269273	0.047567	0.40958	0.13195411	0.0046965	0.9765
Q6XPS7	NA	0.0224424	0.002978	0.8765	0.0281231	0.00289	0.9221	0.0211294	0.001287	0.97823	0.03389271	0.0085963	0.68951
P24270	12359	0.1224182	0.021803	0.2878	0.1669905	0.035963	0.255	0.19728117	0.031985	0.33954	0.27765178	0.0381278	0.44552
Q8C196	227231	0.1863796	0.085154	0.2549	0.6784731	0.175137	0.6001	0.18256257	0.11698	0.15779	0.54151117	0.2152043	0.36532
Q8BH64	259300	0.0409586	0.002866	0.9533	0.0419015	0.010199	0.7377	0.07564416	0.055479	0.48174	0.04979913	0.0052179	0.92864
Q09LZ8	78933	0.024754	0.001974	0.9236	0.0219102	0.001508	0.9462	0.01707769	0.001232	0.93206	0.01867355	0.0013499	0.94099
Q9CQ89	67675	0.0734378	0.010393	0.735	0.0633196	0.003833	0.9479	0.04716293	0.014754	0.40521	0.06646714	0.0959582	0.03314
Q00519	22436	0.1500518	0.006795	0.9439	0.2252042	0.040304	0.5553	0.10390011	0.008079	0.85521	0.15779715	0.0102182	0.90512
Q60931	22335	0.1078787	0.025327	0.145	0.1309281	0.035132	0.1252	0.06520705	0.018785	0.10383	0.0746061	0.0296156	0.06141
P52480	18746	0.1262727	0.008082	0.4102	0.1178856	0.008762	0.3671	0.10263421	0.008207	0.3076	0.09984756	0.0101274	0.23989
O35215	13202	0.050141	0.002253	0.8476	0.0540792	0.002494	0.8593	0.03778681	0.001472	0.88104	0.04153876	0.0020411	0.84496
Q9DCJ5	68375	0.0535775	0.020743	0.0683	0.0511255	0.02886	0.0377	0.04622622	0.023611	0.03958	0.09680995	0.0367751	0.07706
Q61599	11857	0.527528	0.083432	0.5969	0.358481	0.065565	0.5652	0.21832347	0.044283	0.49297	0.42003147	0.0624215	0.64427
Q8K2B3	66945	0.052722	0.005458	0.2291	0.0585685	0.010103	0.1082	0.04207835	0.006729	0.1089	0.04063992	0.0067093	0.11734
Q05816	16592	0.2083408	0.04042	0.3218	0.2223525	0.039929	0.3781	0.1229546	0.022521	0.33564	0.2540868	0.0633521	0.24715
P70188	16579	0.0014561	0.000866	0.168	0.0015	0.000744	0.2531	0.00222674	0.00076	0.38023	0.00138178	0.0007988	0.1996
Q9R1P1	1.01E+08	0.1140331	0.00337	0.9879	0.1226671	0.004331	0.9853	0.08868184	0.002866	0.98559	0.09652806	0.0148337	0.7938
Q9D1M0	110379	0.8164579	0.067548	0.9126	0.5513536	0.109186	0.68	0.58814222	0.078932	0.79862	0.50517512	0.1019429	0.67174
Q5R1I7	NA	0.091838	0.024694	0.6058	0.0654048	0.01308	0.8065	0.30401646	0.108295	0.39641	0.13683762	0.033207	0.65359
P99027	67186	0.4577985	0.069168	0.7578	0.6494949	0.071038	0.8745	0.32183309	0.059255	0.67816	0.39223926	0.0711086	0.71716
Q9EQP2	98878	0.0783217	0.03799	0.1278	0.0928951	0.047468	0.1328	0.0294921	0.009392	0.25373	0.03689706	0.0191647	0.12912
Q2TPA8	72479	0.1017926	0.016365	0.2378	0.0710669	0.013519	0.2084	0.06684889	0.010686	0.23698	0.12102489	0.0201779	0.23987
P99024	22154	0.0744314	0.006407	0.8437	0.0976986	0.005048	0.9493	0.06999081	0.005819	0.87324	0.09103496	0.0080098	0.8777
Q8VDC0	102436	0.0523701	0.006318	0.9075	0.0512224	0.002401	0.987	0.03721304	0.003465	0.94278	0.04862457	0.0025075	0.99208
Q9CQZ6	66495	0.0289234	0.001742	0.9048	0.0310266	0.003004	0.8102	0.02094116	0.001601	0.85502	0.02145344	0.0014915	0.8922
ENSMUSP0(	NA	0.3597385	0.1824	0.4376	0.2541436	0.158426	0.3398	0.88912676	0.274847	0.91278	0.32722481	0.2123948	0.44171
Q30D77	71355	0.1187497	0.022482	0.6659	0.2955639	0.117566	0.345	0.14428778	0.032407	0.58609	0.25935594	0.1349397	0.23538
Q62426	13014	0.1199846	0.005928	0.949	0.1470669	0.012197	0.8634	0.08034238	0.004265	0.92446	0.29070667	0.0686561	0.44902
P61982	22628	0.1135798	0.005492	0.9125	0.1026407	0.004444	0.9303	0.07832616	0.002877	0.93917	0.09000193	0.005194	0.88244
P26041	17698	0.1962384	0.033646	0.3865	0.2943327	0.060588	0.3122	0.12218518	0.028901	0.23872	0.22438336	0.0432464	0.35459

Q9CQV8	54401	0.371261	0.062295	0.5592	0.3537417	0.060139	0.571	0.11675404	0.006668	0.91087	0.16167476	0.0062449	0.96404
Q91YT0	17995	0.04914	0.001399	0.8412	0.0452487	0.001687	0.7808	0.03556678	0.001593	0.68135	0.03832089	0.0014945	0.76409
P56392	12865	0.2419599	0.063564	0.287	0.2630644	0.079321	0.2619	0.30951114	0.084162	0.26249	0.23540905	0.0655578	0.26922
A2AMM0	68016	0.0988702	0.01363	0.7898	0.0878296	0.009504	0.8768	0.0595358	0.006329	0.8634	0.06219255	0.0049782	0.9286
P20108	11757	0.0308747	0.00251	0.6599	0.0259033	0.003322	0.4758	0.0241677	0.002827	0.48054	0.02856494	0.0034819	0.48663
P54726	19358	0.0785169	0.004756	0.9578	0.1051122	0.007374	0.9807	0.05166244	0.009635	0.80418	0.08653754	0.0043964	0.98726
Q8R1A4	NA	0.0649437	0.005773	0.9068	0.0644249	0.00563	0.9291	0.04457419	0.008485	0.66342	0.04461673	0.0042057	0.90365
Q9QXW2	30839	0.0122033	0.024889	0.0292	0.0278833	0.07422	0.0449	0.00632716	0.000955	0.93606	0.00457683	0.0125837	0.04223
Q9WUM5	56451	0.0252196	0.001323	0.7693	0.0224545	0.000589	0.9368	0.02051972	0.001389	0.65879	0.02070861	0.0012499	0.73691
P48772	12869	0.365077	0.055718	0.9555	0.0598559	0.188665	0.0197	0.07099998	0.014918	0.84992	0.03322116	0.0224924	0.35291
Q01768	18103	0.0878188	0.014827	0.3307	0.0715485	0.002373	0.9381	0.12128661	0.027386	0.21887	0.05943862	0.0017729	0.94772
P56375	75572	0.0690784	0.002265	0.9852	0.0619922	0.001883	0.9891	0.04333486	0.00233	0.96109	0.05742052	0.0017447	0.98904
Q3T9C9	NA	0.017803	0.001399	0.9204	0.0234103	0.0024	0.888	0.01329809	0.000753	0.95699	0.01365818	0.0008665	0.95393
Q9QZB1	58175	0.064373	0.103181	0.0291	0.0009108	0.000871	0.0835	0.00184747	0.000982	0.20194	0.00296132	0.0015716	0.22833
Q3UAI4	NA	0.0213581	0.000578	0.9899	0.021135	0.000789	0.9835	0.0166474	0.000544	0.98529	0.01685367	0.0008939	0.96734
P07356	12306	0.0859402	0.014739	0.1726	0.0838742	0.010907	0.2775	0.08034244	0.012861	0.1885	0.08257692	0.0102454	0.3094
P16125	433229	0.1238363	0.010746	0.3897	0.1269112	0.01213	0.3821	0.08341638	0.005912	0.49264	0.09253123	0.0065426	0.52913
Q60590	18405	0.8682667	0.138091	0.8317	1.0384956	0.258772	0.697	0.79877425	0.063067	0.91973	0.88521071	0.1298992	0.85305
Q9D051	68263	0.0368977	0.001219	0.8396	0.0297639	0.001062	0.837	0.02482744	0.000925	0.80447	0.02377141	0.0008399	0.84052
Q9CPV4	67201	0.1264811	0.020583	0.3943	0.0820526	0.005351	0.8189	0.10559686	0.023456	0.26927	0.06396341	0.0031323	0.87975
O35855	12036	0.0497531	0.012893	0.1285	0.0294178	0.004787	0.2979	0.02256445	0.000844	0.87624	0.0251753	0.0013156	0.8027
Q61704	16426	0.5909848	0.221068	0.5436	1.2465573	0.561892	0.5517	0.95175815	0.378168	0.44189	0.54757766	0.2527751	0.40134
Q9WUU7	64138	0.2971113	0.109886	0.3993	0.7228373	0.193514	0.6079	0.74142361	0.140568	0.69865	0.4712211	0.1218907	0.55466
P60335	23983	0.0275348	0.001278	0.9707	0.0208996	0.004087	0.6854	0.03183225	0.012528	0.31562	0.01805981	0.0022521	0.85393
Q8VEM8	1E+08	0.0352262	0.002835	0.5373	0.0460398	0.016815	0.0655	0.02826773	0.00311	0.39414	0.06932467	0.0206852	0.08691
P04117	11770	0.0670504	0.001557	0.9469	0.0736517	0.002434	0.9105	0.03896792	0.001779	0.82187	0.06603298	0.0013797	0.96219
P51881	11740	0.1014403	0.034346	0.1055	0.1789117	0.046268	0.1918	0.16907353	0.045526	0.15891	0.07527733	0.0440621	0.04362
P63330	19052	0.0819553	0.007505	0.9371	0.0832335	0.009103	0.8932	0.06182376	0.009694	0.80266	0.09709751	0.004146	0.98562
P00158	17711	0.0211102	0.000718	0.9841	0.0162841	0.001548	0.9022	0.01401884	0.001012	0.93205	0.01439482	0.0007881	0.96528
P56379	70257	0.0784347	0.013712	0.7003	0.0965147	0.013545	0.8088	0.04059024	0.010059	0.53769	0.05304616	0.0155857	0.49118
ENSMUSP0(	NA	1.0668574	0.314726	0.7418	0.7289617	0.037882	0.9867	0.70403564	0.074285	0.8909	0.82951507	0.0507361	0.97448
Q8C801	NA	0.3230254	0.15022	0.2782	0.0875892	0.043326	0.2901	0.48725657	0.180386	0.42184	0.30918603	0.1921622	0.19051
P70392	NA	0.0122235	0.001993	0.79	0.0130749	0.002026	0.8064	0.01280538	0.001882	0.80795	0.0153432	0.0080389	0.24878
Q9JK42	18604	0.1489561	0.01531	0.8114	0.2058181	0.061341	0.3848	0.25798439	0.078447	0.32958	0.10732786	0.0172426	0.67097
Q62234	17929	0.1254155	0.030315	0.2589	0.0890223	0.035437	0.1363	0.06566064	0.00502	0.78448	0.05511508	0.0126082	0.32884
P97429	11746	0.1651395	0.048528	0.272	0.2666885	0.068377	0.303	0.16951156	0.04682	0.28429	0.14591835	0.0502151	0.24515
P56391	110323	0.0241384	0.000776	0.9709	0.022322	0.00077	0.9711	0.01722201	0.000846	0.93467	0.01786863	0.0006785	0.9652
Q6ZQ80	NA	0.1951861	0.057394	0.794	0.1051082	0.04217	0.7565	0.78265217	0.686088	0.39418	#VALUE!	NA	NA
P63028	22070	0.2650148	0.067248	0.5087	0.2057409	0.014296	0.9367	0.15646457	0.005239	0.97807	0.27831463	0.1074475	0.35861
XP_894449	NA	0.0255566	0.002408	0.8894	0.0200916	0.00346	0.7713	0.02297051	0.004208	0.68036	0.02679604	0.0090858	0.42023
Q99MR8	72039	0.0414864	0.004067	0.6625	0.0341907	0.002982	0.7536	0.0250223	0.001695	0.8015	0.02612309	0.0021725	0.74689
Q3ULD5	78038	0.0456898	0.006919	0.3873	0.1982582	0.045007	0.2573	0.14394718	0.035255	0.19925	0.0927289	0.0274865	0.15301

Q60930	22334	0.0746969	0.018484	0.1263	0.0567725	0.018359	0.0897	0.05497706	0.018789	0.07101	0.04770481	0.0135569	0.11018
P08113	22027	0.2243015	0.035504	0.4343	0.3009079	0.051783	0.413	0.18544231	0.036681	0.32954	0.19707731	0.0335115	0.45159
P80314	12461	0.0674713	0.043218	0.3786	0.1912907	0.007196	0.9916	0.11814616	0.005049	0.98916	0.14423163	0.0171566	0.92175
A2AFQ2	NA	0.0256997	0.001916	0.8071	0.0230352	0.000708	0.968	0.01746709	0.001023	0.87671	0.01730886	0.0007289	0.94314
Q9R0Y5	11636	0.0839631	0.008468	0.3866	0.0913493	0.015679	0.2058	0.04814297	0.001875	0.81671	0.05377327	0.0017977	0.87314
P29758	18242	0.3403896	0.036099	0.6011	0.2754381	0.029639	0.6242	0.21750094	0.027309	0.52236	0.17248727	0.0077827	0.90261
Q9R1P0	26441	0.109298	0.011915	0.9439	0.0936864	0.008804	0.9418	0.06880921	0.006495	0.91075	0.10929236	0.0214649	0.76419
Q61838	11287	0.6869832	0.023125	0.7425	0.6827346	0.02261	0.7556	0.41879472	0.019416	0.58211	0.61486149	0.0217234	0.72887
Q04447	12709	0.1243569	0.03253	0.1791	0.1516616	0.035141	0.2254	0.10258116	0.027758	0.15233	0.20602054	0.046787	0.26785
Q9QWK4	11801	0.2307126	0.074677	0.443	0.4896547	0.144698	0.4683	0.12885236	0.021613	0.7322	0.38668243	0.1252187	0.46436
P61979	15387	0.5888094	0.100215	0.7264	0.8897863	0.08846	0.9101	0.6339534	0.091024	0.77603	0.80166896	0.0976604	0.85966
Q8R1I1	622178	0.0245729	0.000704	0.9886	0.0202297	0.001009	0.971	0.01743042	0.00088	0.96554	0.01730115	0.0010119	0.96057
A2AQ07	NA	0.2079694	0.085611	0.3297	0.1747642	0.095862	0.232	0.32158935	0.17425	0.20761	0.33218148	0.130516	0.35057
P67778	237880	0.044777	0.002906	0.6834	0.0750962	0.018342	0.1715	0.0332188	0.002512	0.62925	0.05969014	0.0148813	0.15457
Q19LI2	117586	0.1077861	0.026133	0.135	0.5345538	0.043941	0.523	0.46349663	0.036499	0.52483	0.54646763	0.0414919	0.55872
Q9D1G1	76308	0.1991298	0.024433	0.8579	0.1708063	0.014639	0.9511	0.1351071	0.01147	0.93278	0.17192511	0.0349231	0.75183
Q68FD5	67300	0.1312173	0.005603	0.9564	0.1292689	0.00736	0.9334	0.09321005	0.004869	0.93139	0.10872408	0.0085643	0.8896
OTTMUSP0(	NA	0.0781359	0.013364	0.7916	0.1347572	0.013301	0.9448	0.08999553	0.010364	0.94962	0.07452419	0.0040008	0.9886
Q91ZJ5	216558	0.1939119	0.022769	0.5053	0.1471716	0.00312	0.9691	0.10755089	0.002551	0.96004	0.14058496	0.0171521	0.48972
Q80XN0	71911	0.0633833	0.018529	0.0938	0.0793685	0.023068	0.1005	0.01726786	0.000961	0.72566	0.03877256	0.0115451	0.09957
ENSMUSP0(	NA	0.036553	0.023009	0.3868	0.1186697	0.066565	0.4428	0.0389394	0.025782	0.43194	#VALUE!	NA	NA
Q6P3A8	12040	0.357537	0.070811	0.4079	0.322825	0.077326	0.3262	0.20126689	0.052721	0.26225	0.25320075	0.0621387	0.34879
O09131	14873	0.0771285	0.004957	0.9132	0.0788948	0.003699	0.9559	0.05487376	0.003443	0.91369	0.06983869	0.0044141	0.92945
O08756	NA	0.0168647	0.002777	0.672	0.0244748	0.004699	0.6011	0.11842633	0.06432	0.16626	0.04072848	0.0152592	0.28356
Q9WTP6	11637	0.0479842	0.006247	0.3554	0.0779789	0.018272	0.1623	0.03923885	0.005324	0.35664	0.07528048	0.0183709	0.15873
O08638	NA	0.0924485	0.041977	0.306	0.1080133	0.03267	0.6861	0.03934583	0.006193	0.81769	0.08635482	0.0080965	0.94203
Q69ZN8	NA	0.0335038	0.002251	0.9406	0.0320799	0.005236	0.7578	0.02318281	0.002281	0.88066	0.02656071	0.0039634	0.78914
P52503	631040	0.0585064	0.001688	0.9203	0.058555	0.003238	0.7842	0.03643555	0.000939	0.9354	0.04555433	0.0012015	0.94108
Q91VD9	227197	0.0553233	0.006679	0.1929	0.0734965	0.012463	0.1261	0.03093364	0.004531	0.14097	0.04821287	0.0108111	0.07452
Q61425	15107	0.0662815	0.013215	0.1238	0.0424358	0.009241	0.1233	0.05389389	0.01235	0.09714	0.04106778	0.0083258	0.13644
Q3TLR7-2	NA	0.0399904	0.005942	0.8499	0.0396333	0.006692	0.7958	0.02212981	0.002801	0.8619	0.01958477	0.0071147	0.48644
Q9DCB8	74316	0.1240121	0.005241	0.9756	0.1244593	0.005204	0.9794	0.09292646	0.005733	0.9494	0.10791414	0.0046174	0.9785
P10107	16952	0.1775672	0.032595	0.2459	0.2123659	0.033664	0.3215	0.13130581	0.021364	0.28665	0.23293946	0.0391131	0.30453
Q61735	16423	0.1117227	0.013455	0.8312	0.134733	0.013831	0.8877	0.11282705	0.009633	0.91344	0.11251669	0.008809	0.93149
Q8VED5	223917	0.0138509	0.003757	0.6601	#VALUE!	NA	NA	#VALUE!	NA	NA	0.00459983	0.0014256	0.83886
P38060	15356	0.1924484	0.086661	0.2605	0.1086582	0.016912	0.7748	0.08536876	0.013186	0.76328	0.10876936	0.0173234	0.79766
P08226	11816	0.2134615	0.06621	0.4261	0.1950527	0.164248	0.1052	0.23078244	0.107334	0.24825	0.28616012	0.1249537	0.32286
Q7TNS2	433771	0.0259801	0.005087	0.9631	#VALUE!	NA	NA	#VALUE!	NA	NA	0.03080233	0.0062037	0.89151
Q8BMF4	235339	0.0387153	0.005612	0.1798	0.0300533	0.00331	0.3083	0.02504293	0.002042	0.41856	0.03783836	0.0088151	0.08969
P47708	19894	0.0168318	0.001117	0.942	0.0272727	0.004963	0.7157	0.02443491	0.015381	0.16258	0.02200369	0.0017272	0.93115
Q9WTR5	12554	0.0672419	0.043298	0.0556	0.1084195	0.035818	0.2029	0.05318777	0.031331	0.06281	0.03947445	0.0340744	0.035
Q9JII6	58810	0.0735226	0.006836	0.8785	0.0740274	0.005823	0.9151	0.04698817	0.002896	0.9327	0.07524831	0.0041308	0.95675

Q922B1	107227	0.0440609	0.013899	0.1015	0.0377786	0.005875	0.3524	0.02797002	0.002326	0.61646	0.03213398	0.0031857	0.55983
Q9DB29	67732	0.0733834	0.014959	0.6493	0.0520656	0.008682	0.7498	0.05907429	0.006175	0.87562	0.05549007	0.0167577	0.47746
Q6PB66	72416	0.1454801	0.018157	0.3306	0.1062415	0.016611	0.2624	0.08664869	0.010783	0.32034	0.10065558	0.0126249	0.36414
Q61207	19156	0.4595573	0.051949	0.5743	0.4985782	0.052852	0.6403	0.18193922	0.019397	0.61105	0.42441334	0.0463738	0.6357
Q9CPX8	66594	0.052232	0.000922	0.9957	0.0506365	0.000912	0.9961	0.03825808	0.000992	0.99068	0.04296229	0.0011945	0.99081
Q8R1G2	69574	0.0823723	0.002653	0.9887	0.0626801	0.010115	0.7934	0.05479362	0.013073	0.61494	0.05854806	0.0039112	0.96971
Q9DB77	67003	0.0418199	0.0084	0.0909	0.047484	0.011554	0.0779	0.03796487	0.009326	0.06409	0.039636	0.0086611	0.08692
Q9CQ54	675851	0.2600493	0.062148	0.2847	0.2499679	0.068252	0.2609	0.11873182	0.040209	0.16539	0.13131295	0.0442234	0.18833
P62082	20115	0.1783008	0.049087	0.3547	0.1993981	0.049856	0.4102	0.10079997	0.05835	0.11485	0.13136361	0.0552938	0.18418
Q922R8	NA	0.0395061	0.018264	0.5391	0.1887244	0.054079	0.5254	0.18795433	0.282079	0.09991	0.36979016	0.1410797	0.57878
Q8BGH2	68653	0.158946	0.060277	0.1481	0.1812031	0.067528	0.1837	0.19727895	0.057817	0.22116	0.0542905	0.0354967	0.06101
P50247	269378	0.0796796	0.017191	0.2323	0.0594829	0.006655	0.5671	0.04769849	0.003975	0.65449	0.04889257	0.0196077	0.0861
Q91WK1	66701	0.0713298	0.001928	0.9949	0.0627501	0.003373	0.9747	0.04221266	0.002612	0.94914	0.0540256	0.0011376	0.99691
Q9CPY7	66988	0.0602898	0.006441	0.9777	0.0690996	0.002338	0.9989	0.06338056	0.023394	0.4785	0.09834442	0.0320229	0.82504
P62259	22627	0.1243902	0.016535	0.3685	0.1049863	0.004291	0.8718	0.07912125	0.003125	0.86859	0.07957847	0.0041454	0.81618
Q8K2C6	68346	0.484083	0.118436	0.603	0.3266094	0.158041	0.2625	0.17305626	0.091976	0.20183	0.77636234	0.1855256	0.59338
P37804	21345	0.1466656	0.024021	0.3755	0.3424123	0.065091	0.3756	0.21325654	0.057909	0.1978	0.15225295	0.0362909	0.2683
Q8BWT1	52538	0.0269801	0.001051	0.6751	0.0258656	0.001525	0.5113	0.02046241	0.001363	0.41637	0.03090302	0.0062443	0.08178
Q62188	22240	0.6532024	0.130463	0.6417	0.5583058	0.08066	0.7997	0.39187255	0.09481	0.56787	0.41376758	0.1025564	0.61945
Q78IK2	1.01E+08	0.0416909	0.001001	0.992	0.0383835	0.001009	0.9918	0.02697762	0.001297	0.96865	0.03228485	0.0009033	0.99069
Q920B9	NA	0.077873	0.076898	0.0683	0.0574298	0.015946	0.5194	0.08158144	0.025843	0.39917	0.07074372	0.032092	0.28823
O88492	57435	0.5569317	0.059762	0.7696	0.4658676	0.062306	0.7085	0.24556058	0.053941	0.42534	0.33461292	0.056096	0.59719
Q9CQN1	68015	0.2069196	0.01772	0.684	0.2051139	0.011335	0.8517	0.20475034	0.02467	0.51837	0.16450684	0.0082201	0.87541
P17665	1E+08	0.0993002	0.00198	0.9949	0.1027341	0.006799	0.9501	0.22076108	0.078383	0.37895	0.17824195	0.0932903	0.23325
O35887	12321	0.3890608	0.07695	0.4365	0.3163049	0.079452	0.3057	0.23759286	0.056805	0.28919	0.20390546	0.0513321	0.31698
Q9DBL1	66885	0.0710904	0.024139	0.121	0.0656277	0.028145	0.093	0.08692725	0.019754	0.2351	0.08421255	0.0314069	0.10862
P16332	17850	0.1112914	0.026608	0.2201	0.0758924	0.00638	0.7238	0.05467602	0.026954	0.06961	0.07803661	0.0141233	0.39893
O55237	21948	0.3137451	0.164302	0.2883	0.2305076	0.099593	0.3488	0.36659744	0.162992	0.28013	0.07632196	0.0205561	0.55619
Q9D8W5	66997	0.0713458	0.024237	0.4406	0.1035207	0.023399	0.6619	0.08286567	0.013332	0.763	0.09200889	0.0143792	0.83655
Q9R111	14544	0.078865	0.096824	0.0622	0.0615234	0.00579	0.9112	0.28013128	0.115086	0.35007	0.06272937	0.0067054	0.87942
Q9CQ92	66437	0.0464302	0.003484	0.9367	0.0433969	0.00167	0.984	0.03809457	0.001414	0.98241	0.03462467	0.0020042	0.96135
O55023	NA	0.2382172	0.109162	0.2538	0.2308144	0.064157	0.4472	0.18917954	0.110682	0.16301	0.10151582	0.0176391	0.73405
Q9Z126	56744	1.3647866	0.277472	0.8287	0.51217	0.32484	0.3321	0.82314004	0.232221	0.61098	0.26917588	0.2621558	0.1309
Q9DBG5	66905	0.4393193	0.051712	0.7427	0.4742659	0.073424	0.6652	0.23581529	0.035513	0.64755	0.53372697	0.0632607	0.80722
P20029	14828	0.3994073	0.038889	0.5396	0.4168218	0.034096	0.6164	0.24376164	0.029117	0.40492	0.34268149	0.0413339	0.45299
P50171	14979	0.3687167	0.710452	0.0511	0.115085	0.018029	0.8907	0.0713023	0.034677	0.58494	0.08288468	0.0081752	0.97164
P01867	NA	0.0751339	0.006877	0.7537	0.2378914	0.041433	0.4518	0.10436109	0.03329	0.19723	0.15154734	0.0434369	0.25268
Q8C5Q4	231413	0.2981534	0.045199	0.9355	0.3797285	0.019887	0.9812	0.24052469	0.025577	0.92665	0.5142624	0.1066409	0.85324
P09528	14319	0.1648095	0.007221	0.8376	0.1892314	0.023135	0.4404	0.12399536	0.014162	0.44143	0.15055839	0.0125368	0.63472
Q8BZF8	226041	0.130492	0.10344	0.1171	0.1755162	0.133115	0.1265	0.20292662	0.082079	0.30391	0.12830537	0.033941	0.61357
P10605	13030	0.6921385	0.147136	0.6299	0.5014685	0.100843	0.6733	0.44720861	0.12452	0.51805	0.22579359	0.0994938	0.3189
P50136	NA	0.2912973	0.039094	0.6417	0.3899983	0.049252	0.6764	0.12137739	0.004544	0.95196	0.1969099	0.0090947	0.94174

Q52KG5	668303	0.1733414	0.144652	0.1069	0.2651771	0.188947	0.1519	0.22119279	0.1321	0.20311	0.22038225	0.1847662	0.11452
Q00915	19659	0.9479427	0.739457	0.4511	0.9963563	0.168604	0.8136	0.48356466	0.178315	0.51234	0.65402566	0.2515211	0.49133
Q9Z2I8	20917	0.1020831	0.017134	0.3272	0.0879605	0.004473	0.8542	0.04025376	0.001774	0.85257	0.06334374	0.0026007	0.89581
Q9DAK9	75454	0.0535301	0.00173	0.9856	0.0499041	0.00155	0.9886	0.0351125	0.001581	0.97239	0.04343871	0.0019285	0.9769
B1AR69	NA	0.077511	0.008848	0.6868	0.0784573	0.014899	0.4888	0.0619135	0.008672	0.58607	0.07725103	0.0111175	0.63295
P70168	16211	0.1507229	0.00549	0.9779	0.1542482	0.006685	0.9708	0.10575491	0.005466	0.9517	0.13646631	0.0050343	0.98657
P48758	12408	0.0607951	0.00201	0.9849	0.0653844	0.001658	0.9923	0.04710782	0.001916	0.97736	0.05752597	0.0021677	0.98325
Q3V118	NA	0.0141352	0.006754	0.2848	0.0127029	0.003155	0.5747	0.01288824	0.005798	0.30997	0.01665891	0.0110686	0.24448
P02089	NA	0.0576707	0.082375	0.0338	0.0226292	0.116972	0.0034	0.15984188	0.187247	0.04948	0.09650722	0.1077286	0.06268
O09061	19170	0.131351	0.012716	0.9303	0.1430097	0.039728	0.6493	0.08967186	0.002919	0.98951	0.09509774	0.0057701	0.98908
P18525	NA	0.1715884	0.072948	0.201	0.5003969	0.125237	0.3995	0.29349089	0.102502	0.30143	0.35339733	0.102115	0.37455
Q91ZD1	107587	0.0009164	0.00034	0.3413	0.0023032	0.000853	0.3778	0.00213079	0.000645	0.43785	0.00992757	0.0064901	0.16317
P62897	672195	0.1231223	0.033743	0.1768	0.0926383	0.032883	0.1281	0.02045106	0.001092	0.84557	0.06114154	0.024268	0.10518
P00329	11522	0.4715186	0.118326	0.5696	0.5004256	0.14223	0.4521	0.44589729	0.096928	0.56946	0.41808656	0.1222268	0.51543
XP_0014794	NA	0.0056577	0.016175	0.011	0.0026055	0.002699	0.1043	0.01482106	0.007503	0.32785	0.01210251	0.0082776	0.21087
Q6P924	NA	0.0036779	0.003838	0.0841	0.0037771	0.003034	0.1469	0.00992322	0.002489	0.56981	0.06738107	0.0335465	0.25161
P97807	14194	0.0292944	0.005091	0.1244	0.0233562	0.000771	0.8202	0.01961629	0.001461	0.43196	0.02932004	0.005837	0.10865
Q9CZ30	67059	0.0781678	0.001005	0.9952	0.0786307	0.001466	0.9917	0.06012013	0.001336	0.98588	0.06709399	0.0026308	0.96441
P09671	20656	0.0289834	0.001156	0.8712	0.0916041	0.029279	0.109	0.02234555	0.000982	0.84484	0.02341814	0.0007651	0.92041
Q91ZJ0	71711	0.0104032	0.000616	0.9597	0.0100652	0.001031	0.8965	0.00820506	0.00061	0.93292	0.00705941	0.0017067	0.58775
P38647	NA	0.0822854	0.001725	0.9003	0.087489	0.005681	0.52	0.076316	0.007489	0.29265	0.07699213	0.0064406	0.39162
Q9D967	67881	0.0717057	0.004678	0.9792	0.0873641	0.006752	0.971	0.05056586	0.005042	0.93494	0.04557988	0.004816	0.96759
P62932	207215	0.0070912	0.001424	0.8921	0.0105651	0.006539	0.2717	0.01922374	0.004549	0.8993	0.00762197	0.0056502	0.37756
OTTMUSP0(	NA	0.0061766	3.13E-05	1	0.0178068	0.002228	0.9697	0.01273722	0.002873	0.90766	#VALUE!	NA	NA
P62869	NA	0.9468381	0.420171	0.7174	1.9542974	1.19853	0.4699	0.98736304	0.71131	0.65833	1.68560749	0.6567856	0.62217
Q91V61	94280	0.0790774	0.008143	0.9895	0.0211719	0.006643	0.772	0.04056328	0.019065	0.5309	0.0678054	0.0225283	0.69369
Q8C4V4	50789	#VALUE!	NA	NA	0.0717956	0.011462	0.9515	0.02649425	0.007062	0.8756	0.05532425	0.0080121	0.9408
ENSMUSP0(	NA	0.1290653	0.061037	0.5278	0.1505758	0.040953	0.8184	0.11954794	0.032665	0.93053	#VALUE!	NA	NA
Q8VFZ7	NA	#VALUE!	NA	NA	0.0004043	0.000488	0.4074	0.00134694	0.003502	0.12889	#VALUE!	NA	NA

STAB 1 (continued) Slope ratio

UniProtKB	Entrez	OCL/YCL			OCR/OCL			ORP/OCL		
		ratio	unadj p	q	ratio	unadj p	q	ratio	unadj p	q
P06151	16828	1.01827	0.06176543	0.14825168	0.78198631	7.60E-20	5.36E-18	0.82860636	6.76E-19	2.47E-16
P21550	13808	0.931006	0.0032804	0.03212917	0.76791262	1.13E-12	1.71E-11	0.77348481	7.47E-14	1.36E-11
P70349	15254	0.989209	0.67009399	0.43991358	0.72543434	3.78E-16	1.60E-14	0.86501778	9.83E-07	2.39E-05
P09411	18655	0.974629	0.15220786	0.23116332	0.7276129	1.64E-20	1.73E-18	0.80390304	9.91E-12	9.03E-10
Q9CZ13	22273	0.982532	0.70760901	0.44920077	0.77980639	3.24E-08	1.96E-07	0.77110467	4.12E-09	1.88E-07
Q9CY10	NA	1.632559	2.37E-07	1.90E-05	0.81932084	0.01217882	0.00903664	0.77341912	0.00016502	0.00122811
OTTMUSF	NA	0.995652	0.55097458	0.39400115	0.71977664	5.89E-09	4.15E-08	0.80548723	3.37E-06	6.47E-05
P06745	14751	0.99226	0.54563311	0.39142046	0.74635651	1.34E-12	1.89E-11	0.79729627	7.39E-10	4.49E-08
P48771	12866	0.926814	0.00917721	0.05306417	0.54053285	7.42E-15	2.24E-13	0.85173193	0.00228272	0.00832412
Q9Z2I9	20916	0.937999	0.38630695	0.34254523	0.74381158	1.25E-10	1.39E-09	0.81674316	3.69E-05	0.00044853
B2RXT3	NA	1.309422	0.02391762	0.09109957	0.61600632	0.00229128	0.0024976	0.65905818	0.00333116	0.01094357
P02088	NA	1.505683	3.75E-11	1.80E-08	0.85375486	0.03019376	0.01808795	0.82750441	3.02E-06	6.12E-05
P59511	223838	0.979808	0.88344165	0.50473976	0.7368048	3.94E-05	9.58E-05	0.78971918	0.00103286	0.00468277
Q8BMS1	97212	0.946592	0.99923958	0.53283905	0.79338947	0.001105	0.00139091	1.06498127	0.63727922	0.30457287
P99028	66576	1.062765	0.02855723	0.10304653	0.72673511	6.28E-12	8.30E-11	0.75307627	2.82E-11	2.06E-09
P70695	14120	0.993473	0.77676281	0.4715723	0.73732628	1.71E-07	9.04E-07	0.84063872	0.01314481	0.02536171
Q5FW75	NA	0.967885	0.05524831	0.13940084	0.74797953	1.03E-08	6.81E-08	0.77130538	2.87E-06	6.12E-05
A2CEK3	NA	1.015276	0.63739081	0.42842669	0.73021757	5.17E-05	0.00011631	0.80489962	0.00496671	0.01331186
XP_00147	NA	0.968989	0.24625622	0.28725945	0.76033432	0.0016821	0.00195446	2.85948456	0.47520428	0.25158037
Q9R0X4	56360	0.348885	0.0174878	0.07561032	1.59642894	0.2078353	0.07325124	1.1611816	0.16338709	0.1225935
Q8BMF3	109264	0.949761	0.84521155	0.49070731	0.77635587	0.50639857	0.14035079	0.80958174	0.54426806	0.27779438
Q9QYR9	171210	1.437624	0.13006688	0.21315196	0.53062121	0.00249462	0.00270531	0.55149192	0.05968923	0.0661371
Q9CQA3	67680	0.939011	0.77724125	0.4715723	0.76482671	0.11077811	0.04698474	0.8032711	0.19697222	0.1386632
Q8BKZ9	27402	0.949055	0.2694339	0.29725687	0.80854367	0.00099349	0.00130441	0.73934268	2.91E-07	8.16E-06
Q91VR2	11949	0.903972	0.13030907	0.21315196	0.83195789	0.00146643	0.00174217	0.83334433	0.0006489	0.00347981
P26443	14661	0.980583	0.2272141	0.27329476	0.87608059	0.11049176	0.04698474	0.7952967	3.14E-05	0.00044029
Q9D1H9	76293									
Q8JZL0	68910	10.58888	0.14491499	0.2258797	0.11000166	0.12359677	0.05026327	0.2511628	0.18289808	0.13229681
Q9CQZ5	67130	0.953156	0.00746747	0.04886048	0.74343702	1.34E-06	5.25E-06	0.81347045	6.63E-05	0.00073263
P53395	13171	0.891426	0.45958039	0.360917	0.74824089	0.00027141	0.00045192	0.82301512	0.00277494	0.00954627
P14869	11837	0.969881	0.21695944	0.26845178	0.66216525	0.00031546	0.00050537	0.60406564	9.76E-05	0.0009366
Q9D0K2	67041	0.919942	0.95016251	0.5249734	0.63667263	0.00137677	0.0016927	0.87390005	0.15318657	0.11750543
P05202	14719	0.934045	0.69900804	0.44667703	1.01593125	0.75608555	0.1901796	1.10882262	0.98897625	0.40115496
P14602	15507	0.914065	0.08039341	0.16837505	0.69183844	1.78E-10	1.71E-09	0.78080414	1.09E-06	2.48E-05
Q9R062	27357	1.388602	0.3975223	0.34332691	0.44934143	0.11408357	0.04765564	0.92401084	0.6668093	0.3137516
Q99K24	106947	1.021842	0.00342794	0.03234632	0.8876708	0.00356194	0.00360402	0.95380144	0.03733734	0.04845327
Q9CQ62	67460	0.841991	0.399184	0.34332691	0.76274042	1.20E-05	3.57E-05	0.95861283	0.14000774	0.11026992
P23927	12955	0.733771	0.00053949	0.01078808	0.61183314	1.70E-06	6.46E-06	0.68473458	0.00024681	0.0016667
Q6P9Q7	NA	0.974814	0.19985757	0.26064038	0.7288306	1.27E-05	3.63E-05	0.84011943	0.02341272	0.03527951

O35658	NA	0.847206	0.15606504	0.23332943	0.8695711	0.00280969	0.00298574	0.89872775	0.02632588	0.0382468
Q3U132	67596	0.891146	0.47505413	0.36303825	0.85599788	0.00192114	0.00216097	0.84658184	0.00396805	0.0120721
Q8K1M6	74006	0.7804	0.15484185	0.23295208	1.07532106	0.58770687	0.15670867	0.95019618	0.61741452	0.30059449
Q9Z2U1	26442	1.110044	0.28633049	0.30536834	0.6664207	0.00039741	0.00062252	0.68717811	0.00086048	0.00444205
Q1XH17	434246	0.898704	0.01663424	0.07323952	0.99379394	0.99389913	0.23431322	0.89558577	0.09641956	0.08901312
B1ATE2	NA	0.790049	0.03151875	0.10709017	1.97570719	0.02519312	0.01566931	1.04185776	0.10381719	0.09144394
P62806	1E+08	1.213936	0.0013836	0.01823938	0.90605412	1.24E-05	3.59E-05	1.15957394	0.04329255	0.05315483
Q9WUR2	23986	0.938853	0.29431563	0.30856279	0.75447666	0.00103658	0.0013233	0.84623546	0.00282461	0.00962632
Q9CQ75	17991	1.00246	0.88099642	0.50394264	0.73602267	0.40588449	0.11888093	0.76856383	0.46088564	0.24715549
Q8VDQ1	77219	0.884734	0.19294799	0.25830548	0.65829688	0.00973506	0.00765303	1.02021231	0.84612993	0.36644683
P16045	16852	0.963877	0.23456047	0.27933072	0.7330259	0.00542558	0.00496685	0.88094483	0.11212603	0.09668315
P47738	11669	0.856531	1.93E-06	0.00011578	0.75732174	1.54E-10	1.63E-09	0.98446023	0.8949264	0.37727429
Q3ULL9	NA	0.859855	0.08027137	0.16837505	0.6506514	0.0008858	0.00117075	0.80776865	0.43817489	0.2402768
Q80Y62	NA	0.952866	0.07364719	0.16173938	0.90375694	0.09732545	0.04305717	0.82960492	0.00493329	0.01331186
Q8CI94	110078	0.765184	0.00428689	0.0373887	0.60623313	1.22E-07	6.79E-07	0.67904391	0.00181666	0.00712321
O08997	11927	0.962065	0.78695083	0.47422972	0.65170445	9.91E-10	8.38E-09	0.84532588	0.00772937	0.01761612
Q64737	14450	1.026117	0.79357539	0.47547162	0.77277389	0.00667805	0.00574066	0.84174843	0.03387303	0.04626247
P68368	22145	0.948765	0.07343669	0.16173938	0.82424749	0.45571458	0.12900877	0.971102	0.92778986	0.38664874
Q3V1I7	NA	0.784279	0.68951954	0.44358482	1.66144767	0.81408898	0.20114334	2.81148668	0.85068395	0.36754608
Q3TEI4	74211	0.998342	0.09080318	0.18077101	0.86778228	0.03667155	0.02107309	0.83134757	0.02101589	0.03437909
Q80SU7	1E+08	0.913815	0.06989008	0.1567367	0.6168717	0.00399208	0.00393577	0.68247835	0.01851911	0.03168029
Q6PHN9	77407	0.979635	0.38747199	0.34254523	0.79691007	0.00046925	0.00070737	0.86115011	0.00058526	0.00323362
Q8BVI5	228960	0.84147	0.11279493	0.19928419	0.97178073	0.42267741	0.12311737	1.24104793	0.96766145	0.39558934
Q8BFS6	223978	0.973487	0.03559822	0.11419116	0.91159241	0.00179999	0.00204647	0.85018496	0.0006968	0.0036825
P17183	13807	0.903412	0.07147954	0.15937033	0.80744352	0.03310935	0.01939503	0.75586922	0.01821846	0.03148583
Q9WUZ7	50795	0.964139	0.62644938	0.426271	0.45917335	1.67E-07	9.04E-07	0.80899373	0.03841899	0.04898531
Q3UHC8	NA	0.992224	0.00343737	0.03234632	0.90736333	0.00029699	0.00049066	0.97883968	0.05550064	0.06285331
Q3UGR5	76987	0.993888	0.30721756	0.3148666	0.72080957	1.76E-07	9.08E-07	0.8071011	8.64E-06	0.00015003
ENSMUSF	NA	1.005858	0.00249314	0.02601104	0.91014509	0.00070471	0.0009935	0.95977757	0.03303318	0.04562811
Q99KR7	105675	0.86579	0.30986374	0.3148666	0.75179764	0.01841749	0.01260431	0.83605544	0.33783459	0.2026495
Q3LAC4	109294	0.663621	0.51603086	0.38159257	2.14982881	0.44848731	0.12781834	0.78351133	0.7280232	0.33060976
Q9D3D9	66043	0.934911	0.14696199	0.22751617	0.79042576	0.03444083	0.01997957	0.77124626	0.00015584	0.0011929
O55143	11938	0.892209	0.30520982	0.31432687	0.71630215	0.00068794	0.00098297	0.86610731	0.01886555	0.03168029
Q9CQ60	66171	1.026501	0.6610147	0.43877487	0.74900393	0.00403734	0.00393577	0.81424034	0.00104016	0.00468277
Q922U2	110308	0.955755	0.11460749	0.19928419	1.57308569	0.15768013	0.06018858	1.00237047	0.0056903	0.01471643
P68037	1E+08	0.965354	0.47003648	0.362331	0.79494816	5.26E-05	0.00011709	0.96213907	0.04198106	0.05227512
P19157	14870	1.004651	0.42764333	0.35022974	0.71072413	0.00321242	0.00333003	0.82579343	0.09760622	0.08965468
P99029	54683	0.94506	1.31E-07	1.26E-05	0.78364259	9.22E-07	3.98E-06	0.82946658	3.52E-05	0.00044853
P35486	18597	0.901788	5.83E-08	6.99E-06	0.78392645	1.63E-10	1.64E-09	0.81673571	1.91E-08	6.96E-07
P56380	66401	0.769149	0.20098528	0.26140074	0.81937938	0.07299021	0.0347639	0.83230599	0.00236104	0.00852447
Q9WUB3	19309	0.942172	0.95681468	0.5249734	0.60195724	4.53E-16	1.60E-14	0.67786258	9.08E-08	2.76E-06
P04247	17189	1.047345	0.44441261	0.35698742	0.7491535	0.08843538	0.04030464	0.85276933	0.23990796	0.15993493

P63325	67097	0.859117	0.04201107	0.12293877	0.71363182	0.00292981	0.00309783	0.67799043	5.67E-06	0.00010338
Q8CE68	NA	0.934307	0.8953435	0.5091186	0.62415943	6.56E-05	0.0001384	0.80911537	0.16720383	0.12443316
P11352	NA	1.421764	3.13E-06	0.00016691	0.7032991	0.00011735	0.00022357	0.91948679	0.00240714	0.00859708
Q91V92	104112	1.066077	0.00538058	0.04098806	0.94173391	0.00538551	0.00495161	1.1083757	0.17399652	0.12740815
P30285	640611	0.944981	0.09476748	0.18192326	0.83965012	0.19868428	0.07097228	0.70575719	0.13535447	0.10776882
Q9CQH3	66046	0.899561	0.06105241	0.14825168	0.79110113	0.00041255	0.00064148	0.83866188	0.04301645	0.05299427
P51174	NA	0.912762	0.26927374	0.29725687	0.82262732	0.0733892	0.03487539	0.79592515	0.02605217	0.03800056
Q9ESN3	60455	0.896438	0.09279102	0.18097356	0.86675015	0.14998169	0.05808882	0.8589127	0.16230516	0.12253812
P00397	NA	1.027543	0.00183738	0.02213162	0.78902592	0.00108523	0.0013742	0.8391623	0.00125347	0.00544243
P17742	268373	1.009397	0.39504399	0.34332691	0.72347966	1.09E-06	4.47E-06	0.9117673	0.00911975	0.01979519
OTTMUSP	NA	0.72603	0.74297775	0.45870601	0.65769755	0.00590691	0.00529292	0.73322818	0.00440541	0.01281746
P97443	12180	1.215882	0.71931297	0.4530351	0.85605377	0.03811751	0.02149513	0.74172841	0.70034241	0.32409341
Q9ERS2	67184	1.135025	0.34135906	0.32765014	0.81328879	0.37707695	0.11420037	0.73214045	0.28100914	0.1772877
O88685	19182	0.980003	0.40591832	0.3434027	1.01329933	0.48722609	0.13653267	0.83385133	0.03269416	0.04550456
Q8VE95	223665	0.831356	0.54210205	0.39128579	0.73641745	0.00044676	0.00067968	0.89990742	0.02131023	0.03437909
O35683	54405	1.184654	0.00029274	0.00702452	0.76357449	0.00785878	0.0064182	0.75900195	0.00929077	0.02004708
Q08AU7	68515	1.136585	0.06322915	0.15025719	1.11102595	0.00445342	0.00430028	1.03328062	0.02140101	0.03437909
B0QZL1	NA	0.970596	0.04628914	0.12694927	0.7384755	4.34E-06	1.53E-05	0.80051893	0.00033599	0.00211242
P17950	NA	0.772108	0.61884843	0.42411121	0.82373593	0.27653485	0.09080524	0.98080302	0.18202197	0.13195985
XP_00147	NA	3.055349	0.06987954	0.1567367	0.44434533	0.17535326	0.06482832	0.61131027	0.33745974	0.2026495
XP_00147	NA	0.964336	0.02483942	0.0931323	1.20073424	1.94E-05	5.26E-05	0.82828213	0.00837793	0.01874282
P50544	11370	0.861533	0.16265694	0.23929985	0.7356094	0.01389095	0.01002562	0.78332094	0.00136473	0.00572021
Q60994	NA	1.856618	0.1955635	0.25837999	0.37724284	0.06305797	0.03090335	0.34816958	0.06390437	0.06883911
Q5DU05	214552	0.859529	0.13057732	0.21315196	1.17357869	0.01759366	0.01219841	1.40456958	0.2788674	0.17654737
P09542	17897	1.223956	0.40625123	0.3434027	0.68824697	0.19557529	0.07009853	0.63896223	0.13051422	0.10623456
Q91Y97	230163	1.817404	0.83798148	0.48865631	0.38602793	0.42971463	0.12449848	0.73139154	0.99500998	0.40315396
Q9JJ26	54483	1.017783	0.02812521	0.10303705	0.84242083	0.00276404	0.00295207	0.84102494	0.01362726	0.02579947
Q4ACU6	58234	1.423529	0.00488803	0.03863107	0.68553029	0.00201611	0.00224392	0.74753479	0.05342251	0.06140658
Q8BIJ6	636969	1.107756	0.17857813	0.25371161	0.75684661	0.21872278	0.07570067	0.67325541	0.40379686	0.22864572
P07309	22139	0.887454	0.10818351	0.19666454	0.91820831	0.81515381	0.20114334	1.0778284	0.06123071	0.06725386
O70468	NA	0.866213	0.53659028	0.38968367	0.7577023	0.16769175	0.06276394	0.90876927	0.75250995	0.33835873
P00920	12349	2.472988	0.06622429	0.1521549	0.83902998	0.50560079	0.14031358	0.55233892	0.14647045	0.11412747
Q9JHI5	56357	0.889847	0.01610849	0.07158135	1.14033539	0.35686119	0.10952847	0.8938931	0.04585172	0.05554885
Q05512-2	NA	0.986986	0.0651646	0.1521549	0.73754429	2.43E-05	6.34E-05	0.87673735	0.02152462	0.034426
P02535	NA	3.281525	0.43328113	0.35242814	0.60049774	0.13550251	0.05366029	0.44434756	0.24310421	0.16118172
P62855	1E+08	1.771201	0.26265658	0.29725687	0.43189036	0.14205551	0.05583708	0.24029168	0.01232601	0.02442815
Q3V3T4	NA	0.848706	0.12149771	0.20750602	0.82669015	0.93429054	0.22375264	0.80507065	0.01549382	0.02783225
Q9QXX4	50799	1.024695	0.51034948	0.37839513	0.7274376	2.85E-05	7.35E-05	0.77342669	0.00131539	0.00557752
P70404	15929	0.806559	0.01205812	0.06156313	0.75650745	1.77E-06	6.57E-06	0.84204097	0.00225966	0.00832412
Q07417	11409	1.064322	0.29051379	0.30845885	0.65177933	0.06536632	0.03170403	0.73908648	0.13471868	0.10765825
P04104	16678	0.895724	0.6659923	0.4396466	1.88091433	0.56012336	0.15108263	1.09224297	0.02502854	0.03695086
P09541	NA	1.00861	0.40474859	0.3434027	0.81609972	0.18139632	0.06646434	1.28704677	0.62969224	0.30253287

Q9D0M3	66445	0.978348	0.29358936	0.30856279	0.74720551	1.71E-06	6.46E-06	0.76350468	1.63E-08	6.60E-07
A2AQP0	668940	1.056775	0.04080351	0.12162997	1.7057564	0.00817485	0.00653521	0.93562901	0.08975868	0.08435886
Q61171	1E+08	1.096488	0.01465926	0.06724555	0.79612167	1.17E-06	4.67E-06	0.86193078	5.48E-08	1.82E-06
XP_20749	NA	1.973733	0.44662183	0.35729428	0.58177394	0.11020982	0.04698474	0.64771139	0.05002973	0.05885081
P48036	11747	1.082311	0.05124934	0.131763	0.64310281	1.65E-18	8.72E-17	0.92201727	0.00174969	0.00693521
XP_90496	NA	0.994183	0.08069314	0.16837505	0.72006793	3.76E-05	9.25E-05	0.82104341	0.07630122	0.07581435
P31786	13167	1.002094	0.38840388	0.34254523	0.68011232	8.94E-06	2.78E-05	0.84864649	0.00360245	0.01144324
Q9CQJ8	66218	0.927287	0.35897329	0.33780097	0.70104487	0.00014896	0.00027156	0.8145712	0.00149932	0.00614314
P18872	14681	1.278634	3.77E-05	0.0013095	1.67322759	0.00818952	0.00653521	1.10506619	0.00011409	0.00104011
O70456	55948	2.823301	0.50594488	0.37703899	0.26011221	0.19490991	0.06997865	0.19093343	0.45511851	0.24590201
Q9CRB9	66075	0.931882	0.12003134	0.20587887	0.94666679	0.01103989	0.00839782	0.82961511	0.00089742	0.00444205
Q9R244	22064	0.615036	0.86151453	0.49515944	1.74759379	0.94837689	0.22584731	1.11977059	0.67700875	0.31650872
P62962	18643	1.000861	0.7703923	0.46919637	0.82062932	0.03270557	0.01925943	0.92308108	0.00461779	0.01288218
Q9DCV4	66302	1.142397	0.00765611	0.04898584	0.8901935	0.03769893	0.02131593	1.11855921	0.00950636	0.02020184
P23953	13884	0.925453	0.03521021	0.11417627	0.80794309	0.17227835	0.06380306	0.90375471	0.00664161	0.01625448
Q06770	12401	1.016159	0.01182637	0.0612281	0.99476288	0.5883918	0.15670867	0.90342913	0.00014297	0.0011468
Q8BH95	93747	1.002821	0.09742653	0.18481007	0.76222771	0.0214211	0.01413113	0.792796	0.01816797	0.03148583
P70398	22284	0.5348	0.50825925	0.37817647	1.02482261	0.00359317	0.0036183	1.22087972	0.40797647	0.23065422
Q8K183	216134	0.974082	0.79971164	0.47775976	0.72527827	7.30E-05	0.00014844	0.79701907	0.00629872	0.01573205
P03903	17720	1.438419	0.00064478	0.01190159	0.83805448	0.03645819	0.02100758	0.76762692	0.00144481	0.00598708
Q8CDU4-2	NA	0.377383	0.30438173	0.31432687	1.54635842	0.1396206	0.05518774	2.35439393	0.28312266	0.17800518
Q9DB20	1E+08	1.797868	0.18515489	0.25514656	0.42780493	0.1119864	0.04723	0.40105567	0.11878135	0.09980321
Q9DCX2	71679	0.937656	0.07492031	0.16265394	0.92017819	0.33406815	0.10427085	0.63070549	0.08925924	0.08410624
Q9D023	70456	0.937823	0.99209955	0.53080101	0.76863019	0.00074257	0.00103309	0.83927933	0.0224777	0.03480531
O08677	16644	1.054229	1.17E-05	0.00051046	0.80203158	0.18684166	0.06809038	0.97477817	0.00116416	0.00517708
Q8QZS1	227095	0.917699	0.01419726	0.06679951	0.77756174	2.15E-05	5.68E-05	0.79278092	0.00089198	0.00444205
XP_48473	NA	1.084819	0.64208368	0.43024953	0.74634728	0.05994739	0.02989864	0.77267055	0.13740079	0.10907206
Q9CQQ7	11950	0.962079	0.94354661	0.52410529	0.91579683	0.18166432	0.06646434	0.80476886	0.0001206	0.00107266
P18242	13033	0.445634	0.00014047	0.00354813	0.65618896	6.46E-06	2.13E-05	0.96113548	0.55573882	0.2806639
P53810	18738	1.019495	0.31892993	0.31658366	0.70686732	7.43E-05	0.00014964	0.86920082	0.00709936	0.01681066
P56716	19888	0.881571	0.05459347	0.138627	0.86936768	0.03448515	0.01997957	1.28876478	0.01594047	0.02835524
Q8BZ25	244859	0.714241	0.12717985	0.21119779	2.21733355	0.79044263	0.19665199	1.96008896	0.35676696	0.21011116
Q3TC72	NA	2.473986	0.39584572	0.34332691	0.30021593	0.3501102	0.10796617	0.35621156	0.41119768	0.23106302
B2RXC6	NA	0.659016	0.31095396	0.3148666	1.45852954	0.7554423	0.1901796	1.51068396	0.96311855	0.39461695
P12979	17928	0.961592	0.3196933	0.31658366	0.83147283	0.14651309	0.05726985	0.72805827	0.00031685	0.00202705
Q62465	26949	1.197982	0.5536862	0.39425094	3.44299379	0.00602956	0.00535742	1.07821964	0.62021768	0.3015356
Q9CR61	66916	0.941817	0.72214538	0.45317138	0.76682903	0.02740222	0.01660379	0.76050403	0.01503127	0.02740638
Q8VCT4	104158	0.8048	0.21392546	0.26806038	0.62728854	0.0166057	0.01158942	0.67706379	0.00096028	0.00459533
P24472	14860	0.924692	0.01026446	0.05597865	0.7563064	7.44E-07	3.28E-06	0.90662921	0.00024114	0.00165915
Q8BK30	78330	1.057235	0.68939327	0.44358482	0.76813538	0.1683462	0.06286861	0.81100114	0.2678128	0.17193686
Q9DCM2	76263	1.100887	0.33165255	0.32285339	2.27450489	0.06818336	0.03284436	0.79984662	0.00017232	0.00125672
B9EHC7	NA	1.087477	0.06441134	0.1521549	0.7266504	0.00116859	0.00146225	0.81226531	0.00194693	0.00747962

P56399	22225	1.041805	0.08302184	0.17174074	0.70411406	1.46E-09	1.14E-08	0.83887678	3.26E-05	0.00044029
Q9JLT4	NA	1.422789	0.84961377	0.49187809	0.4434172	0.84096682	0.2060701	0.51674246	0.8592339	0.37002896
Q91VM9	74776	0.911796	0.00618135	0.04494777	0.73579359	1.10E-06	4.47E-06	0.84959972	0.10586121	0.09235206
P17563	20341	0.85735	0.21094624	0.2666223	1.06021962	0.59626443	0.15781164	0.75824206	0.00126002	0.00544243
P49722	NA	1.088207	0.00840024	0.05039306	0.81093961	0.00013299	0.00024889	0.80463455	0.00097165	0.00459533
Q99J27	11416	1.018768	0.63194829	0.42716152	0.79144316	0.00061083	0.00089084	0.83609209	0.02704332	0.03882507
P52480-2	18746	0.972735	0.21072136	0.2666223	0.75191132	0.00159118	0.00185903	0.81540689	0.02200668	0.0344417
P17751	21991	0.99499	0.61711373	0.42411121	0.71585347	3.06E-14	5.88E-13	0.82903896	1.02E-05	0.00016907
Q9D172	1E+08	0.919732	0.67549954	0.44167003	0.81389181	3.75E-05	9.25E-05	0.85426291	0.00038815	0.00224669
P14131	1E+08	0.94555	0.22247598	0.27099161	0.67564086	0.01633143	0.01143573	0.74331964	0.0012686	0.00544243
Q00623	NA	1.102687	0.00491018	0.03863107	0.79060907	0.25375659	0.08504228	0.92535663	0.00397531	0.0120721
Q91WD5	226646	1.127052	0.01979032	0.07981322	0.89287326	0.01203869	0.00899062	0.79690328	0.00194858	0.00747962
Q8JZQ2	69597	2.895083	0.10474465	0.19334254	0.33014741	0.12224889	0.04990707	0.07727085	0.00396136	0.0120721
P20152	22352	0.786744	0.97597192	0.52813154	0.80364196	0.02233802	0.01450121	1.16276272	0.84466966	0.36644683
P12382	18641	1.198912	0.01347676	0.06443641	0.70737017	0.0122938	0.00909007	0.76395068	0.00197049	0.00748497
Q99L13	58875	0.668569	0.3376323	0.3260897	0.9149667	0.00087264	0.0011606	1.57411053	0.44364824	0.24218559
P41251	18173	1.030799	0.00138584	0.01823938	1.71787192	0.02311163	0.01476956	1.04597291	0.26917896	0.17251022
Q3U0B3	192970	0.828062	0.38495319	0.34254523	2.38420567	0.22459416	0.0771223	1.40637911	0.72248076	0.32958352
Q7TQS8	NA	1.444526	0.00226457	0.02527466	0.85023339	0.00323033	0.00333227	0.80923882	0.02325136	0.03520747
O55124	NA	0.881242	0.48981825	0.37117961	1.01430622	0.35034629	0.10796617	0.73545929	8.51E-07	2.22E-05
O35963	19338	1.083843	0.74257206	0.45870601	0.66315827	0.03728706	0.02120754	0.72893598	0.08651757	0.08215975
P06728	11808	0.91453	0.28022835	0.30302887	0.84066984	0.57266723	0.15368201	1.14525298	0.57696887	0.2870347
O08709	NA	0.955613	0.42716008	0.35022974	0.95592301	0.07270108	0.03472755	0.92748207	0.2221896	0.15085856
Q9WTP7	56248	1.257814	0.01456576	0.06724555	0.76162021	0.02704876	0.01648408	0.60796568	0.01038943	0.02159509
Q791V5	56428	0.932899	0.53752822	0.38968367	0.72278626	0.00021959	0.00037753	0.65230004	9.35E-05	0.00093234
P14152	17449	0.900103	0.18509619	0.25514656	0.74335681	2.90E-07	1.36E-06	0.95624971	0.44557485	0.24251124
P42125	NA	1.483348	0.14061459	0.22125826	0.46186405	0.21101382	0.07397965	0.42241529	0.19477395	0.13791441
Q6NZQ8	72925	0.73969	0.00068161	0.01211553	0.58350553	0.06102315	0.03020402	0.97637044	0.34024817	0.20273577
O35593	59029	0.946618	0.17198823	0.24712757	0.5695858	0.09524819	0.04240431	0.6845825	0.04026978	0.05116622
Q99LB2	NA	0.947516	0.90799492	0.51277868	1.38902234	0.27797981	0.09085648	0.82896599	0.00695081	0.01658042
Q921G7	66841	1.210551	0.30709745	0.3148666	0.51565831	0.0038494	0.00382174	0.53807952	0.0034078	0.01109537
P40142	21881	0.727358	0.0055316	0.04148011	0.95730151	0.01440918	0.01029424	1.04045828	0.25886199	0.1688661
P12787	12858	0.554995	0.50591888	0.37703899	1.28490555	0.45557009	0.12900877	1.30112144	0.22180689	0.15085856
P47934	12908	1.318584	0.24912753	0.28778108	0.85525206	0.23698455	0.08018386	0.69608241	0.15335005	0.11750543
P13020	227753	0.467247	0.7205547	0.45317138	2.54292789	0.33547693	0.1044816	1.54868196	0.37107747	0.21512949
Q9WVK4	13660	0.889345	0.16959746	0.24590099	0.54054014	4.89E-06	1.70E-05	0.85076515	0.01893911	0.03168029
Q8C2L9	NA	1.324073	0.18487434	0.25514656	0.8542009	0.11425504	0.04765564	2.31463172	0.0614357	0.06727638
P29699	11625	0.936763	0.0903523	0.18067452	0.98876844	0.76043583	0.19098418	0.92466364	0.16311635	0.1225935
Q3UMP2	NA	0.9324	0.54299943	0.39128579	0.63924358	0.00021711	0.00037753	0.88011562	0.07333262	0.07469649
P59997	NA	0.699022	0.01511306	0.06842512	1.73583643	0.00142465	0.00173144	1.29055513	0.22912766	0.15359059
O55103	19153	2.009126	0.00086585	0.01484067	0.47837696	0.00500877	0.00468673	0.44903548	0.00219781	0.00826236
Q8K370	71985	1.052986	0.44918737	0.35815034	0.63210586	0.00076788	0.00105443	0.78451878	0.09907935	0.09004434

Q9CZ42	69225	1.211939	0.08967618	0.18067452	0.73728204	0.04283808	0.02328774	0.65565317	0.0029899	0.00992605
Q80W27	NA	0.949533	0.29446935	0.30856279	0.87563653	0.22492004	0.0771223	0.93325487	0.10066032	0.09085796
P34914	13850	1.003094	0.64415212	0.43055925	0.7537582	2.97E-09	2.17E-08	0.875186	0.00296083	0.00992605
O70251	55949	0.914667	0.31740304	0.31658366	0.67654004	0.00269898	0.00289722	0.73976657	0.00015702	0.0011929
Q924D0	NA	1.5462	0.25988429	0.29555377	0.35168311	0.01629125	0.01143573	0.51385804	0.14211625	0.11144915
P80313	12468	1.123752	0.13283101	0.21424029	0.75003978	0.01207428	0.00899062	0.88053428	0.08908103	0.08410624
Q9D8B4	NA	0.961155	0.47123259	0.362331	0.82692942	0.04016667	0.02253053	0.95604335	0.05837263	0.06549556
Q9CZR8	66399	0.976477	0.68695393	0.44358482	0.67693628	6.24E-10	5.50E-09	0.83283663	0.00013667	0.00113265
Q99LX0	57320	1.046088	0.73092497	0.45671382	0.69750906	5.81E-10	5.34E-09	0.82527795	2.02E-05	0.00032027
Q9CQR4	66834	0.992711	0.62463608	0.42581734	0.72107088	0.00148549	0.00175495	1.00235076	0.88020104	0.37409387
Q8CDI6	320696	1.907803	0.00201824	0.02306173	0.88234332	0.09292106	0.04180837	0.95096361	0.00647935	0.01607311
Q9CPP6	68202	1.140954	0.41247571	0.34376821	0.59087088	0.00260969	0.00281566	0.69807636	0.08011658	0.07790712
P03921	NA	0.954813	0.67930332	0.44225753	0.82622007	0.00014022	0.00026011	0.942509	0.04672545	0.05604875
Q9CRB8	67900	0.904759	0.0196977	0.07981322	0.83750526	0.00062147	0.00090015	0.79895994	7.52E-05	0.00078349
Q91X72	15458	1.241784	0.01471241	0.06724555	0.77796635	0.01119151	0.00848264	1.00184922	0.20943821	0.14602938
Q9QUM9	26443	0.595785	0.05111645	0.131763	0.88038049	0.73191847	0.18514132	0.67808559	0.06264265	0.06798558
P62631	13628	0.949077	0.38764904	0.34254523	0.89835222	0.05261424	0.02700555	0.93840437	0.05353297	0.06140658
Q8CHT0	212647	0.93933	0.31102209	0.3148666	0.77435453	0.03383464	0.01971069	0.75755104	0.00155233	0.00628967
O88441	53375	0.880976	0.46907724	0.362331	0.72791811	0.00015451	0.00027926	0.84020685	0.00421117	0.0125872
Q02053	22201	0.890622	0.47087311	0.362331	0.75667564	6.27E-06	2.10E-05	0.86222723	0.00095154	0.00459533
Q9CPU0	109801	0.552189	0.75546362	0.46237046	1.48601547	0.12547285	0.05071701	1.25245962	0.38274797	0.21979872
P54071	269951	0.921848	0.2476156	0.28725945	0.94114892	0.02151721	0.01413113	0.88060905	0.01885119	0.03168029
P28650	11565	1.574758	0.10174441	0.18999684	0.49613203	0.01149516	0.00868168	0.61029409	0.11292377	0.09711929
Q60759	270076	1.773948	0.11354514	0.19928419	3.40342338	0.27223407	0.08981138	3.20303615	0.69044335	0.32155279
A2AGD7	NA	0.818876	0.18004878	0.25460918	2.06259057	0.9318621	0.22342408	1.44157034	0.39629213	0.22533362
XP_48758	NA	0.915376	0.03168612	0.10709017	0.68756955	0.0192312	0.01297333	1.35069686	0.97517293	0.39717349
P80317	NA	0.856454	0.39178123	0.34332691	0.7376257	0.00799676	0.00647919	0.69123819	0.01475871	0.02704464
P47857	18642	0.85343	0.43859148	0.35436818	0.87531473	0.04097216	0.02274107	0.74748105	0.05984864	0.0661371
P08752	14678	1.321823	0.09818042	0.18518169	0.62422991	0.04504151	0.02421479	1.03047872	0.73474724	0.3328343
P52825	12896	0.903824	0.43650529	0.35386429	0.7621208	0.51990657	0.1422305	0.58221534	0.05415493	0.06171263
Q99LC3	67273	0.996588	0.77330032	0.47037054	0.52798971	0.15635385	0.05998872	0.44540803	0.03779902	0.04887845
Q8R1S0	217707	0.914745	0.47152041	0.362331	0.9425444	0.03137135	0.01853092	0.89487626	0.2019231	0.14132997
P25444	667847	1.04679	0.82918417	0.48648184	0.68256358	0.0001442	0.00026517	0.63651734	0.00035948	0.00211432
Q80YV2	232679	1.325823	0.34506745	0.3305485	0.81278995	0.32376125	0.10218731	1.13736635	0.79264242	0.35206281
P13634	NA	1.182185	0.11138752	0.19928419	1.15635	0.90743051	0.21905652	0.89485865	7.36E-05	0.00078349
P01027	1E+08	0.934693	0.02173486	0.08550081	0.87782401	0.38534707	0.11605552	1.09055864	0.01350163	0.02577739
O88844	15926	2.050358	0.13302966	0.21424029	0.48213175	0.1495668	0.05803442	0.55064341	0.1979175	0.1390602
P35700	18477	1.006341	0.78181703	0.47222911	0.735623	0.00033836	0.00053398	0.85623654	0.07236972	0.07433863
Q60988	NA	0.871183	0.04310821	0.12333913	0.80392102	0.03566009	0.02060385	0.97765309	0.00871936	0.01933764
Q9CPU4	66447	0.979953	0.2241323	0.27163029	1.06602429	0.00179328	0.00204647	1.12301344	0.65298602	0.30942315
Q99LC5	110842	0.744072	0.18607535	0.25514656	0.78411505	0.00203816	0.00225659	0.99334965	0.08563858	0.08153736
P18760	1E+08	1.002449	0.93859178	0.52377796	0.68656733	3.17E-07	1.46E-06	0.90716124	0.02271624	0.03480531

Q9D2G2	78920	0.907478	0.06017599	0.14777092	0.82016483	0.12280129	0.05003599	0.76595888	0.11362288	0.09726178
P62245	1E+08	1.522359	0.04168322	0.12272769	0.47393616	0.11038286	0.04698474	0.90728848	0.89191838	0.37706095
Q9D7B6	NA	1.275943	0.03216023	0.10793245	0.86946942	0.01306178	0.00954653	1.35426791	0.06399542	0.06883911
O55143-2	11938	0.795948	0.92934237	0.52128053	0.7265634	0.02189775	0.01433653	0.95206923	0.16791226	0.12470588
Q8K1Z0	67914	1.036788	0.59372542	0.41476092	0.71125217	4.53E-08	2.66E-07	0.83917971	0.00952869	0.02020184
Q99MN9	66904	0.849606	0.97708413	0.52813154	0.68788764	0.22574789	0.0771223	0.62025134	0.10512181	0.09213475
A2AR50	241308	0.848858	0.65935118	0.43827683	1.50321776	0.48117468	0.13513091	0.87516841	0.36514062	0.21304244
Q9DBB8	71755	0.834928	0.3881765	0.34254523	0.32429261	0.0666691	0.03226191	0.71176694	0.07123065	0.0734661
Q9DCT2	624814	0.869537	0.81412855	0.48117814	0.77114302	0.30663837	0.09780474	0.72272945	0.04528351	0.05504335
Q6NSW3	NA	0.564421	0.36499186	0.34008223	1.85474442	0.18730072	0.06809038	1.73182677	0.56665788	0.28403019
Q9CW46	71766	1.210504	0.18607066	0.25514656	1.27183051	0.0271888	0.01652181	1.17059659	0.15068214	0.11653298
Q9DC61	66865	0.933215	0.41555799	0.3438528	0.6207817	1.76E-05	4.90E-05	0.89148739	0.11353683	0.09726178
OTTMUSF	NA	1.428013	0.31089468	0.3148666	0.68093635	0.22805096	0.07765818	0.9963573	0.12973497	0.10591773
O35129	12034	1.074459	0.13482652	0.21568652	0.7280784	0.01999058	0.01333562	0.84216363	0.03656099	0.0482332
Q9DBP5	66588	0.634884	0.1089326	0.19727904	0.71014505	0.0307205	0.01835152	0.90293887	0.63149604	0.30300029
O88668	433375	2.371551	0.15539123	0.23304804	0.76278599	0.44232431	0.12691713	1.03038437	0.96476862	0.39484938
P34884	17319	1.00087	0.70177199	0.44667703	0.77581892	0.00599317	0.00534756	0.79758416	0.00010291	0.0009622
ENSMUSF	NA	2.708612	0.79659768	0.47668732	1.04451615	0.21362161	0.07446765	0.96243508	0.1398723	0.11026992
Q9Z2K1	16666	1.130291	0.02562249	0.09532363	1.54358767	0.55636798	0.15026135	1.47175229	0.03530855	0.04733659
Q9CPQ8	1E+08	1.021653	0.36706652	0.34008223	0.75561222	0.00452546	0.00434998	0.75418067	0.0239237	0.03585722
Q9CQC7	1E+08	1.169969	0.0366278	0.11419116	0.71142499	0.01825516	0.01253378	0.72537377	0.00715006	0.01682147
Q8JZN5	229211	2.162999	0.06657885	0.1521549	0.2774038	0.62776819	0.16308793	0.55870518	0.54952886	0.27870683
Q9Z2W0	13437	1.087677	0.5220676	0.38367073	0.70941313	0.00021896	0.00037753	0.84039698	0.00272467	0.00946259
Q9CWR0	NA	0.892363	0.05566266	0.13940084	0.79545085	0.05376053	0.02746061	2.04290564	0.29191409	0.18290184
O09161	12373	1.475926	0.00304303	0.0310726	0.67086709	0.25678877	0.08578656	0.50238037	0.05318374	0.06140658
Q8BFZ3	238880	1.093366	0.01298924	0.06426593	0.77683159	0.168566	0.06286861	0.93024967	0.00552841	0.01439987
Q8R0P4	66273	0.760586	0.26904586	0.29725687	0.44672923	0.01926799	0.01297333	1.0887353	0.07625192	0.07581435
Q91VA7	NA	0.841185	0.41024295	0.3436018	0.69129167	5.10E-23	1.08E-20	0.79346798	2.46E-13	2.99E-11
Q3UM45	66385	0.73985	0.04833481	0.12815937	0.71371165	0.07846274	0.0368721	0.77358742	0.0429393	0.05299427
A6H6E2	105450	1.115602	0.48068005	0.36559114	0.69697506	0.04065218	0.02262284	0.86347087	0.15338359	0.11750543
ENSMUSF	NA	0.730805	0.06147124	0.14825168	0.90108645	0.5354021	0.14563704	0.98202955	0.97903207	0.39756357
Q9CQ65	66902	0.967457	0.98115694	0.5290752	0.73440523	0.04567691	0.02433062	0.8466478	0.59960766	0.29507046
P10126	13627	0.879526	0.46521178	0.36127739	0.58630038	0.30958719	0.09830049	0.62639171	0.10306516	0.09144394
Q9DC69	66108	1.198963	0.99792172	0.53272823	0.44846173	0.06494455	0.03157187	0.78207772	0.97685284	0.39717349
Q9ERI6	105014	1.184421	0.01234419	0.06171068	0.69627233	0.04508585	0.02421479	0.77091613	0.05506141	0.06255014
P51667	17906	0.876666	0.00679197	0.0468297	0.7908377	0.03735571	0.02120754	0.86891133	0.05125675	0.05971404
Q61292	16779	1.449934	0.25877391	0.29499001	0.70189821	0.14826339	0.05764587	1.01601494	0.31943622	0.1954447
P20065	19241	1.288923	0.90269901	0.5114798	0.55763406	0.31646339	0.10003323	0.73775464	0.94344107	0.38873846
Q9DBJ1	18648	1.05716	0.51091848	0.37839513	0.70701125	0.00144117	0.00174084	0.79832193	0.01874525	0.03168029
P53986	20501	3.054279	0.11377947	0.19928419	0.23262403	0.09350666	0.04198252	0.2998617	0.15679193	0.11955245
P56565	NA	1.092191	0.00753391	0.04886048	0.65225717	1.07E-09	8.70E-09	0.90015706	0.00227807	0.00832412
Q9CQN6	66154	0.700984	0.7416873	0.45870601	1.39085369	0.4880417	0.13653267	0.90938809	0.01587936	0.02835524

Q9D8T7	380773	0.805548	0.04772875	0.1279664	0.91920045	0.02036185	0.01354057	1.0211612	0.00547462	0.01436233
Q8R164	68021	0.294854	0.58203225	0.4107779	0.65830112	0.00646893	0.00567291	0.73414638	0.2206417	0.1503903
P61014	18821	1.023085	0.63295241	0.4272385	0.51554974	0.39794837	0.11782019	0.68051396	0.70378396	0.32445082
Q9EQI5	NA	0.479661	0.31993467	0.31658366	3.68827926	0.61566905	0.1611324	3.48113914	0.92984389	0.38664874
P56480	11947	0.783229	0.04435616	0.12376402	0.94582853	0.29948424	0.09595707	0.89696951	4.19E-05	0.00047747
P01864	NA	5.574737	0.02191323	0.08550081	0.33434292	0.05546199	0.02816266	0.7806164	0.53112499	0.27355804
O08749	13382	0.551499	0.10343875	0.19190656	0.76531901	0.00047165	0.00070737	1.25075577	0.36468303	0.21304244
Q5EBG6	243912	0.909955	0.57304608	0.4050314	0.5737205	0.00779898	0.00641729	0.63005011	0.00897956	0.01972571
Q62388	11920	1.394061	0.41289727	0.34376821	0.50951046	0.04773528	0.02504848	1.08546567	0.68138597	0.31814724
Q00897	20703	0.980784	0.09273432	0.18097356	0.81557648	0.21559794	0.07498734	0.8265967	0.06051923	0.06667321
Q9DCW4	110826	0.79499	0.00191205	0.02238126	0.90344064	0.33255724	0.10427085	0.87387518	0.0050377	0.01331186
ENSMUSF	NA	1.153385	0.61184788	0.42231146	0.63680585	0.03374185	0.01971069	0.56985327	0.00624207	0.01569809
P56501	22229	1.304521	0.19109786	0.25830548	0.80358069	0.15483574	0.05964112	0.84624858	0.49031941	0.2582078
P05201	14718	1.015439	0.44482088	0.35698742	0.70626365	0.0193248	0.01297333	0.78949394	0.11657393	0.09880284
Q9Z2I0	56384	1.044193	0.39840389	0.34332691	0.88807167	0.11706802	0.04835209	0.71952095	0.00435774	0.01281746
P50446	16687	2.357972	0.00657655	0.046415	1.14823977	0.01154736	0.00869007	1.04796714	0.05371797	0.06140658
P18826	18679	0.231365	0.41498351	0.3438528	0.86393816	0.26733435	0.08874875	1.1871659	0.08559507	0.08153736
Q76MZ3	51792	1.128763	0.89593428	0.5091186	0.44398334	0.00718087	0.00607347	0.56597384	0.04933012	0.05830444
Q9Z2Z6	57279	1.120616	0.12959768	0.21315196	0.62891571	0.00170565	0.00197099	0.77662195	0.00446995	0.01281746
O88799	NA	0.973917	0.98907081	0.53069746	0.88904554	0.1284028	0.05132935	1.16746766	0.50484936	0.2622471
P07759	NA	0.697486	0.36565273	0.34008223	1.41244381	0.35074988	0.10796617	1.46230892	0.22256979	0.15085856
Q6IFX2	68239	2.693713	0.03714681	0.11419116	0.76012138	0.45398784	0.12900877	7.91171052	0.01088895	0.02218293
P35441	NA	0.704422	0.73584693	0.45803855	1.7213449	0.44422483	0.12711462	1.87200422	0.42430734	0.23641878
Q61147	12870	1.328148	3.34E-05	0.0013095	0.85900551	0.44089041	0.12667758	0.98008088	0.00602624	0.01545406
Q60864	20867	1.252148	0.02028812	0.08113896	0.70347863	0.08640011	0.03967836	1.02703106	0.93094734	0.38664874
P62075	30055	0.338475	0.00643026	0.04605988	2.66877448	0.40245008	0.11836684	1.02052211	0.45174264	0.2451364
Q8BVI4	110391	0.649431	0.16736468	0.24413881	0.8093455	0.00064574	0.00092893	0.96489174	0.07034359	0.07287321
P61148	14164	0.997882	0.01869431	0.07801541	0.90581633	0.1156966	0.04791101	0.87683441	0.00737137	0.01712121
Q64727	22330	0.945467	0.79134172	0.47532013	1.10206569	0.38818319	0.11610313	0.82888522	0.15413989	0.11783727
Q8QZT1	110446	0.947512	0.00118934	0.01823938	0.80499239	1.23E-05	3.59E-05	0.82422094	4.15E-05	0.00047747
Q924X2	12895	0.750146	0.94273926	0.52410529	0.65270038	0.00081795	0.00111594	0.73845227	0.02107002	0.03437909
P50752	21956	0.906197	0.03356315	0.11185854	1.18701084	0.0223917	0.01450121	0.904552	0.03580539	0.04782684
O54724	19285	0.874476	0.08852082	0.18001236	0.644858	4.27E-05	0.00010033	0.91799455	0.21973611	0.15011896
Q03265	11946	0.907446	0.58951126	0.41422884	0.81221882	0.01949472	0.01304598	0.84705973	0.03526193	0.04733659
P45952	11364	0.560288	0.33921494	0.32624461	0.92617911	0.2826298	0.09180871	0.84198282	0.39260277	0.2236966
P03976	NA	3.165371	0.40755845	0.3434027	0.8252948	0.02100688	0.01392572	0.73771607	0.24199662	0.16073963
Q99J99	NA	0.876038	0.00440106	0.0373887	0.66931333	4.57E-05	0.0001062	0.86360355	0.04192627	0.05227512
Q6ZQ20	NA	1.2408	0.00806054	0.04959503	3.09059368	0.26791752	0.08880294	0.87854692	0.00730396	0.01707339
P62814	11966	1.463757	0.50315176	0.37671238	0.84857225	0.71693257	0.1820037	0.64108371	0.31284794	0.19250553
Q8BWF0	214579	0.58792	0.02234948	0.08649971	1.237509	0.36664092	0.11188056	1.26714238	0.14083655	0.11068363
P12367	NA	1.196319	0.00980382	0.05535353	0.85178222	0.20709398	0.07311182	5.75714109	0.0777309	0.07651877
Q8R0F8	NA	1.027089	0.38301754	0.34254523	0.65277269	1.02E-06	4.31E-06	0.83029807	0.0268968	0.03878499

P21614	14473	1.168133	0.07380588	0.16173938	0.70868236	0.19163462	0.06903714	1.07289663	0.54683584	0.27850305
Q9WV35	11811	1.075464	0.08174304	0.16982739	0.76480579	0.05566767	0.02816266	0.89386309	0.21065041	0.14659428
Q8R2K3	NA	0.96453	0.51898101	0.38249522	0.84373069	0.22731649	0.07753292	0.75148977	0.00012536	0.00108844
Q8VEK3	51810	0.978754	0.33003609	0.32193283	0.3115992	0.04689808	0.02470221	0.34678959	0.06947716	0.07218068
Q8BW75	109731	0.412703	0.28049251	0.30302887	3.02266992	0.77410347	0.19326914	0.5881012	0.27842502	0.17654737
Q08284	NA	1.037985	0.09866841	0.18518169	0.87814206	0.35755992	0.10958388	0.97052506	0.07983778	0.07790712
Q60597	18293	0.790887	0.40896437	0.3434027	0.87216985	0.02207296	0.01440663	0.94231065	0.30910018	0.19136828
Q99N96	94061	0.805751	0.60955581	0.42213287	0.58327964	6.06E-05	0.00013077	0.96837843	0.23020774	0.15374934
A0JLR7	NA	1.140191	0.42567305	0.35022974	0.823035	0.18249234	0.06665197	0.62733753	0.10458965	0.09190235
P07901	15519	1.08814	0.75629427	0.46237046	1.03523043	0.63446416	0.16442349	0.76455794	0.44542387	0.24251124
Q91Z53	76238	1.124065	0.04039644	0.12162997	0.74748106	9.09E-05	0.00017799	0.80917617	0.00979382	0.02064392
P09103	18453	0.934566	0.16305017	0.23929985	0.59746032	0.09761663	0.04309583	0.51095793	0.00100249	0.00462741
Q3TS19	NA	1.158509	0.11335687	0.19928419	0.64662324	0.00139867	0.00170969	1.11453968	0.87246842	0.37298098
P06537	NA									
P70670	17938	0.970962	0.38731372	0.34254523	0.67645916	0.00902995	0.00715189	0.80870537	0.31582516	0.19356006
Q9JKB1	50933	0.948452	0.54181261	0.39128579	1.32327457	0.8467055	0.20675756	1.16953668	0.50264297	0.2622471
Q9JKS4	24131	1.018488	0.43860365	0.35436818	0.73473451	0.20434685	0.07262696	0.84863052	0.4137687	0.23106302
P56382	67126	0.976055	0.06178182	0.14825168	0.80338544	0.37748333	0.11420037	0.73803607	0.01193283	0.02404091
P15626	14863	0.878493	0.77846408	0.47171786	0.78169654	0.00987987	0.00770954	1.00128879	0.94209651	0.38873846
Q9D0F9	72157	0.925376	0.80415217	0.47932998	0.87549682	0.0459355	0.02440688	0.94845942	0.05770246	0.06494345
Q9CZB0	66052	1.065498	0.1714536	0.24712757	0.78085028	4.64E-05	0.00010665	0.81979873	0.00054661	0.00311445
P03888	NA	0.625508	0.74816159	0.45974107	0.95216742	0.39075426	0.11654789	1.12433768	0.94264075	0.38873846
Q8C0M9	66514	2.028488	0.42691304	0.35022974	0.80174041	0.9863044	0.23304235	1.29862192	0.29087155	0.18256231
Q8CG76	110198	0.923456	0.5678916	0.40257398	0.64725268	0.00550636	0.00499753	0.69336893	0.00827686	0.01863103
Q9Z1J3	NA	0.978327	0.63558212	0.42841099	0.66535309	0.00224968	0.00246497	0.91083442	0.87685594	0.37397979
Q91WK5	NA	2.288538	0.6215892	0.4249475	0.94679946	0.77144459	0.19310196	1.77672415	0.97679798	0.39717349
Q64105	NA	1.065908	0.38057291	0.34254523	0.68537379	6.61E-05	0.0001384	1.13160672	0.97698231	0.39717349
Q6P8J7	76722	0.629365	0.84548802	0.49070731	0.73620922	0.05728878	0.02877626	1.14372855	0.41370999	0.23106302
Q99NB1	68738	0.879565	0.9097318	0.51304179	1.0403215	0.10281058	0.04493752	1.44524479	0.55646628	0.2806639
Q6X7S9	386655	1.221063	4.83E-06	0.0002318	0.79308432	0.00084585	0.00113931	0.68126637	0.00020101	0.00143726
Q8CGK3	74142	1.016526	0.34706191	0.33165807	0.78131102	0.00018204	0.0003208	1.01876821	0.20178198	0.14132997
Q9CPQ3	223696	0.659264	0.81729032	0.4824527	0.9872079	0.53580225	0.14563704	1.08825559	0.49070061	0.2582078
Q8BG32	69077	1.125204	0.26054194	0.29560121	0.68277549	8.36E-06	2.68E-05	0.75763098	0.00014466	0.0011468
Q60605	433688	1.147548	0.00169323	0.02138461	0.66750477	0.00779364	0.00641729	0.85876963	0.16665265	0.1242766
P03893	NA	1.08804	0.74260115	0.45870601	1.45564368	0.79935197	0.19840169	0.82654489	0.94632297	0.38948583
Q62048	18611	1.078728	0.62788578	0.426271	0.64426712	0.61514725	0.1611324	1.10495188	0.59739567	0.29438545
Q9D2J7	319196	0.52706	0.01020217	0.05597865	1.73103804	0.02678358	0.0164171	6.87494058	0.21923517	0.15011896
Q9D892	16434	0.989844	0.27254961	0.29931816	0.6981274	8.41E-05	0.00016621	0.91575328	0.32201674	0.19633841
Q9D6Y9	74185	1.496687	0.0036683	0.03321676	0.5435974	0.00218293	0.00240429	0.88291977	0.53415177	0.27434201
P11499	15516	1.076607	0.95810153	0.5249734	0.72137205	0.11109127	0.04698474	0.60774384	0.01421513	0.02631301
P63101	22631	0.908245	0.94890688	0.5249734	0.77584534	0.00172944	0.00198763	0.90880334	0.0257746	0.03774666
Q04857	12833	0.578794	0.01557279	0.06984763	2.44773676	0.31238586	0.09897379	0.89616345	0.00250148	0.00877101

P70195	19177	1.349188	0.00894593	0.0523577	1.62660012	0.29221698	0.09448756	0.81753451	0.01837612	0.03160851
P03987	NA	1.394673	0.03618789	0.11419116	0.91310492	0.27794765	0.09085648	0.9337504	0.42721564	0.23675941
Q99L47	70356	1.514401	0.38175834	0.34254523	0.84455736	0.29759566	0.09564177	0.60053944	0.22679248	0.15286828
Q7TQ48	106393	1.009934	0.7310982	0.45671382	0.61474545	2.81E-08	1.80E-07	0.8122465	0.25392003	0.16653601
P97450	11957	0.961247	0.04629129	0.12694927	0.67358509	6.82E-09	4.65E-08	0.80503282	2.26E-05	0.00034339
P11438	16783	1.153375	0.62847394	0.426271	0.90902202	0.45843798	0.1294332	1.12702214	0.10315511	0.09144394
P51885	17022	0.69598	0.3258433	0.32012816	0.62453867	0.00071417	0.00100016	1.2209698	0.79784139	0.35265678
P06801	17436	0.974872	0.04394688	0.12333913	1.3968288	0.56759374	0.15270806	0.75103074	0.00937628	0.02011257
Q8R4N0	69634	0.961944	0.50986215	0.37839513	0.70439126	4.05E-05	9.62E-05	0.80603108	0.01391131	0.02614884
Q9EPB5	68607	0.863457	0.85798752	0.49476261	0.96122966	0.57831744	0.15441448	3.61343503	0.31183039	0.19240527
Q61554	NA	0.432518	0.20842456	0.26574358	2.31220447	0.59458747	0.15756501	1.40518248	0.02269062	0.03480531
O70370	13040	1.12732	0.14496361	0.2258797	1.06357418	0.57701635	0.15441448	1.07667442	0.36586255	0.21312266
Q99JY0	1E+08	0.942469	0.36656044	0.34008223	0.70184849	3.04E-05	7.75E-05	0.908626	0.03295773	0.04562811
Q8CI04	NA	1.42917	0.03482825	0.11370596	1.33315775	0.48810298	0.13653267	0.74881845	0.91920005	0.38461363
Q924M7	110119	1.581723	0.26829894	0.29725687	0.47262095	0.06948038	0.03331736	0.52970855	0.03446225	0.04671726
Q9D6Y7	110265	1.591275	0.6927634	0.44507506	0.30455833	0.0623172	0.03064689	1.6472158	0.27504031	0.17503614
Q8BFP9	228026	1.365735	0.06324377	0.15025719	1.63950246	0.85237158	0.2079011	0.60415082	0.00748506	0.01727525
P28271	11428	1.196327	0.28203937	0.30302887	0.67939865	0.1138025	0.04765488	0.7598428	0.29618131	0.18493998
Q9JHW2	52633	1.759459	0.29857972	0.31150957	0.42156638	0.11256453	0.04723	0.4801785	0.02227175	0.03470759
ENSMUSF	NA	0.865923	0.96380303	0.52682054	0.88825409	0.04241165	0.02311535	0.84251951	0.34411157	0.20470328
P00493	15452	0.341023	0.01186492	0.0612281	0.82823776	0.61753173	0.16136723	1.0727041	0.30325045	0.18838632
Q9DCS3	26922	1.121575	0.06794815	0.15381926	0.6337296	2.03E-06	7.40E-06	0.81262023	0.02194126	0.0344417
Q8R0N6	76187	0.510184	0.16255252	0.23929985	0.64043229	7.26E-07	3.27E-06	0.78195631	0.04200265	0.05227512
Q921I1	22041	1.02834	0.00236251	0.02576852	0.91619863	0.78633002	0.19585925	0.9762166	0.3498329	0.20776782
Q8C156	215387	0.413216	0.59597046	0.41572412	3.63958422	0.668789	0.17122059	6.08456294	0.35508977	0.2098645
Q61085	14787	1.002956	0.97720605	0.52813154	0.76125897	0.01503245	0.01063177	0.7730547	0.01875691	0.03168029
P48962	11739	1.111189	0.55481572	0.39446994	0.65317307	0.01726754	0.01201168	0.71722233	0.03175374	0.04436498
P01837	NA	1.826924	0.09034129	0.18067452	0.81632917	0.50854341	0.14074611	1.28977256	0.35381756	0.20945208
P17182	1E+08	1.165552	0.27100906	0.29830892	0.71452161	0.0014661	0.00174217	0.82112469	0.02117453	0.03437909
P45376	11677	0.920283	0.15782054	0.23522126	0.75291132	0.00120455	0.00148962	0.82116054	0.02399277	0.03585722
B2RY56	67039	0.891568	0.24151229	0.2847828	0.67140946	0.0018807	0.00212679	1.10909244	0.92077423	0.38461384
Q8BFR5	233870	0.849327	0.99080021	0.53069746	1.07579112	0.06466847	0.0315101	0.86942104	0.00088737	0.00444205
Q9R0H2	59308	0.892076	0.00247568	0.02601104	0.93436207	0.26854411	0.08887133	0.71314933	0.04288942	0.05299427
Q9CZS1	72535	0.532484	0.83022213	0.48649606	0.66507498	0.14631924	0.05726985	1.10546883	0.10768033	0.09326964
Q9DCM0	66071	1.005294	0.09365908	0.18113176	0.52038982	0.02403826	0.01508412	0.90429231	0.36330204	0.2126502
P47955	665298	0.716569	0.33801718	0.3260897	0.93562825	0.2851854	0.09249678	0.79801003	0.00481276	0.01329554
P63158	637733	1.149739	0.06034984	0.14777092	0.70319381	9.83E-05	0.00018898	0.92906766	0.03681538	0.0482332
Q8K3J1	225887	0.94377	0.35763412	0.33720196	0.9009312	0.10453603	0.04529947	0.68455864	0.00058096	0.00323362
Q64669	18104	0.96637	0.22125254	0.27018712	0.76284961	0.08515938	0.03923437	0.82600641	0.13449802	0.10765825
Q3ULJ0	333433	1.610852	0.18770183	0.25664353	0.37621057	0.03101323	0.0183707	0.57696121	0.24424439	0.16164378
Q9CZU6	12974	1.07404	0.84198669	0.49039603	0.54398755	0.03694026	0.02116591	1.1033526	0.41184493	0.23106302
Q8CGW4	214105	1.230934	0.15908278	0.23563896	1.15162111	0.59067973	0.15712014	0.85332471	0.17219398	0.12685242

Q9QVP4	17898	0.977602	0.28024609	0.30302887	0.85136812	0.36601759	0.11185175	0.65696801	0.07722931	0.07651358
P21107	59069	0.883965	0.02915428	0.10441586	0.50084572	0.00047885	0.00071311	0.63054082	0.09534916	0.08847291
Q8CAQ8	76614	1.199162	0.11451316	0.19928419	1.0194541	0.90282889	0.21844441	0.97285769	0.8576393	0.37002896
P54116	13830	0.643382	0.07605742	0.16295305	0.74931916	0.02150659	0.01413113	0.85059242	0.01638929	0.02887193
O89053	12721	2.811564	0.00353671	0.03264108	0.23045953	0.0091461	0.00721686	0.46155555	0.03721155	0.04845327
P97372	19188	1.230504	0.02184851	0.08550081	0.70052223	4.05E-05	9.62E-05	0.8671522	0.05119016	0.05971404
Q8K480	259172	1.265642	0.40639482	0.3434027	0.75528518	0.36778503	0.11206797	0.51663103	0.0875407	0.08291541
P07724	11657	1.045736	5.48E-05	0.00175331	0.82178734	0.60110419	0.15830008	1.0014249	0.07574922	0.07567829
O35841	11800	1.48742	0.237217	0.28109927	0.61871035	0.04695857	0.02470221	0.59801546	0.07784951	0.07651877
Q9D6X6	76453	1.040672	0.22888637	0.27461792	0.67428276	0.01437675	0.01029424	0.75349379	0.18585537	0.13394009
Q9WVH9	23876	1.040078	0.92052542	0.51791165	0.43073499	0.23536261	0.07976269	1.00620785	0.80008421	0.35278994
Q6LCR2	NA	0.906157	0.00831394	0.05039306	0.75722617	0.01363347	0.00990741	0.79520124	0.07271515	0.07448364
P58774	22004	0.521979	0.19423831	0.25830548	1.95115276	0.85738342	0.20864223	1.92559187	0.88019535	0.37409387
Q3TD78	NA	0.441256	0.13890202	0.22000617	0.84298679	0.24685932	0.08299383	0.86149331	0.02690904	0.03878499
Q9CQX8	66128	0.967803	0.96738185	0.52715185	0.81561625	0.01068483	0.00817956	0.76206494	0.00021779	0.00152732
Q93092	21351	1.006197	0.59338309	0.41476092	0.57383745	0.20503806	0.07275036	0.54703892	0.02138182	0.03437909
Q05920	NA	0.977416	0.9587198	0.5249734	0.70032973	0.14048541	0.05532276	0.57003951	0.06513805	0.06945141
Q02566	17888	1.149362	0.06489297	0.1521549	0.70273316	0.05659782	0.02849688	0.78853876	0.00874987	0.01933764
Q9DCD0	110208	0.987987	0.95433384	0.5249734	0.78472683	0.02372887	0.01495653	0.78533337	0.00496213	0.01331186
Q8BGY7	108654	1.109638	0.19316136	0.25830548	0.85554163	0.08066789	0.03782431	1.96771235	0.13758951	0.10907206
Q3TP92	67181	1.473536	0.14368894	0.2253569	0.32468264	0.04905606	0.02555133	0.52094231	0.16996852	0.12594754
Q8CC88	219189	0.936969	0.12314183	0.20931915	0.72146555	3.84E-06	1.38E-05	0.97624244	0.62702313	0.30164793
P48787	21954	0.730769	0.00866395	0.0513334	0.83990691	0.00044596	0.00067968	0.8612842	0.0002588	0.00171586
Q9R0P5	56431	1.303142	0.01914693	0.07899424	0.8049122	0.05142756	0.02672074	0.83117426	0.43469112	0.2390855
P52850	17423	0.406544	0.80949816	0.47962275	1.76783109	0.31264375	0.09897379	1.08401299	0.04928312	0.05830444
P08003	12304	1.08424	0.2210203	0.27018712	0.532	0.18068952	0.06638652	0.87169959	0.71141591	0.32550019
P61971	668830	1.055574	0.79098017	0.47532013	1.41051633	0.90582196	0.21891812	1.37844349	0.84779394	0.36673194
Q3TMY3	NA	2.437853	0.29988844	0.31219628	0.33468309	0.28898169	0.09358453	0.45478772	0.49733214	0.26058289
Q91V64	66307	1.34115	0.01816515	0.07696397	0.62864577	0.02514742	0.01566931	0.70556041	0.04153742	0.0522309
P14069	20200	1.050929	0.93791657	0.52377796	0.64811517	4.74E-05	0.00010778	0.94847284	0.4715917	0.25068482
Q3UED7	NA	0.569219	0.38181512	0.34254523	1.18488458	0.39836285	0.11782019	1.30151818	0.72408928	0.32964432
Q9CQ91	66091	0.973828	0.74372983	0.45870601	0.71013945	0.00720881	0.00607347	0.8928187	0.12156982	0.10144493
Q9WVL0	14874	0.855029	0.53174468	0.38842459	2.80065535	0.09394361	0.04207602	0.81576906	0.09839114	0.09004434
P11087	12842	6.515952	0.20430319	0.26300491	1.06274461	0.94188341	0.22455382	0.2405014	0.21306087	0.14759372
Q9JI75	18105	1.189809	0.8868868	0.50610558	2.58563874	0.92308107	0.22156995	0.8008905	0.89280935	0.37706095
Q8R2Q4	320806	1.198659	0.19474887	0.25830548	0.67455558	0.04173194	0.02292212	0.87486291	0.07501656	0.07567829
P16546	20740	0.786088	0.21231482	0.2674387	0.98292171	0.51947021	0.1422305	1.27419693	0.81346897	0.35610821
Q61646	15439	1.69371	0.00710482	0.04735761	0.84600552	0.06449079	0.03149609	1.49801045	0.1514114	0.11673026
Q9CQ69	22272	0.88722	6.22E-05	0.00186569	0.78245194	0.22505275	0.0771223	0.80693653	0.19130938	0.13625495
Q9JJZ2	53857	0.834598	0.10641223	0.19492125	0.64586842	0.00488395	0.00461074	0.72670026	0.04806235	0.05746338
Q8VDN2	11928	1.789948	0.22619175	0.27329476	0.44037993	0.21375164	0.07446765	0.5594743	0.35626318	0.21011116
Q78ZA7	17955	0.955146	0.13741644	0.21837387	1.02786237	0.63293562	0.16422862	0.65442568	0.43763188	0.2402768

Q3TLK7	NA	2.275561	0.03101784	0.10709017	1.33586237	0.0420222	0.02296227	0.85629984	0.17723767	0.12952139
P35979	668706	0.842496	0.21620653	0.26845178	0.86744635	0.84641727	0.20675756	0.80421894	0.80966352	0.35529537
P18572	12215	1.0302	0.40845286	0.3434027	0.73337897	0.16507803	0.06232764	0.8699177	0.31373899	0.19260522
P59017	94044	1.098307	0.0004351	0.00931026	1.81381604	0.26556397	0.08829964	0.97025048	0.1894263	0.1359761
XP_00147	NA	1.215471	0.03735624	0.11419116	0.881929	0.01036155	0.00805569	0.89547221	0.18990141	0.13604933
Q60977	NA	1.528224	0.08430891	0.17365467	0.71965259	0.16593658	0.06232764	0.76915321	0.17098682	0.12621811
Q8BGK2	234072	0.93561	0.47323726	0.36264109	0.68795327	3.11E-08	1.93E-07	0.88574695	0.06567229	0.06958327
P43024	12861	1.07193	0.07435915	0.16221113	0.78671576	0.0067546	0.00578296	0.80429178	0.00162991	0.00653142
Q4PLS0	NA	0.428755	0.32618487	0.32012816	3.23990223	0.08829695	0.04030464	1.9559634	0.41224362	0.23106302
P68040	14694	0.759627	0.19694173	0.2585839	0.35695097	0.15596522	0.05996696	0.37507014	0.09862736	0.09004434
P19783	12857	0.925391	0.97901835	0.52851583	0.75924415	0.11779303	0.0485567	0.85128535	0.49807263	0.26058289
P24452	NA	2.000643	0.08493365	0.17419387	0.46497622	0.04837652	0.02532213	1.06704622	0.63582845	0.30438434
Q6PGF3	216154	2.12486	0.02466684	0.0931323	0.79941634	0.08373776	0.03890382	1.34889197	0.2749061	0.17503614
Q3V2M1	NA	1.25316	0.85755322	0.49476261	0.335347	0.01786582	0.01233456	0.95512089	0.62610602	0.30160462
Q9D1G3	74770	1.390051	0.04323941	0.12333913	0.64585843	0.05275867	0.02701411	1.00633626	0.44066935	0.24092009
P15327	12183	0.559192	0.3800259	0.34254523	3.0298321	0.74335116	0.1878086	0.91691824	0.69216907	0.32182664
Q9D8U6	69189	1.295923	0.36079216	0.33790045	0.70236261	0.12782746	0.05129328	0.53575799	0.07131884	0.0734661
Q8VCM7	99571	1.032559	0.15212608	0.23116332	0.68042934	0.05562055	0.02816266	0.99180635	0.19072096	0.13610169
P70296	23980	0.792495	0.2494518	0.28778108	0.80952052	0.42371828	0.12322151	0.67784334	0.12537144	0.10366831
P23492	NA	1.942539	0.52806179	0.38632235	0.98705705	0.27390098	0.09008024	1.11915712	0.01210353	0.02425084
P10639	22166	0.42155	0.54272471	0.39128579	1.57397731	0.54742335	0.14803468	1.63660306	0.21895005	0.15011896
P08249	17448	0.945707	0.01814888	0.07696397	0.7768582	2.03E-05	5.43E-05	1.32547359	0.21125214	0.146733
Q91ZA3	110821	0.996724	0.95666144	0.5249734	0.55796955	0.02966887	0.017824	0.61144279	0.02465431	0.0366955
P47754	12343	0.945509	0.07172859	0.15937033	0.7788541	0.01910231	0.01294727	0.88054051	0.59414102	0.29397341
Q71RI9	229905	0.429235	0.09646916	0.18445214	0.78321396	0.00789597	0.00642213	0.7666603	0.00034726	0.00211432
P54822	11564	1.122956	0.46289708	0.36127739	0.73758154	0.22039692	0.07615545	0.6809228	0.09067407	0.08478216
O08553	12934	0.867905	0.13256485	0.21424029	0.85406175	0.40000857	0.11814169	1.35810788	0.4658369	0.2487134
Q8BGQ7	234734	1.16389	0.04971727	0.13110064	0.668321	0.1889596	0.06842315	0.84063663	0.11768083	0.0993363
P16858	1E+08	1.01257	0.26372822	0.29725687	0.81340716	0.00658013	0.00567957	0.97975468	0.67288235	0.31579418
P97823	18777	1.006119	0.38504605	0.34254523	0.84359356	0.00567451	0.00512813	0.96661079	0.10338924	0.09144394
P01680	NA	0.551713	0.45249072	0.35815034	1.45868165	0.16158255	0.06134599	4.86855556	0.59692102	0.29438545
Q9D1I5	73724	0.830816	0.74504885	0.45870601	0.73843215	0.02242359	0.01450121	1.0497875	0.42998605	0.23743073
Q9Z2U2	30046	0.968935	0.82589354	0.48608466	0.3673668	0.46379439	0.13059678	0.75253871	0.95815335	0.39302417
Q06890	12759	1.25254	0.84558857	0.49070731	0.46734298	0.21647041	0.07516716	0.19575622	0.00804496	0.0182215
O88342	22388	0.929757	0.95821013	0.5249734	0.64690896	0.06113102	0.03020402	0.64094205	0.10677552	0.09270612
Q06986	20439	0.591962	0.30852785	0.3148666	0.9862733	0.97061656	0.23008945	1.51257612	0.4681178	0.24920146
Q7TMF3	66414	0.92752	0.37486703	0.34254523	0.78267829	0.44541699	0.12711462	0.71098201	0.12108377	0.10127108
P21981	21817	0.572455	0.28057213	0.30302887	0.90117852	0.39739761	0.11782019	1.54670064	0.12064286	0.10113427
O55126	NA	1.512782	0.09313391	0.18097356	0.5007471	0.01430619	0.0102902	0.53029887	0.04420893	0.05391689
P16015	12350	1.110224	0.87307284	0.50060358	0.36486422	0.06085858	0.03020402	0.81599339	0.58087887	0.28858617
Q8R5L1	NA	0.965895	0.25645128	0.29303838	0.75649813	0.00384041	0.00382174	0.81659558	0.05356103	0.06140658
Q8BMS4	230027	0.955758	0.23266936	0.27776791	0.70597466	0.00773767	0.00641678	0.92704733	0.919719	0.38461363

Q61233	18826	0.798145	0.36119123	0.33790045	0.67197269	0.00200927	0.00224392	1.18256955	0.6634552	0.31257673
O35639	11745	1.124062	0.21998614	0.27001475	0.61749561	0.00343555	0.00350972	0.96988557	0.25940146	0.16891585
Q9CQM9	30926	1.062347	0.68505803	0.44358482	0.81592301	0.84469288	0.20674357	0.92888137	0.60410869	0.29569556
P46412	NA	1.015691	0.00104629	0.01731502	0.71696414	0.33340202	0.10427085	0.81503534	0.33916408	0.20271436
Q9D855	NA	1.027554	0.67701187	0.4420566	0.31916432	0.1248803	0.05059066	0.73604482	0.62468676	0.30160462
Q923D2	233016	1.717455	0.03402382	0.11261184	0.72947624	0.13468887	0.05343815	0.92614275	0.24103359	0.16039211
P14211	12317	0.923377	0.35192608	0.33378734	0.64191669	0.14575248	0.05718394	0.68263319	0.32583829	0.19704744
A2AN99	NA	1.10893	0.20796328	0.26574358	0.64815368	7.06E-05	0.00014637	0.7448943	0.01932157	0.03202622
Q99N87	77721	0.998153	0.33572106	0.3254935	0.85791912	0.92079957	0.22127347	0.75095915	0.0590444	0.06604609
Q9R069	57278	0.132538	0.37532067	0.34254523		NA	NA	7.22587555	0.29316166	0.18336845
P13707	14555	0.930231	0.83422458	0.48765059	0.47341462	0.39360911	0.11723403	0.67862044	0.1284387	0.10548699
Q64433	15528	0.585844	0.26688466	0.29725687	0.65358924	0.37223372	0.1130976	0.73026632	0.1228388	0.10191246
Q11011	19155	0.616489	0.53592372	0.38968367	0.56400661	0.0237642	0.01495653	0.88614354	0.99857	0.40369928
P97467	18484	0.932148	0.837724	0.48865631	0.73759515	0.34155572	0.10606238	0.60040282	0.18052693	0.13113671
O70181	NA	0.7308	0.60354856	0.41918246	1.45878961	0.57769624	0.15441448	1.10954629	0.73834042	0.33398613
Q4KML4	NA	1.920106	0.00529923	0.04098806	0.70370957	0.03703324	0.02116591	0.95437426	0.06259957	0.06798558
P05064	353204	0.765838	0.14883959	0.22968202	0.88512478	0.65666588	0.16893497	1.12618797	0.0800148	0.07790712
Q91XE4	71670	3.605811	0.0297413	0.10495182	0.74507385	0.53189597	0.14494789	0.2297241	0.01927368	0.03202622
P85094	664994	0.74751	0.69859539	0.44667703	0.59533815	0.38871408	0.11610313	0.74678827	0.57273984	0.28571038
P29788	22370	0.891486	0.13172468	0.21424029	0.58642006	0.03098647	0.0183707	1.677631	0.70773517	0.32549663
Q8BS95	67549	0.028388	0.120116	0.20587887	27.6204391	0.36098172	0.11047247	13.8445403	0.20370884	0.14230671
Q9R0P3	1E+08	0.946798	0.39443831	0.34332691	0.77084476	0.00012437	0.00023482	0.86861364	0.12296852	0.10191246
Q9DCS9	68342	1.02552	0.70101565	0.44667703	0.74497894	8.88E-06	2.78E-05	0.82615517	0.00438107	0.01281746
	NA									
P10493	18073	1.246335	0.73785624	0.45869434	0.6142672	0.4058238	0.11888093	5.68741934	0.64168776	0.30587805
Q3TZ89	240667	0.769053	0.39006771	0.34285958	0.95795796	0.27759263	0.09085648	0.83483484	0.06586309	0.06958327
P97352	20196	1.135764	0.58434326	0.41180333	0.87197491	0.4575248	0.12934807	2.14198136	0.08049387	0.07806583
Q3TTY5	16681	1.725308	0.59723517	0.41600168	1.05358061	0.59187633	0.15724065	1.0349244	0.52350617	0.27001531
P14685	22123	1.222076	0.1541205	0.23259599	0.6354598	0.18082375	0.06638652	0.56898072	0.14646216	0.11412747
P29391	NA	1.102978	0.61666305	0.42411121	0.61393651	0.0914059	0.04129453	0.98695159	0.60173076	0.29507046
Q8BP40	66659	0.709617	0.55257125	0.39404167	0.84249615	0.11577353	0.04791101	1.04029762	0.08256947	0.07986641
Q9Z1P6	66416	1.414587	0.07561953	0.16274139	0.47494099	0.01538285	0.01084333	0.52867107	0.03622599	0.04803674
Q9Z2Y8	114863	0.932145	0.00994375	0.05549075	0.67829441	2.38E-07	1.17E-06	0.91185115	0.08552697	0.08153736
Q9D7J4	66359	0.880769	0.41227644	0.34376821	0.81821377	0.01066198	0.00817956	0.86433332	0.15083594	0.11653298
Q9DCZ4	68316	1.276247	0.18286531	0.25514656	0.57924588	0.00321005	0.00333003	0.74405013	0.05032711	0.05901027
P01843	NA	2.008355	0.31163862	0.3148666	1.44596996	0.05495219	0.02800166	0.8124593	0.14511777	0.11355877
Q9D6J6	72900	1.592877	0.36853268	0.34078273	0.40083744	0.05190819	0.02690436	0.43227258	0.10612187	0.0923585
Q91YT2	193670	1.146355	0.18564434	0.25514656	0.46143462	0.10660041	0.04581848	0.32947095	0.06621348	0.06958327
Q9EQI8	67308	1.39286	0.04370382	0.12333913	0.58202959	0.14809846	0.05764587	0.57715008	0.13492037	0.10765825
Q9Z0S1	23827	0.97029	0.57011581	0.40355462	0.78113044	0.20565664	0.07280229	0.94185982	0.22340745	0.15114538
Q63918	20324	1.231121	0.16877926	0.24545623	1.46004725	0.3324719	0.10427085	0.96265154	0.13397487	0.10765825
A3KMP2	239570	0.734452	0.00184461	0.02213162	0.74009297	0.05233659	0.02699408	0.99934727	0.15804643	0.1200686

Q8K4Z3	246703	0.482812	0.1063796	0.19492125	2.02898808	0.15918622	0.060654	0.84305083	0.36837922	0.21390526
P01872	NA	1.711712	0.24735263	0.28725945	0.5648744	0.37841721	0.11431935	0.79603823	0.12732365	0.10504438
P11404	14077	0.991571	0.95738628	0.5249734	0.68923161	0.01884294	0.01281254	0.87190336	0.38567852	0.22046636
Q9D6J5	67264	1.949465	0.45726256	0.35988972	0.31283842	0.54156533	0.14663807	0.4388198	0.45967865	0.24708362
P21956	17304	0.991481	0.87079939	0.49989727	0.83091969	0.02343606	0.01484877	3.24094479	0.63948956	0.30522921
A7YL62	NA	1.459017	0.04076382	0.12162997	0.77307065	0.01368677	0.00991208	1.26296837	0.87934806	0.37409387
P68254	1E+08	1.133371	0.49703888	0.37447244	0.58461419	0.00069926	0.00099242	0.85930577	0.26365714	0.17016769
Q9D1A2	66054	1.414862	0.2860829	0.30536834	0.53251206	0.02601595	0.01603956	0.61206041	0.11215152	0.09668315
Q6ZPL5	NA	13.17036	0.2016483	0.26155425	0.06036682	0.27363512	0.09008024	0.08282569	0.06784443	0.0706858
Q99PT1	192662	1.090285	0.03118654	0.10709017	0.65606207	9.33E-14	1.64E-12	0.90345114	0.00090142	0.00444205
Q9D0S9	68917	0.924953	0.03719826	0.11419116	0.73144768	0.02301564	0.01476956	0.83147662	0.2285821	0.15350707
Q91VI7	107702	0.780689	0.4737788	0.36264109	1.00420194	0.68584717	0.17495233	0.87212114	0.94936461	0.39029718
Q9Z0X1	26926	1.614784	0.18082508	0.25460918	0.52357514	0.05601543	0.02827096	0.41671457	0.0044991	0.01281746
Q9EQ20	104776	0.864531	0.1986943	0.25982939	0.78948753	0.0031317	0.00327851	0.94253233	0.02326836	0.03520747
P14094	11931	0.875797	0.91227355	0.51387136	0.56433928	0.52806568	0.14421117	1.13743193	0.91594871	0.38435928
P21300	11997	1.072629	0.26515384	0.29725687	0.83920917	0.08159605	0.03812504	0.78288531	0.01147008	0.02323699
P63017	15481	1.018591	0.94863982	0.5249734	0.92441549	0.51441517	0.14127651	0.64291088	0.02173181	0.0344417
Q9DCI3	76205									
P31001	13346	0.806239	0.64228699	0.43024953	0.69356399	0.45431931	0.12900877	0.60766156	0.13156454	0.10661353
P45591	12632	0.484147	0.11262825	0.19928419	1.18474424	0.37718303	0.11420037	1.11495986	0.35775195	0.21011116
P63038	15510	1.018612	0.02837624	0.10304653	0.70721977	1.24E-11	1.54E-10	0.79265187	2.56E-09	1.33E-07
Q6ZQA6	78908	3.121065	0.40440419	0.3434027	0.04339072	0.04191959	0.02296227	0.73471295	0.69279645	0.32182664
P26039	21894	1.470508	0.1670556	0.24413881	0.71254313	0.41435731	0.1210273	0.98247951	0.63605003	0.30438434
Q91V12	70025	0.817743	0.82162992	0.48441855	0.94917459	0.92017128	0.22127347	0.94676521	0.74823073	0.33726635
Q9D8S4	104444	1.894869	0.70368362	0.44730054	0.50686813	0.12628027	0.05086548	0.46794954	0.42694171	0.23675941
P45377	14187	0.930887	0.92977173	0.52128053	0.80526125	0.04666938	0.02467283	0.86190644	0.17898274	0.1302745
P58771	22003	1.023864	0.98969882	0.53069746	0.7400044	0.098535	0.04341064	0.80874048	0.17371178	0.12740815
A8DUK7	NA	0.879864	0.37323414	0.34254523	1.33708291	0.7718319	0.19310196	0.70941318	0.01069298	0.02202984
P01865	NA	1.982858	0.04247615	0.12326938	0.62736427	0.18769052	0.06809038	1.01144429	0.84210363	0.36600716
P09405	17975	1.793043	0.13970013	0.22054243	0.661708	0.41089954	0.12018334	0.48672212	0.06622318	0.06958327
Q9CWJ9	108147	2.570002	0.0134112	0.06443641	0.42835338	0.07274966	0.03472755	0.46477366	0.03675106	0.0482332
P84244	667250	0.586916	0.47186371	0.362331	1.54629065	0.53671766	0.14569859	0.79111599	0.24859056	0.16362923
P61089	1E+08	1.029975	0.94334737	0.52410529	0.81892419	0.37139285	0.11300448	0.93389266	0.53944428	0.27589451
A2ASS6	22138	1.323174	0.01828199	0.07696397	0.63419191	0.01070182	0.00817956	0.78336292	0.03379863	0.04626247
Q3UIU2	230075	0.953886	0.30515297	0.31432687	0.7350368	0.03740695	0.02120754	0.80590954	0.33136363	0.20005706
P08228	20655	0.919769	0.92682224	0.52084381	0.72338796	0.62005585	0.16168021	0.79327044	0.60202243	0.29507046
P49817	12389	0.417203	0.29544363	0.30890922	2.31129786	0.44358811	0.12710729	1.5491131	0.6220338	0.3015356
P12710	14080	0.83422	0.37537136	0.34254523	0.99596858	0.51147664	0.14074611	1.35405236	0.54468259	0.27779438
P19123	21924	0.945927	0.70070403	0.44667703	0.76871363	0.00103877	0.0013233	1.00308994	0.91779052	0.38461363
Q99KQ4	59027	1.157632	0.17868501	0.25371161	0.52621704	1.01E-05	3.10E-05	0.99003253	0.93682639	0.38776554
Q9JKF7	27393	1.411864	0.0471055	0.12745391	0.37074346	0.00304523	0.00320384	0.78282133	0.05985123	0.0661371
Q61037	NA	0.993239	0.18535953	0.25514656	0.81842887	0.0010283	0.0013233	0.84144881	0.00035741	0.00211432

P43274	50709	1.31645	0.80038085	0.47775976	0.09057787	0.15685767	0.05998872	0.11951162	0.21983194	0.15011896
Q9CXZ1	NA	0.925857	0.00311199	0.03111475	0.73989257	0.00802802	0.00647969	0.79677572	0.03822321	0.04890669
P58774-2	22004	8.437076	0.47856764	0.36456226	0.13332388	0.40085157	0.11822555	0.14988768	0.72639838	0.33028321
Q9R229	12154	0.952817	0.64279225	0.43024953	1.0032543	0.840596	0.2060701	0.82168631	0.93861463	0.38806523
Q9CR68	66694	0.788674	0.30275482	0.31381883	0.76900809	0.08922562	0.04057734	0.96982682	0.46225127	0.24752382
Q60854	20719	1.828973	0.00444065	0.0373887	0.44890836	0.04362786	0.02359576	0.40111011	0.01520238	0.02758045
Q8BGC4	225791	0.259779	0.11243715	0.19928419	1.36451493	0.45497241	0.12900877	1.00629351	0.38572431	0.22046636
Q6IRU2	326618	1.591109	0.60185119	0.41860938	0.48400516	0.30824277	0.09816841	0.35985733	0.06425062	0.06887952
P19096	14104	1.748752	0.19235991	0.25830548	1.17812107	0.91412066	0.22041992	1.13995292	0.74925235	0.3373099
Q62425	17992	0.954793	0.15857004	0.23560664	0.82874797	0.10766469	0.04618206	0.84649106	0.09683658	0.08917236
P23242	14609	0.980646	0.85979317	0.49476261	0.18087938	1.03E-05	3.11E-05	0.28138466	0.00034905	0.00211432
O35643	11764	0.871099	0.60676469	0.42080718	0.73414388	0.29688864	0.09560178	0.80118006	0.05141859	0.05971404
Q60932	22333	1.23555	0.32534446	0.32012816	0.7001883	0.1657301	0.06232764	0.77437893	0.8922003	0.37706095
Q8VCW8	264895	0.998548	0.2459801	0.28725945	0.56350902	6.91E-06	2.25E-05	0.81493681	0.01025465	0.02149104
P10649	14862	1.684541	0.05179275	0.1322148	0.41675673	0.06735569	0.03251974	0.80039017	0.73172249	0.33187639
Q9CPQ1	621837	0.916605	0.40402279	0.3434027	2.5254965	0.62097321	0.16172001	1.13624582	0.56860545	0.2840365
Q80WQ9	1E+08	1.039249	0.50532688	0.37703899	0.70869639	0.19843079	0.07097228	0.6874631	0.09559378	0.08847477
P55264	11534	0.956434	0.82648151	0.48608466	0.70504773	2.39E-09	1.81E-08	0.96343822	0.0349624	0.04721972
P32261	11905	1.047252	0.09153056	0.18077101	0.7534904	0.02460695	0.01539529	0.91552527	0.01440013	0.02652084
OTTMUSF	NA	1.254661	0.45032931	0.35815034	0.59136242	0.10287167	0.04493752	0.95900128	0.59340871	0.29397341
Q8BLF1	320024		NA	NA		NA	NA		NA	NA
P60766	12540	0.339791	0.64791907	0.43247479	6.66521193	0.04866176	0.02540854	5.40942227	0.5337592	0.27434201
Q8BTM8	192176	0.821162	0.45293745	0.35815034	1.57579696	0.89997591	0.21800355	0.7331985	0.22851062	0.15350707
P62827	1E+08	1.895077	0.03610751	0.11419116	0.65267607	0.34957373	0.10796617	0.53047934	0.05689083	0.0642282
Q3ULW3	NA	0.909654	0.45743601	0.35988972	0.77115478	0.01883933	0.01281254	0.77977367	0.00695666	0.01658042
Q9CR21	1E+08	3.107935	0.171534	0.24712757	0.25484139	0.11977022	0.04908469	0.86118279	0.76050709	0.34153344
P12246	20219	1.727026	0.21111113	0.2666223	1.51758461	0.27936262	0.09116753	0.76146165	0.38653793	0.22058565
Q8BH86	217830	0.600794	0.68827284	0.44358482	0.84047912	0.10338772	0.04493752	4.57578737	0.51012928	0.26461285
Q60692	19175	0.488584	0.00938751	0.053634	1.6476498	0.1627326	0.0616719	1.17125147	0.79566133	0.35254488
P58252	13629	1.071227	0.90819677	0.51277868	0.64305095	0.01291131	0.00948036	0.63181622	0.01262316	0.02461572
P01942	NA	1.490749	1.07E-10	2.57E-08	1.1740834	0.43783914	0.12608382	1.15010244	0.76988146	0.34447067
Q78IK4	68117	1.083144	0.90668848	0.51277868	1.58855575	0.59923667	0.1582023	0.82883606	0.62427403	0.30160462
Q9D020	107569	1.024773	0.18090872	0.25460918	3.2611591	0.07605822	0.03582174	0.78794429	0.01544989	0.02783225
Q99KI0	11429	0.935118	0.45298637	0.35815034	0.74585945	0.28064263	0.09144414	0.72525382	0.10299585	0.09144394
Q7TQI3	107260	0.567875	0.03707008	0.11419116	0.65731894	0.00435299	0.00422258	1.04767981	0.09951201	0.09004434
P14824	11749	0.856636	0.7537912	0.46201729	0.83582052	0.08487425	0.03918839	0.91059012	0.84611022	0.36644683
Q9JI91	NA	1.371621	0.09140963	0.18077101	0.84549621	0.49604082	0.13802277	0.76818127	0.1702749	0.12594754
Q571E4	50917	3.00562	0.4339994	0.35242814	0.33360236	0.43730492	0.12608382	0.65174872	0.83154617	0.3626865
A3KG59	242377	0.971143	0.78225969	0.47222911	0.52541286	0.4242003	0.12322151	0.64373189	0.64256952	0.3058985
Q9DCZ1	66355	0.833216	0.7009876	0.44667703	0.68815251	0.08649833	0.03967836	1.4050861	0.09039203	0.08473572
P07310	12715	1.045247	0.66587275	0.4396466	0.70683785	0.12714344	0.0511158	0.64045884	0.01261338	0.02461572
P00405	17709	0.482358	0.8287263	0.48648184	2.03425469	0.69424103	0.1766673	1.70985561	0.24759938	0.1635673

P63030	1E+08	1.120447	0.8135387	0.48117814	0.69442763	0.01159907	0.00869803	0.73775082	0.07468352	0.07567829
Q99J39	56690	0.925549	0.44669222	0.35729428	0.56752412	0.0007516	0.00103883	0.71737073	0.00678373	0.0163824
P50543	1E+08	1.012626	0.61245358	0.42231146	0.68687787	0.12943587	0.05164469	0.66862658	0.13474256	0.10765825
P17710	15275	0.593609	0.89823255	0.50955064	0.87827727	0.21130137	0.07397965	1.16835416	0.43040211	0.23743073
O09111	104130	0.814696	0.19483782	0.25830548	0.78077313	0.51157034	0.14074611	3.1376126	0.56790459	0.2840365
O70433	14200	1.3341	0.49934711	0.3756218	0.62453753	0.61809246	0.16136723	0.84469653	0.30112102	0.187703
P27773	14827	0.914947	0.12479776	0.20931915	0.45199179	0.00050766	0.00075073	0.54664874	0.00501983	0.01331186
P28474	11532	0.634851	0.15211325	0.23116332	0.82989568	0.0327868	0.01925943	1.39998383	0.78057799	0.34797576
Q9CYR0	381760	0.933342	0.40646673	0.3434027	0.72506167	0.07598314	0.03582174	0.77064491	0.10293071	0.09144394
Q9Z1E4	14936	1.548056	0.12712275	0.21119779	0.55761586	0.1248146	0.05059066	0.58678441	0.04609729	0.05566144
P08074	12409	0.393958	0.00775738	0.04898584	0.90981758	0.17988229	0.06627098	0.93130712	0.95159051	0.39077173
O08528	15277	1.00728	0.39346701	0.34332691	0.35417059	0.00718168	0.00607347	0.98160709	0.56090355	0.28251117
P62821	19324	0.90021	0.43312594	0.35242814	0.90185041	0.66211502	0.16992338	1.14998732	0.26265463	0.17004046
Q8K411	69617	0.842429	0.20313103	0.26276728	0.74352093	0.00042773	0.00066022	1.49156582	0.62577072	0.30160462
Q9CXV1	66925	1.296941	0.97571709	0.52813154	0.28328473	0.08427033	0.03899469	1.72703993	0.36735173	0.21364884
Q9CWL2	69743	1.766234	0.04830968	0.12815937	0.42541223	0.00577001	0.00519225	0.97536813	0.56538776	0.28403019
P19536	NA	0.675476	0.71242521	0.45029542	2.04757006	0.10331147	0.04493752	0.91620824	0.19301329	0.13693362
Q922F6	NA	0.705899	0.42987903	0.35146096	0.59172141	0.04151904	0.02292212	0.91837211	0.62265512	0.3015356
Q61316	NA	0.922589	0.74480667	0.45870601	0.82314458	0.15961422	0.06070769	0.90987822	0.8175746	0.35747637
Q8BH59	78830	0.954058	0.38396915	0.34254523	0.69134885	0.26945465	0.08903333	1.30377391	0.83992229	0.3654947
Q9CQI6	72042	2.99992	0.29441363	0.30856279	0.46356613	0.39561793	0.11766662	0.42168101	0.33887881	0.20271436
Q9D8Y0	NA	0.152999	0.31643792	0.31658366	6.03030508	0.10582965	0.04560125	1.90812843	0.43091638	0.23743073
Q9CZ44	386649	0.908344	0.27897307	0.30302887	0.68799176	0.08166982	0.03812504	3.3538773	0.13407784	0.10765825
Q8VDK1	27045	2.051225	0.26622216	0.29725687	0.41659236	0.23130126	0.07863837	0.40076167	0.17815025	0.1299279
Q80X68	NA	0.293306	0.83379937	0.48765059	2.07286927	0.97515523	0.23066577	1.91682816	0.14890358	0.11577593
Q9CR62	67863	2.02505	0.12784348	0.21156777	0.35137584	0.05779884	0.02892164	0.37157355	0.06441077	0.06887952
O35943	14297	0.622789	0.54514682	0.39142046	1.41774692	0.38811511	0.11610313	1.47978584	0.4730238	0.25108008
Q9QYG0	29811	1.199034	0.00012789	0.00340983	0.60882754	1.76E-14	3.72E-13	0.84918535	0.00360878	0.01144324
Q8K0Z7	70207	0.508084	0.15304246	0.23169764	1.73137948	0.23307261	0.07911341	3.71419819	0.01084419	0.02218293
Q9CW03	13006	1.294581	0.80682062	0.47932998	0.46419179	0.11231045	0.04723	0.48693802	0.10535946	0.09213475
Q61598	14569	0.957324	0.96913229	0.52715185	0.79088874	0.00022783	0.00038619	0.93014685	0.01042273	0.02159509
Q9JMH6	NA	0.943074	0.06650865	0.1521549	0.47863813	6.27E-06	2.10E-05	0.95406709	0.50436669	0.2622471
P80316	12465	0.709053	0.20820244	0.26574358	0.7470093	0.64238407	0.16606893	0.67978559	0.02970573	0.04182409
Q9EPK5	97064	0.917682	0.38899633	0.34254523	0.79395488	0.19075709	0.06895595	0.81671904	0.3610537	0.21167395
P35980	19899	0.20022	0.15102383	0.23116332	2.77403832	0.10528395	0.04553027	1.47990127	0.09218718	0.08597651
Q91WS0	52637	1.776298	0.03589334	0.11419116	0.74946543	0.03824585	0.02151015	0.327218	0.03088739	0.04332055
Q91YY4	246782	0.87229	0.79278421	0.47547162	0.57098833	0.00156133	0.0018343	0.73590968	0.00677298	0.0163824
O70250	56012	0.96534	0.93136513	0.52156457	1.13924628	0.87025798	0.21104664	0.88522368	0.21339621	0.14759372
Q06185	11958	0.946692	0.02276995	0.08742206	0.75928928	0.2011118	0.07171826	0.77250355	0.19056805	0.13610169
Q6PCY5	NA	0.930301	0.00462897	0.03830234	0.67479752	0.10348828	0.04493752	0.8360967	0.59582593	0.29438545
Q3UWA4	195359	1.134386	0.67956553	0.44225753	1.26584727	0.49194898	0.13712334	0.7386045	0.19626812	0.13843478
Q08857	12491	1.032893	0.05134122	0.131763	0.71805835	1.06E-14	2.68E-13	0.96004335	0.03434092	0.04671726

P40124	12331	1.014233	0.21902168	0.26952026	1.43709093	0.29900604	0.09594922	1.52672874	0.55354388	0.28035322
P03911	17719	0.958638	0.13393306	0.2149738	0.77650926	0.02585787	0.0160356	0.84025875	0.07566232	0.07567829
Q3V3A1	271697	1.078982	0.02973453	0.10495182	0.64778662	0.00545488	0.00497215	0.8496667	0.1499794	0.1163643
Q8R404	224904	0.794221	0.14553765	0.22604026	0.66617241	0.00628973	0.0055652	0.68311176	0.00378957	0.01191292
Q9D6R2	67834	0.694699	0.73208368	0.45671382	0.73176903	0.00144886	0.00174084	0.83281854	0.01403649	0.02623073
Q99LY9	1E+08	0.877234	0.07811709	0.165154	0.77949999	0.04036104	0.02257966	0.85176117	0.33788049	0.2026495
Q9D1L0	1E+08	1.159435	0.24568741	0.28725945	0.6208094	0.21271436	0.07435126	0.97722439	0.39671171	0.22533362
Q8QZR5	76282	1.137317	0.23161218	0.27719535	0.62504393	0.00023031	0.00038654	0.8119335	0.01773631	0.03094589
A2A9C3	230676	0.738576	0.11871215	0.2049365	2.19610327	0.11000279	0.04698474	1.48888324	0.66214682	0.31236388
Q9QUH0	93692	1.084654	0.66847623	0.43991358	1.00183959	0.2526758	0.08481448	0.87668462	0.93090782	0.38664874
Q8K0D5	28030	0.59301	0.41330685	0.34376821	0.78709395	0.02961835	0.017824	1.26527779	0.38482155	0.22046636
P43023	12862	0.913243	0.74552138	0.45870601	0.78515354	0.16984129	0.06307143	1.05601306	0.76623457	0.34368232
Q7TNG8	52815	0.99304	0.25126849	0.28918177	0.76781996	0.1195439	0.04908469	0.8103481	0.15703917	0.11955245
Q8R2G4	109979									
P48774	14866	0.770489	0.44395286	0.35698742	0.52622813	0.00638578	0.00562665	0.81638421	0.19585557	0.13841152
Q0KK59	217843	0.678801	0.47851808	0.36456226	0.60258621	0.07412877	0.03514786	0.85201564	0.04059023	0.05121647
P05125	NA	1.459955	0.00011524	0.00325315	0.66617846	0.06215926	0.03064047	1.06166954	0.0024283	0.00859708
Q60936	67426	3.357942	0.07523997	0.16265394	0.46185574	0.12102088	0.04950131	1.16450724	0.31129161	0.19239838
Q01853	269523	1.067642	0.29317264	0.30856279	0.81909446	0.25886379	0.08634337	0.82260069	0.07498496	0.07567829
Q8BM89	271970	1.035513	0.43357269	0.35242814	0.50509449	0.77181006	0.19310196	0.57932703	0.77757371	0.34706076
P56376	66204	0.924967	0.01687078	0.0736057	0.89700927	0.66581436	0.17066565	0.91111608	0.45517652	0.24590201
P03930	17706	0.879994	0.19461679	0.25830548	2.55676549	0.97163075	0.23008945	0.92978913	0.07742459	0.07651358
O08804	NA	0.922183	0.42052079	0.34676353	1.44559963	0.38581066	0.11605552	0.72641638	0.46667878	0.24879861
Q8CI51	56376	1.151011	0.31406649	0.31598913	0.53767209	0.00099927	0.00130441	0.81745218	0.08540691	0.08153736
P62270	1E+08	0.934373	0.95932986	0.5249734	0.85693696	0.56182807	0.1513494	0.8089141	0.51772023	0.26778856
Q9DC70	75406	1.100687	0.32866576	0.32124908	0.55697164	0.19351437	0.06959576	0.72978379	0.47534614	0.25158037
P24527	16993	0.739432	0.15011958	0.23091476	0.89537026	0.65602055	0.16893497	1.19869495	0.70033257	0.32409341
O35459	51798	0.845707	6.71E-07	4.60E-05	0.81563397	2.49E-07	1.20E-06	1.02329199	0.06640459	0.06958327
Q8VHN7	110789	0.385477	0.04344039	0.12333913	9.37727117	0.14829285	0.05764587	6.65849807	0.09385371	0.08730747
Q5SWD9	104662	1.964885	0.62285807	0.42520924		NA	NA	0.71638031	0.65521911	0.30989757
Q3TIT9	NA	0.979628	0.09397774	0.18113176	0.84335356	0.17873557	0.06596344	0.75976566	0.00026523	0.00172714
P50396	14567	0.557736	0.65671884	0.43713254	1.78301429	0.22458441	0.0771223	0.92570866	0.51417582	0.266333
Q9WV98	30056	0.725208	0.38742486	0.34254523	0.40378712	0.43014699	0.12449848	1.31259396	0.79784805	0.35265678
P35505	14085	0.812941	0.46419687	0.36127739	0.75887996	0.00525236	0.00487154	2.02616304	0.27268776	0.17445234
P22599	20701	1.09423	0.21310836	0.26773555	0.78079042	0.29701914	0.09560178	0.75542276	0.07320747	0.07469649
O35678	23945	0.946476	0.99480321	0.53165484	0.70347556	0.11254772	0.04723	1.18355884	0.49381147	0.25947033
P06909	NA	2.929794	0.04578542	0.12694927	0.44311392	0.0521086	0.0269422	0.22703748	0.00903429	0.01972711
P50462	13009	0.929286	0.3492581	0.33257138	0.80930771	0.00982404	0.00769437	0.95648931	0.21777004	0.14983327
Q505D7	403187	0.79592	0.67039589	0.43991358	1.23900333	0.71441717	0.18158312	0.62832984	9.46E-05	0.00093234
P41216	14081	1.214105	0.76059647	0.46440906	0.59775453	0.06313096	0.03090335	0.50639425	0.00387309	0.01207139
P22907	15288	1.361986	0.06626785	0.1521549	0.95059506	0.67942025	0.17373203	0.77360458	0.00013031	0.00110511
Q9CXJ4	74610	3.254102	0.19339914	0.25830548	0.616307	0.56994514	0.15314585	0.68560734	0.24829265	0.16362923

P28656	53605	0.796789	0.4054921	0.3434027	0.82867929	0.65842559	0.16918186	1.05526363	0.22676477	0.15286828
O08600	13804	1.276952	0.09878001	0.18518169	0.42913926	5.50E-05	0.00011991	0.9413913	0.6533675	0.30942315
Q9WVA4	21346	1.040482	0.65083363	0.43381685	0.66752004	0.2236201	0.0771223	0.53175373	0.13094739	0.10634975
P09813	NA	0.998243	0.46452869	0.36127739	0.37148866	0.00717046	0.00607347	0.49654753	0.06378816	0.06883911
Q99LD8	51793	0.316155	0.21703465	0.26845178	0.68655814	0.21068174	0.07397965	1.07608445	0.7988781	0.35268458
A2AKY4	241514	1.237625	0.35051981	0.33311187	0.4312253	1.39E-05	3.92E-05	0.72787281	0.00299422	0.00992605
Q99LP6	17713	1.036283	0.23635636	0.28077268	0.8113437	0.11102385	0.04698474	0.69431623	0.02164492	0.0344417
P62204	12315	1.121324	0.00133576	0.01823938	0.68264943	1.84E-07	9.26E-07	0.96128495	0.33777899	0.2026495
O08797	NA	1.580048	0.00803578	0.04959503	0.7087291	0.02318776	0.01476956	0.78444701	0.06727164	0.07028984
P35969	NA	1.066107	0.84965591	0.49187809	0.7407048	0.02689542	0.01643801	0.78543432	0.12983452	0.10591773
P27005	20201	0.923105	0.66793784	0.43991358	1.69306064	0.60097162	0.15830008	1.47079533	0.70783769	0.32549663
Q3UFU2	NA	1.295134	0.04658531	0.1270297	0.58834858	3.26E-05	8.21E-05	0.89436886	0.28036959	0.17719077
P68510	22629	1.741619	0.26880027	0.29725687	0.58695872	0.64501371	0.16654514	0.44849378	0.19245659	0.13680482
Q6XPS7	NA	1.253123	0.01226357	0.06171068	0.75131921	0.15271933	0.05893325	1.2051567	0.56664668	0.28403019
P24270	12359	1.364098	0.00580782	0.04288136	1.18139149	0.59401545	0.15756501	1.66268	0.46536926	0.2487134
Q8C196	227231	3.640275	0.01042258	0.05620231	0.26907855	0.00402071	0.00393577	0.79813206	0.02313603	0.03520747
Q8BH64	259300	1.02302	0.45259718	0.35815034	1.805286	0.40467537	0.11885602	1.18848126	0.37360906	0.21622625
Q09LZ8	78933	0.88512	0.52641697	0.38570699	0.77943956	0.02798812	0.01691035	0.85227608	0.11852705	0.09980321
Q9CQ89	67675	0.86222	0.94109685	0.52410529	0.74483995	0.04515933	0.02421479	1.04970956	0.07548105	0.07567829
Q00519	22436	1.500843	0.21772091	0.26860833	0.46135955	0.0456755	0.02433062	0.70068475	0.27801593	0.17654737
Q60931	22335	1.21366	0.80800526	0.47932998	0.49803716	0.24246831	0.08164738	0.56982505	0.03803854	0.04890669
P52480	18746	0.93358	0.95567946	0.5249734	0.87062547	0.34325579	0.10643401	0.8469869	0.0060853	0.01545406
O35215	13202	1.078544	0.49428147	0.37356789	0.69873027	0.00030916	0.00050291	0.76810904	0.02856393	0.04052946
Q9DCJ5	68375	0.954233	0.61900788	0.42411121	0.90417207	0.16630413	0.06235494	1.89357572	0.70993777	0.32550019
Q61599	11857	0.679549	0.26658763	0.29725687	0.60902377	0.39675839	0.11782019	1.17169791	0.21370556	0.14759372
Q8K2B3	66945	1.110893	0.35552376	0.3365345	0.71844648	0.04335332	0.0235074	0.69388673	0.02524919	0.03712631
Q05816	16592	1.067254	0.76954044	0.46919637	0.55297164	0.15224646	0.05885818	1.14272091	0.32358899	0.19633841
P70188	16579	1.030136	0.46034549	0.360917	1.48452236	0.33430717	0.10427085	0.92120442	0.73912172	0.33398613
Q9R1P1	1E+08	1.075714	0.97099965	0.52715185	0.72294748	0.00349357	0.00355183	0.78691104	0.0248897	0.03689525
Q9D1M0	110379	0.675299	0.04263776	0.12326938	1.06672424	0.17000457	0.06307143	0.91624532	0.90261533	0.38007632
Q5R1I7	NA	0.712175	0.11641093	0.20168933	4.64823112	0.13216395	0.05253495	2.0921659	0.45894796	0.24708362
P99027	67186	1.418736	0.51964252	0.38249522	0.49551285	0.08388999	0.03890382	0.60391426	0.32338366	0.19633841
Q9EQP2	98878	1.186071	0.98670351	0.53069746	0.31747761	0.38005127	0.11464922	0.39719073	0.35781186	0.21011116
Q2TPA8	72479	0.698154	0.94835345	0.5249734	0.94064738	0.43494743	0.12565292	1.7029713	0.08410653	0.08113793
P99024	22154	1.3126	0.0037784	0.03358015	0.71639521	0.00651546	0.00567291	0.93179387	0.27359518	0.17472632
Q8VDC0	102436	0.978084	0.0345988	0.11370596	0.72649999	0.43822764	0.12608382	0.94928404	0.44989594	0.24449814
Q9CQZ6	66495	1.072718	0.39892658	0.34332691	0.67494192	0.02345255	0.01484877	0.69145317	0.0136547	0.02579947
ENSMUSF	NA	0.706468	0.32740265	0.32066757	3.4985212	0.11844395	0.04873003	1.28755873	0.49775668	0.26058289
Q30D77	71355	2.488966	0.28224263	0.30302887	0.48817791	0.33913722	0.10546624	0.87749523	0.37837435	0.21762983
Q62426	13014	1.225714	0.19720306	0.2585839	0.54629831	0.00031451	0.00050537	1.97669722	0.01222458	0.02435953
P61982	22628	0.903688	0.61948221	0.42411121	0.76311037	0.00032258	0.0005129	0.87686413	0.00761819	0.01747192
P26041	17698	1.499873	0.40928969	0.3434027	0.41512609	0.04144125	0.02292212	0.76234601	0.66880258	0.31428396

Q9CQV8	54401	0.952811	0.31651118	0.31658366	0.33005448	0.00771052	0.00641678	0.45704181	0.04940537	0.05830444
Q91YT0	17995	0.920812	0.25636534	0.29303838	0.78602884	0.00030311	0.00049689	0.84689509	0.00400573	0.0120721
P56392	12865	1.087223	0.71324281	0.45029542	1.17656055	0.62271856	0.16197507	0.89487249	0.93277646	0.38696768
A2AMM0	68016	0.888332	0.0973274	0.18481007	0.67785567	0.29630837	0.09560178	0.70810461	0.43103141	0.23743073
P20108	11757	0.83898	0.28354079	0.30374313	0.93299778	0.33366801	0.10427085	1.10275388	0.86972717	0.37224549
P54726	19358	1.33872	0.10356683	0.19190656	0.49149818	0.00524228	0.00487154	0.82328749	0.02962498	0.04182409
Q8R1A4	NA	0.992011	0.41675791	0.34425214	0.6918789	0.11496511	0.04785742	0.69253908	0.04654604	0.05601781
Q9QXW2	30839	2.284896	0.12517625	0.20931915	0.22691613	0.8699258	0.21104664	0.16414248	0.54929923	0.27870683
Q9WUM5	56451	0.89036	0.49034804	0.37117961	0.91383604	0.99673593	0.23472032	0.9222484	0.67492183	0.31593811
P48772	12869	0.163954	0.7126579	0.45029542	1.18618123	0.99076698	0.23383549	0.55501873	0.80560289	0.35393941
Q01768	18103	0.814729	0.28126415	0.30302887	1.69516669	0.09976163	0.04385967	0.83074595	0.00663101	0.01625448
P56375	75572	0.897418	0.00044619	0.00931026	0.69903675	0.00403871	0.00393577	0.92625329	0.86335055	0.37098139
Q3T9C9	NA	1.314968	0.38117556	0.34254523	0.56804361	0.01275703	0.00939971	0.58342562	0.03598066	0.04788556
Q9QZB1	58175	0.014149	0.54468757	0.39142046	2.02842706	0.02594952	0.01603956	3.25138369	0.26500157	0.17049873
Q3UAI4	NA	0.989554	0.09314149	0.18097356	0.78766897	0.11537846	0.04791101	0.79742865	0.3204403	0.19573063
P07356	12306	0.97596	0.53745986	0.38968367	0.95789214	0.80600322	0.19958405	0.98453293	0.74183417	0.33479693
P16125	433229	1.02483	0.68102374	0.44225753	0.65728163	0.04059858	0.02262284	0.72910234	0.16025149	0.12149069
Q60590	18405	1.196056	0.69921278	0.44667703	0.76916476	0.64124097	0.16597632	0.85239714	0.48948019	0.2582078
Q9D051	68263	0.80666	0.00140619	0.01823938	0.83414606	0.01071427	0.00817956	0.79866563	0.00059754	0.00325218
Q9CPV4	67201	0.648734	0.05886447	0.1456198	1.28694091	0.21845796	0.07570067	0.7795415	0.00610266	0.01545406
O35855	12036	0.591275	0.37934686	0.34254523	0.76703501	0.5118183	0.14074611	0.85578573	0.76922847	0.34447067
Q61704	16426	2.109288	0.35968463	0.33780798	0.76350934	0.20587296	0.07280229	0.43927195	0.18321236	0.13229681
Q9WUU7	64138	2.432884	0.31244906	0.31502223	1.02571298	0.59762992	0.15797533	0.65190478	0.57465054	0.28627191
P60335	23983	0.759022	0.13684582	0.21818956	1.52310604	0.68270739	0.17436174	0.86412408	0.88815633	0.37659709
Q8VEM8	1E+08	1.306976	0.78755005	0.47422972	0.61398431	0.49119113	0.13712334	1.50575446	0.91516584	0.38435928
P04117	11770	1.098452	0.71402473	0.45029542	0.52908409	1.01E-12	1.64E-11	0.89655799	0.04823633	0.05748292
P51881	11740	1.763713	0.0412512	0.12220542	0.94501124	0.38773951	0.11610313	0.42075139	0.00346998	0.01119786
P63330	19052	1.015596	0.62885442	0.426271	0.74277545	0.00530834	0.00490196	1.16656837	0.8688813	0.37224549
P00158	17711	0.771386	0.00133869	0.01823938	0.86088857	0.81991547	0.2020825	0.88397747	0.90800282	0.3819039
P56379	70257	1.230511	0.7271988	0.45561007	0.42056006	0.10158828	0.04457009	0.54961727	0.50386558	0.2622471
ENSMUSF	NA	0.683279	0.66594839	0.4396466	0.96580606	0.32665579	0.10294725	1.13794053	0.16590199	0.12397885
Q8C801	NA	0.271153	0.8928534	0.50890532	5.562977	0.18771863	0.06809038	3.5299571	0.25289806	0.1661646
P70392	NA	1.069655	0.07778989	0.165154	0.97938369	0.22544966	0.0771223	1.17348124	0.35365758	0.20945208
Q9JK42	18604	1.381737	0.10798888	0.19666454	1.25345826	0.74451385	0.18787789	0.52146952	0.03386021	0.04626247
Q62234	17929	0.709819	0.46522209	0.36127739	0.73757507	0.51792903	0.14205705	0.61911537	0.26165218	0.16977512
P97429	11746	1.614928	0.4818663	0.36591348	0.63561643	0.73036536	0.18496971	0.54714911	0.41234615	0.23106302
P56391	110323	0.92475	3.82E-05	0.0013095	0.77152705	0.44511737	0.12711462	0.80049473	0.45489883	0.24590201
Q6ZQ80	NA	0.538503	0.27589862	0.30230431	7.44615477	0.95849614	0.22774418		NA	NA
P63028	22070	0.776337	0.51713095	0.38181774	0.76049344	0.01046885	0.0081093	1.35274361	0.80318136	0.35330118
XP_89444	NA	0.786164	0.31921586	0.31658366	1.143287	0.75633293	0.1901796	1.33369109	0.87622977	0.37397979
Q99MR8	72039	0.824142	0.3806756	0.34254523	0.73184602	0.01309171	0.00954653	0.76404165	0.00487401	0.01331186
Q3ULD5	78038	4.339221	0.00116548	0.01823938	0.72605917	0.53944738	0.14625186	0.4677179	0.02764117	0.03937335

Q60930	22334	0.760038	0.96634895	0.52715185	0.96837512	0.26447151	0.08807489	0.84028052	0.55522908	0.2806639
P08113	22027	1.341533	0.67189655	0.43991358	0.61627605	0.42212631	0.12311737	0.65494238	0.52172152	0.26947597
P80314	12461	2.835142	0.01925805	0.07899424	0.61762639	0.00022828	0.00038619	0.753992	0.2159031	0.14882956
A2AFQ2	NA	0.89632	0.0508911	0.131763	0.75827932	0.19111388	0.06896703	0.75141056	0.01647188	0.02887792
Q9R0Y5	11636	1.08797	0.59183818	0.41464968	0.5270206	0.02317264	0.01476956	0.58865549	0.07523434	0.07567829
P29758	18242	0.809185	0.0505736	0.131763	0.78965465	0.8314098	0.20467693	0.62622889	0.02256305	0.03480531
Q9R1P0	26441	0.857164	0.50147707	0.37663367	0.73446327	0.00483018	0.00460105	1.16657672	0.93553235	0.38766995
Q61838	11287	0.993816	0.33469665	0.32515718	0.61340779	1.00E-07	5.72E-07	0.9005864	0.22987243	0.15374934
Q04447	12709	1.219567	0.11174602	0.19928419	0.67638208	0.05785173	0.02892164	1.35842291	0.64456009	0.30604704
Q9QWK4	11801	2.122358	0.39603437	0.34332691	0.26314942	0.09048322	0.04097303	0.78970426	0.61429485	0.29947548
P61979	15387	1.511162	0.2788838	0.30302887	0.71247825	0.57829207	0.15441448	0.90096796	0.60809744	0.29724896
Q8R1I1	622178	0.823251	0.00058448	0.01122013	0.86162675	0.8613967	0.20937791	0.85523663	0.8315505	0.3626865
A2AQ07	NA	0.840336	0.55792439	0.39609338	1.84013302	0.35651396	0.10952847	1.90074113	0.424656	0.23641878
P67778	237880	1.677116	0.05606009	0.13940084	0.44234993	0.02613159	0.01606402	0.7948489	0.42641524	0.23675941
Q19LI2	117586	4.959394	4.15E-10	6.64E-08	0.86707199	0.3472268	0.10750767	1.02228743	0.39807353	0.22575549
Q9D1G1	76308	0.857764	0.8964101	0.5091186	0.79099606	0.04523045	0.02421479	1.00655028	0.67487403	0.31593811
Q68FD5	67300	0.985151	0.80659477	0.47932998	0.72105561	0.00086059	0.00115182	0.84106927	0.09946316	0.09004434
OTTMUSF	NA	1.724651	0.01341705	0.06443641	0.66783475	0.00653094	0.00567291	0.55302577	0.03817759	0.04890669
Q91ZJ5	216558	0.758961	0.50308728	0.37671238	0.7307858	3.56E-11	4.18E-10	0.95524534	0.58385668	0.28967093
Q80XN0	71911	1.252199	0.12479539	0.20931915	0.21756573	0.04162807	0.02292212	0.48851326	0.33965635	0.20271436
ENSMUSF	NA	3.246511	0.32324817	0.3192043	0.32813269	0.38823017	0.11610313		NA	NA
Q6P3A8	12040	0.902914	0.45689022	0.35988972	0.62345502	0.96999552	0.23008945	0.78432812	0.68997544	0.32155279
O09131	14873	1.022901	0.96986216	0.52715185	0.69553056	9.72E-05	0.00018858	0.88521262	0.10363285	0.09144394
O08756	NA	1.451248	0.01091758	0.05757766	4.83870167	0.13996117	0.05521915	1.66409741	0.5358275	0.27445423
Q9WTP6	11637	1.625095	0.00691782	0.0468297	0.50319853	0.00773643	0.00641678	0.96539588	0.85947477	0.37002896
O08638	NA	1.168362	0.29150961	0.30856279	0.36426837	0.01790669	0.01233456	0.79948315	0.3771874	0.21728986
Q69ZN8	NA	0.957502	0.53286545	0.38865172	0.72265766	0.52851056	0.14421117	0.82795414	0.30610573	0.18983668
P52503	631040	1.000831	0.47038876	0.362331	0.62224476	5.49E-05	0.00011991	0.77797493	0.00454066	0.01283556
Q91VD9	227197	1.328491	0.31664063	0.31658366	0.42088573	0.00117795	0.0014653	0.65598848	0.01409875	0.02623073
Q61425	15107	0.640236	0.19298217	0.25830548	1.27000977	0.40172615	0.11831848	0.96776237	0.79099562	0.35175981
Q3TLR7-2	NA	0.991071	0.61145957	0.42231146	0.55836363	0.04625125	0.02451306	0.49414898	0.01307992	0.02536171
Q9DCB8	74316	1.003606	0.3383744	0.3260897	0.74664146	0.12567185	0.05071701	0.86706379	0.53587553	0.27445423
P10107	16952	1.195975	0.24780253	0.28725945	0.61829995	0.12832692	0.05132935	1.09687799	0.97587691	0.39717349
Q61735	16423	1.205959	0.19597078	0.25837999	0.83741215	0.61407836	0.16111489	0.83510858	0.12251415	0.10191246
Q8VED5	223917	1.1574	0.2876277	0.30607164		NA	NA	0.28693348	0.80317433	0.35330118
P38060	15356	0.56461	0.85290234	0.49316261	0.78566326	0.00654559	0.00567291	1.00102298	0.11417386	0.09750453
P08226	11816	0.913761	0.72236215	0.45317138	1.18317991	0.72458579	0.18372603	1.4670913	0.81282656	0.35610821
Q7TNS2	433771	1.602593	0.22691395	0.27329476		NA	NA	0.73980848	0.60043664	0.29507046
Q8BMF4	235339	0.776264	0.58523584	0.41182762	0.83328431	0.00083716	0.00113484	1.25904244	0.7230519	0.32958352
P47708	19894	1.620312	0.43970553	0.35466138	0.89594791	0.0950916	0.04240431	0.80680308	0.6579561	0.31078898
Q9WTR5	12554	1.612379	0.03063415	0.10709017	0.49057401	0.09158376	0.04129453	0.36409011	0.01261374	0.02461572
Q9JII6	58810	1.006866	0.63693682	0.42842669	0.63473985	0.00500562	0.00468673	1.01649203	0.26119349	0.16977512

Q922B1	107227	0.857417	0.39847416	0.34332691	0.74036653	0.05259355	0.02700555	0.8505864	0.07857782	0.07702701
Q9DB29	67732	0.709502	0.68649032	0.44358482	1.13461158	0.79833289	0.1983816	1.06577126	0.3130487	0.19250553
Q6PB66	72416	0.730282	0.31006598	0.3148666	0.81558198	0.7725207	0.19310196	0.94742204	0.89338632	0.37706095
Q61207	19156	1.08491	0.68038606	0.44225753	0.36491614	7.21E-05	0.00014803	0.85124732	0.77127015	0.34466911
Q9CPX8	66594	0.969452	0.00471432	0.03834739	0.75554425	6.42E-05	0.00013714	0.84844584	0.05949856	0.0661371
Q8R1G2	69574	0.760936	0.09015658	0.18067452	0.87417948	0.30523094	0.09750289	0.93407795	0.88639976	0.37628982
Q9DB77	67003	1.13544	0.3727459	0.34254523	0.79953057	0.165765	0.06232764	0.83472402	0.70364885	0.32445082
Q9CQ54	675851	0.961233	0.67146446	0.43991358	0.47498821	0.5104658	0.14074611	0.52531921	0.61216404	0.2988362
P62082	20115	1.118324	0.45011098	0.35815034	0.5055213	0.023145	0.01476956	0.65880083	0.10100084	0.09094021
Q922R8	NA	4.777094	0.17782461	0.25371161	0.99591942	0.20883497	0.0734811	1.95941855	0.16159532	0.12225531
Q8BGH2	68653	1.140029	0.40675692	0.3434027	1.0887173	0.93734512	0.22380402	0.29961131	0.03690322	0.0482332
P50247	269378	0.746526	0.55169345	0.39400115	0.80188524	0.00772076	0.00641678	0.82195962	0.0046278	0.01288218
Q91WK1	66701	0.879717	0.22309578	0.2710586	0.67271099	0.00328714	0.00337441	0.8609649	0.11667409	0.09880284
Q9CPY7	66988	1.146123	0.77946917	0.47173129	0.91723545	0.28153891	0.09159505	1.42322805	0.00098294	0.00459533
P62259	22627	0.844008	0.18261499	0.25514656	0.75363379	8.06E-05	0.0001608	0.75798886	3.61E-05	0.00044853
Q8K2C6	68346	0.674697	0.37259132	0.34254523	0.52985688	0.46085898	0.12994325	2.37703588	0.71119545	0.32550019
P37804	21345	2.334646	0.04727203	0.12745391	0.62280623	0.01464728	0.01039412	0.44464794	0.01956065	0.0322758
Q8BWT1	52538	0.958693	0.49557985	0.37396026	0.79110377	0.03080845	0.01835221	1.19475187	0.70225817	0.32445082
Q62188	22240	0.854721	0.5907895	0.4145201	0.7018959	0.65370202	0.16858266	0.74111282	0.48272599	0.25511594
Q78IK2	1E+08	0.920668	0.00035232	0.00805178	0.70284506	0.00373584	0.00374414	0.8411136	0.1890953	0.1359761
Q920B9	NA	0.737481	0.25270822	0.29014295	1.42054087	0.80079282	0.1985263	1.23182855	0.70945882	0.32550019
O88492	57435	0.83649	0.03153548	0.10709017	0.52710378	0.09411283	0.04207602	0.71825751	0.86473901	0.37098139
Q9CQN1	68015	0.991274	0.66199983	0.43882185	0.99822739	0.30872961	0.0981756	0.80202666	0.08552939	0.08153736
P17665	1E+08	1.034581	0.8597879	0.49476261	2.1488595	0.08808651	0.04030464	1.73498384	0.69412816	0.32203503
O35887	12321	0.812996	0.75248914	0.46180902	0.75115145	0.46668137	0.13123496	0.64464848	0.26299372	0.17004046
Q9DBL1	66885	0.923158	0.07802613	0.165154	1.32455209	0.7857785	0.19585925	1.28318693	0.25461854	0.16669433
P16332	17850	0.681925	0.97076205	0.52715185	0.72044152	0.09562725	0.04248363	1.02825359	0.30278288	0.18838632
O55237	21948	0.734697	0.41477965	0.3438528	1.59039213	0.50965639	0.14074611	0.33110389	0.17324313	0.127368
Q9D8W5	66997	1.450972	0.46099783	0.360917	0.80047434	0.50277505	0.13971273	0.88879699	0.6224624	0.3015356
Q9R111	14544	0.78011	0.1764557	0.25278996	4.55325082	0.20208888	0.07194538	1.01960251	0.3229208	0.19633841
Q9CQ92	66437	0.934669	0.03765956	0.11438974	0.87781818	0.43036257	0.12449848	0.79786091	0.04049849	0.05121647
O55023	NA	0.968924	0.35643854	0.33673625	0.8196174	0.30108573	0.09632425	0.43981572	0.25739212	0.16820815
Q9Z126	56744	0.375275	0.20875419	0.26574358	1.60716175	0.95711299	0.22767134	0.52555964	0.13402771	0.10765825
Q9DBG5	66905	1.079547	0.62982048	0.4263237	0.49722173	0.13073589	0.05206516	1.12537508	0.11506216	0.09803355
P20029	14828	1.043601	0.98718144	0.53069746	0.58481024	0.06938693	0.03331736	0.82212955	0.32493304	0.19682642
P50171	14979	0.312123	0.34760789	0.33165807	0.61956181	0.15687285	0.05998872	0.72020373	0.37415519	0.21622625
P01867	NA	3.166231	0.00692805	0.0468297	0.43869224	0.00487516	0.00461074	0.63704436	0.10204218	0.09144394
Q8C5Q4	231413	1.273601	0.01356075	0.06443641	0.63341232	0.00786078	0.0064182	1.35428981	0.10224766	0.09144394
P09528	14319	1.148182	0.21529833	0.26837921	0.65525789	0.10587935	0.04560125	0.79563116	0.41312209	0.23106302
Q8BZF8	226041	1.345034	0.1248372	0.20931915	1.15617062	0.69108738	0.17607666	0.73101743	0.50362395	0.2622471
P10605	13030	0.72452	0.98905668	0.53069746	0.89179809	0.49215993	0.13712334	0.45026479	0.04376429	0.05355372
P50136	NA	1.338832	0.40209564	0.3434027	0.31122547	0.00015894	0.00028402	0.50489943	0.02730908	0.03905287

Q52KG5	668303	1.529796	0.02584043	0.09539495	0.83413243	0.0833206	0.03880998	0.83107585	0.01613305	0.0285585
Q00915	19659	1.051072	0.05605036	0.13940084	0.48533306	0.01457942	0.0103808	0.65641743	0.37676643	0.21728986
Q9Z2I8	20917	0.861656	0.87819596	0.50294019	0.45763456	1.14E-14	2.68E-13	0.72013847	3.04E-05	0.00044029
Q9DAK9	75454	0.932263	0.00129832	0.01823938	0.70359938	0.00863625	0.00686579	0.87044372	0.99651104	0.40331402
B1AR69	NA	1.012209	0.36685966	0.34008223	0.7891358	0.1657776	0.06232764	0.98462463	0.43921559	0.24048584
P70168	16211	1.023389	0.68192722	0.44225753	0.68561506	0.00102855	0.0013233	0.88471881	0.16319801	0.1225935
P48758	12408	1.075487	0.38537812	0.34254523	0.72047545	0.00056094	0.00082375	0.87981238	0.12957428	0.10591773
Q3V118	NA	0.89867	0.06700221	0.15239671	1.01459021	0.16927678	0.06302255	1.3114261	0.06532647	0.06945141
P02089	NA	0.392386	0.54891292	0.39318558	7.06352587	0.00811829	0.00652764	4.26472231	0.79526469	0.35254488
O09061	19170	1.088759	0.93672485	0.52377796	0.62703364	0.24151172	0.08145497	0.66497425	0.64360554	0.30599224
P18525	NA	2.916263	0.0663702	0.1521549	0.58651623	0.2566112	0.08578656	0.70623408	0.41012679	0.23106302
Q91ZD1	107587	2.513381	0.23835205	0.28174861	0.92515496	0.92042972	0.22127347	4.31038802	0.72145746	0.32958352
P62897	672195	0.752409	0.20441076	0.26300491	0.22076245	0.07484582	0.03540845	0.66000285	0.86397485	0.37098139
P00329	11522	1.061306	0.73276709	0.45671382	0.89103609	0.85385738	0.20802356	0.83546192	0.78373994	0.34895874
XP_00147	NA	0.460525	0.0109051	0.05757766	5.68839899	0.83393544	0.20482181	4.6450033	0.46007405	0.24708362
Q6P924	NA	1.026988	0.10978727	0.19807938	2.62717979	0.83342033	0.20482181	17.8391904	0.16591338	0.12397885
P97807	14194	0.797295	0.80750423	0.47932998	0.83987349	0.00015982	0.00028402	1.25534032	0.26510518	0.17049873
Q9CZ30	67059	1.005922	0.24301558	0.28585309	0.76458805	1.87E-05	5.14E-05	0.85327942	0.12781294	0.10521002
P09671	20656	3.160573	0.12381325	0.20931915	0.24393615	0.08973486	0.04072135	0.25564515	0.13843493	0.10950419
Q91ZJ0	71711	0.967507	0.30139186	0.31308227	0.81519443	0.93768009	0.22380402	0.70137043	0.45855099	0.24708362
P38647	NA	1.063239	0.39869304	0.34332691	0.87229214	0.09723017	0.04305717	0.88002034	0.54837475	0.27870683
Q9D967	67881	1.21837	0.26591743	0.29725687	0.57879441	0.00461725	0.00441813	0.52172317	0.11677791	0.09880284
P62932	207215	1.489885	0.70875751	0.44933549	1.81954379	0.93572662	0.22380402	0.7214255	0.28177223	0.1774621
OTTMUSF	NA	2.882952	0.52283842	0.38367073	0.71530047	0.81481297	0.20114334		NA	NA
P62869	NA	2.064025	0.21512439	0.26837921	0.5052266	0.32764211	0.10310444	0.86251328	0.56703503	0.28403019
Q91V61	94280	0.267736	0.08565146	0.17491853	1.91590291	0.61109943	0.16053248	3.20261463	0.83247434	0.3626865
Q8C4V4	50789		NA	NA	0.36902311	0.06011493	0.02991165	0.77057949	0.93024212	0.38664874
ENSMUSF	NA	1.166664	0.19350588	0.25830548	0.7939385	0.60914352	0.1602177		NA	NA
Q8VFZ7	NA		NA	NA	3.33179146	0.23992853	0.08105027		NA	NA

STAB2A. Pathway Analysis of YCL vs. OCL			
**Ingenuity Canonical Pathways**	**pvalue**	**Ratio**	**Molecules**
Mitochondrial Dysfunction	8.415E-14	1.41E-01	SDHB,NDUFA9,UQCR11,CPT1B,NDUFB4,UQCRB,COX2 (includes EG:140540),
			NDUFB10,PDHA1,NDUFS1,PARK7,NDUFS6,COX4I1,SDHA,NDUFV1,ATP5A1,
			ATP5C1,UQCR10,NDUFV2,ATP5B,NDUFA6,CYC1,UQCRC1,COX5B,UQCRQ
Glycolysis I	1.615E-11	2.22E-01	Pkm,PGK1,GPI,TPI1,Gapdh/LOC100042025,ENO1,ENO3,PGAM1,ALDOA,BPGM
Gluconeogenesis I	2.458E-10	1.6E-01	PGK1,GPI,Gapdh/LOC100042025,ENO1,ENO3,PGAM1,ALDOA,BPGM
Actin Cytoskeleton Signaling	1.597E-09	6.72E-02	MYH6,PFN1,CFL1,MYL2,TMSB10/TMSB4X,MAPK3,ACTN2,TLN1,TTN,MYL7,KNG1,
			CFL2,MYL4,VCL,MYL3,MSN
Calcium Signaling	1.547E-08	6.16E-02	CALR,MYH6,MYL2,MAPK3,RYR2,TNNT2,Tpm1,TNNI3,MYL7,Tpm4,MYL4,MYL3,CASQ2
Fatty Acid β-oxidation I	3.739E-08	1.33E-01	HADHB,ECI2,IVD,ACAA2,ACSL1,HADHA
Ketolysis	5.015E-08	2.22E-01	HADHB,BDH1 (includes EG:100037356),OXCT1,HADHA
LXR/RXR Activation	7.473E-08	7.35E-02	KNG1,ALB,HPX,TF,APOA1,APOA2,CLU,FGA,GC,A1BG
RhoA Signaling	1.11E-07	7.89E-02	PFN1,CFL2,MYL2,CFL1,MYL4,TTN,MYL3,MSN,MYL7
ILK Signaling	4.316E-07	5.73E-02	MYH6,TMSB10/TMSB4X,CFL2,MYL2,CFL1,MAPK3,ACTN2,MYL4,VIM,MYL3,MYL7
Protein Kinase A Signaling	5.08E-07	3.91E-02	PLN,YWHAH,MYL2,YWHAB,PDIA3,MAPK3,RYR2,YWHAZ,TNNI3,PYGB,TTN,MYL7,
			YWHAQ,PYGM,MYL4,MYL3
RhoGDI Signaling	7.45E-07	5.08E-02	CFL2,MYL2,CFL1,GDI2,MYL4,ARHGDIA,MYL3,ARHGDIB,MSN,MYL7
Regulation of Actin-based Motility by Rho	8.217E-07	7.87E-02	PFN1,MYL2,CFL1,MYL4,ARHGDIA,MYL3,MYL7
Acute Phase Response Signaling	1.082E-06	5.62E-02	ALB,HPX,FTL,TF,APOA1,MAPK3,APOA2,CP,FGA,FGG
PAK Signaling	2.137E-06	6.6E-02	CFL2,MYL2,CFL1,MAPK3,MYL4,MYL3,MYL7
Glycogen Degradation II	2.195E-06	2.5E-01	PYGM,PGM5,PYGB,MTAP
Ketogenesis	3.329E-06	1.43E-01	HADHB,BDH1 (includes EG:100037356),HADHA
Glycogen Degradation III	4.844E-06	2.22E-01	PYGM,PGM5,PYGB,MTAP
Colanic Acid Building Blocks Biosynthesis	9.759E-06	8.11E-02	GPI,UGP2,MPI
Protein Ubiquitination Pathway	1.181E-05	3.73E-02	PSMB3,HSPA8,PSMA6 (includes EG:246582),CRYAB,HSP90AB1,USP5,PSMA5,
			PSMA4,PSMA2,HSPA5
Ethanol Degradation II	1.302E-05	9.3E-02	ALDH1B1,ALDH2,ACSL1,DHRS4
14-3-3-mediated Signaling	1.435E-05	5.98E-02	YWHAQ,YWHAH,PDIA3,YWHAB,MAPK3,YWHAZ,VIM
Oxidative Ethanol Degradation III	1.811E-05	7.5E-02	ALDH1B1,ALDH2,ACSL1
Rapoport-Luebering Glycolytic Shunt	2.003E-05	2E-01	PGAM1,BPGM
Mitochondrial L-carnitine Shuttle Pathway	3.258E-05	1.36E-01	CPT1B,CPT2,ACSL1
Ethanol Degradation IV	3.258E-05	1.03E-01	ALDH1B1,ALDH2,ACSL1
Cellular Effects of Sildenafil (Viagra)	4.043E-05	4.76E-02	MYH6,MYL2,PDIA3,MYL4,NPPA,MYL3,MYL7
Valine Degradation I	4.193E-05	8.57E-02	HADHB,HIBADH,HADHA
GDP-mannose Biosynthesis	4.349E-05	1.43E-01	GPI,MPI
Huntington's Disease Signaling	7.084E-05	3.8E-02	SDHA,TGM2 (includes EG:21817),HSPA8,CTSD,SDHB,ATP5B,MAPK3,CLTC,HSPA5
Lipid Antigen Presentation by CD1	7.084E-05	1.3E-01	CALR,PDIA3,PSAP
TCA Cycle II (Eukaryotic)	7.084E-05	9.52E-02	SDHA,SUCLA2,SDHB,OGDHL

Glycogen Biosynthesis II (from UDP-D-Gluco	8.272E-05	1.67E-01	UGP2,GBE1
Cell Cycle: G2/M DNA Damage Checkpoint R	8.947E-05	8.33E-02	YWHAQ,YWHAH,YWHAB,YWHAZ
Signaling by Rho Family GTPases	0.0001091	3.57E-02	CFL2,MYL2,CFL1,MAPK3,MYL4,VIM,MYL3,MSN,MYL7
Aryl Hydrocarbon Receptor Signaling	0.0001091	4.35E-02	TGM2 (includes EG:21817),ALDH1B1,CTSD,GSTM1,GSTM5,HSP90AB1,MAPK3
Role of Tissue Factor in Cancer	0.0001283	5.26E-02	P4HB,CFL2,CFL1,MAPK3,FGA,FGG
NRF2-mediated Oxidative Stress Response	0.0001337	4.17E-02	GSTM1,FTL,GSTM5,PRDX1,MAPK3,VCP,TXN,CBR1 (includes EG:100360507)
Sucrose Degradation V (Mammalian)	0.0001452	1E-01	TPI1,ALDOA
Leucine Degradation I	0.0002414	7.69E-02	MCCC1,IVD
Semaphorin Signaling in Neurons	0.0003144	7.69E-02	DPYSL2,CFL2,CFL1,MAPK3
Intrinsic Prothrombin Activation Pathway	0.0003439	8.57E-02	KNG1,FGA,FGG
p70S6K Signaling	0.0003765	4.65E-02	YWHAQ,YWHAH,PDIA3,YWHAB,MAPK3,YWHAZ
Glutathione-mediated Detoxification	0.0004125	6.67E-02	GSTM1,Gsta4,GSTM5
PI3K/AKT Signaling	0.0004319	4.17E-02	YWHAQ,YWHAH,HSP90AB1,YWHAB,MAPK3,YWHAZ
Myc Mediated Apoptosis Signaling	0.0006923	6.67E-02	YWHAQ,YWHAH,YWHAB,YWHAZ
Cdc42 Signaling	0.0008841	3.39E-02	CFL2,MYL2,CFL1,MYL4,MYL3,MYL7
Glutaryl-CoA Degradation	0.0009291	8.33E-02	HADHB,HADHA
IGF-1 Signaling	0.0011359	4.76E-02	YWHAQ,YWHAH,YWHAB,MAPK3,YWHAZ
ERK5 Signaling	0.0012579	6.15E-02	YWHAQ,YWHAH,YWHAB,YWHAZ
LPS/IL-1 Mediated Inhibition of RXR Functio	0.0013243	3.35E-02	ALDH1B1,GSTM1,GSTM5,FABP5,CPT1B,CPT2,FABP4,ACSL1
Coagulation System	0.0013243	7.89E-02	KNG1,FGA,FGG
Noradrenaline and Adrenaline Degradation	0.0013243	5.77E-02	ALDH1B1,ALDH2,DHRS4
Phenylalanine Degradation IV (Mammalian, v	0.0013243	5.13E-02	ALDH2,GOT1
Guanosine Nucleotides Degradation III	0.0013243	9.09E-02	GDA,PNP
Aldosterone Signaling in Epithelial Cells	0.0017194	3.57E-02	HSPA8,CRYAB,HSP90AB1,PDIA3,MAPK3,HSPA5
Clathrin-mediated Endocytosis Signaling	0.0018131	3.57E-02	HSPA8,ALB,TF,APOA1,APOA2,CLTC,CLU
Mevalonate Pathway I	0.0019124	7.14E-02	HADHB,HADHA
Histamine Degradation	0.0019124	6.9E-02	ALDH1B1,ALDH2
S-methyl-5'-thioadenosine Degradation II	0.0020178	1.67E-01	MTAP
Acetyl-CoA Biosynthesis III (from Citrate)	0.0020178	1.25E-01	ACLY
Xanthine and Xanthosine Salvage	0.0020178	1.11E-01	PNP
Tight Junction Signaling	0.002374	3.73E-02	MYH6,MYL2,MYL4,VCL,MYL3,MYL7
Glutathione Redox Reactions I	0.0026498	8.7E-02	GPX3,PRDX6
Fatty Acid α-oxidation	0.0026498	9.52E-02	ALDH1B1,ALDH2
Integrin Signaling	0.0028008	3.38E-02	MYL2,MAPK3,ACTN2,TLN1,VCL,TTN,MYL7
Extrinsic Prothrombin Activation Pathway	0.0037121	1E-01	FGA,FGG
Isoleucine Degradation I	0.0037121	6.67E-02	HADHB,HADHA
Gα12/13 Signaling	0.0049603	3.97E-02	MYL2,MAPK3,MYL4,MYL3,MYL7
Atherosclerosis Signaling	0.007105	3.68E-02	ALB,APOA1,APOA2,CLU,PRDX6
Putrescine Degradation III	0.007105	6.67E-02	ALDH1B1,ALDH2

Nitric Oxide Signaling in the Cardiovascular S	0.007105	4.04E-02	KNG1,PLN,HSP90AB1,RYR2
Cardiomyocyte Differentiation via BMP Rece	0.0090949	1E-01	MYL2,NPPA
Tryptophan Degradation X (Mammalian, via T	0.0090949	6.9E-02	ALDH1B1,ALDH2
NADH Repair	0.0117153	1.11E-01	APOA1BP
Guanine and Guanosine Salvage I	0.0117153	1.11E-01	PNP
Purine Ribonucleosides Degradation to Ribos	0.0117153	5.88E-02	PNP
Adenine and Adenosine Salvage I	0.0117153	1.11E-01	PNP
Formaldehyde Oxidation II (Glutathione-depe	0.0117153	1E-01	ESD
Glutamate Degradation II	0.0117153	1E-01	GOT1
PPARα/RXRα Activation	0.0133289	3.26E-02	HSP90AB1,PDIA3,APOA1,MAPK3,APOA2,ADIPOQ
Hematopoiesis from Pluripotent Stem Cells	0.0151902	4.76E-02	IGKC,Ighg2b,Ighg2a
Primary Immunodeficiency Signaling	0.0173415	4.84E-02	IGKC,Ighg2b,Ighg2a
Phospholipase C Signaling	0.0260841	2.69E-02	TGM2 (includes EG:21817),PEBP1,MYL2,MAPK3,MYL4,MYL3,MYL7
IL-12 Signaling and Production in Macrophag	0.0260841	3.21E-02	ALB,APOA1,MAPK3,APOA2,CLU
Dopamine Degradation	0.0345716	5.41E-02	ALDH1B1,ALDH2
ERK/MAPK Signaling	0.0371409	2.91E-02	YWHAQ,YWHAH,YWHAB,MAPK3,YWHAZ,TLN1
Serotonin Degradation	0.0371409	3.95E-02	ALDH1B1,ALDH2,DHRS4
Pyruvate Fermentation to Lactate	0.0371409	1E-01	LDHA
Phenylethylamine Degradation I	0.0371409	9.09E-02	ALDH2
Tetrahydrobiopterin Biosynthesis I	0.0371409	9.09E-02	SPR (includes EG:20751)
Adenine and Adenosine Salvage III	0.0371409	6.67E-02	PNP
Thyroid Hormone Biosynthesis	0.0371409	1.43E-01	CTSD
Tetrahydrobiopterin Biosynthesis II	0.0371409	7.69E-02	SPR (includes EG:20751)
L-cysteine Degradation I	0.0371409	9.09E-02	GOT1
Axonal Guidance Signaling	0.0429333	2.27E-02	DPYSL2,PFN1,CFL2,MYL2,CFL1,PDIA3,MAPK3,MYL4,MYL3,MYL7
Thrombin Signaling	0.0461967	2.93E-02	MYL2,PDIA3,MAPK3,MYL4,MYL3,MYL7
Hepatic Fibrosis / Hepatic Stellate Cell Activa	0.049735	3.42E-02	MYH6,MYL2,MYL4,MYL3,MYL7

STAB2B. Pathway Analysis of OCR vs. OCL			
**Ingenuity Canonical Pathways**	**pvalue**	**Ratio**	**Molecules**
Mitochondrial Dysfunction	6.79E-18	2.54E-01	NDUFA4,SDHB,NDUFA9,UQCRH,COX6A1,UQCR11,NDUFB5,NDUFB4,UQCRB,
			NDUFB8,COX2 (includes EG:140540),NDUFB10,NDUFS1,NDUFB9,NDUFA5,PARK7,
			NDUFAB1,NDUFS6,NDUFB6,NDUFS2,COX4I1,NDUFA8,ATP5J,SDHA,NDUFV1,
			COX7A2,NDUFB3,ATP5A1,SDHC,NDUFS3,NDUFA13,ATP5C1,NDUFS5 (includes
			EG:100361505),UQCR10,NDUFB11,NDUFV2,ATP5B,NDUFA6,NDUFB7,NDUFA12,
			CYC1,UQCRC1,COX5B (includes EG:100002384),NDUFA7,UQCRQ
TCA Cycle II (Eukaryotic)	3.64E-09	1.67E-01	SDHA,SUCLA2,SDHB,OGDHL,ACO2 (includes EG:11429),SDHC,IDH3B
LXR/RXR Activation	6.67E-09	8.09E-02	ALB,HPX,ECHS1,C3,TF,VTN,CLU,SERPINA1,FGA,GC,A1BG
Fatty Acid β-oxidation I	2.17E-08	1.33E-01	HADHB,ECHS1,ECI2,ACADM,ACAA2,ACSL1
Glycolysis I	1.36E-07	1.11E-01	Pkm,PGK1,ENO3,PGAM1,PFKM
14-3-3-mediated Signaling	6.14E-07	6.84E-02	YWHAQ,YWHAG,YWHAH,PDIA3,YWHAB,YWHAZ,TUBA4A,VIM
Cell Cycle: G2/M DNA Damage Checkp	1.77E-06	1.04E-01	YWHAQ,YWHAG,YWHAH,YWHAB,YWHAZ
Aldosterone Signaling in Epithelial Cells	3.52E-06	4.76E-02	HSPA8,HSPA4,CRYAB,HSP90AB1,PDIA3,HSP90AA1,HSPD1,HSPB1
Myc Mediated Apoptosis Signaling	1.04E-05	8.33E-02	YWHAQ,YWHAG,YWHAH,YWHAB,YWHAZ
PI3K/AKT Signaling	1.07E-05	4.86E-02	YWHAQ,YWHAG,YWHAH,HSP90AB1,YWHAB,YWHAZ,HSP90AA1
Creatine-phosphate Biosynthesis	1.39E-05	2.22E-01	CKMT2,CKM
ERK5 Signaling	1.75E-05	7.69E-02	YWHAQ,YWHAG,YWHAH,YWHAB,YWHAZ
Huntington's Disease Signaling	1.87E-05	3.8E-02	SDHA,ATP5J,TGM2 (includes EG:21817),HSPA8,CTSD,HSPA4,SDHB,ATP5B,CLTC
Protein Ubiquitination Pathway	2.38E-05	3.36E-02	HSPA8,HSPA4,CRYAB,HSP90AB1,USP5,HSP90AA1,HSPD1,PSMA2,HSPB1
Valine Degradation I	2.46E-05	8.57E-02	HADHB,ECHS1,BCKDHB
Arginine Biosynthesis IV	5.64E-05	8.33E-02	OAT,GLUD1
Regulation of Actin-based Motility by Rh	6.56E-05	5.62E-02	PFN1,MYL2,CFL1,ARHGDIA,MYL3
Gluconeogenesis I	9.68E-05	6E-02	PGK1,ENO3,PGAM1
p70S6K Signaling	0.000123	4.65E-02	YWHAQ,YWHAG,YWHAH,PDIA3,YWHAB,YWHAZ
Leucine Degradation I	0.000158	7.69E-02	MCCC1,ACADM
Acute Phase Response Signaling	0.000195	3.93E-02	ALB,HPX,FTL,C3,TF,SERPINA1,FGA
Ketolysis	0.000252	1.11E-01	HADHB,OXCT1
IGF-1 Signaling	0.000394	4.76E-02	YWHAQ,YWHAG,YWHAH,YWHAB,YWHAZ
NRF2-mediated Oxidative Stress Respo	0.000432	3.65E-02	AKR7A2,FTL,PRDX1,STIP1,VCP,TXN,CBR1 (includes EG:100360507)
Hypoxia Signaling in the Cardiovascular	0.00052	6.06E-02	P4HB,HSP90AB1,HSP90AA1,LDHA
RhoA Signaling	0.0006	4.39E-02	PFN1,CFL2,MYL2,CFL1,MYL3
Methylglyoxal Degradation III	0.000801	8.7E-02	AKR7A2,AKR1B1
Xanthine and Xanthosine Salvage	0.001548	1.11E-01	PNP
RhoGDI Signaling	0.002018	3.05E-02	CFL2,MYL2,CFL1,GDI2,ARHGDIA,MYL3
Isoleucine Degradation I	0.00213	6.67E-02	HADHB,ECHS1

Oxidative Ethanol Degradation III	0.00213	5E-02	ACSS1,ACSL1
Nitric Oxide Signaling in the Cardiovasc	0.00265	4.04E-02	HSP90AB1,FLT1,RYR2,HSP90AA1
Ethanol Degradation IV	0.003712	6.9E-02	ACSS1,ACSL1
eNOS Signaling	0.005927	3.29E-02	HSPA8,HSPA4,HSP90AB1,FLT1,HSP90AA1
PAK Signaling	0.006294	3.77E-02	CFL2,MYL2,CFL1,MYL3
Clathrin-mediated Endocytosis Signaling	0.008032	3.06E-02	HSPA8,ALB,TF,CLTC,CLU,SERPINA1
ERK/MAPK Signaling	0.008032	2.91E-02	YWHAQ,YWHAG,YWHAH,YWHAB,YWHAZ,HSPB1
NADH Repair	0.008546	1.11E-01	APOA1BP
Methylglyoxal Degradation I	0.008546	9.09E-02	GLO1 (includes EG:109801)
Guanine and Guanosine Salvage I	0.008546	1.11E-01	PNP
Purine Ribonucleosides Degradation to	0.008546	5.88E-02	PNP
Adenine and Adenosine Salvage I	0.008546	1.11E-01	PNP
Lipid Antigen Presentation by CD1	0.008546	8.7E-02	CALR,PDIA3
Semaphorin Signaling in Neurons	0.009683	5.77E-02	DPYSL2,CFL2,CFL1
Aryl Hydrocarbon Receptor Signaling	0.012493	3.11E-02	TGM2 (includes EG:21817),CTSD,HSP90AB1,HSP90AA1,HSPB1
Triacylglycerol Degradation	0.017342	6.06E-02	Ces1d,PRDX6
Pyruvate Fermentation to Lactate	0.026084	2E-01	Ldhb,LDHA
Adenine and Adenosine Salvage III	0.026084	6.67E-02	PNP
Thyroid Hormone Biosynthesis	0.026084	1.43E-01	CTSD
Protein Kinase A Signaling	0.027967	2.2E-02	YWHAQ,YWHAG,MYL2,YWHAH,PDIA3,YWHAB,RYR2,YWHAZ,MYL3
Role of Tissue Factor in Cancer	0.039922	3.51E-02	P4HB,CFL2,CFL1,FGA

**STAB 2C. Pathway Analysis of ORP vs. OCL**			
**Ingenuity Canonical Pathways**	**p-value**	**Ratio**	**Molecules**
Mitochondrial Dysfunction	1.47E-16	1.64E-01	UQCRH,UQCR11,CPT1B,NDUFB4,UQCRB,NDUFB10,NDUFB9,NDUFS1,
			SOD2,PARK7,NDUFS6,NDUFB6,NDUFS2,OGDH,COX4I1,NDUFA8,SDHA,
			NDUFV1,NDUFB3,ATP5A1,NDUFA13,ATP5C1,NDUFV2,ATP5B,UQCRC2,
			CYC1,UQCRC1,UQCRQ,COX5B (includes EG:100002384)
Fatty Acid β-oxidation I	1.06E-09	1.33E-01	HADHB,ECI2,ACADM,ACAA2,ACSL1,HADHA
Mitochondrial L-carnitine Shuttle Pathway	1E-06	1.36E-01	CPT1B,CPT2,ACSL1
TCA Cycle II (Eukaryotic)	1.77E-06	7.14E-02	SDHA,OGDH,IDH3B
Ethanol Degradation II	9.16E-06	6.98E-02	ALDH2,ACSL1,DHRS4
Glycogen Degradation II	1.81E-05	1.25E-01	PYGM,PYGB
Ketolysis	1.81E-05	1.11E-01	HADHB,HADHA
Ketogenesis	2.55E-05	9.52E-02	HADHB,HADHA
Glutaryl-CoA Degradation	3.38E-05	8.33E-02	HADHB,HADHA
Glycogen Degradation III	3.38E-05	1.11E-01	PYGM,PYGB
Mevalonate Pathway I	5.86E-05	7.14E-02	HADHB,HADHA
Isoleucine Degradation I	9.68E-05	6.67E-02	HADHB,HADHA
Oxidative Ethanol Degradation III	9.68E-05	5E-02	ALDH2,ACSL1
Ethanol Degradation IV	0.000151	6.9E-02	ALDH2,ACSL1
Valine Degradation I	0.000187	5.71E-02	HADHB,HADHA
AMPK Signaling	0.000301	2.41E-02	CKM,PPP2CA,CPT1B,CPT2
Gluconeogenesis I	0.000545	4E-02	PGK1,PGAM1
Glycolysis I	0.000763	4.44E-02	PGK1,PGAM1
Noradrenaline and Adrenaline Degradation	0.002508	3.85E-02	ALDH2,DHRS4
Stearate Biosynthesis I (Animals)	0.002801	4E-02	ACOT2,ACSL1
Phenylethylamine Degradation I	0.003132	9.09E-02	ALDH2
Tetrahydrobiopterin Biosynthesis I	0.003132	9.09E-02	SPR (includes EG:20751)
Tetrahydrobiopterin Biosynthesis II	0.003132	7.69E-02	SPR (includes EG:20751)
Creatine-phosphate Biosynthesis	0.006294	1.11E-01	CKM
2-ketoglutarate Dehydrogenase Complex	0.006294	1E-01	OGDH
Rapoport-Luebering Glycolytic Shunt	0.006294	1E-01	PGAM1
Autoimmune Thyroid Disease Signaling	0.009683	3.28E-02	Ighg2b,Ighg2a
Huntington's Disease Signaling	0.012493	1.69E-02	SDHA,TGM2 (includes EG:21817),ATP5B,HSPA5
Hematopoiesis from Pluripotent Stem Cells	0.01519	3.17E-02	Ighg2b,Ighg2a
Primary Immunodeficiency Signaling	0.016227	3.23E-02	Ighg2b,Ighg2a
Superoxide Radicals Degradation	0.018541	1.25E-01	SOD2
Glycogen Biosynthesis II (from UDP-D-Glucose)	0.018541	8.33E-02	UGP2

Glutamate Degradation III (via 4-aminobutyrate)	0.018541	7.69E-02	SUCLG2
Allograft Rejection Signaling	0.02434	2.11E-02	Ighg2b,Ighg2a
Serotonin Degradation	0.027967	2.63E-02	ALDH2,DHRS4
Aldosterone Signaling in Epithelial Cells	0.039922	1.79E-02	PDIA3,HSPA5,HSPB6
Leucine Degradation I	0.046197	3.85E-02	ACADM

STAB 3. Protein abundance ratio (log2 of fold change)

Protein#	Gene	Entrez	OCL vs. YCL				OCL vs. OCR				OCL vs. ORP			
			Fold	SE	unadj p	q	Fold	SE	unadj p	q	Fold	SE	unadj p	q
A2A9C3	SZT2	230676	-0.089379	0.333744	0.793051	0.523966	-0.90429	0.397767	0.042177	0.074923	-0.214235	0.350009	0.550293	0.565425
A2AKY4	Z804A	241514	0.152872	0.439402	0.730709	0.500009	-0.426078	0.464594	0.367521	0.287106	0.22738	0.504433	0.656206	0.603589
A2AMM0	MURC	68016	0.086458	0.307355	0.780709	0.518804	-0.001926	0.376978	0.995962	0.483535	0.084341	0.386116	0.828938	0.647942
A2AQP0	MYH7B	668940	0.097434	0.414986	0.816212	0.531384	-0.300207	0.423487	0.484699	0.332855	0.199338	0.434293	0.650369	0.600663
A2AR50	RGPS1	241308	1.810828	0.923307	0.07163	0.120198	1.052305	0.938946	0.28126	0.244756	-0.254631	0.983108	0.7994	0.635775
A2ASS6	TITIN	22138	0.621704	0.167531	0.000245	0.001487	-0.070704	0.166871	0.672096	0.393016	-0.401511	0.19946	0.045101	0.17308
A3KG59	P20D2	242377	-0.404905	0.115683	0.001696	0.006653	-0.203952	0.088762	0.029875	0.058785	-0.096328	0.10522	0.369037	0.481758
A3KMP2	TTC38	239570	-0.155689	0.420929	0.714474	0.499195	0.007216	0.403395	0.985865	0.482516	0.085055	0.440452	0.848499	0.65282
A6H6E2	MMRN2	105450	0.324914	0.325073	0.32676	0.326525	0.440364	0.212008	0.048219	0.080696	-0.150877	0.440971	0.735219	0.616276
B2RY56	RBM25	67039	-0.43888	0.461275	0.350138	0.338084	-0.26074	0.399174	0.519362	0.34496	0.249164	0.192479	0.207811	0.357798
O08528	HXK2	15277	-0.100501	0.186855	0.591882	0.452213	-0.146812	0.212323	0.490851	0.334808	-0.28538	0.234651	0.226959	0.369779
O08553	DPYL2	12934	-0.641657	0.132798	3.62E-06	4.83E-05	-0.520909	0.115834	1.44E-05	0.000145	-0.168763	0.103174	0.104301	0.257723
O08600	NUCG	13804	0.176726	0.243622	0.477525	0.400653	0.26892	0.2698	0.330238	0.270155	0.219715	0.278935	0.44239	0.51859
O08638	MYH11		-0.252638	0.515834	0.631386	0.46488	-0.934557	0.707299	0.209184	0.211202	-0.354115	0.66958	0.607408	0.588067
O08677	KNG1	16644	-0.762141	0.118157	1.22E-09	4.32E-08	0.024264	0.100074	0.808687	0.429118	0.156683	0.114527	0.173103	0.328086
O08709	PRDX6		-0.268917	0.086695	0.002123	0.008035	-0.384648	0.082338	4.64E-06	5.14E-05	0.006844	0.067188	0.918945	0.673601
O08749	DLDH	13382	0.037626	0.065969	0.568783	0.441151	0.088541	0.076013	0.244849	0.228322	0.045018	0.07743	0.56135	0.56781
O08756	HCD2		0.130408	0.22488	0.565805	0.440121	0.308255	0.191935	0.117793	0.149071	0.22252	0.173345	0.207686	0.357798
O08807	PRDX4	53381	-0.049985	0.206223	0.820405	0.531384	-0.487897	0.189499	0.036757	0.069577	0.01508	0.184779	0.938122	0.677584
O08997	ATOX1	11927	-0.260554	0.13424	0.056472	0.10215	-0.033236	0.127543	0.795154	0.425862	-0.13087	0.153116	0.395901	0.499827
O09061	PSB1	19170	-0.327937	0.28217	0.263313	0.286167	-0.270434	0.223335	0.242503	0.228322	0.063215	0.375786	0.869763	0.658835
O09111	NDUBB	104130	0.21356	0.157566	0.181158	0.225862	0.4449	0.140306	0.002548	0.009386	0.371416	0.189163	0.055523	0.191194
O09131	GSTO1	14873	-0.304133	0.221757	0.177343	0.222854	0.13936	0.190071	0.467325	0.327525	0.096449	0.201419	0.63472	0.598255

O09161	CASQ2	12373	-0.23127	0.259424	0.374295	0.350015	-0.360763	0.243653	0.141198	0.169951	-0.148116	0.261112	0.57162	0.571663
O35129	PHB2	12034	0.381552	0.132457	0.004839	0.015745	0.251967	0.165672	0.131415	0.162136	0.040062	0.191249	0.834533	0.64913
O35215	DOPD	13202	-0.067295	0.067336	0.319105	0.32189	0.101184	0.059022	0.088391	0.122349	-0.105246	0.062076	0.092096	0.237757
O35459	ECH1	51798	0.133593	0.107188	0.214038	0.252129	-0.135842	0.109572	0.216498	0.214531	0.245316	0.118889	0.040372	0.158759
O35593	PSDE	59029	-0.039984	0.382842	0.918043	0.561149	-0.239209	0.495237	0.636548	0.382048	0.46264	0.544316	0.408716	0.505969
O35639	ANXA3	11745	-0.661011	0.136119	4.91E-05	0.000397	-0.587912	0.142043	0.000325	0.001973	-0.765254	0.638607	0.242493	0.38063
O35643	AP1B1	11764	-0.726213	0.885309	0.443409	0.385023	0.059313	1.065045	0.957144	0.476372	1.286709	1.010015	0.243345	0.38063
O35658	C1QBP		-0.128262	0.132445	0.336926	0.329888	-0.023541	0.145559	0.872074	0.450203	0.202589	0.142843	0.161966	0.319316
O35678	MGLL	23945	-0.018727	0.346849	0.957537	0.572822	0.442813	0.313107	0.173465	0.19065	0.309958	0.36091	0.401731	0.501808
O35683	NDUA1	54405	0.052845	0.156069	0.737631	0.50334	0.32538	0.168465	0.064397	0.098699	0.273916	0.145935	0.072731	0.218206
O35841	API5	11800	0.527606	0.351006	0.149246	0.201856	-0.311342	0.389846	0.434917	0.31432	-0.140652	0.506039	0.785116	0.62977
O35855	BCAT2	12036	-0.108939	0.097906	0.267293	0.288146	0.068146	0.096812	0.48238	0.332026	-0.250395	0.099301	0.012586	0.072891
O35887	CALU	12321	-0.749505	0.180226	9.44E-05	0.000699	-0.482245	0.137533	0.000766	0.003609	-0.233484	0.18031	0.199797	0.349671
O35943	FRDA	14297	0.528792	0.194909	0.01885	0.04572	0.020645	0.204821	0.921142	0.466829	0.31034	0.158092	0.071401	0.217637
O35963	RB33B	19338	-0.186497	0.125175	0.148284	0.201853	-0.242624	0.147906	0.112969	0.144203	-0.006497	0.127252	0.959702	0.679307
O54724	PTRF	19285	-0.166161	0.160829	0.311424	0.315857	-0.225173	0.171321	0.20021	0.207412	-0.056652	0.15771	0.722574	0.612491
O55023	IMPA1		-0.086482	0.417316	0.837276	0.537519	-0.249058	0.369936	0.505944	0.341876	-0.100123	0.382744	0.795622	0.634269
O55103	PRAX	19153	0.517495	0.254691	0.059117	0.106215	0.153377	0.298926	0.614126	0.377805	0.418224	0.225922	0.085352	0.230229
O55126	NIPS2		0.116185	0.092445	0.209829	0.248818	0.002578	0.095862	0.978565	0.481108	0.22788	0.104622	0.030256	0.13158
O55143	AT2A2	11938	-0.031603	0.090354	0.726692	0.499208	-0.024367	0.104319	0.815428	0.430497	0.006682	0.103038	0.948326	0.677987
O55237	CD70	21948	0.622866	0.652549	0.351812	0.338084	0.645699	0.552603	0.254587	0.231553	0.094877	0.605594	0.877003	0.6598
O70250	PGAM2	56012	-0.137254	0.199412	0.492263	0.408078	-0.280011	0.178937	0.119643	0.150981	-0.00618	0.150412	0.967285	0.681039
O70251	EF1B	55949	-0.305468	0.164719	0.076	0.125797	-0.638117	0.378697	0.105505	0.137964	-0.679329	0.243806	0.011396	0.069139
O70370	CATS	13040	-0.923375	0.377234	0.02373	0.053656	-0.377615	0.399931	0.354881	0.283285	-0.647743	0.355152	0.080153	0.224939
O70433	FHL2	14200	0.80562	0.37903	0.037027	0.075702	0.68126	0.395101	0.089137	0.122481	0.029146	0.495362	0.953262	0.677987
O70456	1433S	55948	0.110917	0.305543	0.719528	0.499195	-0.096231	0.293336	0.745495	0.413574	0.235446	0.323941	0.474367	0.529163

O70468	MYPC3		0.716962	0.11774	2.83E-09	8.87E-08	0.215554	0.106418	0.04354	0.075276	-0.047083	0.130705	0.718904	0.611451
O88342	WDR1	22388	-0.300755	0.063646	4.65E-06	5.90E-05	-0.168404	0.05766	0.003948	0.012764	-0.162111	0.060239	0.007854	0.056218
O88441	MTX2	53375	0.481732	0.226	0.037611	0.076311	0.377791	0.375178	0.318362	0.265564	0.537056	0.277602	0.058485	0.19405
O88492	PLIN4	57435	-0.182151	0.209848	0.389706	0.358442	-0.113178	0.200371	0.574708	0.364442	-0.531407	0.407441	0.198635	0.348596
O88587	COMT		0.443519	0.204108	0.095493	0.148997	-0.301165	0.191586	0.176765	0.192847	-0.115344	0.331789	0.745631	0.621122
O88668	CREG1	433375	-2.066015	0.527546	0.002049	0.007866	-0.015386	0.186899	0.935644	0.469877	0.194635	0.151044	0.214799	0.362091
O88685	PRS6A	19182	-0.073277	0.204474	0.721266	0.499195	-0.161034	0.226876	0.48063	0.332026	0.238839	0.210851	0.261986	0.40202
O88799	ZAN		-0.376087	0.226364	0.110199	0.16518	-0.399292	0.150733	0.014659	0.035262	-0.269569	0.166021	0.122835	0.276824
O88844	IDHC	15926	-0.295877	0.102624	0.005153	0.016464	-0.034616	0.088441	0.696671	0.401799	0.096869	0.090311	0.287409	0.421874
O89053	COR1A	12721	-1.323007	1.197784	0.292932	0.30307	-1.578031	1.169152	0.206865	0.21064	-1.627672	0.869698	0.094068	0.241636
P00158	CYB	17711	0.171268	0.182141	0.355718	0.340174	0.272892	0.207726	0.200417	0.207412	0.302209	0.191138	0.126945	0.279943
P00329	ADH1	11522	0.943258	0.777316	0.235855	0.267779	-0.059914	0.653702	0.927583	0.467952	-0.63005	0.884117	0.482672	0.530665
P00397	COX1		0.053318	0.081881	0.516828	0.420352	0.131724	0.120202	0.276274	0.242802	0.191168	0.076433	0.014532	0.079124
P00405	COX2	17709	0.188326	0.074332	0.01259	0.033591	0.179301	0.078062	0.023328	0.04944	0.193179	0.099704	0.0552	0.191194
P00493	HPRT	15452	-0.844479	0.718002	0.252696	0.279349	-0.522417	0.639513	0.422022	0.308465	-0.784773	0.574259	0.186924	0.337335
P00920	CAH2	12349	0.132816	0.065276	0.042827	0.082802	-0.254821	0.078846	0.001379	0.005762	-0.322895	0.077931	4.67E-05	0.001102
P01027	CO3	12266	-0.219476	0.098911	0.027266	0.060371	-0.56686	0.115336	1.45E-06	2.01E-05	0.049713	0.102122	0.626782	0.596846
P01680	KV4A1		-0.887879	0.420848	0.050983	0.095457	-0.615974	0.478597	0.217587	0.214634	-0.534269	0.474596	0.279206	0.417382
P01837	IGKC		-1.387798	0.348021	0.000151	0.000993	-1.55664	0.456824	0.001105	0.004847	-0.779697	0.510385	0.131374	0.285637
P01843	LAC1		-0.025175	0.71697	0.972292	0.575201	-0.766525	0.729004	0.306234	0.26035	0.08582	0.79683	0.915361	0.673359
P01864	GCAB		-1.318468	0.523058	0.01509	0.038344	-0.899909	0.531475	0.096893	0.128877	-2.103541	0.505199	0.000129	0.002288
P01867	IGG2B		-1.51157	0.26941	3.12E-07	5.21E-06	-0.878773	0.333578	0.010162	0.026793	-1.725138	0.353541	6.06E-06	0.000193
P01872	IGHM		-0.546286	0.805558	0.511693	0.418785	0.033446	0.84622	0.969123	0.479946	-0.732518	0.773026	0.359405	0.478992
P01942	HBA		0.400007	0.112232	0.000422	0.002125	0.031619	0.103522	0.760242	0.416757	-0.076913	0.112273	0.493888	0.538902
P02088	HBB1		0.152949	0.099273	0.124721	0.178427	-0.066486	0.098762	0.501519	0.340186	-0.25526	0.098678	0.010376	0.066099
P02089	HBB2		0.118266	0.417873	0.779491	0.518804	-0.45918	0.321654	0.165791	0.185603	-0.840175	0.403431	0.0486	0.179718

P02535	K1C10		0.593383	0.551617	0.288837	0.302212	-0.849884	0.475619	0.083431	0.118444	-1.020745	0.455926	0.032027	0.136932
P03888	NU1M		-0.064671	0.245175	0.792485	0.523966	-0.079243	0.243952	0.746032	0.413574	-0.074185	0.249204	0.76665	0.629091
P03893	NU2M		0.043302	0.206625	0.835701	0.537282	-0.049846	0.483065	0.918638	0.466093	0.094527	0.234076	0.690067	0.60803
P03903	NU4LM	17720	0.066964	0.163555	0.685858	0.485308	0.203359	0.18777	0.289133	0.249645	-0.058788	0.1316	0.659447	0.603589
P03911	NU4M	17719	0.195712	0.138851	0.162562	0.212715	0.238469	0.178026	0.184243	0.195702	0.19234	0.179018	0.286175	0.421033
P03921	NU5M		0.168559	0.162988	0.304207	0.310382	-0.003846	0.180397	0.983047	0.481892	0.263409	0.183298	0.155157	0.312793
P03930	ATP8	17706	0.331574	0.179018	0.067735	0.116747	0.102003	0.225202	0.651851	0.38715	0.36021	0.182257	0.05189	0.185708
P03976	KV2A5		1.175872	1.191441	0.333522	0.32897	1.074833	1.110928	0.343354	0.276517	1.702476	1.213163	0.176649	0.328086
P03987	IGHG3		1.199454	0.410337	0.004416	0.014679	-0.092165	0.429464	0.830658	0.437098	-0.806896	0.435526	0.068331	0.2142
P04104	K2C1	16678	-0.689485	0.404776	0.100424	0.155779	-0.722699	0.307934	0.026822	0.054497	-0.670081	0.223592	0.006437	0.047135
P04117	FABP4	11770	-0.209534	0.055756	0.000228	0.001417	-0.063713	0.055739	0.254467	0.231553	-0.004769	0.054631	0.930545	0.675943
P04247	MYG	17189	-0.008748	0.067502	0.896928	0.555883	-0.084445	0.075741	0.265276	0.237375	0.093048	0.071606	0.194262	0.34665
P05064	ALDOA	11674	-0.468075	0.053907	8.24E-17	1.10E-14	-0.044432	0.053096	0.403146	0.300621	-0.106977	0.054136	0.048817	0.179718
P05125	ANF		-1.064629	0.314368	0.000922	0.004101	-0.459281	0.343399	0.183274	0.195611	-0.682685	0.350803	0.053772	0.188216
P05201	AATC	14718	-0.188087	0.042587	1.18E-05	0.000129	0.042414	0.045197	0.348386	0.279897	-0.012833	0.046276	0.781645	0.629514
P05202	AATM	14719	0.019881	0.05829	0.733169	0.500936	0.084043	0.069518	0.227157	0.217314	0.127858	0.068747	0.063436	0.206183
P06151	LDHA	16828	-0.545973	0.060892	1.90E-17	3.38E-15	-0.503066	0.068404	1.41E-12	6.93E-11	-0.032559	0.077276	0.673788	0.605945
P06537	GCR		0.307764	0.15962	0.193648	0.234685	0.445595	NaN	NaN	NA	0.544782	0.46174	0.447595	0.518689
P06728	APOA4	11808	-0.288699	0.245988	0.247316	0.274942	-0.194486	0.38898	0.619756	0.378182	0.33456	0.367645	0.369043	0.481758
P06745	G6PI	14751	-0.201589	0.06605	0.002425	0.008913	0.035211	0.063832	0.581518	0.366918	0.041988	0.069929	0.548581	0.565425
P06801	MAOX	17436	-0.345481	0.2492	0.171553	0.219073	0.269827	0.210008	0.204436	0.209128	0.315557	0.172921	0.074121	0.219434
P06909	CFAH		-0.568521	0.323967	0.091531	0.144078	-0.665386	0.338016	0.060193	0.09421	-0.275088	0.452936	0.549326	0.565425
P07309	TTHY	22139	0.244238	0.165019	0.145825	0.200038	-0.410976	0.207064	0.053153	0.086055	-0.538593	0.200175	0.010043	0.065957
P07310	KCRM	12715	0.030021	0.04882	0.538891	0.427823	-0.193938	0.052373	0.000238	0.001611	-0.285219	0.055664	4.50E-07	2.39E-05
P07356	ANXA2	12306	-0.364969	0.083022	1.52E-05	0.000152	-0.432389	0.09028	2.60E-06	3.28E-05	-0.411634	0.112447	0.000299	0.004435
P07724	ALBU	11657	-0.212788	0.036495	6.78E-09	1.90E-07	-0.335317	0.034642	1.56E-21	3.45E-19	-0.329076	0.033737	8.55E-22	2.72E-19

P07759	SPA3K	20714	0.580303	0.22208	0.013014	0.034378	0.6914	0.212576	0.002327	0.008769	-0.329315	0.283491	0.253973	0.394617
P07901	HS90A	15519	-0.349753	0.15038	0.022277	0.051683	-0.638001	0.1713	0.000361	0.00213	-0.150335	0.184726	0.417941	0.509209
P08003	PDIA4	12304	-1.034539	0.50947	0.048365	0.091844	-0.720577	0.525159	0.177313	0.19297	-0.967314	0.595224	0.112625	0.264484
P08074	CBR2	12409	-1.536717	0.353096	0.0002	0.001254	-1.748822	0.66013	0.01354	0.033134	-0.578982	0.414919	0.176221	0.328086
P08113	ENPL	22027	-0.25983	0.327161	0.42902	0.378948	-0.284589	0.198293	0.154449	0.177691	-0.000854	0.173772	0.996088	0.686749
P08226	APOE	11816	0.612895	0.615609	0.328625	0.326893	-1.173678	0.681341	0.096837	0.128877	-0.313521	0.671462	0.644948	0.598925
P08228	SODC	20655	-0.187903	0.136288	0.170443	0.218633	-0.179477	0.145718	0.220365	0.214634	0.051373	0.150861	0.734054	0.616276
P08249	MDHM	17448	-0.076008	0.044908	0.091314	0.144078	0.051212	0.043654	0.241429	0.228013	0.117483	0.045797	0.010698	0.066813
P08752	GNAI2	14678	-0.31291	0.200675	0.131018	0.18594	-0.305284	0.200951	0.14078	0.169909	-0.328327	0.217561	0.14432	0.299533
P09103	PDIA1	18453	-0.965053	0.170516	6.37E-08	1.20E-06	-0.422961	0.1662	0.011826	0.030455	-0.284906	0.185521	0.126491	0.279943
P09405	NUCL	17975	-0.115677	0.167444	0.496294	0.409955	-0.289123	0.170881	0.104772	0.137964	-0.364245	0.123962	0.007382	0.053441
P09411	PGK1	18655	-0.232368	0.032924	8.30E-12	4.03E-10	-0.120494	0.034657	0.000567	0.002823	-0.094648	0.031655	0.002989	0.025444
P09528	FRIH	14319	-0.274939	0.124079	0.027957	0.061645	-0.14123	0.108486	0.194678	0.202895	0.388844	0.09641	8.45E-05	0.001683
P09541	MYL4		-1.713641	0.367736	6.07E-06	7.54E-05	-0.668352	0.354363	0.060836	0.094549	-0.845445	0.371221	0.024027	0.112545
P09542	MYL3	17897	0.715079	0.105698	9.94E-11	4.08E-09	0.560849	0.110465	7.75E-07	1.14E-05	0.05484	0.115866	0.636465	0.598255
P09671	SODM	20656	-0.100227	0.12944	0.439842	0.383508	0.089156	0.113122	0.43172	0.313998	0.266235	0.095189	0.005814	0.04409
P09813	APOA2		1.23885	0.31354	0.003349	0.011653	1.062424	0.438815	0.027727	0.055853	0.447199	1.142862	0.702436	0.609654
P10107	ANXA1	16952	-0.22306	0.271364	0.412238	0.36724	-0.124679	0.282887	0.659955	0.38924	-0.220767	0.314678	0.483976	0.530665
P10126	EF1A1	13627	-1.424051	0.302683	1.58E-05	0.000152	-0.858893	0.251537	0.001122	0.004873	-0.823726	0.267787	0.00322	0.026988
P10493	NID1	18073	1.233166	0.550173	0.034119	0.0719	0.430918	0.60137	0.480561	0.332026	0.017464	0.764296	0.981967	0.685835
P10605	CATB	13030	-0.613167	0.529805	0.25807	0.283355	0.143698	0.581088	0.806787	0.428999	0.31159	0.678947	0.650591	0.600663
P10639	THIO	22166	-0.289353	0.07824	0.000402	0.0021	-0.43563	0.108589	0.000139	0.001058	-0.039584	0.089495	0.659579	0.603589
P10649	GSTM1	14862	-0.462974	0.141088	0.001268	0.005204	0.014298	0.121629	0.906569	0.462483	0.211661	0.137124	0.124814	0.279943
P11087	CO1A1	12842	-0.684397	0.518333	0.198214	0.237861	-0.733245	0.516498	0.16759	0.186982	-0.646847	0.600588	0.292638	0.425626
P11352	GPX1		0.052905	0.098388	0.592368	0.452213	-0.254829	0.130616	0.054898	0.087785	-0.157694	0.110928	0.159926	0.317385
P11404	FABPH	14077	0.026313	0.060173	0.662486	0.472612	-0.070259	0.055741	0.209324	0.211202	0.171353	0.065993	0.010361	0.066099

P11438	LAMP1	16783	-0.469679	0.191445	0.022544	0.05196	-0.35451	0.194204	0.079902	0.115659	0.228265	0.187311	0.235334	0.373712
P11499	HS90B	15516	-0.301356	0.12834	0.019692	0.047547	-0.969799	0.154455	1.59E-09	4.71E-08	-0.267272	0.149103	0.074402	0.219434
P12246	SAMP	20219	-0.966381	0.432203	0.034917	0.073068	0.744784	0.380003	0.060803	0.094549	0.188443	0.430479	0.665481	0.605694
P12367	KAP2		0.234349	0.250626	0.358365	0.342092	0.05197	0.29537	0.861752	0.446959	-0.684776	0.551366	0.226251	0.369571
P12382	K6PL	18641	0.211037	0.17687	0.243575	0.273422	0.066662	0.155483	0.671641	0.393016	0.20818	0.137729	0.143711	0.299533
P12710	FABPL	14080	0.870335	0.532953	0.111426	0.165623	-0.383424	0.630768	0.547436	0.357113	0.185396	0.555093	0.740635	0.619186
P12787	COX5A	12858	0.246418	0.126294	0.052351	0.097336	0.083093	0.151668	0.584368	0.367669	0.253234	0.164483	0.125284	0.279943
P12979	MYOG	17928	0.390765	0.173474	0.032951	0.070615	0.590618	0.153633	0.000701	0.003374	0.467791	0.166719	0.009795	0.064998
P13020	GELS	227753	-0.75487	0.495093	0.13855	0.193036	-1.102473	0.699321	0.129185	0.160125	-1.053982	0.608231	0.098513	0.248869
P13634	CAH1		0.240062	0.090855	0.008796	0.025509	0.30014	0.097565	0.002347	0.008769	-0.15049	0.098635	0.128557	0.281433
P13707	GPDA	14555	-0.269666	0.147596	0.071468	0.120198	0.407577	0.205589	0.050815	0.083363	-0.186327	0.197982	0.34966	0.468948
P14069	S10A6	20200	-0.883064	0.218253	0.000264	0.001545	-0.052876	0.2129	0.805095	0.428844	0.072655	0.241227	0.764913	0.629091
P14094	AT1B1	11931	0.508237	0.273996	0.067388	0.116747	1.039958	0.242816	5.21E-05	0.000435	0.376534	0.280665	0.184008	0.335879
P14131	RS16	20055	0.013006	0.261197	0.960717	0.573303	-0.268526	0.36204	0.466888	0.327525	-0.130514	0.253869	0.612308	0.589967
P14152	MDHC	17449	-0.057099	0.052338	0.275898	0.293274	-0.052223	0.053686	0.331219	0.270183	0.098018	0.071988	0.174083	0.328086
P14211	CALR	12317	-0.547556	0.150984	0.000428	0.002131	-0.55763	0.181429	0.002593	0.009386	-0.141834	0.173951	0.416622	0.509209
P14602	HSPB1	15507	-0.008365	0.090396	0.926426	0.564318	-0.560575	0.113654	2.67E-06	3.28E-05	-0.056978	0.113778	0.617502	0.591898
P14685	PSMD3	22123	0.158134	1.244758	0.901201	0.556918	0.962508	0.802651	0.250369	0.229602	1.318427	0.89213	0.16521	0.320875
P14824	ANXA6	11749	-0.192825	0.0429	8.77E-06	0.000102	-0.305911	0.050689	3.15E-09	8.21E-08	-0.071478	0.053262	0.180272	0.332761
P14869	RLA0	11837	0.041036	0.182366	0.823723	0.532145	-0.302318	0.230633	0.201378	0.207921	-0.028313	0.182922	0.878286	0.6598
P15327	PMGE	12183	0.570948	0.183804	0.00339	0.011671	-0.048186	0.191083	0.802137	0.428584	-0.027406	0.182265	0.881254	0.66125
P15626	GSTM2	14863	-0.574671	0.087908	9.57E-09	2.55E-07	0.083471	0.071721	0.24828	0.228633	0.215733	0.082418	0.010907	0.067457
P16015	CAH3	12350	0.033362	0.640054	0.95869	0.57287	0.299549	0.546611	0.586518	0.368498	0.157788	0.572022	0.784055	0.62977
P16045	LEG1	16852	-0.552369	0.115404	1.40E-05	0.000147	-0.346684	0.119264	0.005391	0.016377	-0.194446	0.122933	0.120148	0.274772
P16125	LDHB	16832	-0.06296	0.073387	0.391488	0.358442	-0.205525	0.07908	0.009726	0.026109	-0.009443	0.079016	0.90494	0.668007
P16332	MUTA	17850	-0.153504	0.174533	0.381022	0.355454	-0.427993	0.216993	0.051221	0.083531	-0.116084	0.220663	0.600065	0.584511

P16546	SPTA2	20740	0.117957	0.204182	0.568632	0.441151	-0.332454	0.192912	0.0967	0.128877	-0.409334	0.22573	0.08344	0.227924
P16858	G3P	14433	-0.352156	0.097129	0.000314	0.001745	-0.166409	0.090809	0.067395	0.101883	-0.138009	0.098566	0.162043	0.319316
P17182	ENOA	13806	-0.35061	0.054721	1.50E-09	5.02E-08	-0.069651	0.057942	0.231014	0.220527	0.000644	0.060415	0.991504	0.686749
P17183	ENOG	13807	0.119612	0.140519	0.402422	0.362735	-0.264376	0.155701	0.101451	0.134139	-0.253548	0.158598	0.122975	0.276824
P17563	SBP1	20341	-0.138624	0.106005	0.194717	0.235078	-0.13688	0.097168	0.162617	0.183748	-0.146808	0.117748	0.216205	0.362525
P17665	COX7C	12867	0.245079	0.503285	0.63053	0.46488	0.400101	0.516953	0.446213	0.318268	-0.215324	0.72004	0.76748	0.629091
P17710	HXK1	15275	-0.196089	0.133864	0.144992	0.199407	-0.049775	0.150735	0.74168	0.413182	-0.051025	0.160784	0.75142	0.624108
P17742	PPIA	268373	-0.377291	0.066572	6.54E-08	1.20E-06	-0.310295	0.084052	0.000305	0.001927	-0.079168	0.087145	0.3651	0.481545
P17751	TPIS	21991	-0.32883	0.08136	6.96E-05	0.000546	-0.086644	0.076861	0.260652	0.235482	-0.092855	0.07958	0.244435	0.380726
P17950	IER2		0.373782	0.402974	0.373541	0.350015	0.472823	0.314051	0.15804	0.180418	-0.133633	0.493947	0.790691	0.632005
P18242	CATD	13033	-0.541829	0.085868	5.06E-08	9.99E-07	-0.43268	0.087388	5.82E-06	6.14E-05	-0.029989	0.074102	0.687092	0.606591
P18525	HVM54		-0.198443	0.436697	0.651713	0.470588	-1.5921	0.641024	0.017078	0.039194	0.160778	0.409578	0.696595	0.608215
P18572	BASI	12215	0.001223	0.106197	0.990855	0.578391	0.059483	0.116552	0.611963	0.376998	0.106396	0.10618	0.321353	0.449734
P18760	COF1	12631	-0.538903	0.134045	9.75E-05	0.000711	-0.465135	0.123395	0.000246	0.001611	-0.244152	0.13515	0.073301	0.218206
P18826	KPB1	18679	-0.051003	0.824733	0.951223	0.571644	0.195058	0.815989	0.8131	0.43029	-0.235723	0.852459	0.784617	0.62977
P18872	GNAO	14681	-0.159088	0.355844	0.658672	0.471852	-0.638021	0.410075	0.13231	0.162341	-0.475897	0.387869	0.232251	0.371747
P19096	FAS	14104	-0.147706	0.424363	0.728793	0.499866	0.150782	0.50189	0.764693	0.41797	-0.10009	0.443835	0.822248	0.645528
P19123	TNNC1	21924	0.017037	0.098193	0.862462	0.540169	-0.113192	0.108517	0.298454	0.256195	-0.094723	0.109475	0.388251	0.49566
P19157	GSTP1	14870	-0.285445	0.122185	0.023297	0.052901	-0.19932	0.113446	0.0847	0.11948	0.169441	0.114355	0.144818	0.299533
P19536	COX5B		0.377639	0.104771	0.000405	0.0021	0.461625	0.109129	3.71E-05	0.000323	0.40159	0.117307	0.00078	0.009556
P19783	COX41	12857	0.27907	0.081602	0.000888	0.004017	0.280764	0.092153	0.002914	0.009934	0.301717	0.08366	0.000488	0.006764
P20029	GRP78	14828	-0.446403	0.116482	0.000177	0.00115	-0.002078	0.100344	0.983502	0.481892	-0.342752	0.109468	0.002055	0.021047
P20065	TYB4	19241	-0.476434	0.167479	0.00855	0.025069	-0.778467	0.352305	0.036141	0.069299	-0.156378	0.228285	0.499899	0.541598
P20108	PRDX3	11757	-0.035469	0.087042	0.684264	0.484906	0.068674	0.10743	0.523671	0.346717	0.233396	0.097086	0.017564	0.089511
P20152	VIME	22352	-0.727681	0.127386	2.77E-08	5.91E-07	-0.9286	0.142404	3.16E-10	9.99E-09	-0.389865	0.129495	0.002854	0.024903
P21107	TPM3	59069	-0.405597	0.326885	0.260995	0.285391	-0.333703	0.538665	0.552816	0.360093	-0.059186	0.325946	0.863041	0.657432

P21300	ALD1	11997	-0.034458	0.429506	0.936993	0.566245	0.333445	0.410133	0.426832	0.310954	0.39324	0.390039	0.328361	0.454743
P21550	ENOB	13808	0.158049	0.036535	2.06E-05	0.00019	-0.116109	0.046631	0.013305	0.03274	-0.140511	0.055407	0.011754	0.070638
P21614	VTDB	14473	-0.40642	0.110161	0.000385	0.002047	-0.438296	0.168642	0.010841	0.028245	0.047145	0.16331	0.773494	0.629514
P21956	MFGM	17304	0.091374	0.146697	0.538793	0.427823	0.069152	0.169261	0.686215	0.397218	-0.110405	0.392826	0.781078	0.629514
P21981	TGM2	21817	-0.778268	0.105073	2.98E-12	1.59E-10	-0.317767	0.111477	0.004776	0.014898	-0.66191	0.104757	1.71E-09	1.56E-07
P22599	A1AT2	20701	-0.415777	0.188102	0.034123	0.0719	-0.59458	0.179803	0.002069	0.008181	-0.238706	0.227597	0.301255	0.429821
P22907	HEM3	15288	0.319391	0.340696	0.36561	0.347145	-0.254934	0.515844	0.632997	0.382048	-0.363865	0.467295	0.451261	0.519846
P23242	CXA1	14609	0.697233	0.3559	0.064194	0.113053	0.511104	0.392299	0.204991	0.209213	0.585393	0.331756	0.092184	0.237757
P23492	PNPH		-0.912322	0.269041	0.001217	0.005032	-0.474248	0.190071	0.015314	0.036301	-0.456905	0.221636	0.043523	0.168039
P23927	CRYAB	12955	-0.241673	0.067295	0.000422	0.002125	-0.53917	0.069267	4.74E-13	3.00E-11	0.104697	0.081585	0.201108	0.351001
P23953	ESTN	13884	-0.168898	0.119027	0.161649	0.212459	-0.483537	0.127803	0.000389	0.002227	0.024204	0.12325	0.84508	0.652259
P24270	CATA	12359	0.138013	0.113114	0.224529	0.261029	-0.083241	0.112037	0.458816	0.324126	0.000819	0.135509	0.995186	0.686749
P24452	CAPG		0.27709	0.334298	0.41104	0.367063	0.376957	0.329182	0.257299	0.233062	0.469271	0.337548	0.170741	0.327621
P24472	GSTA4	14860	-0.386608	0.116292	0.004621	0.015222	0.391821	0.106672	0.001141	0.004907	-0.035579	0.095768	0.713976	0.611411
P24527	LKHA4	16993	-0.653959	0.382554	0.093567	0.14642	-0.455258	0.415602	0.278915	0.243627	0.11704	0.317635	0.713989	0.611411
P25444	RS2	16898	-0.025888	0.213334	0.904568	0.557647	-0.071184	0.222014	0.751661	0.414619	-0.067345	0.231178	0.774143	0.629514
P26039	TLN1	21894	-0.873953	0.302689	0.005369	0.017053	0.054451	0.292863	0.853129	0.445092	-0.470042	0.241724	0.056951	0.191962
P26041	MOES	17698	-0.43034	0.153316	0.006014	0.018878	-0.425794	0.214098	0.049457	0.081741	-0.164269	0.173417	0.346021	0.468948
P26443	DHE3	14661	-0.12539	0.070794	0.078833	0.128645	0.283232	0.05817	3.11E-06	3.62E-05	-0.143874	0.057764	0.014081	0.078686
P27005	S10A8	20201	-1.396863	0.953233	0.155278	0.207147	-1.202355	0.983823	0.233059	0.222001	-0.312678	1.10312	0.779263	0.629514
P27773	PDIA3	14827	-0.485881	0.143994	0.000919	0.004101	-0.601326	0.155452	0.00016	0.001142	-0.519077	0.165806	0.002087	0.021047
P28271	ACOC	11428	-0.092323	0.128301	0.475005	0.400427	-0.321116	0.156087	0.044594	0.07597	-0.391018	0.14678	0.010483	0.06612
P28474	ADHX	11532	-0.096369	0.103112	0.352266	0.338084	-0.035658	0.115669	0.758524	0.416688	-0.035011	0.102701	0.73394	0.616276
P28650	PURA1	11565	-0.202711	0.108716	0.065497	0.114968	-0.269024	0.183775	0.146673	0.171679	-0.08901	0.113803	0.436305	0.515672
P28656	NP1L1	53605	-0.208808	0.150795	0.177911	0.222854	-0.463715	0.206141	0.033171	0.064442	-0.099466	0.15903	0.537576	0.564102
P29391	FRIL1		-0.935809	0.229379	0.000126	0.000859	-0.526766	0.152336	0.000918	0.004191	0.129954	0.170322	0.448111	0.518689

P29699	FETUA	11625	-0.310745	0.213918	0.150552	0.20236	-0.443303	0.213842	0.041604	0.074911	0.071791	0.247046	0.772112	0.629514
P29758	OAT	18242	-0.429774	0.054	1.95E-12	1.16E-10	-0.304717	0.070205	3.26E-05	0.000289	-0.038998	0.077931	0.617869	0.591898
P29788	VTNC	22370	0.17458	0.174273	0.325691	0.326067	0.692635	0.182897	0.000813	0.003789	-0.167189	0.398242	0.678353	0.606092
P30285	CDK4	12567	0.249998	0.263264	0.351054	0.338084	0.164962	0.260956	0.532812	0.350673	-0.442597	0.267205	0.11066	0.262067
P31001	DESM	13346	0.403478	0.200282	0.045483	0.087304	0.028004	0.18991	0.882943	0.453173	-0.150844	0.198626	0.448718	0.518689
P31786	ACBP	13167	-0.171825	0.057624	0.003796	0.012902	0.110341	0.079975	0.171525	0.189464	0.048715	0.066417	0.465593	0.528122
P32261	ANT3	11905	-0.306261	0.126562	0.022819	0.05226	-0.264343	0.145834	0.081453	0.116968	-0.068125	0.16586	0.684906	0.606591
P34884	MIF	17319	-0.355192	0.149153	0.021538	0.05063	0.212125	0.149172	0.162072	0.183601	0.38762	0.236627	0.108698	0.262067
P34914	HYES	13850	-0.080956	0.090123	0.369682	0.349148	-0.254358	0.100869	0.01215	0.030929	0.081564	0.107701	0.449442	0.518689
P35441	TSP1		-0.085201	0.457615	0.85296	0.538926	0.515416	0.592338	0.388006	0.29479	-0.439901	0.524236	0.406039	0.505969
P35486	ODPA	18597	-0.21074	0.080391	0.009111	0.02586	-0.101204	0.088953	0.255956	0.232321	-0.168917	0.089594	0.060212	0.197721
P35505	FAAA	14085	-0.757617	0.586475	0.207787	0.246945	-0.232238	0.511364	0.653487	0.38715	-0.552156	0.613064	0.376724	0.487331
P35700	PRDX1	18477	-0.188161	0.071115	0.00889	0.025643	-0.316965	0.072617	2.19E-05	0.000206	-0.026355	0.07423	0.723011	0.612491
P35969	VGFR1	14254	-0.163066	0.121909	0.189902	0.231888	-0.397035	0.129922	0.00468	0.014702	0.000735	0.141454	0.99589	0.686749
P35979	RL12	269261	-0.729849	1.123049	0.52499	0.423817	0.481806	1.10029	0.66732	0.392017	-0.332246	1.111644	0.769134	0.629091
P35980	RL18	19899	0.582904	0.88726	0.547078	0.430575	0.044796	0.593421	0.94168	0.471459	-0.19794	0.649532	0.769423	0.629091
P37804	TAGL	21345	-0.044504	0.259345	0.864084	0.54055	-0.281977	0.307342	0.361172	0.285673	0.273825	0.290877	0.349032	0.468948
P38060	HMGCL	15356	-0.084688	0.134513	0.534458	0.427823	0.069534	0.129378	0.595709	0.371637	-0.322232	0.152353	0.045986	0.17518
P38647	GRP75		0.015034	0.060208	0.802934	0.52776	-0.021813	0.064028	0.733498	0.41165	0.004862	0.06471	0.940145	0.677655
P40124	CAP1	12331	-0.382642	0.32758	0.246624	0.274745	-0.216144	0.28878	0.456485	0.322994	-0.387313	0.339513	0.257587	0.399258
P40142	TKT	21881	-0.630751	0.141925	1.40E-05	0.000147	-0.160142	0.144412	0.268636	0.237978	-0.039826	0.132812	0.764585	0.629091
P41216	ACSL1	14081	0.273623	0.047481	1.41E-08	3.41E-07	0.269125	0.05058	1.53E-07	2.82E-06	0.366293	0.050939	2.39E-12	3.81E-10
P41251	NRAM1	18173	0.218402	0.456031	0.636518	0.464984	-0.240026	0.513976	0.644895	0.386224	-0.040906	0.480382	0.932909	0.675981
P42125	D3D2		0.142038	0.159549	0.374536	0.350015	-0.048473	0.173766	0.780606	0.422689	-0.060472	0.178498	0.735213	0.616276
P43023	CX6A2	12862	0.498351	0.276032	0.076914	0.126675	0.485734	0.28055	0.089316	0.122481	0.435727	0.290313	0.139935	0.296164
P43024	CX6A1	12861	0.020471	0.18342	0.912024	0.559392	0.398644	0.161921	0.020765	0.045532	0.201211	0.150625	0.194133	0.34665

P43274	H14	50709	0.430906	0.329975	0.203032	0.241834	-0.370654	0.297775	0.22433	0.215976	0.330166	0.287885	0.262741	0.40202
P45376	ALDR	11677	-0.064959	0.034885	0.063497	0.112196	-0.386397	0.038166	4.18E-21	6.17E-19	0.054207	0.038422	0.159332	0.317194
P45377	ALD2	14187	0.551484	0.29968	0.069441	0.118386	0.590312	0.30569	0.057015	0.089872	0.486197	0.345799	0.163906	0.319316
P45591	COF2	12632	-0.300081	0.085391	0.000809	0.003746	-0.336488	0.078736	5.88E-05	0.000474	0.119543	0.106116	0.263786	0.40202
P45952	ACADM	11364	0.040919	0.111341	0.713613	0.499195	0.330539	0.126196	0.009458	0.025545	0.397558	0.127443	0.002091	0.021047
P46412	GPX3		-0.59594	0.10875	1.20E-06	1.83E-05	-0.231848	0.123131	0.065308	0.09975	-0.621962	0.376468	0.104909	0.257723
P47708	RP3A	19894	0.986006	0.597438	0.110893	0.165292	-0.217077	0.904465	0.812283	0.43029	0.37549	0.645009	0.565897	0.568618
P47738	ALDH2	11669	-0.310946	0.063362	1.66E-06	2.46E-05	0.040344	0.05735	0.482416	0.332026	-0.250234	0.065819	0.000183	0.003152
P47754	CAZA2	12343	-0.291346	0.158737	0.077912	0.127923	-0.439126	0.172715	0.017304	0.039509	-0.31413	0.189392	0.110205	0.262067
P47857	K6PF	18642	-0.079776	0.056673	0.160065	0.211943	-0.368156	0.071087	3.62E-07	6.00E-06	0.064486	0.065449	0.32516	0.452755
P47934	CACP	12908	0.339438	0.059717	4.48E-08	9.19E-07	0.030841	0.082922	0.710329	0.408082	0.27917	0.059781	5.76E-06	0.000193
P47955	RLA1	56040	-0.21196	0.182367	0.250873	0.278316	-0.500253	0.335786	0.14282	0.170657	-0.1358	0.213229	0.528128	0.558012
P48036	ANXA5	11747	-0.398489	0.049839	6.73E-14	5.13E-12	-0.170301	0.06111	0.005758	0.017006	-0.028667	0.056755	0.614001	0.589967
P48758	CBR1	12408	-0.185834	0.071276	0.014918	0.03809	-0.309991	0.074882	0.000324	0.001973	-0.021405	0.099885	0.832127	0.648427
P48771	CX7A2	12866	0.160228	0.135971	0.243899	0.273422	0.475985	0.145822	0.001926	0.007755	0.201294	0.143175	0.166055	0.321534
P48772	COX8B	12869	-0.20423	1.650463	0.904998	0.557647	1.035077	1.317535	0.452278	0.320529	1.206527	1.524832	0.449146	0.518689
P48774	GSTM5	14866	-0.476304	0.318803	0.185783	0.227901	-0.060345	0.219155	0.790025	0.424621	-0.042872	0.20993	0.842276	0.652259
P48787	TNNI3	21954	0.244105	0.071208	0.000808	0.003746	-0.043138	0.094122	0.647497	0.386224	0.038081	0.09049	0.674602	0.605945
P48962	ADT1	11739	0.37945	0.103405	0.000311	0.001745	0.421677	0.090139	5.30E-06	5.72E-05	0.235019	0.104253	0.025331	0.117216
P49722	PSA2		-0.287326	0.066401	4.79E-05	0.000393	-0.196313	0.06478	0.003377	0.01108	0.034002	0.073325	0.644269	0.598925
P49817	CAV1	12389	0.159964	0.911254	0.861459	0.540169	0.218673	0.840945	0.79608	0.425862	0.167731	0.856582	0.845723	0.652259
P50136	ODBA		-0.081661	0.132812	0.541011	0.427823	-0.044597	0.155293	0.774902	0.421662	0.007163	0.169716	0.966481	0.681039
P50171	DHB8	14979	-0.807884	0.951367	0.420463	0.372701	-0.940166	1.02654	0.390201	0.29595	-0.658721	0.994822	0.529064	0.558012
P50247	SAHH	269378	-0.241417	0.201935	0.234111	0.267507	-0.264001	0.225826	0.244476	0.228322	-0.142392	0.262153	0.588007	0.579859
P50396	GDIA	14567	-0.107877	0.157453	0.498889	0.410826	-0.028839	0.292873	0.922316	0.466889	0.240612	0.139451	0.097866	0.248388
P50446	K2C6A	16687	-0.087678	0.512366	0.865451	0.540614	-0.540713	0.309853	0.093256	0.126708	-0.362332	0.364269	0.331774	0.45707

P50462	CSRP3	13009	0.218881	0.146025	0.137639	0.192269	0.090993	0.134511	0.50062	0.340097	-0.059474	0.13873	0.669351	0.605694
P50543	S10AB	20195	-0.900869	0.355422	0.014571	0.037383	-0.594336	0.29355	0.048371	0.080696	0.017462	0.334323	0.958587	0.679307
P50544	ACADV	11370	0.162017	0.04079	7.80E-05	0.000595	0.277312	0.045913	2.40E-09	6.65E-08	0.337711	0.043196	1.93E-14	4.11E-12
P50752	TNNT2	21956	0.215868	0.0595	0.000347	0.001891	-0.108437	0.074515	0.146897	0.171679	0.031657	0.074625	0.671821	0.605945
P51174	ACADL	11363	-0.062447	0.077104	0.418412	0.3715	0.10958	0.064624	0.090623	0.123891	0.188987	0.066178	0.004503	0.036315
P51667	MLRV	17906	0.685855	0.141662	3.02E-06	4.23E-05	0.320154	0.13093	0.015561	0.036662	0.022355	0.184501	0.903728	0.667886
P51881	ADT2	11740	-0.13413	0.195429	0.493697	0.408441	0.237338	0.18882	0.210992	0.212401	-0.126933	0.211788	0.55005	0.565425
P51885	LUM	17022	-0.317923	0.282045	0.262274	0.286167	0.058924	0.278352	0.832738	0.437546	0.040692	0.253035	0.87254	0.6598
P52480	KPYM	18746	-0.247381	0.043436	1.88E-08	4.36E-07	-0.169361	0.047648	0.000407	0.002277	-0.022496	0.049156	0.647374	0.600303
P52503	NDUS6	407785	0.279601	0.066744	4.30E-05	0.000364	0.387567	0.074781	5.61E-07	8.57E-06	0.308401	0.064566	3.77E-06	0.000133
P52825	CPT2	12896	0.200221	0.081948	0.01525	0.038567	-0.038987	0.088805	0.661017	0.389348	0.306504	0.083563	0.000299	0.004435
P52850	NDST2	17423	-0.397211	0.207588	0.06676	0.11604	-0.247401	0.192117	0.209172	0.211202	-0.193611	0.225812	0.399706	0.501244
P53395	ODB2	13171	0.177781	0.096943	0.068926	0.117886	0.052486	0.101985	0.607662	0.375393	-0.069084	0.118478	0.560901	0.56781
P53810	PIPNA	18738	-0.413997	0.336521	0.231047	0.266287	-0.349506	0.184394	0.071251	0.105198	-0.153924	0.18647	0.418847	0.509209
P53986	MOT1	20501	0.356491	0.183573	0.071163	0.120198	0.570511	0.232766	0.02799	0.055853	0.396296	0.184403	0.049602	0.180565
P54071	IDHP	269951	0.034479	0.04535	0.447479	0.385609	0.032518	0.050298	0.518284	0.34496	0.096541	0.060563	0.111691	0.263527
P54116	STOM	13830	-0.257872	0.26788	0.345742	0.335443	-0.034509	0.218917	0.876119	0.45124	-0.002256	0.270095	0.993413	0.686749
P54726	RD23A	19358	0.170161	0.463286	0.718214	0.499195	-0.547918	0.404054	0.202266	0.208352	-0.226763	0.513028	0.668913	0.605694
P54822	PUR8	11564	-0.077887	0.091128	0.399279	0.36235	-0.11841	0.118145	0.323748	0.268394	0.025429	0.102888	0.806471	0.639879
P55264	ADK	11534	-0.116605	0.07045	0.104157	0.158799	0.11038	0.077644	0.161104	0.182972	-0.207226	0.084242	0.01755	0.089511
P56375	ACYP2	75572	-0.047224	0.118133	0.692604	0.488869	-0.185612	0.12339	0.144562	0.170755	0.064769	0.113934	0.574993	0.573236
P56376	ACYP1	66204	-0.16294	0.07206	0.032345	0.069595	-0.205033	0.070118	0.007071	0.020076	-0.204084	0.107317	0.069811	0.216339
P56379	68MP	70257	0.436221	0.204379	0.042408	0.082549	0.318913	0.192314	0.109277	0.141117	0.356834	0.190285	0.072975	0.218206
P56380	AP4A	66401	-0.07806	0.18909	0.682059	0.483987	-0.059549	0.181028	0.743954	0.413457	-0.016888	0.186274	0.928214	0.67579
P56382	ATP5E	67126	0.392801	0.149817	0.011389	0.030851	0.43687	0.168018	0.012047	0.030845	0.405631	0.159611	0.014257	0.078976
P56391	CX6B1	110323	0.127951	0.120484	0.293065	0.30307	0.345525	0.148689	0.024002	0.050625	0.168705	0.134669	0.216247	0.362525

P56392	CX7A1	12865	0.268397	0.12269	0.032301	0.069595	0.325427	0.141322	0.024406	0.050881	0.1978	0.135229	0.148441	0.303577
P56399	UBP5	22225	-0.158891	0.049767	0.002439	0.008913	-0.262935	0.089474	0.00494	0.015196	-0.155959	0.082664	0.065137	0.206833
P56480	ATPB	11947	0.186923	0.050639	0.000237	0.001451	0.206204	0.056028	0.000247	0.001611	0.196634	0.063955	0.002177	0.021335
P56501	UCP3	22229	0.864074	0.347106	0.037559	0.076311	-0.966776	0.719744	0.236943	0.224254	-0.429462	0.857674	0.637821	0.598255
P56565	S10A1		0.074855	0.124665	0.549592	0.431281	-0.100927	0.151562	0.507098	0.341876	-0.312783	0.172097	0.072626	0.218206
P56716	RP1	19888	-0.082472	0.386561	0.832936	0.536798	-0.389043	0.370507	0.305639	0.260345	-0.560185	0.299308	0.075962	0.220966
P58252	EF2	13629	-0.132537	0.112071	0.238309	0.269991	-0.255223	0.121288	0.036597	0.069572	-0.247881	0.133717	0.06526	0.206833
P58771	TPM1	22003	0.271954	0.074426	0.000307	0.001745	-0.028298	0.101062	0.779677	0.422689	0.01371	0.10559	0.89679	0.667886
P58774	TPM2	22004	-0.494864	1.030319	0.633566	0.464984	-0.034375	1.124103	0.975769	0.480762	0.782649	0.914866	0.397792	0.499827
P59017	B2L13	94044	0.348052	0.203311	0.091991	0.144377	0.097434	0.212274	0.647866	0.386224	0.591436	0.204176	0.005283	0.040549
P59511	ATS20	223838	0.630197	0.308351	0.053721	0.099537	-0.30586	0.343322	0.382216	0.292306	0.263731	0.26804	0.336908	0.462557
P59997	KDM2A		-0.481535	0.16757	0.00798	0.02379	-0.444344	0.198046	0.033594	0.064977	-0.220553	0.209205	0.302269	0.429821
P60335	PCBP1	23983	-0.246728	0.412317	0.554755	0.434693	-0.249723	0.440365	0.575522	0.364442	0.273537	0.583693	0.643744	0.598925
P60766	CDC42	12540	0.263022	0.841787	0.758494	0.51118	-0.952824	0.744194	0.215835	0.214531	-0.199692	0.967656	0.839476	0.652178
P61014	PPLA	18821	0.739521	0.256282	0.007756	0.023251	0.452564	0.30498	0.149854	0.17376	0.405143	0.22137	0.079675	0.224586
P61027	RAB10	19325	0.019356	0.546903	0.974982	0.575938	-0.275579	0.287419	0.381691	0.292306	-0.47829	0.321439	0.210979	0.361301
P61089	UBE2N	93765	-0.400469	0.146642	0.012875	0.03418	-0.527897	0.16514	0.006005	0.017384	0.094562	0.216336	0.669206	0.605694
P61148	FGF1	14164	0.090629	0.146023	0.540234	0.427823	0.004483	0.141238	0.974939	0.480762	0.067809	0.14257	0.638646	0.598255
P61971	NTF2	68051	-0.1795	0.061253	0.005018	0.016131	-0.126997	0.075117	0.096773	0.128877	0.149469	0.068928	0.035007	0.145759
P61979	HNRPK	15387	-0.070478	0.172507	0.68665	0.485308	-0.433902	0.204123	0.044016	0.075276	-0.280217	0.177478	0.129308	0.282107
P61982	1433G	22628	-0.079003	0.152748	0.606472	0.45904	-0.459227	0.197089	0.022174	0.047447	0.048793	0.169958	0.774817	0.629514
P62075	TIM13	30055	0.048126	0.136938	0.726897	0.499208	-0.094263	0.179922	0.602856	0.373988	0.139327	0.151854	0.364244	0.481413
P62082	RS7	20115	-0.439904	0.392643	0.268374	0.288146	-0.400556	0.432768	0.359608	0.285365	-0.56181	0.46274	0.230778	0.371747
P62204	CALM	12313	-0.33479	0.099548	0.001437	0.005767	-0.161144	0.119441	0.183026	0.195611	-0.18044	0.129351	0.169317	0.325902
P62245	RS15A	267019	0.12575	0.254053	0.625312	0.464731	-0.055526	0.252166	0.82766	0.436431	-0.012384	0.238669	0.959087	0.679307
P62259	1433E	22627	-0.09579	0.114482	0.403866	0.362811	-0.226947	0.124226	0.069382	0.103474	0.074239	0.116426	0.524585	0.556049

P62270	RS18	20084	-0.306553	0.267692	0.262985	0.286167	-0.244261	0.232087	0.302276	0.258974	-0.175974	0.210197	0.410747	0.505969
P62631	EF1A2	13628	-0.10126	0.180987	0.576564	0.444642	-0.322744	0.187368	0.08685	0.120864	0.064984	0.176736	0.713616	0.611411
P62806	H4	69386	0.22639	0.169676	0.184982	0.227901	-0.674187	0.150934	2.02E-05	0.000199	-0.743545	0.178824	6.88E-05	0.001462
P62814	VATB2	11966	-0.989698	0.865717	0.277221	0.294095	0.044491	0.536912	0.935133	0.469877	-0.950695	0.729295	0.218999	0.365217
P62821	RAB1A	19324	-0.021405	0.335474	0.94976	0.571644	-0.003856	0.372842	0.991863	0.483494	0.151533	0.383251	0.69696	0.608215
P62827	RAN	19384	-0.260371	0.185131	0.162641	0.212715	-0.185013	0.189026	0.329965	0.270155	-0.125749	0.211115	0.552787	0.566127
P62855	RS26	27370	0.002016	0.223054	0.99286	0.578391	-0.10468	0.286865	0.718662	0.409085	-0.332508	0.270989	0.232229	0.371747
P62869	ELOB	67673	-0.202553	0.348931	0.586758	0.449216	-0.405393	0.650255	0.566785	0.363196	-0.083334	0.265453	0.762722	0.629091
P62897	CYC	13063	0.334801	0.203659	0.102994	0.157477	0.116511	0.163411	0.477308	0.331896	0.310279	0.186525	0.099228	0.248869
P62932	FBX40	207215	-0.003514	0.295412	0.990742	0.578391	-0.293098	0.631639	0.653648	0.38715	-0.17331	0.465399	0.717373	0.611451
P62962	PROF1	18643	-0.349733	0.077272	1.59E-05	0.000152	-0.245705	0.072947	0.001059	0.004689	-0.26361	0.085601	0.002704	0.023925
P63017	HSP7C	15481	-0.171902	0.069529	0.014448	0.037246	-0.298295	0.081771	0.000361	0.00213	-0.108349	0.082504	0.191185	0.34405
P63028	TCTP	22070	-0.169826	0.143491	0.246551	0.274745	0.043454	0.126894	0.734189	0.41165	-0.376432	0.162904	0.02938	0.129078
P63030	BR44L	55951	0.239807	0.158041	0.141719	0.196425	0.288722	0.165418	0.093195	0.126708	0.303912	0.167056	0.081922	0.226906
P63038	CH60	15510	0.017357	0.076477	0.820553	0.531384	-0.308431	0.066167	4.14E-06	4.70E-05	-0.195255	0.088991	0.028761	0.128103
P63101	1433Z	22631	-0.328403	0.124079	0.009108	0.02586	-0.386041	0.092547	5.45E-05	0.000447	-0.169403	0.132983	0.205091	0.355035
P63158	HMGB1	15289	-0.001924	0.158737	0.990421	0.578391	-0.215361	0.172558	0.223136	0.215797	-0.074074	0.187657	0.696528	0.608215
P63325	RS10	67097	-0.084372	0.188178	0.657604	0.471852	-0.276193	0.220253	0.221448	0.214634	-0.158302	0.189363	0.411416	0.505969
P63330	PP2AA	19052	0.100295	0.282235	0.72645	0.499208	-0.13984	0.211133	0.515319	0.34496	-0.601174	0.16674	0.002023	0.021047
P67778	PHB	18673	0.230497	0.068969	0.001008	0.004339	0.30982	0.079845	0.000147	0.001104	0.130367	0.076375	0.089751	0.236264
P68037	UB2L3	22195	-0.168931	0.076127	0.035417	0.073252	-0.258071	0.140285	0.077269	0.112214	-0.073698	0.092265	0.432263	0.513754
P68040	GBLP	14694	0.39651	0.558842	0.484316	0.403811	0.601191	0.580751	0.31011	0.262263	-0.206552	0.620225	0.742006	0.619519
P68254	1433T	22630	-0.518929	0.159991	0.002065	0.007872	-0.915889	0.237155	0.000313	0.001953	-0.332474	0.214088	0.126998	0.279943
P68368	TBA4A	22145	-0.085798	0.108813	0.437537	0.383302	-0.313735	0.117964	0.013218	0.032709	-0.101636	0.133965	0.455433	0.523705
P68510	1433F	22629	-0.488697	0.173993	0.007391	0.022666	-0.494146	0.197557	0.016927	0.039051	-0.207047	0.214872	0.341511	0.467869
P70168	IMB1	16211	-0.282529	0.157052	0.081465	0.130937	-0.322421	0.168133	0.063586	0.097794	-0.118834	0.204036	0.565501	0.568618

P70188	KIFA3	16579	1.042555	0.842528	0.226993	0.263032	0.768847	0.861294	0.380225	0.292306	1.027302	0.928988	0.279762	0.417382
P70195	PSB7	19177	-0.218598	0.439	0.623696	0.464731	-0.484606	0.404246	0.243962	0.228322	0.06891	0.547761	0.901209	0.667886
P70296	PEBP1	23980	-0.264357	0.039038	1.21E-10	4.60E-09	-0.322207	0.053203	6.19E-09	1.44E-07	-0.011058	0.042824	0.79651	0.634269
P70349	HINT1	15254	-0.179698	0.054577	0.001353	0.00547	-0.027929	0.05779	0.629889	0.381671	0.149051	0.06077	0.015946	0.083266
P70392	RGRF2		-0.101538	0.209069	0.632482	0.46488	0.255813	0.256833	0.330575	0.270155	-0.404228	0.500786	0.428607	0.513754
P70398	USP9X		-1.835934	1.220131	0.144931	0.199407	-1.76249	1.123934	0.129419	0.160125	-0.651247	0.733987	0.384116	0.493157
P70404	IDHG1	15929	-0.10555	0.086054	0.221169	0.258814	0.104842	0.070614	0.138891	0.168167	0.19805	0.078527	0.01235	0.072179
P70670	NACAM	17938	0.099352	0.302995	0.750488	0.50886	-0.037801	0.57471	0.948996	0.473896	0.214074	0.341877	0.546752	0.565425
P70695	F16P2	14120	-0.011794	0.143144	0.935156	0.566214	-0.160987	0.170527	0.354955	0.283285	0.33137	0.13871	0.025905	0.118723
P80313	TCPH	12468	0.01086	0.377442	0.977283	0.575938	-0.263713	0.357901	0.468367	0.327737	0.084051	0.470198	0.859765	0.657432
P80314	TCPB	12461	0.561527	0.46965	0.259423	0.284256	-0.472047	0.223183	0.056024	0.088626	-0.376168	0.356611	0.312274	0.442074
P80316	TCPE	12465	-0.082031	0.154083	0.598984	0.45531	-0.541155	0.135674	0.000482	0.00257	-0.023191	0.147639	0.876496	0.6598
P80317	TCPZ		-0.077548	0.144477	0.594084	0.452876	-0.419936	0.180196	0.025048	0.051255	-0.333136	0.202621	0.107441	0.262067
P84244	H33	15078	-0.400008	1.33928	0.771303	0.516411	-1.265773	1.551311	0.433541	0.31432	1.595486	1.581914	0.342705	0.468496
P85094	ISC2A	664994	-0.029108	0.17499	0.868125	0.540614	0.195791	0.147551	0.186674	0.197339	0.108123	0.148273	0.467132	0.528122
P97352	S10AD	20196	0.515641	0.337606	0.161023	0.212159	0.205043	0.38933	0.608887	0.375626	-0.279236	0.372506	0.474951	0.529163
P97372	PSME2	19188	-0.197603	0.183935	0.295476	0.304384	-0.636283	0.335432	0.074004	0.108182	-0.545847	0.246002	0.038871	0.155525
P97429	ANXA4	11746	-0.530765	0.206044	0.012314	0.033021	-0.119115	0.211455	0.575132	0.364442	0.016706	0.23012	0.942373	0.677987
P97443	SMYD1	12180	-0.647715	0.319157	0.051678	0.09642	-0.741965	0.411496	0.082977	0.118219	-0.176435	0.317141	0.582931	0.576638
P97450	ATP5J	11957	0.259931	0.121286	0.036712	0.075346	0.410463	0.142678	0.005776	0.017006	0.253914	0.129377	0.055385	0.191194
P97467	AMD	18484	-1.636999	0.733994	0.033632	0.071499	-1.262358	0.765681	0.110008	0.141236	-1.595544	0.85783	0.075726	0.220966
P97807	FUMH	14194	-0.016232	0.081634	0.842482	0.538781	-0.137131	0.075324	0.069382	0.103474	0.019499	0.087017	0.822809	0.645528
P97823	LYPA1	18777	0.00263	0.168186	0.987564	0.578391	-0.23319	0.172566	0.180452	0.195076	-0.161027	0.159801	0.316987	0.446761
P99024	TBB5	22154	-0.299918	0.22233	0.184247	0.227901	-0.382878	0.213648	0.080684	0.11641	-0.518573	0.375588	0.175656	0.328086
P99027	RLA2	67186	-0.125082	0.201705	0.540575	0.427823	-0.322914	0.234682	0.18057	0.195076	-0.244797	0.213213	0.26223	0.40202
P99028	QCR6	66576	0.124437	0.10084	0.219375	0.25728	0.266781	0.081556	0.001362	0.005746	0.289369	0.077114	0.000268	0.00422

P99029	PRDX5	54683	-0.019873	0.041956	0.636125	0.464984	0.02035	0.059412	0.732221	0.41165	0.012035	0.050787	0.812874	0.642481
Q00519	XDH	22436	-0.238836	0.241603	0.327376	0.32653	0.025509	0.241531	0.916297	0.465438	-0.073171	0.198543	0.714059	0.611411
Q00623	APOA1		0.439911	0.118764	0.000322	0.001772	-0.155382	0.135217	0.252751	0.230831	-0.058699	0.16213	0.718024	0.611451
Q00897	A1AT4	20703	-0.067269	0.249763	0.788907	0.523599	-0.427691	0.283139	0.137463	0.167273	-0.044812	0.354192	0.899955	0.667886
Q00915	RET1	19659	-0.452387	0.922601	0.634474	0.464984	-0.467765	0.605242	0.451615	0.320529	0.350669	0.562312	0.542244	0.565361
Q01768	NDKB	18103	0.099803	0.154216	0.518694	0.420352	-0.021251	0.199759	0.915447	0.465438	0.067452	0.152179	0.658402	0.603589
Q01853	TERA	269523	-0.231129	0.069496	0.000989	0.004296	-0.381307	0.069442	8.59E-08	1.73E-06	-0.139777	0.079092	0.078272	0.222907
Q02053	UBA1	22201	-0.196118	0.117406	0.096853	0.150677	-0.26587	0.132138	0.045913	0.077621	-0.210978	0.148454	0.157344	0.315434
Q02566	MYH6	17888	0.745142	0.111034	4.96E-11	2.20E-09	-0.166184	0.112958	0.141854	0.170278	-0.271143	0.123762	0.028957	0.128103
Q03265	ATPA	11946	0.213801	0.050097	2.23E-05	0.000202	0.227092	0.053411	2.39E-05	0.000216	0.215921	0.050732	2.36E-05	0.000685
Q04447	KCRB	12709	-0.461092	0.191903	0.017731	0.043204	-0.049363	0.174582	0.777803	0.422689	-0.352259	0.20381	0.086677	0.232005
Q04857	CO6A1	12833	-0.501937	0.299381	0.105609	0.160098	-0.2113	0.270476	0.441733	0.317115	-0.191691	0.319265	0.553858	0.566127
Q05816	FABP5	16592	-0.470051	0.152227	0.002585	0.009385	-0.378877	0.156048	0.016853	0.039051	-0.560584	0.211417	0.009359	0.063691
Q05920	PYC		-0.521891	0.233233	0.026813	0.059616	-0.070444	0.235521	0.765285	0.41797	-0.431426	0.268412	0.110268	0.262067
Q06185	ATP5I	11958	0.321549	0.120587	0.010139	0.028404	0.53549	0.144129	0.00049	0.002582	0.353544	0.128967	0.008517	0.059622
Q06770	CBG	12401	-0.53195	0.132252	0.000104	0.00074	-0.535619	0.150416	0.000542	0.00276	0.179274	0.212474	0.400678	0.501475
Q06890	CLUS	12759	-1.382827	0.519521	0.01339	0.035165	-0.662506	0.27124	0.021995	0.047399	-0.192551	0.492775	0.69958	0.608833
Q06986	SIAH2	20439	-0.236032	0.458166	0.611149	0.46062	0.339924	0.299261	0.26678	0.237978	0.182793	0.423735	0.670387	0.605771
Q07417	ACADS	11409	-0.002144	0.048513	0.964768	0.573303	-0.086201	0.049989	0.085435	0.119754	0.003823	0.051056	0.940348	0.677655
Q08857	CD36	12491	-0.024809	0.102608	0.809602	0.528785	-0.004302	0.117384	0.970863	0.479946	0.172377	0.108218	0.115631	0.268746
Q08AU7	MADL2	68515	-0.206481	0.413643	0.621853	0.464099	-0.372719	0.432684	0.397198	0.29956	-0.211596	0.486957	0.668139	0.605694
Q09LZ8	CBPC6	78933	0.328355	0.151581	0.040032	0.079707	0.420231	0.127022	0.002751	0.009646	0.181572	0.137005	0.197554	0.348596
Q0KK59	UNC79	217843	0.18712	0.51959	0.725009	0.499208	0.261685	0.447773	0.568241	0.363196	-0.412025	0.491017	0.415497	0.509023
Q11011	PSA	19155	-0.3002	0.192887	0.122349	0.176452	-0.355139	0.168943	0.037634	0.070633	0.029895	0.16655	0.857861	0.657432
Q19LI2	A1BG	117586	-1.199389	0.201365	1.04E-08	2.65E-07	-0.553263	0.161236	0.000708	0.003374	-0.1232	0.149169	0.409743	0.505969
Q1XH17	TRI72	434246	-0.197827	0.098397	0.045192	0.087058	-0.207579	0.111511	0.063559	0.097794	0.108181	0.092641	0.243776	0.38063

Q2TPA8	HSDL2	72479	0.038306	0.10952	0.726849	0.499208	0.129926	0.089097	0.146183	0.171679	0.052095	0.098891	0.598895	0.584511
Q30D77	COOA1	71355	0.202968	0.317651	0.528435	0.42478	0.295447	0.325367	0.372197	0.289228	0.236707	0.384728	0.544175	0.565425
Q3LAC4	PREX2	109294	-0.201624	0.106925	0.070563	0.119915	-0.077734	0.106152	0.47054	0.328738	-0.175772	0.137965	0.214851	0.362091
Q3TC72	FAHD2	68126	-0.021591	0.143538	0.881122	0.546722	0.178465	0.152055	0.246696	0.228371	-0.083111	0.202087	0.68297	0.606591
Q3TEI4	CO039	74211	0.208336	0.145303	0.165689	0.21512	0.431094	0.162023	0.013683	0.0333	0.233529	0.156801	0.152	0.309365
Q3TP92	CNEP1	67181	-0.264283	0.696052	0.715434	0.499195	-1.355531	1.085879	0.243409	0.228322	-1.458384	0.676764	0.083721	0.227924
Q3TTY5	K22E	16681	-0.033352	0.410568	0.935879	0.566214	-0.524587	0.421138	0.224004	0.215976	-0.558375	0.443985	0.220621	0.366005
Q3TZ89	SC31B	240667	0.162399	0.200454	0.425204	0.376279	0.187813	0.20554	0.369243	0.287943	0.297767	0.233271	0.213999	0.362091
Q3U0B3	DHR11	192970	-0.552306	0.324559	0.101215	0.156551	-0.221565	0.361471	0.545443	0.357113	-0.975008	0.529016	0.078246	0.222907
Q3U132	K0748	67596	0.224191	0.227995	0.335261	0.329468	0.329755	0.23134	0.165935	0.185603	0.419953	0.213652	0.061019	0.199343
Q3UGR5	HDHD2	76987	-0.073867	0.066221	0.267917	0.288146	-0.475529	0.065002	1.65E-10	5.62E-09	-0.18406	0.0982	0.064727	0.206833
Q3UIU2	NDUB6	230075	0.231439	0.099054	0.021968	0.05119	0.431226	0.117811	0.00045	0.002432	0.381818	0.10091	0.000311	0.004507
Q3ULD5	MCCB	78038	0.500168	0.343232	0.147664	0.201524	-0.024489	0.299917	0.935061	0.469877	-0.41441	0.304689	0.17648	0.328086
Q3ULJ0	GPD1L	333433	0.062497	0.330722	0.850654	0.538926	-0.279598	0.418591	0.506362	0.341876	-0.056892	0.429547	0.895034	0.667658
Q3UM45	PP1R7	66385	0.076457	0.388459	0.845953	0.538926	-0.225467	0.238141	0.354036	0.283285	0.006034	0.336357	0.985884	0.686749
Q3UWA4	TRI40	195359	0.077715	0.148821	0.605948	0.45904	-0.087242	0.150748	0.567756	0.363196	0.153235	0.160596	0.349512	0.468948
Q3V3A1	CDK15	271697	0.267765	0.552461	0.642704	0.467244	-0.531078	0.54372	0.357301	0.284133	-0.139518	0.668395	0.841561	0.652259
Q4ACU6	SHAN3	58234	0.148318	0.242363	0.548214	0.430834	-0.01417	0.290002	0.961598	0.477498	-0.238344	0.399229	0.562587	0.56781
Q4KML4	CF115		0.163343	0.135743	0.234632	0.267529	0.204373	0.142972	0.15909	0.181149	-0.013961	0.148335	0.925414	0.675186
Q505D7	OPA3	403187	-0.192488	0.454614	0.675478	0.479955	-0.488667	0.331678	0.152671	0.176103	-0.34179	0.408584	0.411113	0.505969
Q52KG5	KI26A	668303	-0.024294	0.208632	0.90831	0.558398	-0.031523	0.245761	0.899103	0.459339	0.155201	0.145098	0.29638	0.427987
Q571E4	GALNS	50917	0.253157	0.241282	0.303735	0.310382	0.355809	0.32492	0.283528	0.246245	0.182005	0.302809	0.553438	0.566127
Q5DU05	CE164		-0.336484	0.163361	0.050913	0.095457	-0.368363	0.193427	0.070016	0.104069	-0.194979	0.211388	0.367326	0.481758
Q5EBG6	HSPB6	243912	-0.301417	0.130339	0.022591	0.05196	-0.381865	0.213388	0.076587	0.111589	0.536159	0.132021	9.33E-05	0.0018
Q5SWD9	TSR1	104662	1.354205	2.517742	0.619201	0.462767	NA	NA	NA	NA	1.496029	2.901812	0.628146	0.597253
Q60590	A1AG1	18405	-2.374329	0.811272	0.010415	0.028797	-0.381684	0.481061	0.436406	0.31432	0.335309	0.567095	0.563141	0.56781

Q60597	ODO1	18293	-0.000965	0.041589	0.981496	0.577445	0.035886	0.045904	0.43456	0.31432	-0.157434	0.045077	0.000505	0.006839
Q60605	MYL6	17904	-0.048624	0.271968	0.858458	0.538926	-0.164606	0.271656	0.54593	0.357113	-0.514144	0.21406	0.018344	0.092748
Q60692	PSB6	19175	0.217653	0.193538	0.271441	0.290256	0.236146	0.187328	0.218648	0.214634	-0.278668	0.660059	0.676647	0.605945
Q60759	GCDH	270076	0.773219	1.064821	0.47616	0.400625	-0.9781	0.903916	0.290437	0.250283	-0.735663	1.025716	0.481141	0.530665
Q60854	SPB6	20719	-0.478405	0.124753	0.000184	0.001183	-0.029847	0.081993	0.716337	0.408888	0.113267	0.091637	0.218439	0.365217
Q60864	STIP1	20867	-0.12109	0.133096	0.373255	0.350015	-0.463919	0.181522	0.020467	0.045104	-0.239305	0.177305	0.19854	0.348596
Q60930	VDAC2	22334	0.131465	0.175064	0.45355	0.388478	0.181167	0.16168	0.263818	0.236661	0.362732	0.180759	0.046198	0.17518
Q60931	VDAC3	22335	0.163894	0.060785	0.007619	0.023099	0.030164	0.085426	0.724392	0.410582	0.150118	0.139178	0.282153	0.418011
Q60932	VDAC1	22333	0.371705	0.094333	9.86E-05	0.000711	0.195618	0.112515	0.083005	0.118219	0.437633	0.106925	5.38E-05	0.001224
Q60936	ADCK3	67426	0.162255	0.644969	0.803089	0.52776	0.5018	0.511911	0.334552	0.270663	0.355066	0.513853	0.495067	0.539114
Q60988	STIL		-0.204738	0.344308	0.558438	0.436938	-0.16815	0.169817	0.334529	0.270663	-0.508733	0.212689	0.027886	0.12689
Q60994	ADIPO		-0.422033	0.134693	0.002815	0.010015	-0.483205	0.150873	0.002304	0.008769	-0.15262	0.136834	0.270135	0.408762
Q61037	TSC2		0.133845	0.168434	0.434015	0.380917	0.10215	0.173433	0.560951	0.362724	0.076486	0.209412	0.718131	0.611451
Q61085	RHPN1	14787	-0.112147	0.142107	0.438432	0.383302	0.019917	0.116588	0.865733	0.448498	-0.29662	0.215711	0.182355	0.333818
Q61147	CERU	12870	-1.491845	0.178464	7.44E-15	7.94E-13	-0.276958	0.136772	0.04393	0.075276	0.014028	0.158514	0.929559	0.675943
Q61171	PRDX2	21672	-0.07833	0.052135	0.134623	0.189856	-0.157608	0.044889	0.000556	0.002797	-0.080704	0.045785	0.079664	0.224586
Q61207	SAP	19156	-0.695649	0.142103	3.57E-06	4.83E-05	-0.297857	0.153712	0.05539	0.088253	-0.234486	0.169319	0.169333	0.325902
Q61233	PLSL	18826	-1.003933	0.320049	0.002316	0.008581	-0.378301	0.286674	0.190346	0.200264	-0.324419	0.301519	0.284959	0.420214
Q61292	LAMB2	16779	-0.071843	0.283186	0.816125	0.531384	0.17269	0.312245	0.618766	0.378182	-0.119784	0.278674	0.69632	0.608215
Q61316	HSP74		-0.136218	0.084497	0.110057	0.16518	-0.520705	0.171039	0.003009	0.010174	-0.167601	0.076152	0.030362	0.13158
Q61425	HCDH	15107	-0.062615	0.111406	0.57448	0.44455	-0.037408	0.121137	0.757673	0.416688	0.095261	0.131471	0.46929	0.528198
Q61554	FBN1		-0.950849	0.267432	0.001165	0.004932	-1.115656	0.326617	0.001626	0.006668	-0.840262	0.287567	0.006142	0.045499
Q61598	GDIB	14569	-0.297296	0.070872	4.30E-05	0.000364	-0.206468	0.071726	0.004473	0.014255	-0.27342	0.095571	0.004755	0.037862
Q61599	GDIR2	11857	-0.75358	0.23815	0.002698	0.009661	-0.696275	0.326009	0.038055	0.070823	-0.143542	0.254947	0.57615	0.573493
Q61646	HPT	15439	-0.378992	0.434932	0.387077	0.358442	-0.529529	0.357227	0.142941	0.170657	0.472628	0.442993	0.290223	0.424051
Q61704	ITIH3	16426	-0.758611	0.685577	0.294405	0.303867	-1.348131	1.118736	0.251412	0.230082	-0.092174	0.512502	0.86054	0.657432

Q61735	CD47	16423	-0.270894	0.41153	0.516158	0.420352	0.094018	0.247009	0.706696	0.407051	0.201845	0.278537	0.475666	0.529163
Q61838	A2M	11287	-0.255677	0.053805	2.55E-06	3.68E-05	-0.671728	0.062012	4.21E-25	1.86E-22	-0.085072	0.054043	0.116012	0.268746
Q62048	PEA15	18611	-0.466424	0.334782	0.175819	0.222322	-0.646353	0.358862	0.083759	0.118531	0.275955	0.309764	0.38223	0.491917
Q62188	DPYL3	22240	-0.515802	0.365023	0.169496	0.218468	-0.283644	0.283286	0.326292	0.26866	-0.385618	0.349451	0.281736	0.418011
Q62234	MYOM1	17929	0.269222	0.156958	0.089901	0.142776	-0.082224	0.135858	0.546661	0.357113	-0.182972	0.162516	0.263763	0.40202
Q62388	ATM	11920	0.217843	0.556424	0.700589	0.492674	-0.442644	0.503558	0.391639	0.296533	0.396376	0.554123	0.483587	0.530665
Q62425	NDUA4	17992	0.092792	0.143454	0.519122	0.420352	0.324334	0.136123	0.018952	0.04223	0.248914	0.14101	0.08061	0.22523
Q62426	CYTB	13014	-0.518861	0.148107	0.001068	0.00456	-0.22571	0.132929	0.095607	0.128877	-0.305255	0.164553	0.070296	0.216339
Q62465	VAT1	26949	0.363688	0.193898	0.109813	0.16518	0.101604	0.277068	0.732409	0.41165	-0.022276	0.169182	0.90038	0.667886
Q63844	MK03	26417	2.373329	0.176429	0.000888	0.004017	NA	NA	NA	NA	0.96395	1.148948	0.439734	0.517752
Q63918	SDPR	20324	0.028357	0.304202	0.926294	0.564318	-0.318278	0.53289	0.554286	0.36052	0.021264	0.301985	0.944331	0.677987
Q64105	SPRE		-0.206141	0.05248	0.000134	0.000908	-0.07476	0.053649	0.165672	0.185603	0.208604	0.055691	0.000272	0.00422
Q64433	CH10	15528	-0.025338	0.076757	0.741699	0.504825	-0.198106	0.097165	0.042934	0.075144	-0.137596	0.103419	0.185166	0.337028
Q64669	NQO1	18104	-0.20993	0.10797	0.055415	0.101269	0.100188	0.108846	0.360139	0.285365	0.384981	0.172612	0.028843	0.128103
Q64727	VINC	22330	-0.229282	0.095824	0.017358	0.042488	-0.12373	0.100972	0.221416	0.214634	-0.255268	0.098729	0.010251	0.066099
Q64737	PUR2	14450	-0.23122	0.121832	0.063495	0.112196	-0.273869	0.130497	0.041132	0.074911	-0.062103	0.075211	0.413229	0.507219
Q68FD5	CLH	67300	-0.497749	0.101154	1.15E-05	0.000129	-0.399378	0.143741	0.007772	0.021788	-0.191637	0.125255	0.133702	0.288727
Q6IFX2	K1C42	68239	0.616087	0.586197	0.307975	0.313626	-0.461807	0.409141	0.279413	0.243627	-2.069703	1.084387	0.085397	0.230229
Q6IRU2	TPM4	326618	-0.68719	0.184287	0.000944	0.004164	-0.562485	0.258729	0.038993	0.071965	-0.272305	0.189245	0.163093	0.319316
Q6NSW3	SPKAP	77629	-0.16019	0.11699	0.182631	0.227168	-0.628342	0.381698	0.111764	0.143077	0.007062	0.137507	0.959465	0.679307
Q6NZQ8	MARH1	72925	-0.15051	0.255642	0.561107	0.438384	-0.06838	0.255479	0.791077	0.424621	0.00151	0.267074	0.995537	0.686749
Q6P3A8	ODBB	12040	0.000972	0.102537	0.992462	0.578391	0.362844	0.099761	0.000519	0.002703	-0.175886	0.228185	0.443658	0.51859
Q6P8J7	KCRS	76722	0.130038	0.063334	0.040742	0.080093	0.389482	0.068623	2.73E-08	6.04E-07	0.198452	0.075989	0.009398	0.063691
Q6PB66	LPPRC	72416	-0.012122	0.065357	0.853019	0.538926	-0.044083	0.077561	0.570317	0.363711	-0.175151	0.074956	0.020374	0.100613
Q6PGF3	MED16	216154	-0.100646	0.502519	0.842817	0.538781	0.337833	0.517319	0.51946	0.34496	0.148043	0.520229	0.778413	0.629514
Q6PHN9	RAB35	77407	-0.15704	0.201045	0.441787	0.384576	-0.117152	0.210471	0.582738	0.367164	0.008915	0.216398	0.967494	0.681039

Q6X7S9	EID2	386655	-0.074464	0.081473	0.369129	0.349148	-0.622983	0.248119	0.018596	0.041812	0.215228	0.133332	0.120117	0.274772
Q6ZQA6	IGSF3	78908	-0.050496	0.190923	0.795556	0.524749	0.086066	0.196912	0.669228	0.392617	0.243052	0.194238	0.232872	0.371805
Q71RI9	KAT3	229905	-0.181316	0.080527	0.025706	0.057394	0.160236	0.069042	0.02155	0.046791	-0.054705	0.073515	0.457971	0.524957
Q76MZ3	2AAA	51792	-0.030863	0.203662	0.879818	0.546548	0.156649	0.154749	0.31353	0.26402	0.017828	0.17917	0.920932	0.673601
Q78IK2	USMG5	66477	0.258941	0.157809	0.112873	0.16669	0.357682	0.206862	0.095652	0.128877	0.174982	0.188256	0.36189	0.480294
Q78IK4	APOOL	68117	0.497024	0.312152	0.119614	0.173918	0.241067	0.34232	0.485592	0.332952	0.170663	0.522458	0.745876	0.621122
Q78ZA7	NP1L4	17955	0.091417	0.179563	0.612873	0.461268	-0.069013	0.195936	0.726124	0.410763	0.078109	0.210269	0.71203	0.611411
Q791V5	MTCH2	56428	0.385145	0.275585	0.168418	0.217604	0.418411	0.257444	0.109725	0.141236	0.49704	0.300729	0.105185	0.257723
Q7TMF3	NDUAC	66414	0.212935	0.100295	0.038435	0.077104	0.273312	0.108393	0.014728	0.035262	0.261488	0.107726	0.018931	0.094962
Q7TNG8	LDHD	52815	-0.124184	0.650093	0.850645	0.538926	0.447374	0.784482	0.575948	0.364442	0.370704	0.820721	0.656613	0.603589
Q7TNS2	CA151	433771	1.134815	0.323436	0.072508	0.12091	-0.328532	0.443392	0.594037	0.371116	0.568622	0.416196	0.243632	0.38063
Q7TQ48	SRCA	106393	0.102969	0.048161	0.033503	0.071499	0.068533	0.059078	0.247127	0.228371	-0.227368	0.058234	0.000124	0.002262
Q7TQI3	OTUB1	107260	-0.392406	0.137388	0.008507	0.025069	-0.105125	0.127268	0.416612	0.306533	-0.30252	0.123291	0.022136	0.107253
Q80SU7	GVIN1	1E+08	-0.044265	0.24557	0.85835	0.538926	-0.201859	0.305234	0.514222	0.34496	-0.051278	0.26375	0.847485	0.65282
Q80WQ9	ZBED4	223773	-0.081991	0.24802	0.743609	0.50548	0.301787	0.243194	0.225713	0.2164	0.198173	0.285339	0.494027	0.538902
Q80XN0	BDH	71911	-0.593482	0.141319	3.94E-05	0.000345	0.111308	0.122836	0.365845	0.286301	0.067069	0.134673	0.619018	0.592109
Q80YV2	NIPA	232679	0.16981	0.34602	0.627882	0.46488	-0.17641	0.39028	0.655322	0.387539	-0.716884	0.424479	0.104761	0.257723
Q8BFP9	PDK1	228026	0.031322	0.551405	0.955172	0.572049	-0.425861	0.494873	0.397667	0.29956	0.006075	0.566265	0.991542	0.686749
Q8BFR5	EFTU	233870	0.060915	0.051078	0.234097	0.267507	-0.042048	0.051048	0.410848	0.303806	-0.020797	0.055363	0.70751	0.610402
Q8BFS6	CPPED	223978	0.065246	0.149503	0.666786	0.47441	-0.001791	0.214993	0.993422	0.483494	0.026654	0.154738	0.864814	0.657432
Q8BFZ3	ACTBL	238880	-0.645555	0.263892	0.021509	0.05063	-0.702689	0.3304	0.043091	0.075144	-0.293728	0.323289	0.372615	0.485426
Q8BG32	PSD11	69077	-0.469474	0.217415	0.042542	0.082549	-0.184428	0.105582	0.096018	0.128877	0.178632	0.09089	0.064161	0.206833
Q8BGC4	ZADH2	225791	-0.720724	0.458895	0.127132	0.181326	-0.25727	0.47759	0.593833	0.371116	0.403383	0.538792	0.45988	0.525028
Q8BGH2	SAM50	68653	0.314114	0.295842	0.291992	0.30307	0.25768	0.310825	0.409873	0.303806	-0.025933	0.279379	0.926324	0.675186
Q8BGK2	ARHL1	234072	-0.053995	0.120921	0.656159	0.471852	-0.454063	0.182337	0.01456	0.035242	0.150565	0.122608	0.222296	0.366964
Q8BGQ7	SYAC	234734	-0.44851	0.329785	0.192684	0.234685	-0.597221	0.501554	0.267885	0.237978	-0.885953	0.549226	0.13502	0.290589

Q8BGY7	CR019	108654	0.254786	0.257073	0.331522	0.32775	-0.238097	0.218103	0.285386	0.246891	0.134852	0.206286	0.51978	0.554648
Q8BH59	CMC1	78830	0.268549	0.085158	0.001754	0.006831	0.324658	0.081057	7.57E-05	0.000599	0.104548	0.099124	0.292339	0.425626
Q8BH64	EHD2	259300	0.28449	0.362263	0.443744	0.385023	-0.178965	0.87659	0.843326	0.441432	0.196003	0.494004	0.697978	0.60827
Q8BH86	CN159	217830	-0.249197	0.23201	0.28805	0.301981	-0.036252	0.120618	0.765081	0.41797	-0.272337	0.226656	0.235825	0.373712
Q8BH95	ECHM	93747	0.07122	0.113225	0.530041	0.425321	0.324791	0.096776	0.00094	0.00425	0.205455	0.123913	0.098964	0.248869
Q8BIJ6	SYIM	381314	0.009682	0.168472	0.95454	0.572049	-0.072335	0.192348	0.709518	0.408082	-0.141252	0.146673	0.344074	0.468948
Q8BK30	NDUV3	78330	0.296566	0.148498	0.05638	0.10215	0.336279	0.164587	0.051295	0.083531	0.300877	0.162845	0.077018	0.222633
Q8BKZ9	ODPX	27402	-0.059926	0.075602	0.430325	0.378948	0.083808	0.076787	0.278358	0.243627	-0.130758	0.071777	0.07254	0.218206
Q8BM89	ARSJ	271970	0.931312	1.352349	0.508397	0.417368	2.016243	0.819137	0.02996	0.058785	0.968708	0.982745	0.345458	0.468948
Q8BMF3	MAON	109264	0.023246	0.113611	0.838085	0.537519	0.082759	0.12148	0.496538	0.337843	0.289146	0.124706	0.021509	0.1054
Q8BMF4	ODP2	235339	-0.035972	0.047015	0.444672	0.385202	0.033585	0.05311	0.527524	0.348227	0.058144	0.054207	0.284157	0.420004
Q8BMS1	ECHA	97212	0.202355	0.040289	6.20E-07	9.74E-06	0.069942	0.048397	0.148776	0.17336	0.302563	0.048495	7.13E-10	7.57E-08
Q8BMS4	COQ3	230027	0.169364	0.090133	0.066448	0.115875	0.107601	0.077037	0.169053	0.187748	0.075865	0.08319	0.366878	0.481758
Q8BP40	PPA6	66659	0.071195	0.269043	0.793387	0.523966	-0.493591	0.389601	0.216418	0.214531	-0.529368	0.277627	0.068593	0.2142
Q8BS95	GPHR	67549	0.444813	0.943028	0.641086	0.466704	-1.898787	1.404474	0.188034	0.198303	-0.33296	1.220673	0.787576	0.630306
Q8BTM8	FLNA	192176	-0.60347	0.808425	0.476741	0.400625	-1.453144	1.434246	0.334862	0.270663	-0.470828	0.954266	0.636837	0.598255
Q8BVI4	DHPR	110391	-0.013497	0.155951	0.931171	0.565288	-0.004181	0.110629	0.969916	0.479946	0.061084	0.113269	0.590751	0.579942
Q8BVI5	STX16	228960	0.271855	0.194091	0.173608	0.220047	0.22091	0.218003	0.320607	0.266934	0.175421	0.214147	0.42148	0.510724
Q8BW75	AOFB	109731	-0.349065	0.334993	0.30417	0.310382	0.160616	0.348398	0.64735	0.386224	0.546179	0.270081	0.050626	0.182211
Q8BWF0	SSDH	214579	-0.121633	0.117262	0.301309	0.309365	0.152771	0.122373	0.213896	0.213866	-0.006643	0.113916	0.953581	0.677987
Q8BWT1	THIM	52538	0.185536	0.046463	7.37E-05	0.00057	0.273963	0.045903	4.21E-09	1.04E-07	0.144997	0.043847	0.001007	0.011884
Q8BZ25	ANKK1	244859	-0.030635	0.492517	0.951283	0.571644	0.251961	0.671497	0.714629	0.408888	0.36727	0.538072	0.506018	0.545444
Q8BZF8	PGM5	226041	1.035416	0.389591	0.013776	0.035686	-0.524806	0.581532	0.3751	0.289958	0.021813	0.594557	0.97108	0.682056
Q8C0M9	ASGL1	66514	0.023501	0.146401	0.873709	0.543386	0.144793	0.129094	0.272279	0.240244	0.120062	0.125042	0.346543	0.468948
Q8C156	CND2	215387	-0.958323	0.538103	0.08708	0.138709	-0.452172	0.546694	0.416005	0.306533	-0.841585	0.632323	0.196253	0.347284
Q8C196	CPSM	227231	-0.128268	0.454696	0.780288	0.518804	0.300764	0.522814	0.570688	0.363711	-0.74887	0.496481	0.146361	0.300772

Q8C4V4	FBXL3	50789	-0.179225	0.370098	0.65352	0.471021	-0.666451	0.425538	0.192376	0.201444	0.049676	0.326447	0.885001	0.662501
Q8C5Q4	GRSF1	231413	0.068034	0.235174	0.778267	0.518804	0.170146	0.181811	0.365218	0.286301	-0.028323	0.176452	0.875385	0.6598
Q8CAQ8	IMMT	76614	0.459968	0.101566	7.53E-06	8.93E-05	0.344202	0.096829	0.000416	0.002277	0.44511	0.112151	8.44E-05	0.001683
Q8CC88	K0564	219189	0.020203	0.111724	0.856746	0.538926	0.018061	0.1156	0.876061	0.45124	0.268582	0.12886	0.039061	0.155525
Q8CDI6	CD158	320696	0.254082	0.211573	0.24061	0.27202	0.035971	0.256634	0.889609	0.455539	-0.23005	0.350311	0.51762	0.553798
Q8CG76	ARK72	110198	0.020396	0.082115	0.804487	0.528029	-0.174921	0.052663	0.001357	0.005746	0.127143	0.064819	0.053582	0.188216
Q8CGK3	LONM	74142	-0.054625	0.073813	0.461003	0.392881	-0.380002	0.099531	0.00023	0.001602	-0.147028	0.119854	0.222927	0.366964
Q8CGW4	SOX30	214105	0.246452	0.286446	0.397445	0.36188	-0.197676	0.403363	0.628358	0.381671	-0.247374	0.616042	0.691566	0.608215
Q8CHT0	AL4A1	212647	-0.077728	0.082455	0.348013	0.337033	-0.076407	0.085511	0.373593	0.289298	-0.026136	0.082881	0.75317	0.624746
Q8CI04	COG3		2.10262	1.370115	0.163421	0.213213	-0.014233	1.950415	0.994414	0.483494	0.919141	1.739322	0.613522	0.589967
Q8CI51	PDLI5	56376	-0.025946	0.246704	0.917048	0.561149	-0.087099	0.242802	0.723072	0.410582	-0.001276	0.185785	0.99458	0.686749
Q8CI94	PYGB	110078	-0.352957	0.045586	4.45E-14	3.96E-12	-0.014715	0.041791	0.724877	0.410582	0.581662	0.043399	1.56E-35	9.95E-33
Q8JZL0	ZN467	68910	0.996075	0.652957	0.139212	0.193453	1.101558	0.660898	0.107566	0.139721	0.626291	0.719236	0.392505	0.498097
Q8JZN5	ACAD9	229211	-1.730391	1.259618	0.202766	0.241834	-0.154533	0.746266	0.84412	0.441432	-1.405363	0.775698	0.112927	0.264484
Q8JZQ2	AFG32	69597	0.010429	0.224255	0.963421	0.573303	-0.090887	0.187891	0.633587	0.382048	-0.293833	0.213648	0.18591	0.337335
Q8K0D5	EFGM	28030	0.073939	0.232104	0.752817	0.509145	-0.272379	0.215338	0.219147	0.214634	-0.547498	0.259624	0.047768	0.177956
Q8K0Z7	TACO1	70207	0.167657	0.165726	0.32223	0.323209	0.420547	0.154824	0.011804	0.030455	0.298822	0.242367	0.231898	0.371747
Q8K183	PDXK	216134	-0.307226	0.124447	0.020451	0.048937	-0.420509	0.126237	0.002598	0.009386	-0.207193	0.130988	0.126794	0.279943
Q8K1M6	DNM1L	74006	-0.905804	0.493675	0.076145	0.125797	-0.476466	0.536989	0.382757	0.292306	0.328091	0.588806	0.581971	0.576584
Q8K1Z0	COQ9	67914	0.115888	0.074021	0.119494	0.173918	-0.19229	0.082376	0.020897	0.045597	-0.060735	0.078515	0.440503	0.517752
Q8K2B3	DHSA	66945	0.145255	0.054321	0.007711	0.023246	0.305407	0.055453	5.51E-08	1.16E-06	0.289073	0.059132	1.35E-06	5.73E-05
Q8K2C6	SIRT5	68346	-0.218304	0.25649	0.403477	0.362811	-0.230498	0.256495	0.377088	0.290942	-0.472148	0.27139	0.094716	0.242323
Q8K370	ACD10	71985	0.026678	0.138384	0.847469	0.538926	-0.363178	0.143739	0.012795	0.03202	0.345042	0.149723	0.02307	0.109677
Q8K3J1	NDUS8	225887	-0.120628	0.218038	0.584825	0.449216	0.222604	0.241772	0.365661	0.286301	0.190586	0.218084	0.390827	0.496958
Q8K411	PREP	69617	-0.626862	0.42194	0.149393	0.201856	0.141712	0.064196	0.036309	0.069321	-0.238359	0.284841	0.410952	0.505969
Q8K480	MFRP	259172	-0.011289	0.264108	0.96651	0.573412	-0.537816	0.24005	0.041801	0.074923	-0.371507	0.282945	0.213736	0.362091

Q8K4Z3	AIBP	246703	-0.296635	0.060253	4.42E-06	5.75E-05	-0.214541	0.088772	0.017825	0.040282	0.11463	0.052648	0.03252	0.137373
Q8QZR5	ALAT1	76282	0.051626	0.413508	0.901728	0.556918	-0.519246	0.530049	0.339584	0.273979	-0.329126	0.442208	0.464956	0.528122
Q8QZS1	HIBCH	227095	-0.025882	0.057692	0.654077	0.471021	-0.132372	0.070407	0.061193	0.094771	0.065444	0.062612	0.296948	0.427987
Q8QZT1	THIL	110446	0.086882	0.042292	0.040826	0.080093	0.027718	0.048227	0.56591	0.363196	-0.060809	0.044866	0.176433	0.328086
Q8R0F8	FAHD1		-0.153541	0.184242	0.414016	0.368209	0.242995	0.158504	0.137342	0.167273	0.038134	0.163355	0.817484	0.643187
Q8R0N6	HOT	76187	0.210728	0.178333	0.244686	0.273728	0.25377	0.109015	0.024582	0.050881	-0.079821	0.143476	0.581243	0.576584
Q8R0P4	CK067	66273	-0.166282	0.152669	0.286072	0.301091	-0.061362	0.133968	0.650734	0.38715	-0.011307	0.134228	0.933568	0.675981
Q8R164	BPHL	68021	-0.100112	0.107003	0.353724	0.338874	-0.106229	0.106773	0.324393	0.268394	0.152862	0.102015	0.140711	0.296819
Q8R1A4	DOCK7		-0.259233	0.129182	0.056673	0.102168	-0.081792	0.15272	0.597184	0.372033	-0.112248	0.110615	0.321251	0.449734
Q8R1G2	CMBL	69574	0.436379	0.372416	0.254419	0.280479	-0.70143	0.509468	0.183079	0.195611	-0.301991	0.675325	0.660392	0.603589
Q8R1I1	QCR9	66152	0.474024	0.171187	0.010231	0.028434	0.552438	0.220997	0.019068	0.04223	0.368473	0.163528	0.033653	0.141044
Q8R1S0	COQ6	217707	-0.426127	0.423987	0.328966	0.326893	-0.146558	0.437949	0.742526	0.413182	-0.924389	0.438048	0.053312	0.188216
Q8R2G4	NAR3	109979	0.533302	0.288422	0.101627	0.156734	0.585879	0.244355	0.043335	0.075273	0.225518	0.261925	0.437788	0.516467
Q8R2Q4	RRF2M	320806	0.013388	0.29965	0.964787	0.573303	0.276055	0.323652	0.402885	0.300621	-0.041072	0.396029	0.91834	0.673601
Q8R404	QIL1	224904	0.327773	0.42855	0.451253	0.387132	-0.083356	0.554105	0.881629	0.453173	0.351191	0.4756	0.468066	0.528122
Q8R4N0	CLYBL	69634	-0.087661	0.073364	0.233187	0.267507	-0.038482	0.095895	0.688518	0.397615	-0.075804	0.109009	0.487456	0.533562
Q8VCA8	SCRN2	217140	-2.394247	2.026692	0.447193	0.385609	-3.860537	NaN	NaN	NA	0.188018	NaN	NaN	NA
Q8VCM7	FIBG	99571	-0.728329	0.199347	0.000518	0.002537	-0.370353	0.299888	0.221428	0.214634	-0.169198	0.354282	0.634527	0.598255
Q8VCT4	CES3	104158	-0.116762	0.135165	0.389699	0.358442	-0.708032	0.158914	2.10E-05	0.000202	0.232746	0.159004	0.146272	0.300772
Q8VCW8	ACSF2	264895	-0.146374	0.206063	0.481834	0.40237	0.682026	0.243733	0.008025	0.02225	0.474593	0.207738	0.028169	0.127269
Q8VDC0	SYLM	102436	-0.396627	0.451096	0.395227	0.360734	-0.653439	0.727053	0.38513	0.293229	0.824643	0.506939	0.138242	0.295786
Q8VDK1	NIT1	27045	-0.272249	0.760868	0.723371	0.499208	-0.098749	0.840229	0.907346	0.462483	-0.267446	0.903601	0.769792	0.629091
Q8VDN2	AT1A1	11928	0.1743	0.224126	0.438888	0.383302	0.627693	0.182151	0.00087	0.004015	-0.217054	0.27626	0.434342	0.514307
Q8VDQ1	PTGR2	77219	-0.080701	0.176004	0.65039	0.470368	-0.059005	0.158991	0.713555	0.408875	0.126867	0.192781	0.516744	0.553798
Q8VE95	CH082	223665	-0.091671	0.08497	0.290951	0.30307	0.346048	0.081554	0.000247	0.001611	0.339627	0.1497	0.032562	0.137373
Q8VED5	K2C79	223917	1.147676	0.833785	0.193819	0.234685	NA	NA	NA	NA	-2.158019	0.857123	0.039942	0.158043

Q8VEK3	HNRPU	51810	-0.416212	0.167939	0.020305	0.048806	-0.578917	0.1696	0.002192	0.008593	-0.37802	0.182821	0.050097	0.18133
Q8VEM8	MPCP	18674	0.392926	0.075573	4.42E-07	7.14E-06	0.333149	0.09028	0.000281	0.001805	0.36201	0.081105	1.30E-05	0.000395
Q8VHN7	GPR98	110789	0.126769	0.245729	0.610652	0.46062	-0.611926	0.322356	0.071487	0.105198	0.089299	0.249533	0.724194	0.612678
Q91V12	BACH	70025	0.187206	0.348353	0.595741	0.453491	0.744443	0.347539	0.042119	0.074923	0.492525	0.394268	0.224715	0.368006
Q91V61	SFXN3	94280	-1.216675	1.447901	0.448033	0.385609	-2.868572	1.124081	0.037997	0.070823	0.02821	1.211297	0.98207	0.685835
Q91V64	ISOC1	66307	0.324983	0.237547	0.185749	0.227901	-0.513824	0.317942	0.12174	0.152757	0.379951	0.378948	0.32932	0.455082
Q91V92	ACLY	104112	2.041277	0.564038	0.001828	0.007067	0.233994	0.624774	0.711768	0.408379	-0.386248	0.652259	0.560719	0.56781
Q91VD9	NDUS1	227197	0.210394	0.052407	6.85E-05	0.000546	0.275165	0.05565	1.04E-06	1.49E-05	0.328238	0.051165	3.44E-10	4.38E-08
Q91VI7	RINI	107702	0.560662	0.35675	0.123925	0.177765	-0.144015	0.203267	0.482743	0.332026	0.122908	0.246618	0.621526	0.593616
Q91VM9	IPYR2	74776	-0.155335	0.088424	0.081254	0.130937	-0.060403	0.087527	0.491323	0.334808	0.029554	0.081196	0.716494	0.611451
Q91VR2	ATPG	11949	0.270075	0.061892	2.02E-05	0.000189	0.322741	0.066189	2.13E-06	2.86E-05	0.256517	0.061264	4.28E-05	0.00105
Q91WD5	NDUS2	226646	0.135442	0.074861	0.071277	0.120198	0.273342	0.078264	0.00054	0.00276	0.311856	0.084444	0.00026	0.00422
Q91WK1	SPRY4	66701	-0.028286	0.114525	0.808062	0.528785	0.216596	0.148043	0.156984	0.179904	-0.237156	0.082599	0.011084	0.067897
Q91WK5	GCSH		-0.303627	0.418306	0.479105	0.401347	0.234292	0.407701	0.573044	0.364442	0.345517	0.32918	0.305819	0.4339
Q91WS0	CISD1	52637	0.008647	0.101586	0.932387	0.565383	0.010439	0.1103	0.924857	0.467642	0.459153	0.101839	2.56E-05	0.000708
Q91X72	HEMO	15458	-1.341048	0.124552	5.46E-22	1.46E-19	-0.685755	0.143299	2.95E-06	3.53E-05	0.071662	0.152005	0.637764	0.598255
Q91XE4	ACY3	71670	0.353477	1.195816	0.770587	0.516411	1.161365	0.969691	0.24748	0.228371	-0.68052	1.391912	0.631539	0.598255
Q91Y97	ALDOB	230163	-0.361847	0.740927	0.631913	0.46488	-0.29589	0.577707	0.615115	0.377888	-0.389908	0.709866	0.589963	0.579942
Q91YT0	NDUV1	17995	0.168862	0.045783	0.000255	0.001531	0.33105	0.046292	3.84E-12	1.55E-10	0.217833	0.044131	1.18E-06	5.63E-05
Q91YT2	RN185	193670	-1.5422	0.749213	0.050563	0.095341	-0.942652	0.911469	0.311789	0.263054	0.041788	0.977776	0.966296	0.681039
Q91YY4	ATPF2	246782	-0.248122	0.42559	0.565327	0.440121	-0.419716	0.54531	0.45006	0.320352	-0.259426	0.621139	0.680243	0.606591
Q91Z53	GRHPR	76238	-0.218959	0.046417	6.67E-06	8.09E-05	0.023288	0.041893	0.579359	0.366077	0.081793	0.046844	0.083698	0.227924
Q91Z61	DIRA1	208666	-0.775007	0.665563	0.36436	0.346575	0.257089	0.815256	0.805527	0.428844	-0.95763	1.568195	0.58458	0.577372
Q91ZA3	PCCA	110821	-0.023838	0.089701	0.790621	0.523966	0.083238	0.091726	0.364914	0.286301	0.112096	0.091994	0.224072	0.367898
Q91ZD1	OSR2	107587	0.394781	0.856822	0.648809	0.470368	-0.000785	0.836478	0.999258	0.483726	-0.878817	1.235214	0.483652	0.530665
Q91ZJ0	MUS81	71711	0.195034	0.221753	0.388222	0.358442	0.090698	0.511042	0.860623	0.446896	0.47044	0.213047	0.037476	0.151101

Q91ZJ5	UGPA	216558	-0.239742	0.066445	0.000431	0.002131	-0.047376	0.087702	0.589921	0.37011	0.304526	0.086076	0.000552	0.007331
Q920B9	SP16H		0.118516	0.413241	0.776632	0.518678	0.001759	0.418578	0.99668	0.483535	0.115539	0.535783	0.831086	0.648427
Q921G7	ETFD	66841	0.272498	0.061498	1.16E-05	0.000129	0.400038	0.057608	1.25E-11	4.61E-10	0.342031	0.061252	4.10E-08	2.45E-06
Q921I1	TRFE	22041	-0.916372	0.072235	6.21E-33	3.31E-30	-0.56274	0.07354	7.19E-14	5.31E-12	-0.256123	0.063611	6.36E-05	0.001397
Q922B1	MACD1	107227	-0.057196	0.118345	0.629547	0.46488	-0.037153	0.102071	0.716346	0.408888	0.123995	0.112976	0.274156	0.412885
Q922R8	PDIA6		-1.006186	0.790623	0.223873	0.260834	-1.61209	0.718543	0.041556	0.074911	0.166289	0.680058	0.81014	0.641313
Q922U2	K2C5	110308	0.168506	0.355714	0.637853	0.464984	0.080378	0.249612	0.748842	0.413579	-0.135823	0.346468	0.696935	0.608215
Q923D2	BLVRB	233016	-0.269668	0.167063	0.109306	0.16518	-0.192858	0.125733	0.127907	0.159142	-0.196677	0.140245	0.163837	0.319316
Q924D0	RT4I1	170728	-0.01382	0.333405	0.96712	0.573412	0.134316	0.371613	0.719462	0.409085	0.591049	0.362451	0.110431	0.262067
Q924M7	MPI	110119	-0.35271	0.112775	0.002153	0.008047	-0.057494	0.127909	0.65379	0.38715	-0.171342	0.111742	0.127772	0.280679
Q924X2	CPT1B	12895	0.203319	0.0738	0.006195	0.019331	0.021848	0.074225	0.76867	0.419302	0.261591	0.092963	0.005204	0.040428
Q93092	TALDO	21351	-0.1709	0.084739	0.049104	0.092917	-0.327448	0.086435	0.000415	0.002277	-0.063705	0.099298	0.524211	0.556049
Q99J27	ACATN	11416	-0.262023	0.137939	0.068639	0.117771	0.0872	0.164479	0.600499	0.373573	-0.092751	0.17681	0.604689	0.587928
Q99J39	DCMC	56690	-0.125701	0.237083	0.600651	0.455928	-0.106242	0.292084	0.719111	0.409085	-0.021334	0.309901	0.945711	0.677987
Q99J99	THTM		-0.169931	0.268422	0.53563	0.427823	-0.809894	0.499245	0.121229	0.152548	-0.293121	0.362937	0.431143	0.513754
Q99JY0	ECHB	231086	0.176379	0.04695	0.000188	0.001192	0.161758	0.045391	0.000392	0.002227	0.282149	0.047396	4.45E-09	3.55E-07
Q99K24	S39A3	106947	-0.000923	0.433657	0.998318	0.580304	-0.243639	0.419799	0.566661	0.363196	-0.22279	0.364974	0.547317	0.565425
Q99KI0	ACON	11429	-0.024403	0.036604	0.505083	0.415286	0.119703	0.040141	0.002915	0.009934	0.089591	0.036728	0.014855	0.080195
Q99KQ4	NAMPT	59027	0.211493	0.099106	0.037871	0.076549	-0.044058	0.107514	0.683522	0.39628	0.056669	0.098495	0.567689	0.568624
Q99KR7	PPIF	105675	0.250495	0.10148	0.016831	0.041388	-0.049592	0.098992	0.618463	0.378182	0.028011	0.113779	0.806568	0.639879
Q99L13	3HIDH	58875	-0.314035	0.075587	4.64E-05	0.000387	0.180747	0.082457	0.029355	0.058306	-0.123335	0.09903	0.214335	0.362091
Q99L47	F10A1	70356	0.179893	0.195964	0.367397	0.348222	-1.105499	0.481508	0.029994	0.058785	0.170885	0.213223	0.430743	0.513754
Q99LB2	DHRS4		0.21773	0.064216	0.000814	0.003746	0.038569	0.074536	0.605308	0.374984	0.307841	0.072581	3.25E-05	0.000829
Q99LC3	NDUAA	67273	0.2003	0.095113	0.036142	0.074463	0.203346	0.098272	0.039492	0.072283	0.139576	0.094725	0.141881	0.29732
Q99LC5	ETFA	110842	-0.046758	0.076753	0.54263	0.428338	-0.173687	0.083334	0.037582	0.070633	0.094999	0.087927	0.280443	0.417419
Q99LD8	DDAH2	51793	-0.643903	0.448445	0.168195	0.217604	-0.409931	0.517587	0.439943	0.316343	-0.016878	0.492582	0.973065	0.682698

Q99LP6	GRPE1	17713	0.032438	0.146191	0.825452	0.532618	-0.172283	0.156095	0.275342	0.242464	0.051505	0.148721	0.730875	0.616276
Q99LX0	PARK7	57320	-0.214784	0.039957	2.16E-07	3.72E-06	-0.157663	0.043721	0.000392	0.002227	0.152719	0.043902	0.000624	0.008107
Q99LY9	NDUS5	595136	0.089204	0.1192	0.457552	0.390651	0.335522	0.130391	0.012911	0.032128	0.273782	0.117448	0.023906	0.112545
Q99MN9	PCCB	66904	-0.139776	0.082259	0.090304	0.14299	0.040553	0.100525	0.686934	0.397218	-0.032429	0.096408	0.736842	0.616825
Q99MR8	MCCA	72039	0.412199	0.15857	0.010876	0.02961	0.456505	0.161664	0.005806	0.017006	0.278686	0.17293	0.110641	0.262067
Q99N87	RT05	77721	0.17144	0.117905	0.16005	0.211943	0.433647	0.131385	0.002901	0.009934	0.069209	0.130046	0.599929	0.584511
Q99N96	RM01	94061	-0.410191	0.508571	0.431061	0.378948	-0.728599	0.848141	0.402262	0.300621	-0.544471	0.720246	0.458949	0.524957
Q99NB1	ACS2L	68738	-0.134249	0.067718	0.048018	0.091512	-0.196204	0.075077	0.009267	0.025183	0.035702	0.083086	0.667633	0.605694
Q99PT1	GDIR1	192662	-0.487858	0.086118	1.45E-07	2.57E-06	-0.37886	0.125961	0.003294	0.010888	-0.154067	0.093953	0.10428	0.257723
Q9CPP6	NDUA5	68202	0.091127	0.189433	0.632001	0.46488	0.514857	0.173221	0.004002	0.012845	0.476684	0.185215	0.012315	0.072179
Q9CPQ1	COX6C	12864	0.115668	0.400343	0.774933	0.518191	-0.649409	0.497672	0.204303	0.209128	-0.542033	0.482282	0.272652	0.411593
Q9CPQ3	TOM22	223696	-0.076129	0.206698	0.716328	0.499195	0.157443	0.184659	0.402664	0.300621	0.362467	0.187964	0.067437	0.212676
Q9CPQ8	ATP5L	27425	0.229172	0.097936	0.023081	0.052634	0.376773	0.103021	0.000587	0.002889	0.361524	0.107937	0.001565	0.017185
Q9CPU0	LGUL	109801	-0.154908	0.096491	0.110794	0.165292	-0.370917	0.116622	0.00183	0.007435	-0.092131	0.103432	0.374811	0.486299
Q9CPU4	MGST3	66447	-0.415334	0.652016	0.539988	0.427823	-1.520313	0.764439	0.087046	0.120864	-0.311544	0.895656	0.734526	0.616276
Q9CPV4	GLOD4	67201	-0.411405	0.095244	3.53E-05	0.000314	-0.320117	0.083884	0.000231	0.001602	0.092844	0.121717	0.447302	0.518689
Q9CPX8	QCR10	66594	0.402477	0.12305	0.003021	0.010675	0.408582	0.168207	0.022355	0.047605	0.52432	0.162497	0.0036	0.029788
Q9CPY7	AMPL	66988	-0.537934	0.637698	0.487726	0.405387	-0.780298	0.952216	0.443851	0.31812	-0.79058	0.623088	0.332194	0.45707
Q9CQ54	NDUC2	68197	0.212306	0.12861	0.102705	0.157477	0.019143	0.132607	0.885579	0.454	-0.008811	0.135233	0.948229	0.677987
Q9CQ60	6PGL	66171	-0.245577	0.166712	0.144302	0.199407	-0.458539	0.200051	0.024232	0.050868	-0.108655	0.189261	0.567379	0.568624
Q9CQ62	DECR	67460	0.252557	0.064346	0.000121	0.000837	-0.043759	0.067714	0.518914	0.34496	0.327876	0.096502	0.000839	0.010081
Q9CQ65	MTAP	66902	-0.482266	0.113478	0.00026	0.001543	-0.284363	0.119444	0.024893	0.051255	-0.129948	0.139508	0.360884	0.47996
Q9CQ69	QCR8	22272	0.311224	0.11003	0.006587	0.020435	0.281548	0.114017	0.016792	0.039051	0.351379	0.108921	0.002239	0.021608
Q9CQ75	NDUA2	17991	0.158309	0.121538	0.19836	0.237861	0.256673	0.12283	0.041463	0.074911	0.259506	0.117454	0.03185	0.136932
Q9CQ89	CUTA	67675	-0.091143	0.205606	0.660537	0.471852	0.074153	0.175411	0.675604	0.393114	0.12997	0.192565	0.505249	0.545444
Q9CQ91	NDUA3	66091	0.07559	0.163664	0.648022	0.470368	-0.082576	0.254717	0.748389	0.413579	0.38613	0.157955	0.022223	0.107253

Q9CQ92	FIS1	66437	-0.384811	0.27823	0.179925	0.22485	-0.01341	0.182351	0.941986	0.471459	0.233092	0.319035	0.472391	0.528886
Q9CQA3	DHSB	67680	0.224637	0.084475	0.008257	0.024479	0.230155	0.087554	0.009015	0.024649	0.185434	0.102222	0.070766	0.216737
Q9CQC7	NDUB4	68194	0.29646	0.093975	0.002647	0.009545	0.349472	0.099988	0.000966	0.004323	0.332946	0.1001	0.001675	0.018084
Q9CQH3	NDUB5	66046	0.176256	0.100283	0.08313	0.132813	0.335045	0.120859	0.00704	0.020076	0.234995	0.116057	0.046757	0.176252
Q9CQI6	COTL1	72042	0.104209	0.427142	0.80917	0.528785	-0.228831	0.395211	0.567566	0.363196	0.166412	0.407324	0.686494	0.606591
Q9CQJ8	NDUB9	66218	0.142368	0.091382	0.121734	0.176042	0.33379	0.095431	0.000647	0.003151	0.266676	0.08683	0.002642	0.023706
Q9CQM9	GLRX3	30926	-0.115225	0.116033	0.329845	0.327157	-0.397784	0.137311	0.007547	0.021293	-0.122853	0.139304	0.386574	0.49451
Q9CQN1	TRAP1	68015	-0.109784	0.067268	0.105382	0.160098	-0.371149	0.093567	0.000126	0.000979	-0.065984	0.086205	0.445654	0.518689
Q9CQN6	TM14C	66154	0.234992	0.320507	0.46954	0.397074	0.111434	0.342344	0.74731	0.413579	0.108551	0.271416	0.692596	0.608215
Q9CQQ7	AT5F1	11950	0.1797	0.12412	0.149466	0.201856	0.09584	0.125528	0.44618	0.318268	0.15465	0.121527	0.204967	0.355035
Q9CQR4	ACO13	66834	0.0766	0.117496	0.51612	0.420352	-0.137758	0.140838	0.330522	0.270155	-0.040175	0.147459	0.785917	0.62977
Q9CQV8	1433B	54401	-0.398356	0.143436	0.007606	0.023099	-0.603659	0.221772	0.008762	0.024107	-0.202835	0.166854	0.229943	0.371747
Q9CQX8	RT36	66128	0.071585	0.078164	0.362543	0.345463	-0.003776	0.071888	0.958241	0.476372	0.050797	0.083318	0.543995	0.565425
Q9CQZ5	NDUA6	67130	0.295384	0.11823	0.015613	0.039114	0.31732	0.122652	0.012457	0.031529	0.29929	0.120191	0.01621	0.083956
Q9CQZ6	NDUB3	66495	0.266479	0.123215	0.035095	0.073153	0.379267	0.120018	0.002606	0.009386	0.404504	0.119507	0.00141	0.015967
Q9CR21	ACPM	70316	0.175958	0.080253	0.031361	0.068306	0.307315	0.077135	0.000153	0.00112	0.154987	0.118532	0.195244	0.346783
Q9CR61	NDUB7	66916	0.141447	0.098134	0.151459	0.203068	0.305934	0.100599	0.002766	0.009646	0.149525	0.101997	0.144777	0.299533
Q9CR62	M2OM	67863	0.031112	0.095994	0.746153	0.506564	0.213268	0.10525	0.043855	0.075276	0.310063	0.10057	0.002309	0.02195
Q9CR68	UCRI	66694	0.235567	0.125546	0.061725	0.109791	0.166	0.121744	0.17389	0.19065	0.099742	0.147532	0.499638	0.541598
Q9CRB8	MTFP1	67900	0.42161	0.111435	0.000415	0.002125	0.070478	0.106612	0.511482	0.344308	0.473506	0.111574	9.99E-05	0.001871
Q9CRB9	CHCH3	66075	0.358807	0.102411	0.000693	0.003334	0.334236	0.106454	0.002241	0.008633	0.35446	0.116277	0.002996	0.025444
Q9CW03	SMC3	13006	0.03936	0.296819	0.898236	0.556048	0.299263	0.444979	0.522817	0.34667	0.492261	0.385725	0.242595	0.38063
Q9CW46	RAVR1	71766	0.091618	0.294556	0.7587	0.51118	-0.591252	0.461989	0.212366	0.212817	-0.411332	0.447936	0.36843	0.481758
Q9CWJ9	PUR9	108147	-0.596255	0.331715	0.081144	0.130937	-0.292425	0.494023	0.558193	0.361998	-0.450672	0.33046	0.181601	0.333396
Q9CWL2	CASZ1	69743	0.439084	0.228801	0.074207	0.123358	0.263559	0.22121	0.249849	0.229601	0.086619	0.204057	0.677238	0.605945
Q9CWR0	ARHGP	52666	-0.763344	0.685767	0.275841	0.293274	-1.179675	0.539585	0.038783	0.071876	-0.027689	0.450067	0.951498	0.677987

Q9CXJ4	ABCB8	74610	0.944146	0.520551	0.081276	0.130937	0.430194	0.67412	0.529175	0.348797	0.395781	0.840448	0.642337	0.598925
Q9CXV1	DHSD	66925	0.274746	0.330685	0.409723	0.366837	0.158719	0.333369	0.635919	0.382048	0.101978	0.347305	0.770258	0.629091
Q9CXZ1	NDUS4		0.300591	0.163992	0.078283	0.128138	0.402707	0.17971	0.033795	0.065083	0.330443	0.164443	0.05586	0.19132
Q9CYR0	SSBP	381760	0.199645	0.142563	0.173214	0.220047	-0.253971	0.181475	0.173487	0.19065	-0.103468	0.166234	0.539533	0.564382
Q9CZ13	QCR1	22273	0.210068	0.06183	0.000733	0.003492	0.157776	0.055407	0.004583	0.014499	0.185843	0.060129	0.002114	0.021047
Q9CZ30	OLA1	67059	-0.037894	0.083161	0.650526	0.470368	-0.258056	0.114013	0.027813	0.055853	-0.267215	0.10462	0.013946	0.078686
Q9CZ42	CARKD	69225	0.084394	0.119812	0.485274	0.403978	0.069692	0.08525	0.418485	0.307401	0.067819	0.095636	0.482688	0.530665
Q9CZ44	NSF1C	386649	0.38612	0.196187	0.067823	0.116747	0.422265	0.19664	0.044877	0.07616	-0.633892	0.748327	0.411198	0.505969
Q9CZB0	C560	66052	0.243663	0.192569	0.211393	0.250117	0.510743	0.208051	0.017475	0.039693	0.288589	0.182151	0.119684	0.274772
Q9CZR8	EFTS	66399	-0.142007	0.139297	0.312533	0.315857	-0.029471	0.164561	0.858579	0.446896	-0.525964	0.193788	0.008994	0.062277
Q9CZS1	AL1B1	72535	-0.727728	0.242631	0.0032	0.011233	-0.001992	0.23468	0.993239	0.483494	-0.570623	0.241646	0.019584	0.097468
Q9CZU6	CISY	12974	0.026097	0.089258	0.770163	0.516411	-0.075343	0.098693	0.445707	0.318268	-0.108163	0.110151	0.326812	0.453584
Q9D020	5NT3	107569	-0.092742	0.464829	0.843082	0.538781	-0.116672	0.425173	0.785429	0.423747	0.166874	0.375403	0.659658	0.603589
Q9D023	BR44	70456	0.238844	0.101818	0.021668	0.050711	0.30903	0.149674	0.042457	0.074923	0.164924	0.136813	0.232199	0.371747
Q9D051	ODPB	68263	-0.013634	0.043538	0.75438	0.509556	0.113759	0.050336	0.0245	0.050881	0.014093	0.050787	0.781601	0.629514
Q9D0F9	PGM1	72157	-0.070792	0.08321	0.395471	0.360734	-0.196229	0.099785	0.05001	0.082347	0.147673	0.095231	0.121947	0.276515
Q9D0K2	SCOT1	67041	-0.237152	0.072569	0.001204	0.00502	-0.214718	0.077068	0.005653	0.016919	-0.20388	0.083705	0.015452	0.082033
Q9D0M3	CY1	66445	0.350122	0.057153	3.64E-09	1.08E-07	0.300756	0.062358	2.52E-06	3.28E-05	0.328013	0.057165	3.09E-08	2.19E-06
Q9D0S9	HINT2	68917	-0.060468	0.069246	0.386476	0.358442	0.059258	0.057842	0.31026	0.262263	0.036086	0.055925	0.521765	0.554907
Q9D172	ES1	28295	-0.159513	0.066012	0.016541	0.040865	-0.096595	0.072275	0.182852	0.195611	0.078312	0.089892	0.384742	0.493157
Q9D1A2	CNDP2	66054	0.239863	0.189984	0.213116	0.251598	-0.248639	0.279127	0.377686	0.290942	-0.030862	0.253221	0.903565	0.667886
Q9D1G1	RAB1B	76308	-0.507748	0.334933	0.146891	0.200984	-0.284913	0.196511	0.165296	0.185603	-0.153004	0.228381	0.513073	0.551184
Q9D1G3	HHATL	74770	0.036909	0.220205	0.868238	0.540614	-0.402657	0.350908	0.262497	0.236321	0.009094	0.219677	0.967273	0.681039
Q9D1H9	MFAP4	76293	-2.566609	0.30481	0.000387	0.002047	-2.591612	0.309972	7.98E-06	8.23E-05	-2.077713	0.617991	0.015191	0.081324
Q9D1I5	MCEE	73724	-0.199558	0.174295	0.265115	0.286957	-0.305063	0.205816	0.151856	0.175621	-0.095302	0.155821	0.547359	0.565425
Q9D1L0	CHCH2	14004	-0.121113	0.133355	0.372114	0.350015	0.23612	0.085354	0.010296	0.026986	0.033985	0.120765	0.780807	0.629514

Q9D1M0	SEC13	110379	-0.326313	0.226002	0.160724	0.212159	-0.186438	0.260728	0.48094	0.332026	-0.148677	0.288468	0.610988	0.58974
Q9D2G2	ODO2	78920	-0.069805	0.046757	0.136603	0.191322	0.018294	0.065423	0.779972	0.422689	0.095746	0.050474	0.058968	0.194639
Q9D2J7	ANKR5	319196	-0.266409	0.590442	0.659889	0.471852	-0.652055	0.635059	0.324785	0.268394	-1.491463	0.687939	0.058302	0.19405
Q9D3D9	ATPD	66043	0.525725	0.271094	0.056084	0.102142	0.287354	0.285552	0.317503	0.265535	0.201777	0.296518	0.498441	0.541598
Q9D6J5	NDUB8	67264	0.31516	0.324557	0.334565	0.32939	0.813931	0.266758	0.003138	0.010531	0.489388	0.347959	0.163891	0.319316
Q9D6J6	NDUV2	72900	0.205978	0.068926	0.003363	0.011653	0.313038	0.071216	2.27E-05	0.00021	0.236092	0.067991	0.00072	0.009171
Q9D6R2	IDH3A	67834	-0.080035	0.061707	0.196007	0.2361	0.042203	0.080768	0.601842	0.373883	0.144025	0.06852	0.036798	0.149313
Q9D6X6	PRS23	76453	0.738768	0.291185	0.02479	0.055815	0.626056	0.277231	0.039261	0.072158	0.600553	0.358404	0.12197	0.276515
Q9D6Y7	MSRA	110265	-0.459368	0.538595	0.401218	0.362735	-0.327421	0.468432	0.490547	0.334808	0.028646	0.484455	0.953254	0.677987
Q9D6Y9	GLGB	74185	-0.416567	0.136355	0.0049	0.015848	0.103799	0.13961	0.463851	0.326365	0.163833	0.093816	0.092124	0.237757
Q9D7B6	ACAD8	66948	0.012472	0.203134	0.951166	0.571644	-0.104082	0.234008	0.657422	0.388263	-0.013335	0.207232	0.948828	0.677987
Q9D7J4	FA36A	66359	0.49949	0.450773	0.289546	0.302361	0.019309	0.577397	0.973921	0.480762	1.057234	0.443862	0.036379	0.148583
Q9D855	QCR7		0.271407	0.093224	0.004429	0.014679	0.22488	0.091202	0.015325	0.036301	0.332542	0.089826	0.00036	0.005103
Q9D892	ITPA	16434	-0.359626	0.163995	0.039179	0.078301	0.052551	0.157015	0.740766	0.413182	-0.143258	0.113323	0.220023	0.365967
Q9D8B4	NDUAB		0.041594	0.163907	0.801008	0.527693	0.189273	0.16035	0.244493	0.228322	0.031635	0.174187	0.856826	0.657432
Q9D8S4	ORN	104444	-0.474011	0.373894	0.22302	0.26041	-0.570706	0.292154	0.068482	0.102825	-0.662999	0.435209	0.153568	0.310781
Q9D8T7	SLIRP	380773	0.134911	0.24459	0.586335	0.449216	0.246532	0.20708	0.245985	0.228371	0.182609	0.203368	0.378948	0.48868
Q9D8U6	MCEM1	69189	0.498946	0.239799	0.047456	0.090765	0.326547	0.219644	0.149119	0.17336	0.383388	0.217257	0.090343	0.236842
Q9D8W5	PSD12	66997	-0.015625	0.416765	0.970465	0.574757	-0.019902	0.428183	0.963366	0.47784	0.575073	0.385087	0.153671	0.310781
Q9D8Y0	EFHD2		-0.616786	0.291876	0.040427	0.079899	0.106327	0.24477	0.665989	0.391755	0.071098	0.26889	0.79289	0.632968
Q9D967	MGDP1	67881	0.792797	0.544845	0.176306	0.222411	-0.870235	0.556693	0.143975	0.170755	-0.44279	0.58672	0.472066	0.528886
Q9DAK9	PHP14	75454	-0.097443	0.126472	0.447966	0.385609	-0.023118	0.110456	0.835848	0.43866	-0.034107	0.159625	0.832611	0.648427
Q9DB20	ATPO	28080	0.480993	0.08307	2.63E-08	5.84E-07	0.569	0.105242	1.76E-07	3.12E-06	0.492954	0.098328	1.24E-06	5.63E-05
Q9DB29	IAH1	67732	-0.943275	0.423953	0.035341	0.073252	-0.777324	0.310023	0.019028	0.04223	-0.541689	0.388352	0.17584	0.328086
Q9DB77	QCR2	67003	0.168657	0.0775	0.030072	0.065765	0.111558	0.091769	0.224783	0.215976	0.264069	0.086979	0.00255	0.02321
Q9DBB8	DHDH	71755	0.318794	0.297902	0.320067	0.322251	-0.138498	0.815928	0.870791	0.450066	-0.12803	0.747341	0.868824	0.658835

Q9DBG5	PLIN3	66905	0.360584	0.232185	0.127427	0.181326	0.487912	0.264579	0.071907	0.105465	-0.175268	0.337048	0.606154	0.587928
Q9DBJ1	PGAM1	18648	-0.693374	0.080288	1.35E-13	9.02E-12	-0.600774	0.074435	2.01E-12	8.90E-11	-0.241665	0.085803	0.005992	0.044911
Q9DBL1	ACDSB	66885	0.145225	0.198043	0.464921	0.394418	-0.079093	0.233199	0.735126	0.41165	-0.087985	0.216577	0.68537	0.606591
Q9DBP5	KCY	66588	-0.703113	0.169319	0.000314	0.001745	-0.231496	0.123169	0.071429	0.105198	-0.114261	0.138738	0.418291	0.509209
Q9DC61	MPPA	66865	-0.218514	0.135434	0.113344	0.16669	-0.142144	0.15265	0.356423	0.283944	-0.245581	0.150579	0.109737	0.262067
Q9DC69	NDUA9	66108	0.464427	0.11515	8.17E-05	0.000614	0.330988	0.11075	0.003203	0.010668	0.294343	0.118475	0.013968	0.078686
Q9DC70	NDUS7	75406	0.205434	0.23809	0.38993	0.358442	-0.033687	0.22788	0.882723	0.453173	0.247824	0.262783	0.347674	0.468948
Q9DCB8	ISCA2	74316	-0.126796	0.093012	0.184502	0.227901	0.034615	0.111731	0.759176	0.416688	0.134307	0.087829	0.139292	0.295786
Q9DCD0	6PGD	110208	-0.27432	0.179612	0.132526	0.187581	-0.02871	0.133744	0.830899	0.437098	-0.081109	0.151583	0.595174	0.582418
Q9DCJ5	NDUA8	68375	0.031292	0.136151	0.818508	0.531384	0.422353	0.075945	1.04E-07	2.01E-06	0.300745	0.087633	0.000766	0.009556
Q9DCM0	ETHE1	66071	0.357249	0.280768	0.243865	0.273422	0.071297	0.296623	0.815435	0.430497	-0.086542	0.350256	0.810389	0.641313
Q9DCM2	GSTK1	76263	0.224601	0.272318	0.411351	0.367063	0.16444	0.305102	0.59105	0.370294	0.065704	0.204548	0.748731	0.622686
Q9DCS3	MECR	26922	-0.112934	0.307489	0.716768	0.499195	-0.594517	0.241314	0.022044	0.047399	-0.098343	0.256175	0.705321	0.610402
Q9DCS9	NDUBA	68342	0.210063	0.078459	0.008602	0.025082	0.270209	0.085332	0.002015	0.008039	0.279471	0.082676	0.001041	0.012056
Q9DCT2	NDUS3	68349	0.224139	0.121366	0.066106	0.115657	0.415167	0.079179	3.65E-07	6.00E-06	0.278	0.111652	0.013562	0.077833
Q9DCV4	RMD1	66302	-0.200359	0.276402	0.474538	0.400427	-0.126699	0.265109	0.636176	0.382048	0.236706	0.376043	0.534334	0.56171
Q9DCW4	ETFB	110826	-0.077782	0.07359	0.291454	0.30307	-0.132725	0.090802	0.14495	0.170755	0.138498	0.09333	0.139028	0.295786
Q9DCX2	ATP5H	71679	0.2497	0.077823	0.001485	0.005871	0.244306	0.080781	0.002717	0.009629	0.246642	0.080926	0.002537	0.02321
Q9DCZ1	GMPR1	66355	-0.18099	0.245538	0.464365	0.394418	-0.246394	0.26838	0.362587	0.285771	-0.356986	0.280706	0.209123	0.359087
Q9DCZ4	APOO	68316	0.224171	0.195053	0.254925	0.280479	0.249262	0.188751	0.191418	0.200915	0.021851	0.266134	0.934842	0.675981
Q9EPB5	SERHL	68607	-0.150572	0.387153	0.700765	0.492674	0.433704	0.278714	0.131777	0.162136	-0.245199	0.242439	0.32192	0.449734
Q9EPK5	WWTR1	97064	-0.514066	0.904864	0.574831	0.44455	0.781222	0.780881	0.32632	0.26866	-0.320281	0.885845	0.720849	0.612287
Q9EQ20	MMSA	104776	-0.0803	0.070136	0.252851	0.279349	-0.070257	0.06844	0.305183	0.260345	-0.148663	0.077765	0.056598	0.191786
Q9EQI8	RM46	67308	0.275475	0.27394	0.331671	0.32775	0.389822	0.335269	0.267547	0.237978	-0.198184	0.41498	0.643221	0.598925
Q9EQP2	EHD4	98878	-0.137128	0.224369	0.543699	0.428547	0.068728	0.105089	0.515939	0.34496	-0.169982	0.096901	0.085652	0.230229
Q9ERI6	RDH14	105014	0.088768	0.150577	0.562841	0.439095	0.03484	0.166888	0.837269	0.438885	0.103243	0.165521	0.540619	0.56459

Q9ERS2	NDUAD	67184	0.182908	0.104067	0.082141	0.131627	0.327228	0.104074	0.002236	0.008633	0.32509	0.104435	0.002508	0.02321
Q9ESN3	TMM8A	60455	-0.295704	0.130289	0.032114	0.069595	-0.406676	0.134096	0.005435	0.016377	-0.017072	0.165218	0.918561	0.673601
Q9JHI5	IVD	56357	-0.208426	0.057899	0.000371	0.002	-0.131013	0.071438	0.067641	0.101907	-0.16652	0.067627	0.014391	0.07903
Q9JHW2	NIT2	52633	0.014619	0.164689	0.929403	0.564857	0.153648	0.123906	0.217143	0.214634	0.000913	0.165406	0.995604	0.686749
Q9JI75	NQO2	18105	0.309661	0.401261	0.448878	0.385714	-0.477915	0.622186	0.450581	0.320352	-0.571752	0.699737	0.424558	0.512242
Q9JI91	ACTN2		0.497596	0.170245	0.003752	0.012833	0.082017	0.164094	0.617602	0.378182	0.11124	0.180557	0.53838	0.564102
Q9JII6	AK1A1	58810	-0.120636	0.23436	0.6105	0.46062	0.154357	0.152151	0.317728	0.265535	-0.031179	0.158036	0.844976	0.652259
Q9JJ26	MEFV	54483	0.001156	0.099955	0.990858	0.578391	0.122071	0.077563	0.127619	0.159142	0.131924	0.115632	0.265171	0.403167
Q9JJZ2	TBA8	53857	0.626188	0.380497	0.111861	0.165808	0.596721	0.333424	0.085161	0.119749	0.452127	0.320993	0.171799	0.328086
Q9JK42	PDK2	18604	-0.348671	0.175936	0.05458	0.100431	-0.278542	0.204818	0.181656	0.195611	-0.51753	0.238088	0.036385	0.148583
Q9JKB1	UCHL3	50933	-0.022789	0.363352	0.95049	0.571644	0.325512	0.388806	0.410408	0.303806	-0.007596	0.35098	0.982921	0.685835
Q9JKF7	RM39	27393	-0.201173	0.230197	0.390824	0.358442	-0.182062	0.21972	0.415168	0.306489	-0.092741	0.244109	0.70765	0.610402
Q9JKS4	LDB3	24131	0.153725	0.106206	0.149799	0.201856	0.084514	0.108111	0.435554	0.31432	-0.188288	0.138813	0.177214	0.328179
Q9JLT4	TRXR2		0.023366	0.199954	0.907873	0.558398	-0.037975	0.212666	0.859716	0.446896	0.116495	0.252536	0.648738	0.600663
Q9JLZ3	AUHM	11992	0.271599	0.158621	0.336512	0.329888	0.321747	0.291924	0.38529	0.293229	0.182775	0.211757	0.479036	0.530665
Q9JMH6	TRXR1		-0.302401	0.168868	0.080214	0.130498	-0.39721	0.256258	0.127701	0.159142	0.074186	0.113849	0.518114	0.553798
Q9QUH0	GLRX1	93692	-0.142993	0.148736	0.345565	0.335443	0.076708	0.130925	0.562999	0.363196	-0.211269	0.121674	0.095323	0.242901
Q9QUM9	PSA6	26443	-0.211027	0.08245	0.013761	0.035686	-0.217282	0.094156	0.02511	0.051255	0.01457	0.101249	0.886204	0.662623
Q9QVP4	MLRA	17898	-1.790238	0.633395	0.006625	0.020435	-1.027924	0.62433	0.10559	0.137964	-1.650484	0.660942	0.015857	0.083266
Q9QWK4	CD5L	11801	-0.503884	0.453384	0.278402	0.294178	-0.452821	0.50871	0.38261	0.292306	-0.972031	0.52179	0.077234	0.222633
Q9QXW2	FBXW5	30839	0.70355	0.560442	0.235365	0.267779	-0.001254	0.481492	0.998006	0.483648	-0.091882	0.60233	0.883758	0.662348
Q9QXX4	CMC2	50799	0.312579	0.11811	0.008967	0.025724	0.130792	0.10101	0.197243	0.205085	0.043339	0.112468	0.70058	0.608872
Q9QYG0	NDRG2	29811	0.007483	0.129991	0.954149	0.572049	0.060389	0.09852	0.540591	0.355143	0.055079	0.107947	0.610481	0.58974
Q9QYR9	ACOT2	171210	0.023916	0.075677	0.752209	0.509145	-0.023353	0.0965	0.808948	0.429118	0.288368	0.101994	0.005044	0.039672
Q9QZB1	RGS20	58175	-0.894791	1.014434	0.385827	0.358442	-1.460387	0.706337	0.04877	0.080906	-1.498388	1.03085	0.159025	0.317194
Q9R062	GLYG	27357	-0.319365	0.210586	0.135436	0.190186	-0.028746	0.219031	0.896099	0.458332	0.51875	0.285299	0.075138	0.220583

Q9R069	BCAM	57278	-0.123451	0.648613	0.852517	0.538926	1.37366	0.822819	0.138956	0.168167	-0.135249	0.566405	0.815663	0.643187
Q9R0H2	MUCEN	59308	0.0212	0.228708	0.927459	0.564318	0.057603	0.19325	0.770356	0.419704	0.094014	0.196155	0.63865	0.598255
Q9R0P3	ESTD	13885	-0.253285	0.087467	0.004117	0.013816	-0.14641	0.087945	0.09718	0.128877	-0.072282	0.091765	0.431667	0.513754
Q9R0P5	DEST	56431	-0.113925	0.135503	0.40195	0.362735	0.036081	0.145703	0.804786	0.428844	-0.416751	0.163257	0.011891	0.070796
Q9R0X4	ACOT9	56360	-0.23471	0.337425	0.49732	0.410167	0.401759	0.39558	0.321939	0.26754	0.253102	0.308196	0.421696	0.510724
Q9R0Y5	KAD1	11636	-0.063681	0.100383	0.52636	0.424281	-0.183874	0.113091	0.105143	0.137964	0.120933	0.116867	0.301763	0.429821
Q9R111	GUAD	14544	-1.155155	0.409607	0.010575	0.029087	-0.794141	0.39034	0.054725	0.087785	-0.176565	0.32441	0.591733	0.579942
Q9R1P0	PSA4	26441	-1.054482	0.304354	0.004677	0.015311	-0.387771	0.600529	0.526611	0.348143	-0.577363	0.279303	0.056428	0.191786
Q9R1P1	PSB3	26446	-0.291084	0.080804	0.001307	0.005323	-0.195225	0.094127	0.048116	0.080696	-0.601224	0.542482	0.279195	0.417382
Q9R229	BMP10	12154	0.250836	0.440089	0.576616	0.444642	0.480046	0.555402	0.405865	0.301632	0.611352	0.756666	0.433653	0.514307
Q9R244	TRPC2	22064	-0.403137	1.108901	0.720956	0.499195	-0.935686	1.172185	0.436419	0.31432	-0.352134	1.497899	0.817805	0.643187
Q9WTP6	KAD2	11637	-0.121273	0.129869	0.351562	0.338084	-0.212207	0.124076	0.088887	0.122481	-0.096631	0.133874	0.471372	0.528886
Q9WTP7	KAD3	56248	0.105105	0.141192	0.466236	0.394907	-0.069995	0.145658	0.635584	0.382048	-0.016708	0.165939	0.920977	0.673601
Q9WTR5	CAD13	12554	-0.434811	0.315367	0.172018	0.219073	-0.101305	0.245568	0.681094	0.395389	-0.379436	0.333987	0.259743	0.400651
Q9WUB3	PYGM	19309	-0.16411	0.042847	0.000138	0.000923	-0.030432	0.043721	0.486599	0.333127	0.244525	0.051887	2.92E-06	0.00011
Q9WUM5	SUCA	56451	0.007555	0.169526	0.964498	0.573303	0.182248	0.142909	0.203661	0.209128	0.003989	0.150116	0.978826	0.68523
Q9WUR2	PECI	23986	0.259647	0.075623	0.000767	0.003622	0.721991	0.070306	3.19E-19	3.54E-17	0.224026	0.076787	0.004099	0.033478
Q9WUU7	CATZ	64138	-1.365766	0.545418	0.02105	0.049922	-0.915868	0.423889	0.04243	0.074923	-0.331826	0.345099	0.347223	0.468948
Q9WUZ7	SH3BG	50795	0.175603	0.131691	0.193952	0.234685	-0.213738	0.131026	0.11489	0.145939	0.109423	0.148514	0.468393	0.528122
Q9WV35	ABEC2	11811	0.022343	0.205565	0.914315	0.560153	-0.650957	0.226762	0.008037	0.02225	-0.416167	0.219158	0.070188	0.216339
Q9WV98	TIM9	30056	0.29345	0.332277	0.382568	0.356273	0.11811	0.345506	0.734931	0.41165	-0.161351	0.427222	0.70809	0.610402
Q9WVA4	TAGL2	21346	-0.846972	0.322595	0.010167	0.028404	-0.418203	0.338199	0.219267	0.214634	-0.271138	0.352091	0.443413	0.51859
Q9WVH9	FBLN5	23876	-0.289704	0.274575	0.301471	0.309365	-0.090172	0.267494	0.738972	0.41276	-0.446964	0.393223	0.267382	0.405559
Q9WVK4	EHD1	13660	0.344552	0.280752	0.23398	0.267507	-0.248872	0.494938	0.619862	0.378182	-1.099194	0.612908	0.089727	0.236264
Q9WVL0	MAAI	14874	-0.077822	0.22499	0.730876	0.500009	-0.001773	0.182754	0.992298	0.483494	-0.190305	0.180265	0.296393	0.427987
Q9Z0S1	BPNT1	23827	-0.266964	0.129597	0.055052	0.10095	-0.212356	0.135454	0.136505	0.167026	-0.171153	0.115018	0.157457	0.315434

Q9Z0X1	AIFM1	26926	0.194795	0.12612	0.123809	0.177765	0.223028	0.120583	0.065675	0.099965	0.151573	0.144233	0.294508	0.42737
Q9Z126	PLF4	56744	-0.562337	1.239067	0.659635	0.471852	0.764748	1.51011	0.621042	0.378381	-0.981938	1.07066	0.377137	0.487331
Q9Z1E4	GYS1	14936	-0.288131	0.237283	0.227237	0.263032	-0.020607	0.23183	0.929329	0.468299	-0.322396	0.26925	0.233852	0.372437
Q9Z1J3	NFS1		-0.103682	0.186748	0.583508	0.449216	-0.138747	0.224093	0.541208	0.355143	-0.182759	0.228607	0.432211	0.513754
Q9Z1P6	NDUA7	66416	0.364287	0.169326	0.038229	0.07698	0.642337	0.139786	5.14E-05	0.000435	0.351421	0.184137	0.06506	0.206833
Q9Z2I0	LETM1	56384	0.133309	0.121149	0.277916	0.294178	0.065521	0.246508	0.791762	0.424621	0.150071	0.110003	0.180732	0.332761
Q9Z2I8	SUCB2	20917	-0.13296	0.068531	0.054479	0.100431	0.495821	0.063021	6.81E-13	3.77E-11	0.310296	0.061395	1.45E-06	5.76E-05
Q9Z2I9	SUCB1	20916	0.117741	0.046442	0.011713	0.031566	0.13328	0.051509	0.010106	0.026793	0.112612	0.053547	0.03631	0.148583
Q9Z2K1	K1C16	16666	0.56973	0.40014	0.169912	0.218476	-0.02263	0.295109	0.939721	0.471391	-0.130819	0.308436	0.676785	0.605945
Q9Z2U1	PSA5	26442	-0.311817	0.058985	1.58E-05	0.000152	-0.159971	0.079502	0.054671	0.087785	-0.183787	0.287283	0.528401	0.558012
Q9Z2U2	ZN292	30046	0.208745	0.895401	0.817559	0.531384	0.696781	0.707348	0.334032	0.270663	0.026905	0.88569	0.976028	0.684023
Q9Z2W0	DNPEP	13437	-0.171239	0.13154	0.201733	0.241364	0.161354	0.115678	0.17116	0.189464	-0.05386	0.142563	0.70786	0.610402
Q9Z2Y8	PROSC	114863	-0.33794	0.114285	0.006004	0.018878	-0.105695	0.087579	0.235818	0.223667	-0.016001	0.087362	0.855991	0.657432
Q9Z2Z6	MCAT	57279	0.261077	0.078569	0.001184	0.004975	0.438098	0.081871	4.31E-07	6.81E-06	0.312261	0.071488	2.87E-05	0.000762
REFSEQ:NP_001098112			1.048503	0.852973	0.265	0.286957	-0.837548	1.629599	0.62918	0.381671	0.681259	1.126653	0.578019	0.574455
REFSEQ:XP_001473992;XI			0.408389	0.474651	0.398083	0.36188	0.200721	0.474871	0.676287	0.393114	-0.066613	0.482224	0.891334	0.665677
REFSEQ:XP_001476577;XI			-0.249788	0.196995	0.216038	0.253924	-0.225083	0.177267	0.21587	0.214531	-0.012372	0.208278	0.953125	0.677987
REFSEQ:XP_001476714			-0.809974	0.718831	0.271971	0.290256	-2.79092	0.824072	0.002654	0.00948	-1.525095	1.077974	0.172515	0.328086
REFSEQ:XP_001477931;XI			-0.323673	0.293366	0.281305	0.296658	-0.397776	0.344806	0.261034	0.235482	-1.52683	0.743018	0.053186	0.188216
REFSEQ:XP_001479404			0.241172	1.060468	0.822526	0.532016	-0.767829	1.159773	0.517361	0.34496	-0.677652	1.365168	0.626371	0.596846
REFSEQ:XP_207492;XP_3			0.246287	0.675019	0.719468	0.499195	0.713901	0.629853	0.270434	0.239093	0.141866	0.837285	0.867577	0.658747
REFSEQ:XP_484732			-0.350155	0.361084	0.341115	0.333378	-0.087594	0.314392	0.782745	0.422814	-0.060301	0.256795	0.81634	0.643187
REFSEQ:XP_487581;XP_9			-0.563215	0.165399	0.002156	0.008047	-0.539965	0.178032	0.005432	0.016377	0.12486	0.172417	0.475961	0.529163
REFSEQ:XP_894449			0.523483	0.200818	0.015466	0.03893	0.089948	0.464012	0.847925	0.442899	-0.074506	0.574226	0.897943	0.667886
REFSEQ:XP_904967			0.213978	0.14707	0.157655	0.209794	0.176192	0.148614	0.246516	0.228371	0.103807	0.137926	0.458994	0.524957
SWISS-P	TR	11938	-0.201318	0.386473	0.616536	0.462767	0.105478	0.387946	0.791843	0.424621	-0.23735	0.285915	0.424109	0.512242

SWISS-P	TREM		-1.021257	0.379256	0.009209	0.026001	-0.940759	0.426829	0.031573	0.061608	-1.681063	0.428937	0.000256	0.00422
SWISS-PROT:P52		18746	-0.286113	0.096042	0.003866	0.013058	-0.276949	0.095533	0.004891	0.01515	-0.09012	0.096646	0.354211	0.47306
SWISS-P	TR	22004	-0.247091	0.818117	0.767412	0.516398	-0.352526	1.015049	0.73492	0.41165	-0.706203	1.15492	0.554531	0.566127
SWISS-PROT:Q05512-2			0.094638	0.197322	0.637279	0.464984	-0.340664	0.260501	0.207423	0.210724	-0.321342	0.19584	0.120339	0.274772
SWISS-P	EN		1.825848	0.686022	0.016443	0.04081	0.790878	0.861174	0.369936	0.287977	1.192842	0.788461	0.14868	0.303577
SWISS-P	EN		-0.160234	0.405606	0.696297	0.490826	-0.477355	0.240855	0.059562	0.093555	-0.569659	0.469824	0.238183	0.376511
TREMBL:A0JLR7;A0PJF4;(			-0.290585	0.18195	0.116555	0.170398	-0.214924	0.16053	0.186337	0.197339	-0.24971	0.144978	0.091304	0.237757
TREMBL:	E		0.030735	0.171743	0.858444	0.538926	0.172792	0.099383	0.086257	0.120525	-0.019925	0.089375	0.824264	0.645874
TREMBL:	ENSEMBL:		-0.322572	0.463345	0.492493	0.408078	-0.481135	0.658289	0.471924	0.329186	-0.706125	0.527451	0.193735	0.34665
TREMBL:	E		0.315054	0.160145	0.059904	0.107268	0.009717	0.144569	0.946927	0.473397	-0.079151	0.163341	0.632369	0.598255
TREMBL:	ENSEM	545486	-0.317237	0.722842	0.664847	0.473663	-0.121674	0.738169	0.870456	0.450066	-0.23432	0.784494	0.767859	0.629091
TREMBL:	ENSEMBL:		-0.043672	0.088302	0.625056	0.464731	-0.014827	0.082451	0.858685	0.446896	0.058277	0.097527	0.55574	0.566452
TREMBL:	E		0.530281	0.611929	0.391615	0.358442	0.050637	0.550261	0.927163	0.467952	0.075012	0.781542	0.924055	0.675077
TREMBL:	VEGA:OTT		0.533628	0.11972	1.58E-05	0.000152	-0.266112	0.187207	0.157185	0.179904	-0.381071	0.165078	0.022429	0.107431
TREMBL:	ENSEMBL:		-0.127023	0.077039	0.113393	0.16669	0.187823	0.080508	0.027994	0.055853	0.155452	0.087009	0.087787	0.233994
TREMBL:B1AR69;Q3UW38			0.542274	0.250443	0.034224	0.0719	-0.230633	0.223104	0.305207	0.260345	-0.272679	0.269835	0.316661	0.446761
TREMBL:	ENSEMBL:		0.071885	0.754231	0.925018	0.564318	-0.784458	0.707244	0.279332	0.243627	0.259208	0.833958	0.759156	0.628893
TREMBL:	E		1.952323	0.904196	0.040242	0.079829	0.558652	1.074381	0.60748	0.375393	1.753439	0.965016	0.081725	0.226906
TREMBL:	ENSEMBL:		-0.211138	0.082344	0.010711	0.029312	0.739423	0.0922	1.07E-14	9.48E-13	0.001485	0.092158	0.98715	0.686749
TREMBL:B9EHC7;Q56A03			0.227639	0.091599	0.01614	0.040246	0.182243	0.102657	0.081599	0.116968	0.129316	0.100584	0.204609	0.355035
TREMBL:	ENSEMBL:		-0.631184	0.395859	0.134845	0.189856	-0.175554	0.374141	0.647324	0.386224	0.177044	0.412151	0.676623	0.605945
TREMBL:	E		-0.677723	0.417407	0.116514	0.170398	-0.844657	0.28499	0.006586	0.018941	0.058794	0.193923	0.764474	0.629091
TREMBL:O55124;Q14BI5;Q			0.150771	0.047195	0.001474	0.005871	-0.151722	0.054833	0.005836	0.017006	-0.102971	0.065264	0.115193	0.268746
TREMBL:O70181;Q60835;(			1.370362	0.340226	0.000269	0.001558	1.194725	0.349283	0.001538	0.006366	1.059319	0.375132	0.008098	0.057322
TREMBL:	E		-0.399518	0.112674	0.000546	0.00265	-0.332056	0.131142	0.012531	0.031537	-0.116594	0.137176	0.397055	0.499827
TREMBL:	E		0.238245	0.17443	0.183687	0.227901	0.174839	0.136723	0.212272	0.212817	0.385152	0.161569	0.025392	0.117216

TREMBL:	E		-0.128249	0.261701	0.626583	0.46488	0.052356	0.192568	0.786982	0.424068	-0.16731	0.404405	0.681186	0.606591
TREMBL:	E		-0.088382	0.383359	0.819471	0.531384	-0.007222	0.336704	0.983051	0.481892	-0.118719	0.344999	0.733757	0.616276
TREMBL:Q3TLK7			0.746546	0.812445	0.3718	0.350015	-0.071324	0.650932	0.914032	0.465356	0.098405	0.789919	0.902514	0.667886
TREMBL:	E		-0.482059	0.645136	0.461635	0.392881	-0.901573	0.797258	0.268446	0.237978	0.597201	0.594959	0.325504	0.452755
TREMBL:	ENSEMBL:		0.153966	0.304696	0.618864	0.462767	0.522563	0.310785	0.10823	0.140173	-0.023239	0.369821	0.950725	0.677987
TREMBL:Q3UAI4;Q3UBH2;			0.002026	0.128627	0.987555	0.578391	0.060195	0.140823	0.67257	0.393016	0.250723	0.138469	0.082728	0.227924
TREMBL:	ENSEMBL:		-0.007223	0.256641	0.977856	0.575938	-0.123315	0.358236	0.734099	0.41165	-0.165409	0.404583	0.687481	0.606591
TREMBL:Q3UFU2;Q3UJU1			-0.809276	0.641044	0.22897	0.264463	-0.46156	0.398658	0.263944	0.236661	-0.886088	0.483095	0.08961	0.236264
TREMBL:Q3UHC8;Q6JIZ0;(			-0.373674	0.362649	0.312312	0.315857	-0.716803	0.476926	0.144898	0.170755	-0.562425	0.367444	0.138933	0.295786
TREMBL:Q3ULL9;Q8BTY0;			0.120862	0.14225	0.400706	0.362735	0.139884	0.165103	0.401896	0.300621	0.115898	0.157481	0.466962	0.528122
TREMBL:	E		0.389729	0.229981	0.102095	0.157002	-0.126578	0.254248	0.622939	0.379015	-0.321499	0.297123	0.289988	0.424051
TREMBL:	E		-0.065697	0.179113	0.716744	0.499195	-0.231251	0.226789	0.317279	0.265535	-0.390849	0.230885	0.10343	0.257723
TREMBL:	E		-0.255158	1.034994	0.807458	0.528785	0.324691	1.036019	0.7568	0.416688	-0.806774	1.186836	0.50485	0.545444
TREMBL:	ENSEMBL:		-0.829537	1.240901	0.518949	0.420352	-2.017701	0.710597	0.016098	0.037727	-1.233285	1.613772	0.466674	0.528122
TREMBL:Q3V2M1;Q62029;			0.027763	0.242312	0.909662	0.558585	0.097052	0.285457	0.736596	0.411952	0.146286	0.334537	0.665819	0.605694
TREMBL:	ENSEMBL:		0.527024	0.544685	0.344269	0.335232	-0.433839	0.732604	0.559763	0.362485	0.154221	0.890697	0.864277	0.657432
TREMBL:	E		-0.24377	1.192393	0.847992	0.538926	-2.706665	2.191115	0.284317	0.246448	-3.45924	1.406533	0.05727	0.19202
TREMBL:Q5FW75;Q8K3Q4			0.581329	0.255026	0.025522	0.057222	-0.2362	0.229947	0.307721	0.261114	-0.544488	0.27174	0.049087	0.179718
TREMBL:	ENSEMBL:		-0.513879	0.60195	0.406704	0.364746	-0.430117	0.588423	0.474209	0.330259	-0.720055	0.672146	0.30098	0.429821
TREMBL:Q60977			-0.008188	0.274739	0.976677	0.575938	0.133999	0.276465	0.634085	0.382048	0.182879	0.250639	0.478544	0.530665
TREMBL:Q69ZN8;Q6PDJ2;			-0.07361	0.351356	0.835689	0.537282	0.284665	0.329407	0.395395	0.298866	-0.047557	0.422577	0.911331	0.671169
TREMBL:	E		-0.335779	0.2971	0.270045	0.289358	-0.307903	0.35904	0.399612	0.300515	-0.365742	0.345012	0.300606	0.429821
TREMBL:	E		0.1162	0.642987	0.85799	0.538926	-0.941911	0.56412	0.106977	0.139365	-0.744708	0.700693	0.298443	0.42917
TREMBL:Q6P9Q7			-0.268592	0.190408	0.164201	0.213708	-0.380557	0.256579	0.143946	0.170755	-0.227189	0.207057	0.277903	0.417382
TREMBL:	REFSEQ:X		0.219554	0.158515	0.177801	0.222854	0.457635	0.135793	0.002356	0.008769	0.497921	0.138215	0.001429	0.015967
TREMBL:	E		0.287671	0.295022	0.344032	0.335232	0.203459	0.270365	0.464196	0.326365	-0.775241	0.359427	0.047649	0.177956

TREMBL:	E		-0.181978	0.27449	0.528571	0.42478	-0.264482	0.20554	0.234157	0.222569	-0.02142	0.186404	0.910788	0.671169
TREMBL:	E		0.078774	0.156104	0.618072	0.462767	-0.29485	0.325013	0.372965	0.289298	-0.105672	0.157243	0.507983	0.546638
TREMBL:	E		-0.614153	1.053408	0.585187	0.449216	-1.849911	1.1871	0.194153	0.202825	-1.992125	1.039711	0.195425	0.346783
TREMBL:	E		0.866573	0.404417	0.041663	0.081437	0.883198	0.436115	0.053233	0.086055	0.256988	0.472165	0.591273	0.579942
TREMBL:	ENSEMBL:		0.497835	0.187118	0.020771	0.049481	0.066678	0.236314	0.781951	0.422814	0.035584	0.203608	0.864178	0.657432
TREMBL:	E		-0.150186	0.446718	0.740223	0.504463	-0.168532	0.396403	0.674855	0.393114	0.47795	0.387634	0.231203	0.371747
TREMBL:Q80Y62;Q8BU90;			0.102152	0.201435	0.616897	0.462767	0.23069	0.241608	0.348815	0.279897	0.054604	0.248099	0.827832	0.647873
TREMBL:	ENSEMBL:		-0.907944	1.314521	0.509292	0.417461	-0.754604	1.03767	0.479989	0.332026	-3.457627	1.347902	0.04261	0.165515
TREMBL:Q8C801			-0.105592	0.543597	0.847766	0.538926	-1.452631	0.749131	0.06673	0.101223	-1.149996	0.736829	0.13353	0.288727
TREMBL:Q8C8U6			-0.626621	0.689888	0.430686	0.378948	NA	NA	NA	NA	-1.166725	1.085322	0.394845	0.499827
TREMBL:	ENSEMBL:		-0.022604	0.135425	0.867659	0.540614	0.093269	0.126798	0.463061	0.326365	0.153898	0.181459	0.397733	0.499827
TREMBL:	ENSEMBL:		-0.577369	0.624135	0.524766	0.423817	-0.387846	NaN	NaN	NA	0.77412	NaN	NaN	NA
TREMBL:	ENSEMBL:		0.002279	0.112193	0.983947	0.578249	-0.147252	0.149791	0.334643	0.270663	0.106828	0.12199	0.389871	0.496733
TREMBL:	ENSEMBL:		-0.141777	0.118883	0.243808	0.273422	-0.154411	0.123212	0.221282	0.214634	0.131106	0.129288	0.320667	0.449734
TREMBL:	ENSEMBL:		-0.662818	0.745338	0.537264	0.427823	-1.971803	1.682086	0.361834	0.285686	-0.611045	0.745338	0.562826	0.56781
TREMBL:	ENSEMBL:		-0.058201	0.052301	0.26663	0.288013	0.114845	0.044205	0.009813	0.026183	0.253124	0.044972	4.23E-08	2.45E-06
TREMBL:Q922F6			0.105015	0.169411	0.541176	0.427823	-0.850481	0.262259	0.003734	0.012162	-0.079376	0.167239	0.639531	0.598255
TREMBL:	ENSEMBL:		0.103191	0.138276	0.456248	0.390162	-0.101407	0.152454	0.506637	0.341876	-0.2133	0.160909	0.186437	0.337335
TREMBL:	ENSEMBL:		1.188912	0.525385	0.151982	0.203258	1.042126	0.342204	0.055633	0.088322	-0.167486	0.525385	0.780101	0.629514
TREMBL:	ENSEMBL:		0.401622	0.612305	0.518679	0.420352	0.566889	0.696605	0.424112	0.309481	0.411898	0.715403	0.57279	0.571935
TREMBL:	ENSEMBL:		-1.731192	1.627424	0.480337	0.401749	NA	NA	NA	NA	-1.471135	1.627424	0.532083	0.560269
VEGA:OTTMUSP00000026			0.103588	0.347137	0.769488	0.516411	-0.382415	0.456521	0.421784	0.308465	-0.353738	0.430784	0.430701	0.513754
VEGA:OTTMUSP00000027			-0.098188	0.095145	0.311582	0.315857	0.162278	0.118935	0.184128	0.195702	0.092576	0.102187	0.373969	0.486197
VEGA:OTTMUSP00000031			-0.763115	0.168475	0.000116	0.000817	0.287018	0.208299	0.179974	0.195076	0.092138	0.157218	0.563311	0.56781
VEGA:OTTMUSP00000032			0.679849	0.283409	0.028193	0.061911	0.290733	0.269376	0.296463	0.25498	0.045925	0.293888	0.878054	0.6598
VEGA:OTTMUSP00000036			0.351976	0.257834	0.18816	0.230287	-0.015051	0.283076	0.958256	0.476372	-0.593107	0.385852	0.141652	0.29732

VEGA:OTTMUSP00000041	-0.777339	0.148006	0.013443	0.035165	-0.26807	0.378311	0.517686	0.34496	-0.690409	0.33737	0.110134	0.262067
ENSEMBL:ENSMUSP00000	1.419829	0.685567	0.072114	0.12063	1.340839	0.874609	0.169124	0.187748	2.107221	0.954698	0.078379	0.222907
ENSEMBL:ENSMUSP00000	0.132802	0.363463	0.717783	0.499195	-0.525371	0.377168	0.175436	0.19187	-0.449063	0.484187	0.362923	0.480664
ENSEMBL:ENSMUSP00000	0.520524	0.138011	0.00099	0.004296	0.616115	0.138394	0.000154	0.00112	0.397349	0.184153	0.041633	0.162713
ENSEMBL:ENSMUSP00000	0.05393	0.296035	0.856858	0.538926	0.023936	0.423542	0.955365	0.476372	0.428613	0.305684	0.173675	0.328086
ENSEMBL:ENSMUSP00000	0.014424	2.258186	0.995483	0.579287	0.056718	3.815155	0.990536	0.483494	-0.45422	2.28522	0.86082	0.657432
ENSEMBL:ENSMUSP00000	-0.553861	0.372967	0.1717	0.219073	-0.691427	0.313982	0.04267	0.075001	-0.028212	0.33298	0.933876	0.675981
ENSEMBL:ENSMUSP00000	-1.495132	1.331045	0.287563	0.301981	-1.064497	2.415742	0.674899	0.393114	-2.234123	1.689029	0.222485	0.366964
ENSEMBL:ENSMUSP00000	0.546961	0.512914	0.321644	0.323209	0.476607	0.763173	0.566148	0.363196	-0.659509	0.939416	0.521354	0.554907
ENSEMBL:ENSMUSP00000	0.334569	0.208543	0.120723	0.175054	0.209951	0.256313	0.420164	0.308123	-0.681077	0.531898	0.212619	0.362091
ENSEMBL:ENSMUSP00000	-0.779233	0.39796	0.061034	0.108925	-0.800139	0.386427	0.048461	0.080696	-0.319275	0.33642	0.352061	0.471176

STAB 3. Continued

Protein#	Peptide
A2A9C3	R.IPMGQAVNSQLSLLFTEECDK.V
A2AKY4	N.LAPFEDQLPMEAVIVNEDGPVSK.S
A2AMM0	R.VVIFQEDIPCPASLSVVK.D
A2AQP0	R.VIFQLPGER.G
A2AR50	R.GLTHTSSTAITNGLSLGSSESSEFSEEMSAGLESR.G
A2ASS6	A.ANLIVEEEDLR.I
A3KG59	R.FFECEPPAASWAVQPHFGLPTAFR.A
A3KMP2	R.VYPFWTPDIPLNSYVK.G
A6H6E2	R.AWFELIQGSATK.G
B2RY56	K.LGASNSPGQPNSVK.R
O08528	K.CDVSFLESEDGSGK.G
O08553	K.GTVVYGEPITASLGTDGSHYWSK.N
O08600	R.ASGLLFVPNILAR.A
O08638	K.DVASLGSQLQDTQELLQEETR.Q
O08677	G.EEAQEIDCNDEAVFQAVDFSLK.Q
O08709	K.DFTPVCTTELGR.A
O08749	H.ILGPGAGEMVNEAALALEYGASCEDIAR.V
O08756	K.IHTLEDFQR.V
O08807	R.QITLNDLPVGR.S
O08997	K.AVSYLGPK.-
O09061	K.AMTTGAIAAMLSTILYSR.R
O09111	K.IQLPEDD.-
O09131	K.MTLESFSKVPPLIASFVR.S

O09161	K.LEVQAFER.I
O35129	R.AQFLVEK.A
O35215	K.FLTEELSLDQDR.I
O35459	K.CPKPVIAAIHGGCIGGGVDLVSACDIR.Y
O35593	R.LGGGMPGLGQGPPTDAPAVDTAEQVYISSLALLK.M
O35639	K.GTGTDEDALIEILTTR.S
O35643	R.LGAPISSGLSDLFDLTSGVGTLSGSYVAPK.A
O35658	K.AFVEFLTDEIKEEK.K
O35678	R.LTLPFLLLQGSADR.L
O35683	R.VQYQWYLMER.D
O35841	K.HFPELADSAINAQLDLCEDEDVSIR.R
O35855	K.AADLQIQMTK.E
O35887	K.EEIVDKYDLFVGSQATDFGEALVR.H
O35943	K.LGGDLGTYVINK.Q
O35963	K.IQLWDTAGQER.F
O54724	K.IIGAVDQIQLTQAQLEER.Q
O55023	K.LQVSQQEDITK.S
O55103	K.SLSLQEGDQLLSAR.V
O55126	K.DAHSNLLAK.K
O55143	K.CHQYDGLVELATICALCNDSALDYNEAK.G
O55237	R.WGAGPALGR.S
O70250	K.HLEGMSDQAIMELNLPTGIPIVYELDQNLKPTKPMR.F
O70251	K.TPAGLQVLNDYLADK.S
O70370	K.NSWGLNFGDQGYIR.M
O70433	K.CAGCTNPISGLGGTK.Y
O70456	R.VLSSIEQK.S

O70468	K.ALDFSEAPSFTQPLANR.S
O88342	K.AHDGGIYAISWSPDSTHLLSASGDK.T
O88441	K.TLDQVLEDVDQCCQALSQR.L
O88492	K.DTVTTGLTGAVNVAK.G
O88587	R.YLPDTLLLEECGLLR.K
O88668	R.IWVLDYFGGPK.V
O88685	R.QTYFLPVIGLVDAEK.L
O88799	R.TTGPSLTGTSGPPGGYPNGEGYYLHMDPK.T
O88844	K.GWPLYLSTK.N
O89053	R.DAGPLLISLK.D
P00158	R.DVNYGWLIR.Y
P00329	K.INEAFDLLR.S
P00397	K.VFSWLATLHGGNIK.W
P00405	R.ILYMMDEINNPVLTVK.T
P00493	K.VIGGDDLSTLTGK.N
P00920	K.AVQQPDGLAVLGIFLK.I
P01027	G.IPMYSIITPNVLR.L
P01680	R.TSNLASGVPAR.F
P01837	K.DSTYSMSSTLTLTKDEYER.H
P01843	K.ATLVCTITDFYPGVVTVDWK.V
P01864	K.NTATVLDSDGSYFMYSK.L
P01867	K.CPAPNLEGGPSVFIFPPNIK.D
P01872	K.STGKPTLYNVSLIMSDTGGTCY.-
P01942	A.AGHLDDLPGALSALSDLHAHK.L
P02088	F.NDGLNHLDSLK.G
P02089	K.VNPDEVGGEALGR.L

P02535	K.QSLEASLAETEGR.Y
P03888	K.GPNIVGPYGILQPFADAMKLFMK.E
P03893	R.LIYSTSLTMFPTNNNSK.M
P03903	K.VSNTYGTDYVQNLNLLQC.-
P03911	K.LITGLTM.-
P03921	K.ANPYSSFSTLLGFFPSIIHR.I
P03930	K.IYLPHSLPQQ.-
P03976	R.FSSSGSGTDFTLR.I
P03987	K.DALMISLTPK.V
P04104	K.SLNDKFASFIDK.V
P04117	K.LGVEFDEITADDR.K
P04247	A.AEIQPLAQSHATK.H
P05064	K.ELSDIAHR.I
P05125	A.NPVYSAVSNTDLMDFK.N
P05201	K.ESDSVLR.V
P05202	A.SSWWTHVEMGPPDPILGVTEAFKR.D
P06151	K.DLADELALVDVMEDKLK.G
P06537	R.ILLDFSKGSASNAQQQQQQQPQPDLSK.A
P06728	A.EVTSDQVANVVWDYFTQLSNNAK.E
P06745	K.EVMQMLVELAK.S
P06801	R.AIFASGSPFDPVTLPDGR.T
P06909	K.VYVQGQSLK.V
P07309	K.TAESGELHGLTTDEK.F
P07310	F.LVWVNEEDHLR.V
P07356	K.ELPSALKSALSGHLETVILGLLK.T
P07724	C.FSALTVDETYVPK.E

P07759	K.AVLDVAETGTEAAAATGVIGGIR.K
P07901	K.HLEINPDHSIIETLR.Q
P08003	K.IDATSASMLASK.F
P08074	R.GVPGSIVNVSSMVAHVTFPNLITYSSTK.G
P08113	K.GVVDSDDLPLNVSR.E
P08226	R.LQAEIFQAR.L
P08228	I.SLSGEHSIIGR.T
P08249	K.AGAGSATLSMAYAGAR.F
P08752	R.IAQSDYIPTQQDVLR.T
P09103	A.DALEEEDNVLVLK.K
P09405	K.VEGSEPTTPFNLFIGNLNPNK.S
P09411	K.ALESPERPFLAILGGAK.V
P09528	K.HTLGHGDES.-
P09541	K.EAFSLFDR.T
P09542	K.DTGTYEDFVEGLR.V
P09671	H.KHSLPDLPYDYGALEPHINAQIMQLHHSK.H
P09813	K.THEQLTPLVR.S
P10107	K.CLTTIVK.C
P10126	K.DGSASGTTLLEALDCILPPTRPTDKPLR.L
P10493	R.VPQIPYGASVHIEPYTELYHYSSSVITSSSTR.E
P10605	K.NGPVEGAFTVFSDFLTYK.S
P10639	K.EAFQEALAAAGDK.L
P10649	K.HHLDGETEEER.I
P11087	R.GFSGLDGAK.G
P11352	K.YVRPGGGFEPNFTLFEK.C
P11404	I.LTLTHGSVVSTR.T

P11438	K.ALQATVGNSYK.C
P11499	K.HLEINPDHPIVETLR.Q
P12246	K.APPSIVLGQEQDNYGGGFQR.S
P12367	K.MFESFIESVPLFK.S
P12382	R.VFANAPDSACVIGLR.K
P12710	K.AIGLPEDLIQK.G
P12787	F.NKPDIDAWELR.K
P12979	K.VNEAFEALK.R
P13020	K.NWRDPDQTDGPGLGYLSSHIANVER.V
P13634	K.ADGLAILGVLMK.V
P13707	K.LISEVIGER.L
P14069	K.LQDAEIAR.L
P14094	K.SYEAYVLNIIR.F
P14131	K.LLEPVLLLGK.E
P14152	I.VVGNPANTNCLTASK.S
P14211	K.EQFLDGDAWTNR.W
P14602	K.YTLPPGVDPTLVSSSLSPEGTLTVEAPLPK.A
P14685	K.LQLDSPEDAEFIVAK.A
P14824	K.DLIEDLKYELTGK.F
P14869	K.AFLADPSAFAAAAPAAAATTAAPAAAAAPAK.A
P15327	K.LNNDGLEEAR.N
P15626	K.VTYVDFLVYDVLDQHR.I
P16015	K.GDNQSPIELHTK.D
P16045	K.DSNNLCLHFNPR.F
P16125	K.GEMMDLQHGSLFLQTPK.I
P16332	K.ILFDGIPLEK.M

P16546	K.IAALQAFADQLIAVDHYAK.G
P16858	E.GLMTTVHAITATQK.T
P17182	K.DATNVGDEGGFAPNILENK.E
P17183	R.DGKYDLDFK.S
P17563	K.CGPGYSTPLEAMK.G
P17665	K.NLPFSVENK.W
P17710	K.ATDCVGHDVATLLR.D
P17742	K.FEDENFILK.H
P17751	K.CLGELICTLNAANVPAGTEVVCAPPTAYIDFAR.Q
P17950	R.ELYLSAK.V
P18242	K.AIGAVPLIQGEYMIPCEK.V
P18525	K.LVESGGGLVQPGGSLK.L
P18572	K.VLQEDTLPDLHTK.Y
P18760	K.EILVGDVGQTVDDPYTTFVK.M
P18826	K.AALEALDELDLFGVK.G
P18872	R.LFDVGGQR.S
P19096	K.VGDPQELNGITR.S
P19123	K.AAFDIFVLGAEDGCISTK.E
P19157	K.YVTLIYTNYENGK.N
P19536	K.AASGTKEDPNLVPSISNK.R
P19783	K.ADWSSLSR.D
P20029	K.DAGTIAGLNVMR.I
P20065	K.NPLPSKETIEQEK.Q
P20108	K.ANEFHDVNCEVVAVSVDSHFSHLAWINTPR.K
P20152	K.FADLSEAANR.N
P21107	K.MELQEIQLK.E

P21300	K.MPLVGLGTWK.S
P21550	K.DATNVGDEGGFAPNILENNEALELLK.T
P21614	K.FSSSTFEQVNQLVK.E
P21956	K.VAYSLDGR.K
P21981	K.LVVNFQCDK.L
P22599	R.LSISGDYNLK.T
P22907	R.GAQLAAENLGISLASLLLNK.G
P23242	R.SDPYHATTGPLSPSK.D
P23492	K.MLGADAVGMSTVPEVIVAR.H
P23927	K.HFSPEELK.V
P23953	G.HSLLPPVVDTTQGK.V
P24270	K.EAETFPFNPFDLTK.V
P24452	K.VSDATGQMNLTK.V
P24472	K.EKEESYDLILSR.A
P24527	K.SLSNVIAHEISHSWTGNLVTNK.T
P25444	K.SLEEIYLFSLPIK.E
P26039	K.VGAIPANALDDGQWSQGLISAAR.M
P26041	K.ALTSELANAR.D
P26443	K.DIVHSGLAYTMER.S
P27005	K.ALSNLIDVYHNYSNIQGNHHALYK.N
P27773	A.SDVLELTDENFESR.V
P28271	K.AVLAESYER.I
P28474	K.AAVAWEAGKPLSIEEIEVAPPK.A
P28650	K.VEVEYETLPGWK.A
P28656	K.GIPEFWLTVFK.N
P29391	K.TQEAMEAALAMEK.N

P29699	K.ANLMHNLGGEEVSVACK.L
P29758	K.LPSDVVTSVR.G
P29788	R.FEDGVLDPGYPR.N
P30285	R.DPHSGHFVALK.S
P31001	K.LQEEIQLR.E
P31786	K.QATVGDVNTDRPGLLDLK.G
P32261	R.DIPVNPLCIYR.S
P34884	K.LLCGLLSDR.L
P34914	K.ACGANLPENFSISQIFSQAMAAR.S
P35441	K.GDVNDNFQGVLQNVR.F
P35486	K.EIEDAAQFATADPEPPLEELGYHIYSSDPPFEVR.G
P35505	K.SFGTTISPWVVPMDALMPFVVPNPK.Q
P35700	K.DISLSEYK.G
P35969	K.LGDLLQANVQQDGK.D
P35979	R.CTGGEVGATSALAPK.I
P35980	K.ILTFDQLALESPK.G
P37804	K.QMEQVAQFLK.A
P38060	K.EVSVFGAVSELFTR.K
P38647	K.DAGQISGLNVLR.V
P40124	K.GAVPYVQAFDSLLANPVAEYLK.M
P40142	K.AVELAANTK.G
P41216	K.AILDDLLK.L
P41251	K.IPIPSADQGTFSLR.K
P42125	D.GALQLGTLFSPAEALK.V
P43023	F.HNPHVNPLPTGYEHP.-
P43024	R.TKPFPWGDGNHTLFHNPHVNPLPTGYEDE.-

P43274	K.TSGPPVSELITK.A
P45376	K.EVGVALQEK.L
P45377	K.ALGVSNFNHFQIER.L
P45591	K.HEWQVNGLDDIKDR.S
P45952	K.AAHKQEPGLGFSFELTEQQK.E
P46412	K.FLVGPDGIPVMR.W
P47708	R.CVHLAAMDANGYSDPFVK.L
P47738	K.DGMTIAKEEIFGPVMQILK.F
P47754	K.FTVTPSTTQVVGILK.I
P47857	K.AIAVLTSGGDAQGMNAAVR.A
P47934	K.AYNNLIK.D
P47955	K.AAGVSVEPFWPGLFAK.A
P48036	K.ALLLLCGGEDD.-
P48758	K.IGVTVLSR.I
P48771	K.GGASDALLYR.A
P48772	K.TPTSAVEQAVGISAIVVGFMVPAGWVLAHLESYKK.S
P48774	K.LTFVDFLTYDVLDQNR.I
P48787	K.NIDALSGMEGR.K
P48962	G.DQALSFLKDFLAGGIAAAVSK.T
P49722	K.AANGVVLATEK.K
P49817	K.HLNDDVVK.I
P50136	R.AVAENQPFLIEAMTYR.I
P50171	R.LAAEGAAVAACDLDGAAAQDTVR.L
P50247	K.DGPLNMILDDGGDLTNLIHTK.Y
P50396	K.QLICDPSYIPDR.V
P50446	R.TAAENEFVTLK.K

P50462	K.ALDSTTVAAHESEIYCK.V
P50543	K.DGNNTQLSK.T
P50544	F.CLTEPSSGSDVASIR.S
P50752	K.AKELWQSIHNLEAEKFDLQEK.F
P51174	E.PGAGSDLQGVR.T
P51667	K.GADPEETILNAFK.V
P51881	K.DFLAGGVAAAISK.T
P51885	K.LPAGLPTSLLTLYLDNNK.I
P52480	K.CCSGAIIVLTK.S
P52503	I.ACDGGGGALGHPK.V
P52825	K.CLEDMFDALEGK.A
P52850	R.YSTELAPGR.G
P53395	K.AASLGLLQFPILNASVDENCQNITYK.A
P53810	R.VILPVSVDEYQVGQLYSVAEASK.N
P53986	K.SDANTDLIGGSPK.G
P54071	A.GCIHGLSNVK.L
P54116	K.NSTIVFPLPVDMLQGIMGSNH.-
P54726	R.QVIQQNPALLPALLQQLGQENPQLLQQISR.H
P54822	R.IRADAYFSPIHSQLEHLLDPSSFTGR.A
P55264	R.NWVLVEK.A
P56375	K.LEYSDFSIR.Y
P56376	K.VIANLDYSDFQIVK.-
P56379	-.MFQTLIQK.V
P56380	K.MDNSTIEFLLLQASDGIHHWTPPK.G
P56382	R.DALKTEFK.A
P56391	K.NCWQNYLDFHR.C

P56392	F.QADNDLPVHLK.G
P56399	K.IVILPDYLEIAR.D
P56480	I.YPAVDPLDSTSR.I
P56501	K.FLGAGTAACFADLLTFPLDTAK.V
P56565	K.DLLQTELSGFLDVQKDADAVDK.V
P56716	R.LEELEDGK.S
P58252	K.EGALCEENMR.G
P58771	K.AISEELDHALNDMTSI.-
P58774	K.LDKENAIDR.A
P59017	K.QQGPSPPGVQLDVAPQSLNPEVLLK.L
P59511	K.CVNEQNVQANESLCDPLTKPLSIK.K
P59997	R.VLMQHCPAR.N
P60335	R.LVVPATQCGSLIGK.G
P60766	K.NVFDEAILAALEPPEPK.K
P61014	K.VQYLTR.S
P61027	K.AFLTLAEDILR.K
P61089	R.LLAEPVPGIK.A
P61148	K.AILFLPLPVSSD.-
P61971	K.IQHSITAQDHQPTPDSCIISMVVGQLK.A
P61979	R.IITITGTQDQIQNAQYLLQNSVK.Q
P61982	K.ELEAVCQDVLSLLDNYLIK.N
P62075	K.LDPGAIMEQVK.V
P62082	K.DVNFEFPEFQL.-
P62204	K.EAFSLFDKDGDGTITTK.E
P62245	K.ILGFFF.-
P62259	K.AAFDDAIAELDTLSEESYK.D

P62270	K.YSQVLANGLDNK.L
P62631	K.ADPPQEAAQFTSQVIILNHPGQISAGYSPVIDCHTAHIACK.F
P62806	K.TVTAMDVVYALK.R
P62814	R.GPVVLAEDFLDIMGQPINPQCR.I
P62821	K.EFADSLGIPFLETSAK.N
P62827	K.LVLVGDGGTGK.T
P62855	R.DISEASVFDAYVLPK.L
P62869	R.ADDTFEALR.I
P62897	K.GITWGEDTLMEYLENPK.K
P62932	K.SQELPATMEMTGLAPWQDGVLER.L
P62962	K.TFVSITPAEVGVLVGK.D
P63017	K.CNEIISWLDK.N
P63028	K.GKLEEQKPER.V
P63030	R.LINYEMSK.R
P63038	K.CEFQDAYVLLSEK.K
P63101	K.FLIPNASQPESK.V
P63158	K.IKGEHPGLSIGDVAK.K
P63325	R.IAIYELLFK.E
P63330	K.ELDQWIEQLNECK.Q
P67778	K.AAELIANSLATAGDGLIELR.K
P68037	K.TDQVIQSLIALVNDPQPEHPLR.A
P68040	R.YWLCAATGPSIK.I
P68254	K.AVTEQGAELSNEER.N
P68368	R.AVFVDLEPTVIDEIR.N
P68510	K.ELETVCNDVLALLDK.F
P70168	K.GALQYLVPILTQTLTK.Q

P70188	K.KLSIFMENKNDMVEMDIVEK.L
P70195	K.VTPLEIEVLEETVQTMDTS.-
P70296	K.GNDISSGTVLSDYVGSGPPSGTGLHR.Y
P70349	K.CAADLGLK.R
P70392	R.ALSQDDQDDIHLK.L
P70398	R.ELDMEPYTVAGVAK.L
P70404	K.AVLASMDNENMHTPDIGGQGTTSQAIQDIIR.H
P70670	K.GSALSGASSPLYPLEVSFLPEAGLAVQGPK.G
P70695	R.VPLILGSPEDVQEYLSCVQR.N
P80313	R.INALTAASEAACLIVSVDETIK.N
P80314	R.VQDDEVGDGTTSVTVLAAELLR.E
P80316	K.LGFAGVVQEISFGTTK.D
P80317	K.NAIDDGCVVPGAGAVEVALAEALIK.Y
P84244	R.FQSAAIGALQEASEAYLVGLFEDTNLCAIHAK.R
P85094	K.EPVPDSGLLSLFQGQSPLTSC.-
P97352	K.TLDVNQDSELR.F
P97372	R.KIISLSQLLQEDSLNVADLSSLR.A
P97429	K.GLGTDEDAIIGILAYR.N
P97443	K.QEPVFADTNLYVLR.L
P97450	R.QASGGPVDIGPEYQQDLDRELYK.L
P97467	K.SALIQQPK.Q
P97807	K.DMLGPK.-
P97823	K.ALINPANVTFK.I
P99024	K.FWEVISDEHGIDPTGTYHGDSDLQLDR.I
P99027	K.LASVPAGGAVAVSAAPGSAAPAAGSAPAAAEEK.K
P99028	R.EHCEQLEK.C

P99029	K.ALNVEPDGTGLTCSLAPNILSQL.-
Q00519	K.ITYEDLPAIITIQDAIK.N
Q00623	K.VAPLGAELQESAR.Q
Q00897	K.AVLTIDETGTEAAAATVLQVATYSMPPIVR.F
Q00915	K.EFEEDLTGIDDR.K
Q01768	K.DRPFFPGLVK.Y
Q01853	K.GDDLSTAILK.Q
Q02053	K.AVTLHDQGTTQWADLSSQFYLR.E
Q02566	K.APGVMDNPLVMHQLR.C
Q03265	F.AQFGSDLDAATQQLLSR.G
Q04447	K.DLFDPIIEER.H
Q04857	K.VFSVAITPDHLEPR.L
Q05816	K.ELGVGLALR.K
Q05920	K.DFTATFGPLDSLNTR.L
Q06185	M.VPPVQVSPLIK.F
Q06770	K.HYYESEALTIPSK.D
Q06890	R.ASGIIDTLFQDR.F
Q06986	K.WQGSLEAVMSHLMHAHK.S
Q07417	K.ELVPIAAQLDR.E
Q08857	R.EVVLEEGTTAFK.N
Q08AU7	R.GGFGGVQGTFCMAAWGFCFAFSVLVVACEFTK.L
Q09LZ8	K.IAPMLNPDGVYLGNYR.C
Q0KK59	R.LLSQFGIWFLVSLCTPSENTPTESLAR.L
Q11011	K.LEAAAQVR.Q
Q19LI2	K.ELDNPGPFTCR.Y
Q1XH17	K.AVAVVAQQLLSQGEHYWEVEVGDKPR.W

Q2TPA8	K.DSLSDEVVR.A
Q30D77	R.QGLAGPEGNPGSK.G
Q3LAC4	K.QAKPKISPLHSSDFCPTNCHVNVMEVSYPK.T
Q3TC72	K.TFDTFCPLGPALVTK.D
Q3TEI4	K.GQPLDGTFLRGLPSGGPGK.D
Q3TP92	K.LWSFFIYLLR.R
Q3TTY5	R.DYQELMNTK.L
Q3TZ89	K.ILWQFLK.V
Q3U0B3	R.TVGNIEELAAECK.S
Q3U132	V.LEEEAAAALQDALDPEPSSLDDVFQEGNPINK.I
Q3UGR5	K.DGLALGPGPFVTALEYATDTK.A
Q3UIU2	R.FWDNFLR.D
Q3ULD5	K.SGVTDHYALDDHHALHLTR.K
Q3ULJ0	K.LPENVVAVPNLSEAVQDADLLVFVIPHQFIHK.I
Q3UM45	R.AIENIDTLTNLESLFLGK.N
Q3UWA4	K.DLDASTLK.D
Q3V3A1	P.SQLYQLPDEESLFAVSGVKLKPEMCDLSASYR.K
Q4ACU6	R.SLGEEPVGGLGSLLDPAK.K
Q4KML4	K.FGVLFQDDR.C
Q505D7	R.EELQEVR.G
Q52KG5	R.DPPGPVGLMGR.Q
Q571E4	R.EPAIAWWPGHIAAGQVSHQLG.S
Q5DU05	K.LTSSSSSQTVDDFLLEK.W
Q5EBG6	K.HFLPEEISVK.V
Q5SWD9	R.TLNVNSLLHIVGHGDFQMNQIDAPVDPFPLNPR.V
Q60590	K.YEGGVETFAHLIVLR.K

Q60597	H.AQSLVEAQPNVDK.L
Q60605	K.EAFQLFDR.T
Q60692	R.LAAIQESGVER.Q
Q60759	K.TWITNSPVADLFIVWAR.C
Q60854	K.FKLEENYNMNDALYK.L
Q60864	K.LDPQNHVLYSNR.S
Q60930	K.GFGFGLVK.L
Q60931	K.AADFQLHTHVNDGTEFGGSIYQK.V
Q60932	K.GYGFGLIK.L
Q60936	K.VALLDFGATR.E
Q60988	R.NLSSNLNISQVQGTYKHGYITMDETRK.L
Q60994	K.AVLFTYDQYQEK.N
Q61037	R.DLAVHSASLEDVK.T
Q61085	R.GDSPVLIAAVVPGGQAESAGLK.E
Q61147	K.ALYFEYTDGTFSK.T
Q61171	K.EGGLGPLNIPLLADVTK.S
Q61207	K.EVVDSYLPVILDMIK.G
Q61233	K.AYYHLLEQVAPK.G
Q61292	R.LQELEGTYEENER.A
Q61316	K.FLEMCDDLLAR.V
Q61425	H.TVVLVDQTEDILAK.S
Q61554	K.NCVDINECVLNSLLCDNGQCR.N
Q61598	K.DLGTDSQIFISR.A
Q61599	K.ATFMVGSYGPRPEEYEFLTPVEEAPK.G
Q61646	K.DITPTLTLYVGK.N
Q61704	R.ESPGNVQIVNGYFVHFFAPQGLPVVPK.N

Q61735	K.ISVSDLINGIASLK.M
Q61838	K.AAPLSLCALTAVDQSVLLLKPEAK.L
Q62048	K.ISEEEELDTK.L
Q62188	K.SAADLISQAR.K
Q62234	K.DGICTLLITEFSK.K
Q62388	K.MTGGLGFHEVLNNLISR.I
Q62425	K.LGPNEQYK.F
Q62426	R.QIVAGTNLFIK.V
Q62465	R.CLVLTGFGGYDK.V
Q63844	K.YIHSANVLHRDLKPSNLLINTTCDLK.I
Q63918	K.LVNMLDAVR.E
Q64105	K.SDGALVDCGTSAQK.L
Q64433	K.FLPLFDR.V
Q64669	K.NFQYPSESSLAYK.E
Q64727	K.AIPDLTAPVAAVQAAVSNLVR.V
Q64737	K.GVEITGFPEAQALGLQVFHAGTALK.D
Q68FD5	K.LLLPWLEAR.I
Q6IFX2	R.ALEEANADLEVK.I
Q6IRU2	K.IQALQQQADDAEDR.A
Q6NSW3	R.ASTDLGK.L
Q6NZQ8	-.MLGWCEAIAR.N
Q6P3A8	K.GLLLSCIEDKNPCIFFEPK.I
Q6P8J7	K.HNNCMAECLTPTIYAK.L
Q6PB66	K.DESSDNFGSFFLR.H
Q6PGF3	K.LALHVEK.S
Q6PHN9	K.LLIIGDSGVGK.S

Q6X7S9	R.EAAFDIEYLR.N
Q6ZQA6	R.LHAESPSSGVYR.L
Q71RI9	K.DSTLDAAEEIFR.A
Q76MZ3	K.IGPILDNSTLQSEVKPILEK.L
Q78IK2	K.YFNSYTLTGR.M
Q78IK4	K.IAYPLGLATLGATVCYPAQSVIIAK.I
Q78ZA7	K.EAATVEELNPK.G
Q791V5	K.VLQYYQESEKPEELGSVTVQK.E
Q7TMF3	R.IGTLVGEDK.Y
Q7TNG8	R.LHPAPEATVAATCAFPSVQAAVDSTVQILQAAVPVAR.I
Q7TNS2	R.MWPLAFGSGVGLGMAYSNCQHDFQAPYLLHGK.Y
Q7TQ48	K.EEISLLEDLNQVIENR.L
Q7TQI3	R.LELSVLYK.E
Q80SU7	V.AQIGGPAEADGIAQWTAGLVVSNQTWSVIDR.E
Q80WQ9	K.LFSTPMDAGSFGQPR.L
Q80XN0	K.AILITGCDSGFGFSLAK.H
Q80YV2	E.NGETEVDACTPAEPGWKAVLTILLAHK.R
Q8BFP9	R.AVPLAGFGYGLPISR.L
Q8BFR5	K.GEETPVIVGSALCALEQR.D
Q8BFS6	K.FFVLCGDLVHAMPGTPWR.Q
Q8BFZ3	R.VAPDEHPILLTEAPLNPK.I
Q8BG32	K.EQSILELGSLLAK.T
Q8BGC4	R.DCPVPLPGDGDLLVR.N
Q8BGH2	K.ETSYGLSFFKPQPGNFER.N
Q8BGK2	K.AAMLLGSVGDALGYGNICR.E
Q8BGQ7	K.NVGCLQEALQLATSFAQLR.L

Q8BGY7	R.YTVTLGGTSFTVK.Y
Q8BH59	K.FGLYLPK.F
Q8BH64	R.FMCAQLPNQVLESISIIDTPGILSGAK.Q
Q8BH86	K.DPLHYSIVSAPAAQK.V
Q8BH95	K.AQFGQPEILLGTIPGAGGTQR.L
Q8BIJ6	K.SLGNVINPDTIISGGK.D
Q8BK30	K.TQSVLKEPEPTDTTTYK.N
Q8BKZ9	K.STVPHAYATADCDLGAVLK.V
Q8BM89	A.MGALAGFSVLSLLTYGYLCWGQDLEEEGSLK.A
Q8BMF3	K.EMFAQDHPEVNSLEEVVR.L
Q8BMF4	C.ILAIGASEDK.L
Q8BMS1	K.ADMVIEAVFEDLGVK.H
Q8BMS4	K.ILDVGCGGGLLTEPLGR.L
Q8BP40	K.NYVEDIPFLSPVYNPQEVFIR.S
Q8BS95	K.IFMATINIVLDR.V
Q8BTM8	K.VGSAADIPINISETDLSLLTATVVPPSGR.E
Q8BVI4	K.AALDGTPGMIGYGMAK.G
Q8BVI5	R.HAQSDYLK.R
Q8BW75	K.IPEDEIWQPEPESLDVPARPITSTFLER.H
Q8BWF0	K.EVGEVLCTDPLVSK.I
Q8BWT1	I.TAHLVHELR.R
Q8BZ25	K.APGPEYDVYSFAIVIWEILTQK.K
Q8BZF8	K.TIEEYAICPDLR.I
Q8C0M9	R.GNLAYATSTGGIVNK.M
Q8C156	K.WDSETHNESVSALVDK.F
Q8C196	K.QADAVYFLPITPQFVTEVIK.A

Q8C4V4	K.MSSCPHVSPAGILCVADQCHGLR.E
Q8C5Q4	R.GLPFQANAQDIINFFAPLKPVR.I
Q8CAQ8	K.AVDEAADALLK.A
Q8CC88	K.DLLGQDVFLIGPPGPLR.R
Q8CDI6	K.LLADLHK.R
Q8CG76	K.ALQTTYGTNAPR.M
Q8CGK3	K.EVEDELGPKPQLEMVTEAATDTSK.E
Q8CGW4	K.QELGAGLDLSVGSR.R
Q8CHT0	K.FAVELEGEQPISVPPSTNHTVYR.G
Q8CI04	R.LVYRTHIYIQTDITGYKPAPGDLAYPDK.L
Q8CI51	K.DFNMPLTISSLK.D
Q8CI94	K.AAPGYHMAKMIIKLVTSIGDVVNHDPVVGDR.L
Q8JZL0	K.LEPGTPGALGGIALSGWAPIPEKPYGCEECER.R
Q8JZN5	K.GVFPFPEVSQHELSEINQFVGPLEK.F
Q8JZQ2	K.GAILTGPPGTGK.T
Q8K0D5	K.LEFSDETFGSNVPK.Q
Q8K0Z7	K.FICDASSLHQVR.K
Q8K183	K.VVPVADIITPNQFEAELLSGR.K
Q8K1M6	K.LQDVFNTVGADIIQLPQIVVVGTQSSGK.S
Q8K1Z0	K.LVQLGQAEK.R
Q8K2B3	K.ANAGEESVMNLDK.L
Q8K2C6	K.WQAQDLATPQAFAR.N
Q8K370	K.AEGLWNLFLPLETDPEK.K
Q8K3J1	K.LCEAICPAQAITIEAEPR.A
Q8K411	K.IHGFTVNQVTPVPELFLTAVK.L
Q8K480	K.SLTSLPCYQTFQR.F

Q8K4Z3	K.GNPSGIQPDLLISLTAPK.K
Q8QZR5	R.DGGIPADPNNIFLSTGASDAIVTMLK.L
Q8QZS1	K.DVTDEDLNSYFK.S
Q8QZT1	K.DGLTDVYNK.I
Q8R0F8	R.STVLSEPVLFLKPSTAYAPEGSPVLMPAYCR.N
Q8R0N6	K.AANLYASSPHSEFLDYVNAPIGK.G
Q8R0P4	R.VLQTEQAVK.E
Q8R164	K.TDFAPQLQSLNK.K
Q8R1A4	K.ISENFYFDLNSEQMK.G
Q8R1G2	K.NPTLFIFAENDTVIPLEQVSTLTQK.L
Q8R1I1	R.LYSLLFR.R
Q8R1S0	R.IHPLAGQGVNMGFGDISSLVHHLSTAAFNGK.D
Q8R2G4	K.FLNKEDDSVVLIPLSEVFQVSR.K
Q8R2Q4	R.VVLGFVPLAEIMGYSTVLR.T
Q8R404	R.KAEEVVPPAMYQFSQYVCQQTGLEMPQLPTPPK.I
Q8R4N0	K.AVCEETLK.T
Q8VCA8	R.GLLTGEQTPPAQGLGSLFQAFVER.E
Q8VCM7	K.EGFGHLSPTGTTEFWLGNEK.I
Q8VCT4	K.AVIGDHGDEIFSVFGSPFLK.D
Q8VCW8	R.GGENIYPAELEDFFLK.H
Q8VDC0	R.YWGTPIPIVHCPACGPVPVPLQDLPVILPSIASLTGR.G
Q8VDK1	K.TCAELVQEAAR.L
Q8VDN2	K.DMTSEELDDILR.Y
Q8VDQ1	K.TGNVAEQLR.E
Q8VE95	R.CEDRPVVFTHLLASDSESPR.L
Q8VED5	R.NLDLDSIIAEVK.A

Q8VEK3	K.EKPYFPIPEDCTFIQNVPLEDR.V
Q8VEM8	K.ALYSNILGEENTYLWR.T
Q8VHN7	R.SGEKPSINSVYMLTAGFR.L
Q91V12	K.ATLWYVPLSLK.N
Q91V61	R.SGDAPITVQQLGTAYVSATTGAVATALGLK.S
Q91V64	K.FSMVLPEVEAALAEIPGVR.S
Q91V92	R.SGGMSNELNNIISR.T
Q91VD9	K.DCFIVYQGHHGDVGAPMADVILPGAAYTEK.S
Q91VI7	K.LENCGITAANCK.D
Q91VM9	K.ESNVEEEVWHFLR.N
Q91VR2	K.ELIEIISGAAAL.D
Q91WD5	K.AVTNMTLNFGPQHPAAHGVLR.L
Q91WK1	R.GPVAPAFALWDGELLTHSGLEVPK.G
Q91WK5	K.QEEFGALESVK.A
Q91WS0	K.AMVNLQIQK.D
Q91X72	K.ELGSPPGISLETIDAAFSCPGSSR.L
Q91XE4	R.VAVTGGTHGNEMCGVYLAR.Y
Q91Y97	K.LDQGGAPLAGTNK.E
Q91YT0	H.TICALGDGAAWPVQGLIR.H
Q91YT2	K.GPSASASTENSNAGGPSGSSNGTGESGGQDSTFECNICLDTAK.D
Q91YY4	K.LFTVPSEALAIAVATEWDSQQDTIK.F
Q91Z53	K.LLDAAGANLR.V
Q91Z61	R.SVSLSVDGK.R
Q91ZA3	K.FLSDVYPDGFK.G
Q91ZD1	R.FDFANLAVAATQEDPPK.M
Q91ZJ0	R.NLILGTHRPAR.Y

Q91ZJ5	K.GGTLTQYEGK.L
Q920B9	K.NLGFGMGIEFR.E
Q921G7	K.AAQIGAHTLSGACLDPAAFK.E
Q921I1	F.ASCHLAQAPNHVVVSR.K
Q922B1	K.LEVDAIVNAANSSLLGGGGVDGCIHR.A
Q922R8	R.ALDLFSDNAPPPELLEIINEDIAK.K
Q922U2	K.YEELQQTAGR.H
Q923D2	K.TVAGQEAVIVLLGTGNDLSPTTVMSEGTR.N
Q924D0	K.SLTHTQAASLPYVALTAWSAINK.V
Q924M7	K.ESVGGDTEAMASALR.N
Q924X2	K.ALADDVELYCFQFLPFGK.G
Q93092	K.ALAGCDFLTISPK.L
Q99J27	K.LVEEGVPK.E
Q99J39	R.LAQGFGVDHGQVAEQSAGVLQLR.Q
Q99J99	R.DGIEPGHIPGSVNIPFTEFLTNEGLEK.S
Q99JY0	K.AQDEGHLSDIVPFKVPGK.D
Q99K24	R.ERPPFIDLETFNAGSDAGSDSEYESPFVGVGNR.S
Q99KI0	A.VPSTIHCDHLIEAQVGGEK.D
Q99KQ4	K.YLLETSGNLDGLEYK.L
Q99KR7	K.HVGPGVLSMANAGPNTNGSQFFICTIK.T
Q99L13	K.DFSSVFQYLREEEPF.-
Q99L47	K.AIDLFTDAIK.L
Q99LB2	K.NFAAELAPK.N
Q99LC3	K.EIAQQLGMK.H
Q99LC5	D.PEAPIFQVADYGIVADLFK.V
Q99LD8	R.GGGDLPNSQEALQK.L

Q99LP6	K.DLLEVADILEK.A
Q99LX0	K.DGLILTSR.G
Q99LY9	K.IEFDDFEECLLR.Y
Q99MN9	K.AYNMLDIIHAVIDER.E
Q99MR8	K.ESVCQAALGLILK.E
Q99N87	R.SGFLWPGLNVPLIK.S
Q99N96	K.IATLDMPSDQIAANLQAVINEVCK.H
Q99NB1	D.PSVITEILSAFQK.Y
Q99PT1	K.SIQEIQELDKDDESLR.K
Q9CPP6	K.KLEALLQGGEVEEVILQAEK.E
Q9CPQ1	R.VHIAGAFIVALGVAAAYKFGVAEPR.K
Q9CPQ3	R.LWGLTEMFPER.V
Q9CPQ8	K.VELVPPTPAEIPTAIQSVK.K
Q9CPU0	K.ATLELTHNWGTEDDETQSYHNGNSDPR.G
Q9CPU4	R.AHQNTLEVYPPFLFFLTVGGVYHPR.I
Q9CPV4	K.LELQGIQGAVDHAAAFGR.I
Q9CPX8	R.LILDWVPYINGK.F
Q9CPY7	K.LNLPINIIGLAPLCENMPSGK.A
Q9CQ54	K.KTYAEILEPFHPVR.-
Q9CQ60	K.LPIPDSQVLTINPALPVEDAAEDYAR.K
Q9CQ62	K.EEWDIIEGLIR.K
Q9CQ65	K.YVDTPFGKPSDALILGK.I
Q9CQ69	R.EFGNLAR.I
Q9CQ75	K.TVSLNNLSADEVTR.A
Q9CQ89	K.TQSSLVPALTEFVR.S
Q9CQ91	K.ATPYNYPVPVRDDGNMPDVPSHPQDPLGPSLDWLK.N

Q9CQ92	R.GIVLLEELLPK.G
Q9CQA3	K.CGPMVLDALIK.I
Q9CQC7	K.YKPAPLATLPSTLDPAEYDVSPETR.R
Q9CQH3	K.TLAILQIESEKAELR.L
Q9CQI6	R.AAYNLVR.D
Q9CQJ8	K.QLQEETSPDGIMTEALPPAR.R
Q9CQM9	K.YEISSVPTFLFFK.N
Q9CQN1	K.AFLEALQNQAETSSK.I
Q9CQN6	K.AGSVPSLAAGLFFGGLAGLGAYQLSQDPR.N
Q9CQQ7	K.AQQALVQK.R
Q9CQR4	K.IGEEIVITAHILK.Q
Q9CQV8	K.TAFDEAIAELDTLNEESYK.D
Q9CQX8	K.GSTSPDLLMHQGPPDTAEIIK.S
Q9CQZ5	R.EVPNTVHLMQLDITVK.Q
Q9CQZ6	K.IEGTPLETVQK.K
Q9CR21	K.LMCPQEIVDYIADKK.D
Q9CR61	K.HEQHDWDYCEHLDYVK.R
Q9CR62	K.ALIGMTAGATGAFVGTPAEVALIR.M
Q9CR68	K.EIDQEAAVEVSQLRDPQHDLDR.V
Q9CRB8	K.WTTTTLGLLAIPVIIHPIDR.S
Q9CRB9	R.LSENVIDR.M
Q9CW03	K.AEEELGELEAK.L
Q9CW46	R.CFLVYSER.T
Q9CWJ9	R.DVSELTGFPEMLGGR.V
Q9CWL2	K.AHFHCVVEECGALFSTLDGAIKHANFHFR.T
Q9CWR0	R.DPLGPGSTK.T

Q9CXJ4	K.QPTVLILDEATSALDAESER.V
Q9CXV1	K.AASLHWTSER.V
Q9CXZ1	K.LDITTLTGVPEEHIK.T
Q9CYR0	K.NPVTIFSLATNEMWR.S
Q9CZ13	G.TATFAQALQSVPETQVSILDNGLR.V
Q9CZ30	K.IGIVGLPNVGK.S
Q9CZ42	K.DMENLFQLVR.N
Q9CZ44	K.TGFSLDNGDLR.S
Q9CZB0	K.SLCLGPTLIYSAK.F
Q9CZR8	K.VPSGFYVGSYVHGVTQSPSLQNLVLGK.Y
Q9CZS1	K.EEIFGPVQPLFK.F
Q9CZU6	H.ASASSTNLK.D
Q9D020	K.DNSNIILLGDSQGDLR.M
Q9D023	K.WGLVCAGLADMAR.P
Q9D051	H.CLEAAAVLSK.E
Q9D0F9	K.DLEALMLDR.S
Q9D0K2	K.CTLPLTGK.Q
Q9D0M3	A.SDLELHPPSYPWSHR.G
Q9D0S9	R.DVAPQAPVHFLVIPR.K
Q9D172	K.ITSLAQLNAANHDAAIFPGGFGAAK.N
Q9D1A2	K.TVFGVEPDLTR.E
Q9D1G1	K.EFADSLGVPFLETSAK.N
Q9D1G3	K.TALPAAELGLYSLVLSGALAYAGR.G
Q9D1H9	R.ADGEYWLGLQNLHLLTLK.Q
Q9D1I5	K.MELLHPLGSDSPITGFLQK.N
Q9D1L0	K.LCEGFNEVLR.Q

Q9D1M0	R.DVAWAPSIGLPTSTIASCSQDGR.V
Q9D2G2	K.ASAFALQEQPVVNAVIDDATK.E
Q9D2J7	R.AAELGHELSMEILAKAKADMTIVDNEGK.G
Q9D3D9	K.AQSELSGAADEAAR.A
Q9D6J5	K.QYPYNNLYLER.G
Q9D6J6	K.DIEEIIDELK.A
Q9D6R2	F.AGGVQTVTLIPGDGIGPEISASVMK.I
Q9D6X6	K.LSTGCTGTLVAEKHVLTAAHCIHDGK.T
Q9D6Y7	R.EGQVFYYAEDYHQQYLSK.N
Q9D6Y9	R.LEAALADVPELAR.L
Q9D7B6	K.AAQLGFGGVYVR.T
Q9D7J4	R.ESILYGSLGSIVTGLGHFLVTSR.I
Q9D855	K.YEEDKFYLEPYLK.E
Q9D892	K.KLEEVIQILGDNFPCTLEAQK.I
Q9D8B4	K.LEGWELFPTPK.V
Q9D8S4	K.ESTVTLQQAEYEFLSFVR.Q
Q9D8T7	K.ALHGAQTSDEER.F
Q9D8U6	K.AELSNVSDTVWNIR.E
Q9D8W5	K.DPNNLLNDWSQKLNSLMSLVNKTTHLIAK.E
Q9D8Y0	R.EFLLIFR.K
Q9D967	R.DGMSLQTLTQGLETFAK.A
Q9DAK9	R.NGYDCECLGGGR.I
Q9DB20	K.FSPLTANLMNLLAENGR.L
Q9DB29	K.VSSLPWLLPYWK.D
Q9DB77	I.IENLHDVAYK.N
Q9DBB8	K.ESPVVPLAESELLAEILEEAR.K

Q9DBG5	K.TLTTAAVSTAQPILSK.L
Q9DBJ1	K.NLKPIKPMQFLGDEETVR.K
Q9DBL1	K.FAQEHVAPLVSSMDENSK.M
Q9DBP5	R.IQTYLESTKPIIDLYEEMGK.V
Q9DC61	K.GLDTVVDLLADVVLHPR.L
Q9DC69	K.AVQHSNVVINLIGR.E
Q9DC70	G.PSPSPSPSLSSTQSAVSK.A
Q9DCB8	R.VVVDSDSLAFVK.G
Q9DCD0	K.GILFVGSGVSGGEEGAR.Y
Q9DCJ5	K.FDQCVLDK.L
Q9DCM0	L.SQQSASGAPVLLR.Q
Q9DCM2	K.LIENTDAACK.Y
Q9DCS3	K.LPAVGGNEGVGQVIAVGSSVSALKPGDWVIPANAGLGTWR.T
Q9DCS9	K.AYDLVVDWPVTLVR.E
Q9DCT2	K.DFPLTGYVELR.Y
Q9DCV4	K.VLFANPPSSTYEEALR.Y
Q9DCW4	K.AGDLGVDLTSK.V
Q9DCX2	K.IPVPEDKYTALVDQEEKEDVK.S
Q9DCZ1	K.GDVENTILDILGGLR.S
Q9DCZ4	K.IDELSLYSVPEGQSK.Y
Q9EPB5	K.ALQGYYDVR.R
Q9EPK5	R.QEAALCR.Q
Q9EQ20	K.DMDLYSYR.L
Q9EQI8	K.ALTPLQEEMAGLLQQIEVER.S
Q9EQP2	K.SISIIDSPGILSGEK.Q
Q9ERI6	R.QELCQAGGAGPDGTDGQLVVK.E

Q9ERS2	R.ENLEEEAIIMK.D
Q9ESN3	R.LELQGCVSSVSPGCPVR.V
Q9JHI5	A.HSILPVDDDINGLNEEQK.Q
Q9JHW2	K.ASYVAWGHSTVVDPWGQVLTK.A
Q9JI75	K.ALTSDIFEEQR.K
Q9JI91	K.ALDYIASK.G
Q9JII6	K.MPLIGLGTWK.S
Q9JJ26	R.GPESLDSQTKPWTR.S
Q9JJZ2	R.AVMVDLEPTVVDEVR.A
Q9JK42	K.QFLDFGSSNACEK.T
Q9JKB1	K.FLENYDAIR.V
Q9JKF7	R.IGDFIDVSEGPLIPR.T
Q9JKS4	K.ASGAGLLGGSLPVK.D
Q9JLT4	K.IIVDAQEATSVPHIYAIGDVAEGRPELTPTAIK.A
Q9JLZ3	K.AVGLISHVLEQNQEGDAAYR.K
Q9JMH6	K.VLVLDFVTPTPLGTR.W
Q9QUH0	K.TQEILSQLPFK.Q
Q9QUM9	K.AINQGGLTSVAVR.G
Q9QVP4	K.ETYSQLGR.V
Q9QWK4	K.LVGGDTPCSGR.L
Q9QXW2	K.QILPHQMTTAGPVLGEGR.G
Q9QXX4	K.ASFANEDGQVSPGSLLLAGAIAGMPAASLVTPADVIK.T
Q9QYG0	K.CPVMLVVGDQAPHEDAVVECNSK.L
Q9QYR9	F.LVELEVLDGHEPDGGQR.L
Q9QZB1	R.GSNACCFCWCCCCTCSCLTVR.N
Q9R062	K.GALVLGSSLK.Q

Q9R069	R.GEQVALDCTPR.E
Q9R0H2	K.LLTVKTISHESGEHSAQGK.T
Q9R0P3	K.AFSGYLGPDESK.W
Q9R0P5	K.CIVVEEGKEILVGDVGATITDPFK.H
Q9R0X4	K.MSPLSIVTVLVDKIDMCK.H
Q9R0Y5	K.GELVPLDTVLDMLR.D
Q9R111	K.FLYLGDDR.N
Q9R1P0	R.YLLQYQEPIPCEQLVTALCDIK.Q
Q9R1P1	R.FGPYYTEPVIAGLDPK.T
Q9R229	K.ITIFEVLESADGSEEER.S
Q9R244	R.NQSEVTAVLNDLGEDSETEPEAEGLGQAFEEGIPNLAR.L
Q9WTP6	K.DLVMFI.-
Q9WTP7	K.HLSSGDLLR.Q
Q9WTR5	K.DIQGSLQDIFK.F
Q9WUB3	K.AAPGYHMAKMIIKLITAIGDVVNHDPAVGDR.L
Q9WUM5	K.EKISALQSAGVVVSMSPAQLGTTIYK.E
Q9WUR2	K.AAEMLLFGK.K
Q9WUU7	R.VGDYGSLSGR.E
Q9WUZ7	K.EENIIYSFLGLAPPPGSK.V
Q9WV35	K.ELIDLPPFEIVTGVR.L
Q9WV98	K.LTETCFLDCVK.D
Q9WVA4	K.QMEQISQFLQAAER.Y
Q9WVH9	R.DQPFTILYR.D
Q9WVK4	R.FMCAQLPNPVLDSISIIDTPGILSGEK.Q
Q9WVL0	K.GIDYEIVPINLIK.D
Q9Z0S1	K.LTDIHGNALQYNK.E

Q9Z0X1	K.CLIATGGTPR.S
Q9Z126	K.HITSLEVIK.A
Q9Z1E4	K.VGGIYTVLQTK.A
Q9Z1J3	R.VLDAMLPYLVNYYGNPHSR.T
Q9Z1P6	K.LSNNYYCTR.D
Q9Z2I0	K.LFEDELTLDNLTRPQLVALCK.L
Q9Z2I8	K.SSGLPITSAVDLEDAAK.K
Q9Z2I9	K.ALIADSGLK.I
Q9Z2K1	K.ASLENSLEETK.G
Q9Z2U1	R.AIGSASEGAQSSLQEVYHK.S
Q9Z2U2	K.LINEDSTNAENQGNTTLK.G
Q9Z2W0	K.GFFELFPSVSR.N
Q9Z2Y8	K.HGLLPSETIAVVEHIK.A
Q9Z2Z6	K.NLFTPEGK.S
REFSEQ:N	K.TITFSMLVFCSVWISFVFTYLR.S
REFSEQ:X	K.VIHDNFGIVEGLMTTVHAITVTQK.T
REFSEQ:X	K.CYAHLYPR.A
REFSEQ:X	K.RADAAPTVSIFPPSSEQLTSGGASVVCFLNNFYPK.D
REFSEQ:X	K.QASEGTLK.G
REFSEQ:X	R.NNYTASSTSKPSVLTFFQGSR.V
REFSEQ:X	K.AAGVSIEPFWPGLFAK.A
REFSEQ:X	K.IIHDNFGIVEGLMTTVHAITATQK.T
REFSEQ:X	R.IMIDLGTGNNINR.A
REFSEQ:X	K.QLSAFAEYVAEILPK.Y
REFSEQ:X	K.LIASVAEDEAAVPNNK.I
SWISS-PR	R.NYLEQPAILE.-

SWISS-PR	K.ECPPCAAPDLLGGPSVFIFPPK.I
SWISS-PR	K.CLAAALIVLTESGR.S
SWISS-PR	K.TIDDLEETLASAK.E
SWISS-PR	R.WMNVGHEDDELKPYVEPLLTTGPR.D
SWISS-PR	K.TSPGPVSLNVGGHMSYLK.G
SWISS-PR	K.GLQYLNLGNGCHKLIYLDLSGCTQISVQGFR.N
TREMBL:A	K.ADLDVSGPK.V
TREMBL:A	K.GGIVGMTLPIAR.D
TREMBL:A	K.ISALEASLK.L
TREMBL:A	R.STGVALSIAVGLSWWPR.L
TREMBL:A	K.GHYTEGAELIENVMDVVR.R
TREMBL:A	K.VDLGVLGK.Q
TREMBL:A	Q.ADGPDMQSLFTQYFQSMTDYGK.D
TREMBL:A	F.TPAAQAAFQKVVAGVAAALAHKYH.-
TREMBL:B	K.YVNEFLAPALCTQK.V
TREMBL:B	D.QIIQANPLLEAFGNAK.T
TREMBL:B	K.IIYGDPATFLPHLPQK.S
TREMBL:B	R.AVIGILQMICK.T
TREMBL:B	K.DLEQIFCQFDPK.L
TREMBL:B	A.EEGLNFPTYDGK.D
TREMBL:O	K.NVCYSPASISSALAMVLLGAK.G
TREMBL:O	R.LGMTDAFEEGMADFSGIASK.E
TREMBL:O	K.AVLEEAEFQR.K
TREMBL:O	R.DLYAFYPLLIR.F
TREMBL:Q	K.GLIDEANQDFTNR.I
TREMBL:Q	R.LAQAVHER.H

TREMBL:Q	R.FRQDSNEAVGGFFSQIGQLYMVHHLWAYK.D
TREMBL:Q	K.YAVGSACIGGGQGIALIIQNTA.-
TREMBL:Q	K.QQVSEAALTLAGLNK.R
TREMBL:Q	R.AEAPSPGVPPEQSQPYAVLR.R
TREMBL:Q	R.GLLSSAEGEPAVPVLSNHRPPQPLK.G
TREMBL:Q	R.FTVAELK.Q
TREMBL:Q	K.LYFLDMVTEDAK.T
TREMBL:Q	R.TAIEAMAAVFGGTQSLHTNSFDEALGLPTVK.S
TREMBL:Q	K.CISVPEDVYEQGCVKDVDEGLQAAEK.I
TREMBL:Q	K.ELFLGNIEQK.G
TREMBL:Q	K.GSWPISTKEGLPIQGWLGTKVR.T
TREMBL:Q	R.LIDMLSEAGLPVIEATSFVSPK.W
TREMBL:Q	K.SSVHSLELCK.Q
TREMBL:Q	R.SVAGALAQTLAAAAASVSTPGPSSATPSQPPATCSSRFR.V
TREMBL:Q	R.LQKEFSPFGTITSTKVMTEGGR.S
TREMBL:Q	K.TAAVSSVLFCFVLFFSVGNLKLTVSGGEHS.-
TREMBL:Q	K.GEDLSLTCQTYTSMFLNFLCEVFSSETCEDHLNEEF.Q
TREMBL:Q	K.NNIDKLEGDHQLIQEALVFDNK.H
TREMBL:Q	K.HLSLHITATQPGDSAIYFCSAS.-
TREMBL:Q	R.NASLAISNLR.V
TREMBL:Q	R.AEDIPSLK.L
TREMBL:Q	K.LAASEAATAISHQAIQILGGMGYVTEMPAER.Y
TREMBL:Q	S.CGCCHCCCHCCCLHSRPSYR.K
TREMBL:Q	K.GQMEAIPCVVGDEEVWTSDIQYQLSPFNHAHK.V
TREMBL:Q	-.MGLVTCSHSQQAK.G
TREMBL:Q	R.QAGVLAAAALVGLAEAEEVLPR.D

TREMBL:Q	K.SLCPMPPR.K
TREMBL:Q	-.MPGETHPAAPGPADLAR.C
TREMBL:Q	K.LMSGPVIISNFSAAMVHTIVNETLESMTSFK.A
TREMBL:Q	K.MAVNFLSGK.K
TREMBL:Q	K.YLHLYEQNQLLRR.E
TREMBL:Q	K.NYIWNTLNSGR.V
TREMBL:Q	R.NLGVTVSQYDNPSFNLLR.L
TREMBL:Q	R.GPLVSTVMAFSAGCLSLGAHCR.T
TREMBL:Q	K.GCGCYSGEVTGAAVGVLGSFFPSPSQASR.F
TREMBL:Q	R.WSGALESSSLQHPCWLWLLPSWR.L
TREMBL:Q	K.CLGPFDEWESR.L
TREMBL:Q	R.FAQFFLCPLFDASCK.D
TREMBL:Q	R.QATTIIADNIIFLSDQTK.E
TREMBL:Q	K.AEEQEPELTSTPNFVVEVTK.T
TREMBL:Q	H.VPMYLFLAMLASSDLGLSLFTFPTLLR.I
TREMBL:Q	K.EHHLSEVQNMASEEKLEQVLSSMK.E
TREMBL:Q	K.GIVDSEDLPLNISR.E
TREMBL:Q	A.DALANAAGHLDDLPGALSALSDLHAHK.L
TREMBL:Q	K.FSLIMELSTQLSLQTEKITQLEDTLAEK.E
TREMBL:Q	K.TCLDPNAPGVK.R
TREMBL:Q	K.DLYANTVLSGGTTMYPGLADR.M
VEGA:OTT	K.TIAECLADELINAAK.-
VEGA:OTT	R.LVLGDNSLAIR.E
VEGA:OTT	K.FGIEAFSDCLR.Y
VEGA:OTT	K.EGALGCGGLPVLDGSR.G
VEGA:OTT	P.WLSPANYIDNVGNLHFLYSELALVPK.C

VEGA:OTT	R.LILTEGEYQYSNGSLSSGSWAASQK.E
ENSEMBL:	R.APNSGSPASKAAAGTDGLSHR.C
ENSEMBL:	K.ESLSAKTTSQSPDAQASCGHPQTDSVAGPAMATTK.E
ENSEMBL:	K.QMVDTLVESSTNVEMILK.F
ENSEMBL:	K.TPFCVLVSCGHPGSPPHAQMSGDSYIVGAVVR.Y
ENSEMBL:	R.YCLVWHQWKPGQTPTPLITWTSDR.Y
ENSEMBL:	R.LKAEEVAFWTELLAK.N
ENSEMBL:	K.LLIYSTSNLASGVPSR.F
ENSEMBL:	K.LLIYSASNR.Y
ENSEMBL:	K.DAPVSAGLGMYSCPVYMTGPFGTTK.L
ENSEMBL:	R.WSPECASDRVNPAPGNQASQLEAQQKT.-

STAB 4. Top pathway abundance ratio (for heat map)

IPA	Protein	Abundance (Log2 fold change)		
		YCL/OCL	OCR/OCL	ORP/OCL
Mitochondrial Dysfunction	Ogdh	-0.000965	0.035886	-0.157433667
Mitochondrial Dysfunction	Pdha1	-0.21074	-0.101204	-0.16891713
Mitochondrial Dysfunction	Sod2	-0.100227	0.089156	0.266235408
Mitochondrial Dysfunction	Park7	-0.214784	-0.157663	0.15271864
Mitochondrial Dysfunction	Atp5o	0.480993	0.569	0.492953955
Mitochondrial Dysfunction	Uqcr10	0.474024	0.552438	0.368472695
Mitochondrial Dysfunction	Uqcr11	0.402477	0.408582	0.524319651
Mitochondrial Dysfunction	Atp5i	0.321549	0.53549	0.353544166
Mitochondrial Dysfunction	Cox5b	0.377639	0.461625	0.401590114
Mitochondrial Dysfunction	Atp5e	0.392801	0.43687	0.405630937
Mitochondrial Dysfunction	Atp5a1	0.213801	0.227092	0.21592083
Mitochondrial Dysfunction	Sdhb	0.224637	0.230155	0.185434314
Mitochondrial Dysfunction	Uqcrc1	0.210068	0.157776	0.185842717
Mitochondrial Dysfunction	Atp5b	0.186923	0.206204	0.19663429
Mitochondrial Dysfunction	Mtco2	0.188326	0.179301	0.193178831
Mitochondrial Dysfunction	Uqcrc2	0.168657	0.111558	0.264069308
Mitochondrial Dysfunction	Cpt1b	0.203319	0.021848	0.261590743
Mitochondrial Dysfunction	Ndufa8	0.031292	0.422353	0.300744656
Mitochondrial Dysfunction	Ndufv1	0.168862	0.33105	0.217833337
Mitochondrial Dysfunction	Ndufv2	0.205978	0.313038	0.236092135
Mitochondrial Dysfunction	Ndufs2	0.135442	0.273342	0.31185622
Mitochondrial Dysfunction	Uqcrh	0.124437	0.266781	0.2893686
Mitochondrial Dysfunction	Ndufa13	0.182908	0.327228	0.325090127
Mitochondrial Dysfunction	Sdha	0.145255	0.305407	0.289073359
Mitochondrial Dysfunction	Ndufb9	0.142368	0.33379	0.266676018
Mitochondrial Dysfunction	Ndufs6	0.279601	0.387567	0.308401194
Mitochondrial Dysfunction	Ndufb6	0.231439	0.431226	0.381817684
Mitochondrial Dysfunction	Ndufb3	0.266479	0.379267	0.404503913
Mitochondrial Dysfunction	Atp5l	0.229172	0.376773	0.36152389
Mitochondrial Dysfunction	Ndufa9	0.464427	0.330988	0.294342764
Mitochondrial Dysfunction	Uqcrb	0.271407	0.22488	0.332542405
Mitochondrial Dysfunction	Ndufb10	0.210063	0.270209	0.27947102
Mitochondrial Dysfunction	Ndufs1	0.210394	0.275165	0.328238393
Mitochondrial Dysfunction	Cyc1	0.350122	0.300756	0.328012793
Mitochondrial Dysfunction	Uqcrq	0.311224	0.281548	0.351379341
Mitochondrial Dysfunction	Atp5c1	0.270075	0.322741	0.256517177
Mitochondrial Dysfunction	Cox4i1	0.27907	0.280764	0.301717023
Mitochondrial Dysfunction	Ndufa6	0.295384	0.31732	0.299289844
Glycolysis/gluconeogenesis	Pgm5	1.035416	-0.524806	0.021813422
Glycolysis/gluconeogenesis	Acsl1	0.273623	0.269125	0.366292588
Glycolysis/gluconeogenesis	Dhrs4	0.21773	0.038569	0.307841156
Glycolysis/gluconeogenesis	Eno3	0.158049	-0.116109	-0.140510973
Glycolysis/gluconeogenesis	Bpgm	0.570948	-0.048186	-0.027405624
Glycolysis/gluconeogenesis	Pygb	-0.352957	-0.014715	0.581662089
Glycolysis/gluconeogenesis	Gpi	-0.201589	0.035211	0.041987634
Glycolysis/gluconeogenesis	Eno1	-0.127023	0.187823	0.155452083
Glycolysis/gluconeogenesis	Mb	-0.008748	-0.084445	0.09304761
Glycolysis/gluconeogenesis	Pygm	-0.16411	-0.030432	0.24452494

Glycolysis/gluconeogenesis	Pfkm	-0.079776	-0.368156	0.064485843
Glycolysis/gluconeogenesis	Acss1	-0.134249	-0.196204	0.035701843
Glycolysis/gluconeogenesis	Pgk1	-0.232368	-0.120494	-0.094647592
Glycolysis/gluconeogenesis	Pkm	-0.247381	-0.169361	-0.02249623
Glycolysis/gluconeogenesis	Mtap	-0.482266	-0.284363	-0.129947768
Glycolysis/gluconeogenesis	Aldh2	-0.310946	0.040344	-0.250233734
Glycolysis/gluconeogenesis	Aldoa	-0.468075	-0.044432	-0.106977368
Glycolysis/gluconeogenesis	Eno1	-0.35061	-0.069651	0.000644293
Glycolysis/gluconeogenesis	Gapdh	-0.352156	-0.166409	-0.138009041
Glycolysis/gluconeogenesis	Tpi1	-0.32883	-0.086644	-0.092855093
Glycolysis/gluconeogenesis	Aldh1b1	-0.727728	-0.001992	-0.570623245
Glycolysis/gluconeogenesis	Pgam1	-0.693374	-0.600774	-0.241664647
Actin cytoskeleton	Mapk3	2.373329	0	0.963950101
Actin cytoskeleton	Actn2	0.497596	0.082017	0.111240327
Actin cytoskeleton	Myl3	0.715079	0.560849	0.0548398
Actin cytoskeleton	Myl2	0.685855	0.320154	0.022354779
Actin cytoskeleton	Ppp2ca	0.100295	-0.13984	-0.601173549
Actin cytoskeleton	Actn2	0.581329	-0.2362	-0.544488023
Actin cytoskeleton	Ttn	0.621704	-0.070704	-0.401510637
Actin cytoskeleton	Myh6	0.745142	-0.166184	-0.271142875
Actin cytoskeleton	Myl4	-1.713641	-0.668352	-0.845445273
Actin cytoskeleton	Myl7	-1.790238	-1.027924	-1.650483784
Actin cytoskeleton	Kng1	-0.762141	0.024264	0.156682842
Actin cytoskeleton	Tln1	-0.873953	0.054451	-0.470042231
Actin cytoskeleton	Mb	-0.008748	-0.084445	0.09304761
Actin cytoskeleton	Cfl2	-0.300081	-0.336488	0.119543063
Actin cytoskeleton	Msn	-0.43034	-0.425794	-0.164268811
Actin cytoskeleton	Cfl1	-0.538903	-0.465135	-0.244151927
Actin cytoskeleton	Vcl	-0.229282	-0.12373	-0.255267902
Actin cytoskeleton	Pfn1	-0.349733	-0.245705	-0.263609822
Fatty Acid oxidation	Mb	-0.008748	-0.084445	0.09304761
Fatty Acid oxidation	Ivd	-0.208426	-0.131013	-0.166520495
Fatty Acid oxidation	Eci2	0.259647	0.721991	0.224026456
Fatty Acid oxidation	Acsl1	0.273623	0.269125	0.366292588
Fatty Acid oxidation	Hadha	0.202355	0.069942	0.302563045
Fatty Acid oxidation	Hadhb	0.176379	0.161758	0.282148709
Fatty Acid oxidation	Acadm	0.040919	0.330539	0.397558304
Fatty Acid oxidation	Acaa2	0.185536	0.273963	0.144996716
Fatty Acid oxidation	Echs1	0.07122	0.324791	0.205455169
TCA Cycle	Ogdh	-0.000965	0.035886	-0.157433667
TCA Cycle	Mb	-0.008748	-0.084445	0.09304761
TCA Cycle	Idh3b	-0.058201	0.114845	0.25312374
TCA Cycle	Aco2	-0.024403	0.119703	0.089590605
TCA Cycle	Sucla2	0.117741	0.13328	0.112611883
TCA Cycle	Ogdhl	-0.211138	0.739423	0.001485204
TCA Cycle	Sdhc	0.243663	0.510743	0.288589181
TCA Cycle	Sdha	0.145255	0.305407	0.289073359
TCA Cycle	Sdhb	0.224637	0.230155	0.185434314
LXR/RXR activation	Hpx	-1.341048	-0.685755	0.071662308
LXR/RXR activation	A1bg	-1.199389	-0.553263	-0.123199988
LXR/RXR activation	Clu	-1.382827	-0.662506	-0.192550776

LXR/RXR activation	Kng1	-0.762141	0.024264	0.156682842
LXR/RXR activation	C1qbp	-0.128262	-0.023541	0.202588801
LXR/RXR activation	C1qbp	-0.141777	-0.154411	0.131106357
LXR/RXR activation	Mb	-0.008748	-0.084445	0.09304761
LXR/RXR activation	Etfa	-0.046758	-0.173687	0.09499863
LXR/RXR activation	Fga	-0.399518	-0.332056	-0.116593958
LXR/RXR activation	Apoa1bp	-0.296635	-0.214541	0.114630357
LXR/RXR activation	Alb	-0.212788	-0.335317	-0.329075547
LXR/RXR activation	Serpina1d	-0.067269	-0.427691	-0.04481209
LXR/RXR activation	Apoa2	1.23885	1.062424	0.4471989
LXR/RXR activation	Zc3hc1	0.16981	-0.17641	-0.716883725
LXR/RXR activation	Apoa2	0.530281	0.050637	0.07501192
LXR/RXR activation	Echs1	0.07122	0.324791	0.205455169
LXR/RXR activation	Vtn	0.17458	0.692635	-0.167188967
BCAA metabolism	Mccc1	0.412199	0.456505	0.278685802
BCAA metabolism	Hadha	0.202355	0.069942	0.302563045
BCAA metabolism	Hadhb	0.176379	0.161758	0.282148709
BCAA metabolism	Acadm	0.040919	0.330539	0.397558304
BCAA metabolism	Echs1	0.07122	0.324791	0.205455169
BCAA metabolism	Mb	-0.008748	-0.084445	0.09304761
BCAA metabolism	Ivd	-0.208426	-0.131013	-0.166520495
BCAA metabolism	Hibadh	-0.314035	0.180747	-0.123335415
BCAA metabolism	Bckdhb	0.000972	0.362844	-0.175886253
Ketogenesis & Ketolysis	Hadha	0.202355	0.069942	0.302563045
Ketogenesis & Ketolysis	Hadhb	0.176379	0.161758	0.282148709
Ketogenesis & Ketolysis	Bdh1	-0.593482	0.111308	0.067069456
Ketogenesis & Ketolysis	Mb	-0.008748	-0.084445	0.09304761
Ketogenesis & Ketolysis	Oxct1	-0.237152	-0.214718	-0.203880388
Calcium signaling	Myl4	-1.713641	-0.668352	-0.845445273
Calcium signaling	Myl7	-1.790238	-1.027924	-1.650483784
Calcium signaling	Mapk3	2.373329	0	0.963950101
Calcium signaling	Casq2	-0.23127	-0.360763	-0.148116363
Calcium signaling	Calr	-0.547556	-0.55763	-0.141834295
Calcium signaling	Tpm4	-0.68719	-0.562485	-0.272305042
Calcium signaling	Tnnt2	0.215868	-0.108437	0.03165674
Calcium signaling	Tpm1	0.271954	-0.028298	0.013710201
Calcium signaling	Tnni3	0.244105	-0.043138	0.038081398
Calcium signaling	Mb	-0.008748	-0.084445	0.09304761
Calcium signaling	Casq2	0.227639	0.182243	0.129315704
Calcium signaling	Myh6	0.745142	-0.166184	-0.271142875
Calcium signaling	Myl3	0.715079	0.560849	0.0548398
Calcium signaling	Myl2	0.685855	0.320154	0.022354779
Cell cycle	Mb	-0.008748	-0.084445	0.09304761
Cell cycle	Ywhag	-0.079003	-0.459227	0.048792735
Cell cycle	Ywhaq	-0.518929	-0.915889	-0.332474095
Cell cycle	Ywhaz	-0.328403	-0.386041	-0.169402676
Cell cycle	Ywhah	-0.488697	-0.494146	-0.20704702
Cell cycle	Ywhab	-0.398356	-0.603659	-0.202835352
PAK signaling	Myl4	-1.713641	-0.668352	-0.845445273
PAK signaling	Myl7	-1.790238	-1.027924	-1.650483784
PAK signaling	Mapk3	2.373329	0	0.963950101

PAK signaling	Myl3	0.715079	0.560849	0.0548398
PAK signaling	Myl2	0.685855	0.320154	0.022354779
PAK signaling	Cfl1	-0.538903	-0.465135	-0.244151927
PAK signaling	Mb	-0.008748	-0.084445	0.09304761
PAK signaling	Cfl2	-0.300081	-0.336488	0.119543063
Oxidative stress response	Mapk3	2.373329	0	0.963950101
Oxidative stress response	Gsta4	-0.386608	0.391821	-0.03557949
Oxidative stress response	Gstm1	-0.462974	0.014298	0.211660682
Oxidative stress response	Gstm5	-0.476304	-0.060345	-0.04287232
Oxidative stress response	Prdx5	-0.019873	0.02035	0.012034977
Oxidative stress response	Mb	-0.008748	-0.084445	0.09304761
Oxidative stress response	Akr7a2	0.020396	-0.174921	0.127142907
Oxidative stress response	Sod2	-0.100227	0.089156	0.266235408
Oxidative stress response	Fth1	-0.274939	-0.14123	0.388844
Oxidative stress response	Stip1	-0.12109	-0.463919	-0.239305416
Oxidative stress response	Prdx1	-0.188161	-0.316965	-0.026354856
Oxidative stress response	Cbr1	-0.185834	-0.309991	-0.021404883
Oxidative stress response	Txn	-0.289353	-0.43563	-0.039583797
Oxidative stress response	Vcp	-0.231129	-0.381307	-0.139776704
Oxidative stress response	Gpx3	-0.59594	-0.231848	-0.621961648
Oxidative stress response	Ftl1	-0.935809	-0.526766	0.12995364
14-3-3 signaling	Mapk3	2.373329	0	0.963950101
14-3-3 signaling	Mb	-0.008748	-0.084445	0.09304761
14-3-3 signaling	Ywhag	-0.079003	-0.459227	0.048792735
14-3-3 signaling	Tuba4a	-0.085798	-0.313735	-0.101636122
14-3-3 signaling	Ywhaq	-0.518929	-0.915889	-0.332474095
14-3-3 signaling	Vim	-0.727681	-0.9286	-0.389865293
14-3-3 signaling	Pdia3	-0.485881	-0.601326	-0.519077221
14-3-3 signaling	Ywhaz	-0.328403	-0.386041	-0.169402676
14-3-3 signaling	Ywhah	-0.488697	-0.494146	-0.20704702
14-3-3 signaling	Ywhab	-0.398356	-0.603659	-0.202835352
Myc mediated apoptosis	Mb	-0.008748	-0.084445	0.09304761
Myc mediated apoptosis	Ywhag	-0.079003	-0.459227	0.048792735
Myc mediated apoptosis	Ywhaq	-0.518929	-0.915889	-0.332474095
Myc mediated apoptosis	Ywhaz	-0.328403	-0.386041	-0.169402676
Myc mediated apoptosis	Ywhah	-0.488697	-0.494146	-0.20704702
Myc mediated apoptosis	Ywhab	-0.398356	-0.603659	-0.202835352
Acute phase response	Apoa2	1.23885	1.062424	0.4471989
Acute phase response	Mapk3	2.373329	0	0.963950101
Acute phase response	Hpx	-1.341048	-0.685755	0.071662308
Acute phase response	Fgg	-0.728329	-0.370353	-0.169198194
Acute phase response	Ftl1	-0.935809	-0.526766	0.12995364
Acute phase response	Apoa2	0.530281	0.050637	0.07501192
Acute phase response	Mb	-0.008748	-0.084445	0.09304761
Acute phase response	Etfa	-0.046758	-0.173687	0.09499863
Acute phase response	Sod2	-0.100227	0.089156	0.266235408
Acute phase response	Cpt1b	0.203319	0.021848	0.261590743
Acute phase response	Zc3hc1	0.16981	-0.17641	-0.716883725
Acute phase response	Fga	-0.399518	-0.332056	-0.116593958
Acute phase response	Apoa1bp	-0.296635	-0.214541	0.114630357
Acute phase response	Alb	-0.212788	-0.335317	-0.329075547

Acute phase response	Serpina1d	-0.067269	-0.427691	-0.04481209

STAB 5. Top pathway half life ratios (for heat map)

IPA	Protein	Half-life ratio		
		YCL/OCL	OCR/OCL	ORP/OCL
Mitochondrial dysfunction & ETC	Uqcrb	0.03921443	1.64762873	0.442134477
Mitochondrial dysfunction & ETC	Cycs	-0.410411603	2.17943328	0.599455841
Mitochondrial dysfunction & ETC	Ndufab1	1.635956339	1.97232851	0.215608602
Mitochondrial dysfunction & ETC	Sdhd	0.375112814	1.81967524	-0.788301437
Mitochondrial dysfunction & ETC	Sod2	1.660186104	2.03542452	1.967785463
Mitochondrial dysfunction & ETC	Ndufa9	0.261786731	1.15694324	0.354616105
Mitochondrial dysfunction & ETC	Xdh	0.585773031	1.11603659	0.513162596
Mitochondrial dysfunction & ETC	Ndufs1	0.409788088	1.2484995	0.608257627
Mitochondrial dysfunction & ETC	Ndufb8	0.963078018	1.67651041	1.188299471
Mitochondrial dysfunction & ETC	Ndufa10	-0.004931458	0.92141828	1.166800525
Mitochondrial dysfunction & ETC	Txnrd2	0.508721814	1.17326337	0.952482651
Mitochondrial dysfunction & ETC	Ndufa7	0.500380734	1.07417982	0.919557712
Mitochondrial dysfunction & ETC	Ndufv2	0.671634678	1.31891082	1.209986777
Mitochondrial dysfunction & ETC	Aifm1	0.691341351	0.9335315	1.262868537
Mitochondrial dysfunction & ETC	Cox7a1	0.120647702	-0.23457557	0.160245965
Mitochondrial dysfunction & ETC	Atp5b	-0.352494794	0.08034944	0.156869151
Mitochondrial dysfunction & ETC	Uqcr10	-0.280595238	0.21486505	0.225604445
Mitochondrial dysfunction & ETC	Ogdh	-0.338456534	0.19731899	0.085725352
Mitochondrial dysfunction & ETC	Mt-Cyb	-0.374475377	0.21610158	0.177918502
Mitochondrial dysfunction & ETC	Prdx3	-0.25329084	0.10005445	-0.141110836
Mitochondrial dysfunction & ETC	Uqcrfs1	-0.342499561	0.37892931	0.04420095
Mitochondrial dysfunction & ETC	Ndufa11	-0.057158299	0.2741639	0.064852061
Mitochondrial dysfunction & ETC	Cox6a2	-0.130928788	0.34895329	-0.078627676
Mitochondrial dysfunction & ETC	Ndufa5	0.190240987	0.75908519	0.51854323
Mitochondrial dysfunction & ETC	Ndufb3	0.101270491	0.56716473	0.532296549
Mitochondrial dysfunction & ETC	Ndufb4	0.226469912	0.49121644	0.463203525
Mitochondrial dysfunction & ETC	Sdha	0.151719839	0.47704741	0.527227915
Mitochondrial dysfunction & ETC	Uqcrc2	0.183251294	0.3227749	0.260628813
Mitochondrial dysfunction & ETC	Cox6a1	0.100211215	0.34608561	0.314209121
Mitochondrial dysfunction & ETC	Ndufv3	0.080296356	0.3805675	0.302224145
Mitochondrial dysfunction & ETC	Sdhc	0.091528012	0.35688214	0.286658344
Mitochondrial dysfunction & ETC	Uqcrh	0.087822972	0.46049849	0.409132104
Mitochondrial dysfunction & ETC	Park7	0.065003677	0.51971613	0.277047997
Mitochondrial dysfunction & ETC	Ndufb10	0.036355755	0.42472845	0.275515321
Mitochondrial dysfunction & ETC	Ndufs8	-0.083493427	0.15051116	0.546753956
Mitochondrial dysfunction & ETC	Ndufa13	0.18272377	0.29816036	0.449807656
Mitochondrial dysfunction & ETC	Ndufs2	0.172554076	0.16347269	0.327523467
Mitochondrial dysfunction & ETC	Cpt1b	-0.414757549	0.61550721	0.437423413
Mitochondrial dysfunction & ETC	Cox7a2	-0.109648202	0.88754579	0.231528664
Mitochondrial dysfunction & ETC	Ndufs6	0.001198164	0.68444591	0.362204437
Mitochondrial dysfunction & ETC	Ndufa3	-0.038260868	0.49382574	0.163560856
Mitochondrial dysfunction & ETC	Ndufb9	-0.108911906	0.51242131	0.295887296
Mitochondrial dysfunction & ETC	Atp5j	-0.057021439	0.57006789	0.31288049
Mitochondrial dysfunction & ETC	Pdha1	-0.149139915	0.35120979	0.292058781
Mitochondrial dysfunction & ETC	Uqcrq	-0.172635622	0.35392596	0.309472897
Mitochondrial dysfunction & ETC	Ndufs5	-0.188965726	0.35937909	0.231479138
Mitochondrial dysfunction & ETC	Ndufb5	-0.152707478	0.33806597	0.253838809
Mitochondrial dysfunction & ETC	Ndufv1	-0.119021067	0.34734585	0.239744839

Mitochondrial dysfunction & ETC	Fis1	-0.09747299	0.18800594	0.325790837
Mitochondrial dysfunction & ETC	Ndufa4	-0.066740322	0.27099467	0.240433257
Mitochondrial dysfunction & ETC	Atp5c1	-0.145649821	0.26541758	0.263015377
Mitochondrial dysfunction & ETC	Atp5a1	-0.140115531	0.30005964	0.239464387
Mitochondrial dysfunction & ETC	Prdx5	-0.08152245	0.35173229	0.269744247
Mitochondrial dysfunction & ETC	Mtnd4	-0.060941797	0.36492497	0.251094439
Mitochondrial dysfunction & ETC	Cox4i1	-0.111865428	0.3973642	0.23228529
Mitochondrial dysfunction & ETC	Uqcr11	-0.044758122	0.40441185	0.237105527
Mitochondrial dysfunction & ETC	Sdhb	-0.090786643	0.38679519	0.316041125
Mitochondrial dysfunction & ETC	Cox6b1	-0.112864501	0.37421135	0.321036186
Mitochondrial dysfunction & ETC	Ndufs4	-0.111138061	0.43461228	0.327754413
Mitochondrial dysfunction & ETC	Ndufa6	-0.069215247	0.42771756	0.297838156
Mitochondrial dysfunction & ETC	Ndufb6	-0.068110849	0.44411161	0.31131019
Mitochondrial dysfunction & ETC	Ndufs3	-0.201681076	0.37492964	0.468472421
Mitochondrial dysfunction & ETC	Ndufa2	0.003544919	0.44217789	0.379763016
Mitochondrial dysfunction & ETC	Cyc1	-0.031581101	0.420423	0.389291089
Mitochondrial dysfunction & ETC	Uqcrc1	-0.025423434	0.35881212	0.375001381
Mitochondrial dysfunction & ETC	Ndufb7	-0.086481659	0.38302315	0.394972208
Mitochondrial dysfunction & ETC	Ndufb11	-0.295665944	0.35702469	-1.649667236
Mitochondrial dysfunction & ETC	Cat	0.447947747	-0.24048712	-0.733510535
Mitochondrial dysfunction & ETC	Ndufa8	-0.067586304	0.14533075	-0.921113116
Mitochondrial dysfunction & ETC	Cox8b	-2.608634743	-0.24632444	0.849391643
Mitochondrial dysfunction & ETC	Hsd17b10	0.537293754	-2.27461999	-0.734739889
Mitochondrial dysfunction & ETC	Cox7c	0.049046019	-1.10357116	-0.794922225
Mitochondrial dysfunction & ETC	Maob	-1.27682433	-1.59582344	0.765863657
Mitochondrial dysfunction & ETC	Cox5b	-0.56602402	-1.03391282	0.126252565
Mitochondrial dysfunction & ETC	Cox5a	-0.84945402	-0.36166231	-0.379755626
Mitochondrial dysfunction & ETC	Mtco2	-1.051824896	-1.02450032	-0.773874504
Glycolysis and Gluconeogenesis	Eno1	0.221013679	0.48495045	0.284326783
Glycolysis and Gluconeogenesis	Pfkl	0.261725448	0.49946272	0.388448584
Glycolysis and Gluconeogenesis	Rdh14	0.244181739	0.52227641	0.375354177
Glycolysis and Gluconeogenesis	Akr1a1	0.009872254	0.65576268	-0.023598906
Glycolysis and Gluconeogenesis	Gapdh	0.018021978	0.29795041	0.029507541
Glycolysis and Gluconeogenesis	Aldh2	-0.22342203	0.40102175	0.022595176
Glycolysis and Gluconeogenesis	Mdh1	-0.151837342	0.42787323	0.064540691
Glycolysis and Gluconeogenesis	Tpi1	-0.007246195	0.48226379	0.270488195
Glycolysis and Gluconeogenesis	Fbp2	-0.009447993	0.43962492	0.25044218
Glycolysis and Gluconeogenesis	Aldh4a1	-0.036801402	0.45634457	0.25133367
Glycolysis and Gluconeogenesis	Mtap	-0.047730401	0.44535177	0.24016615
Glycolysis and Gluconeogenesis	Pgam1	0.080194027	0.50019493	0.324957461
Glycolysis and Gluconeogenesis	Pgm2	0.021872272	0.45360172	0.313119224
Glycolysis and Gluconeogenesis	Pgk1	-0.037075534	0.45875698	0.31490659
Glycolysis and Gluconeogenesis	Gpi	-0.011209663	0.42206317	0.326812171
Glycolysis and Gluconeogenesis	Pkm	-0.099154976	0.19987586	0.239588447
Glycolysis and Gluconeogenesis	Pfkm	-0.228655768	0.19212624	0.419891098
Glycolysis and Gluconeogenesis	Eno3	-0.103137738	0.38098594	0.370555137
Glycolysis and Gluconeogenesis	Eno2	-0.146543654	0.30856675	0.403791457
Glycolysis and Gluconeogenesis	Adhfe1	-0.970911153	0.64288204	0.3548401
Glycolysis and Gluconeogenesis	Acsl1	0.27989309	0.74237494	0.981667079
Glycolysis and Gluconeogenesis	Pygb	-0.386121448	0.72205541	0.55842322
Glycolysis and Gluconeogenesis	Pygm	-0.085938058	0.73226708	0.560935264

Glycolysis and Gluconeogenesis	Agl	-0.098032076	0.68001351	0.305582662
Glycolysis and Gluconeogenesis	Hsd17b10	0.537293754	-2.27461999	-0.734739889
Glycolysis and Gluconeogenesis	Dhrs4	-0.077778147	-0.47406981	0.270615176
Glycolysis and Gluconeogenesis	Me1	-0.036714664	-0.48215521	0.41305613
Glycolysis and Gluconeogenesis	Adh5	-0.655510988	0.2689981	-0.485410159
Glycolysis and Gluconeogenesis	Acss1	-0.185137368	-0.05702944	-0.531313876
Glycolysis and Gluconeogenesis	Mdh2	-0.080535103	0.36427682	-0.406507927
TCA cycle	Sdhd	0.375112814	1.81967524	-0.788301437
TCA cycle	Dld	-0.858571075	0.38586686	-0.322800108
TCA cycle	Mdh2	-0.080535103	0.36427682	-0.406507927
TCA cycle	Fh	-0.326815074	0.25175606	-0.328078525
TCA cycle	Got2	-0.098436064	-0.02280278	-0.149028597
TCA cycle	Mdh1	-0.151837342	0.42787323	0.064540691
TCA cycle	Ogdh	-0.338456534	0.19731899	0.085725352
TCA cycle	Suclg1	-0.167539265	0.12999275	0.116772721
TCA cycle	Idh3a	-0.525540583	0.45053973	0.263925909
TCA cycle	Idh3g	-0.31014809	0.4025738	0.24803766
TCA cycle	Idh3b	-0.24950466	0.53263356	0.333756098
TCA cycle	Got1	0.022104019	0.50172125	0.340999902
TCA cycle	Sdhc	0.091528012	0.35688214	0.286658344
TCA cycle	Sucla2	-0.09234239	0.42699089	0.292045622
TCA cycle	Sdhb	-0.090786643	0.38679519	0.316041125
TCA cycle	Dlst	-0.140065714	0.28601421	0.384661157
TCA cycle	Aco2	-0.096780244	0.42302429	0.463442099
TCA cycle	Cs	0.103048108	0.87835446	-0.141893906
TCA cycle	Ogdhl	0.388930411	0.69898293	0.601522274
TCA cycle	Aco1	0.258611304	0.55766974	0.396227127
TCA cycle	Sdha	0.151719839	0.47704741	0.527227915
BCAA metabolism	Acadm	-0.835758878	0.11063688	0.248137291
BCAA metabolism	Bcat2	-0.758099933	0.38263567	0.224678469
BCAA metabolism	Hibadh	-0.580852653	0.12820886	-0.654536847
BCAA metabolism	Dld	-0.858571075	0.38586686	-0.322800108
BCAA metabolism	Hadha	-0.079186016	0.33389884	-0.090828055
BCAA metabolism	Aldh6a1	-0.210011003	0.34101162	0.085385993
BCAA metabolism	Bckdhb	-0.147340243	0.68164262	0.350470766
BCAA metabolism	Hmgcl	-0.100978502	0.64556233	0.184235028
BCAA metabolism	Hadhb	-0.085483038	0.51076847	0.138241501
BCAA metabolism	Mccc1	-0.279036024	0.45038796	0.388276815
BCAA metabolism	Echs1	0.004064143	0.39170605	0.334978413
BCAA metabolism	Acat1	-0.077784501	0.31295294	0.278896982
BCAA metabolism	Dbt	-0.165812817	0.41842529	0.281009164
BCAA metabolism	Hibch	-0.123906564	0.36297086	0.335005857
BCAA metabolism	Ivd	-0.168370595	-0.18945821	0.161825792
BCAA metabolism	Acad8	0.351563722	0.20179281	-0.437513166
BCAA metabolism	Hsd17b10	0.537293754	-2.27461999	-0.734739889
BCAA metabolism	Mccc2	2.117435912	0.46184097	1.096289457
BCAA metabolism	Bckdha	0.420975217	1.68396796	0.985932048
2-oxobutanoate	Bckdha	0.420975217	1.68396796	0.985932048
2-oxobutanoate	Dld	-0.858571075	0.38586686	-0.322800108
2-oxobutanoate	Pccb	-0.235134436	0.53975517	0.689075139
2-oxobutanoate	Pcca	-0.004734122	0.84174169	0.709710569

2-oxobutanoate	Mut	0.373101428	0.76525693	0.161058141
2-oxobutanoate	Mcee	-0.267398932	0.43746272	-0.070097319
2-oxobutanoate	Dbt	-0.165812817	0.41842529	0.281009164
2-oxobutanoate	Bckdhb	-0.147340243	0.68164262	0.350470766
Acetyl-CoA	Pdhb	-0.309966992	0.26162808	0.324336461
Acetyl-CoA	Pdhx	-0.075436405	0.3066024	0.435684907
Acetyl-CoA	Dbt	-0.165812817	0.41842529	0.281009164
Acetyl-CoA	Pdha1	-0.149139915	0.35120979	0.292058781
Acetyl-CoA	Dld	-0.858571075	0.38586686	-0.322800108
Acetyl-CoA	Dlat	-0.36538043	0.26311928	-0.332326914
Acetyl-CoA	Gstz1	-0.225955186	-1.48576445	0.293767308
Oxidative stress	Gstk1	0.138666698	-1.18555253	0.322204716
Oxidative stress	Nqo2	0.250730347	-1.37052072	0.320323096
Oxidative stress	Txn	-1.246224346	-0.65441474	-0.710704457
Oxidative stress	Mgst3	-0.029214964	-0.09224031	-0.167375194
Oxidative stress	Cat	0.447947747	-0.24048712	-0.733510535
Oxidative stress	Sod2	1.660186104	2.03542452	1.967785463
Oxidative stress	Gsta4	-0.112954732	0.40295726	0.141415447
Oxidative stress	Gstm2	-0.1868976	0.35531945	-0.001858129
Oxidative stress	Akr7a2	-0.114884693	0.62759905	0.528304914
Oxidative stress	Nqo1	-0.049352168	0.39052943	0.275775127
Oxidative stress	Sod1	-0.120656771	0.46715852	0.334115301
Oxidative stress	Gsto1	0.03266651	0.5238142	0.175904069
Oxidative stress	Prdx1	0.009118844	0.44296151	0.223918685
Oxidative stress	Gstp1	0.00669378	0.49263842	0.276147155
Oxidative stress	Gpx3	0.022461449	0.48002713	0.295065487
Oxidative stress	Fth1	0.199351693	0.60986528	0.329828315
Oxidative stress	Vcp	0.094428569	0.28789826	0.281735807
Oxidative stress	Cct7	0.168323397	0.41496099	0.18354893
Oxidative stress	Cbr1	0.104990396	0.47297882	0.184732187
Oxidative stress	Gstm1	0.752355802	1.26272259	0.321224651
Oxidative stress	Txnrd1	-0.084557818	1.06299277	0.067837375
Oxidative stress	Gstm5	-0.376153716	0.92623972	0.292679814
Oxidative stress	Ftl1	0.141404252	0.70383863	0.018948779
Oxidative stress	Akr1a1	0.009872254	0.65576268	-0.023598906
Oxidative stress	Gpx1	0.507682391	0.50778972	0.121099247
Oxidative stress	Stip1	0.324404918	0.50742151	-0.038479814
Ketolysis and ketgenesis	Bdh1	0.324464034	2.20047674	1.033530383
Ketolysis and ketgenesis	Hadhb	-0.085483038	0.51076847	0.138241501
Ketolysis and ketgenesis	Oxct1	-0.120384998	0.65137636	0.194459806
Ketolysis and ketgenesis	Hmgcl	-0.100978502	0.64556233	0.184235028
Ketolysis and ketgenesis	Hadha	-0.079186016	0.33389884	-0.090828055
Ketolysis and ketgenesis	Acat1	-0.077784501	0.31295294	0.278896982
Ethanol degradation	Hsd17b10	0.537293754	-2.27461999	-0.734739889
Ethanol degradation	Acsl1	0.27989309	0.74237494	0.981667079
Ethanol degradation	Akr1a1	0.009872254	0.65576268	-0.023598906
Ethanol degradation	Aldh4a1	-0.036801402	0.45634457	0.25133367
Ethanol degradation	Aldh2	-0.22342203	0.40102175	0.022595176
Ethanol degradation	Rdh14	0.244181739	0.52227641	0.375354177
Ethanol degradation	Adh1	0.0858411	0.16644423	0.259354016
Ethanol degradation	Adhfe1	-0.970911153	0.64288204	0.3548401

Ethanol degradation	Aldh1b1	-0.909189204	0.58841111	-0.144658346
Ethanol degradation	Dhrs4	-0.077778147	-0.47406981	0.270615176
Ethanol degradation	Acss1	-0.185137368	-0.05702944	-0.531313876
Fatty acid oxidation	Adh5	-0.655510988	0.2689981	-0.485410159
Fatty acid oxidation	Hsd17b10	0.537293754	-2.27461999	-0.734739889
Fatty acid oxidation	Eci1	0.568857548	1.11445985	1.243266033
Fatty acid oxidation	Acsl1	0.27989309	0.74237494	0.981667079
Fatty acid oxidation	Ivd	-0.168370595	-0.18945821	0.161825792
Fatty acid oxidation	Acadm	-0.835758878	0.11063688	0.248137291
Fatty acid oxidation	Hadha	-0.079186016	0.33389884	-0.090828055
Fatty acid oxidation	Acaa2	-0.060859339	0.33806116	-0.256711028
Fatty acid oxidation	Hadhb	-0.085483038	0.51076847	0.138241501
Fatty acid oxidation	Eci2	-0.091028542	0.40645182	0.240868955
Fatty acid oxidation	Echs1	0.004064143	0.39170605	0.334978413
Aryl hydrocarbon& receptor	Gstk1	0.138666698	-1.18555253	0.322204716
Aryl hydrocarbon& receptor	Nqo2	0.250730347	-1.37052072	0.320323096
Aryl hydrocarbon& receptor	Ctsd	-1.166069217	0.60781678	0.057188294
Aryl hydrocarbon& receptor	Aldh1b1	-0.909189204	0.58841111	-0.144658346
Aryl hydrocarbon& receptor	Aldh5a1	-0.766308439	-0.30743901	-0.341578643
Aryl hydrocarbon& receptor	Tgm2	-0.804765695	0.15011518	-0.62919399
Aryl hydrocarbon& receptor	Atm	0.479293884	0.97281634	-0.1183141
Aryl hydrocarbon& receptor	Gstm1	0.752355802	1.26272259	0.321224651
Aryl hydrocarbon& receptor	Hsp90b1	0.423882473	0.69835136	0.610560102
Aryl hydrocarbon& receptor	Hsp90aa1	0.121863577	-0.04995193	0.387302268
Aryl hydrocarbon& receptor	Cdk4	-0.081643194	0.25213981	0.502756176
Aryl hydrocarbon& receptor	Hsp90ab1	0.106492158	0.47118458	0.718464727
Aryl hydrocarbon& receptor	Mgst3	-0.029214964	-0.09224031	-0.167375194
Aryl hydrocarbon& receptor	Gstm2	-0.1868976	0.35531945	-0.001858129
Aryl hydrocarbon& receptor	Aldh6a1	-0.210011003	0.34101162	0.085385993
Aryl hydrocarbon& receptor	Gstm5	-0.376153716	0.92623972	0.292679814
Aryl hydrocarbon& receptor	Hspb1	-0.129631076	0.53149292	0.356967396
Aryl hydrocarbon& receptor	Gsto1	0.03266651	0.5238142	0.175904069
Aryl hydrocarbon& receptor	Nqo1	-0.049352168	0.39052943	0.275775127
Aryl hydrocarbon& receptor	Aldh4a1	-0.036801402	0.45634457	0.25133367
Aryl hydrocarbon& receptor	Gstp1	0.00669378	0.49263842	0.276147155
Methylglyoxal degradation	Akr1a1	0.009872254	0.65576268	-0.023598906
Methylglyoxal degradation	Akr7a2	-0.114884693	0.62759905	0.528304914
Methylglyoxal degradation	Akr1b7	0.101151163	0.25289765	0.353127126
Methylglyoxal degradation	Akr1b1	-0.119850607	0.40944815	0.2842638
Methylglyoxal degradation	Akr1b8	-0.103322776	0.31247119	0.214396825
Protein ubiquitination	Psma6	-0.747137022	0.18380093	0.560460707
Protein ubiquitination	Cryab	-0.446598931	0.70878983	0.546383218
Protein ubiquitination	Hspe1	-0.771411071	0.61354387	0.4535054
Protein ubiquitination	Usp9x	-0.902927542	-0.03537421	-0.287921076
Protein ubiquitination	Psmb6	-1.033322438	-0.72040964	-0.228050857
Protein ubiquitination	Psmd14	-0.07914502	0.81201492	0.546703682
Protein ubiquitination	Hspb6	-0.136132579	0.80158003	0.666461518
Protein ubiquitination	Psmd3	0.289334136	0.65412724	0.81354833
Protein ubiquitination	Hsp90b1	0.423882473	0.69835136	0.610560102
Protein ubiquitination	Hsp90ab1	0.106492158	0.47118458	0.718464727
Protein ubiquitination	Psma5	0.150616689	0.58549489	0.541244015

Protein ubiquitination	Psmb1	0.122684884	0.67338525	0.58862961
Protein ubiquitination	Hspa5	0.06156991	0.77395953	0.28256235
Protein ubiquitination	Psme2	0.299249292	0.51349727	0.205642865
Protein ubiquitination	Psmd11	0.170186518	0.55051682	0.400432766
Protein ubiquitination	Usp5	0.059085316	0.50611895	0.253469182
Protein ubiquitination	Hspd1	0.026604565	0.49976949	0.335240722
Protein ubiquitination	Psmb3	0.105295	0.46803725	0.345727542
Protein ubiquitination	Psma4	-0.222356147	0.44523774	-0.222281184
Protein ubiquitination	Psma2	0.121952986	0.30233361	0.3135944
Protein ubiquitination	Hspa9	0.088465692	0.1971167	0.18439122
Protein ubiquitination	Ube2l3	-0.050869413	0.33106732	0.055682663
Protein ubiquitination	Hspa4	-0.116239721	0.28078225	0.136254624
Protein ubiquitination	Hspb1	-0.129631076	0.53149292	0.356967396
Protein ubiquitination	Uba1	-0.167114636	0.4022531	0.213859966
Protein ubiquitination	Psmb7	0.43209111	-0.70185963	0.290648459
Protein ubiquitination	Hspa8	0.026574651	0.11338666	0.637309324
Protein ubiquitination	Psmc3	-0.02914132	-0.01906041	0.262137909
Protein ubiquitination	Hsp90aa1	0.121863577	-0.04995193	0.387302268

STAB. 6 Metabolic profiling

Metabolites	ORP/OCL	p-value
**Glycolysis**		
Glucose	1.06 ± 0.181	0.5624
G1P/G6P	0.452 ± 0.233	0.0499
F6P/F1P	0.450 ± 0.231	0.0460
F16BP/F26BP	0.508 ± 0.494	0.2262
D-GA3P	0.697 ± 0.242	0.1502
DHAP	0.690 ± 0.253	0.1541
2/3-Phosphoglyceric Acid	0.853 ± 0.527	0.7120
PEP	1.081 ± 0.820	0.8625
Pyruvate	1.017 ± 0.326	0.9161
**TCA cycle**		
Citric Acid	1.348 ± 0.140	0.0146
α-Ketoglutaric Acid	1.341 ± 0.139	0.0010
Succinate	1.353 ± 0.371	0.0928
Fumaric	1.411 ± 0.196	0.0076
Malate	1.317 ± 0.193	0.0207
Oxaloacetate	1.315 ± 0.261	0.0772
